# Late-stage trifluoromethylthiolation of benzylic C-H bonds

**DOI:** 10.1038/s41467-019-12844-9

**Published:** 2019-10-25

**Authors:** Wentao Xu, Wenliang Wang, Tao Liu, Jin Xie, Chengjian Zhu

**Affiliations:** 1 State Key Laboratory of Coordination Chemistry, Jiangsu Key Laboratory of Advanced Organic Materials, Chemistry and Biomedicine Innovation Center (ChemBIC), School of Chemistry and Chemical Engineering, Nanjing University, 210023 Nanjing, China; 2 State Key Laboratory of Organometallic Chemistry, Shanghai Institute of Organic Chemistry, 200032 Shanghai, China

**Keywords:** Photocatalysis, Organic chemistry, Synthetic chemistry methodology

## Abstract

The benzylic positions in drugs are sites that readily react with cytochrome P450 oxidases via single-electron oxidation. New synthetic methodologies to incorporate a fluoroalkyl group at the benzylic site are continually being developed, and in this paper, we report a metal-free and site-selective organophotoredox-catalyzed trifluoromethylthiolation of benzylic C-H bonds for a wide variety of alkyl arenes and heteroarenes. The precise and predictive regioselectivity among various C(sp^3^)-H bonds originates from an inner-sphere benzylic radical initiation mechanism, and avoids the use of external oxidants or hydrogen atom abstractors. Its practicality stems from the trifluoromethylthiolation of a series of drugs and complex organic molecules, which is overwhelmingly selective for benzyl groups. This operationally simple protocol can provide a general and practical access to structurally diverse benzylic trifluoromethyl sulfides produced from ubiquitous benzylic C-H bonds. Large scale trifluoromethylthiolation can be achieved with continuous flow photoredox technology.

## Abstract (web summary)

Fluoroalkylation of C-H bonds is a class of reaction highly sought after in medicinal chemistry. Here, the authors report a regioselective organophotoredox-catalyzed, trifluoromethylthiolation of benzylic C-H bonds for a wide variety of alkyl (hetero)arenes, and demonstrate its utility in continuous flow.

## Introduction

**Fig. 1:**
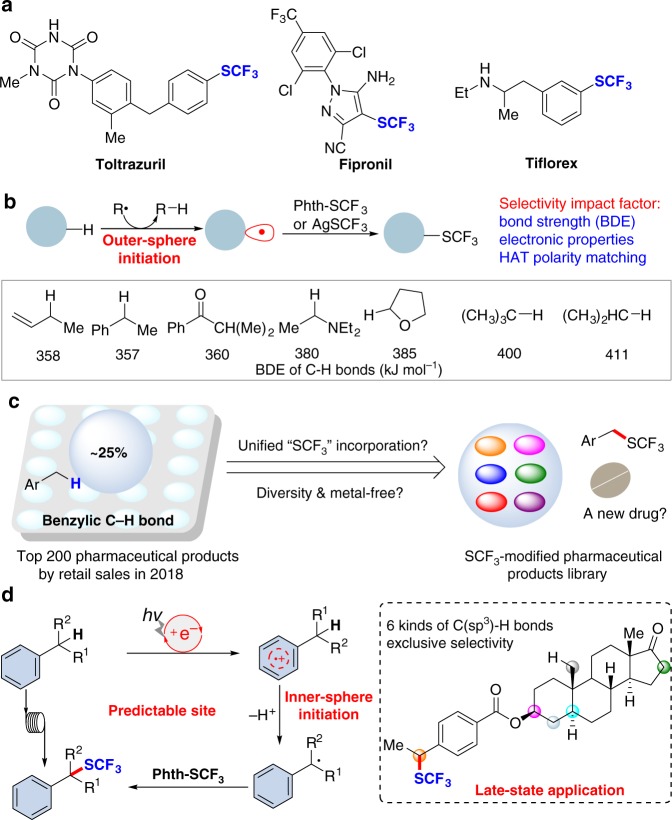
The state-of-the-art strategies of precise C–H trifluoromethylthiolation. **a** The importance of trifluoromethylthiolated drugs in the market. **b** Previous strategies for C(sp^3^)–H bond trifluoromethylthiolation via generation of key alkyl radicals by an outer-sphere radical initiation process. **c** The prevalence of the benzylic moiety in biologically important compounds. **d** Our work via inner-sphere radical initiation for precise benzylic C–H bond trifluoromethylthiolation

Challenges in modern drug discovery have played an important role in the evolution of direct synthetic methodology^1–6^. The cytochrome P450 enzymatic metabolism of therapeutics in vivo is a common route of drug metabolism^7^. Due to the unique electron-negativity and high lipophilicity, the introduction of a trifluoromethylthio group (SCF_3_) into pharmaceutical candidates can significantly protect against in vivo enzymatic metabolism and increase the cell membrane permeability^8^. As a consequence, the development of organic synthetic strategies accessing trifluoromethylthiolated compounds has gained considerable momentum in recent years^9–15^. Although several trifluoromethylthiolated drugs (e.g., tiflorex, toltrazuril and tiflorex in Fig. 1a) have been approved by FDA, the future development of such compounds depends on the evolution of synthetic strategies entailing versatility, diversity and availability.

Direct radical trifluoromethylthiolation of C(sp^3^)–H bonds can provide a powerful platform with which to construct organofluorine compounds^16–19^. Seminal work from Qing and co-workers^16^, Chen and co-workers^17^, Tang and co-workers^18^, and Glorius and co-workers^19^ has significantly stressed the potential synthetic value of such reactions. Currently, the regioselectivity of the reaction relies mainly on the physicochemical properties (e.g., exchange constants and polarity) of intermolecular hydrogen-atom-transfer (HAT) reagents or oxidants in terms of C–H bond dissociation energy and electronic properties (Fig. 1b)^20^. However, based on this outer-sphere radical initiation mechanism, it is still rather difficult to predict the regioselectivity precisely, especially for the complex organic molecular architectures bearing nearly resembling C(sp^3^)–H bonds shown in Fig. 1b.

Benzylic C–H bonds are common in biologically important compounds and about 25% of top-selling 200 pharmaceuticals contain this structural motif ^21^. In general, the benzylic positions in small-molecule drugs are sites easily metabolized by cytochrome P450 oxidases. If a metal-free and unified benzylic C–H bond trifluoromethylthiolation strategy is available, drug discovery across of a wide range of structurally diversity compounds could be expedited (Fig. 1c). Although benzylic radical C–H bond functionalization has been well studied^22–26^, the late-stage benzylic C–H trifluoromethylthiolation strategy remains a significant obstacle. The use of strong external oxidants will result in rapid oxidation of benzylic C–H bonds into a carbonyl unit^27^. The only successful benzylic C–H trifluoromethylthiolation strategy is copper-catalyzed oxidative trifluoromethylthiolation with AgSCF_3_ but the reaction needs a large excess of simple toluene analogs (60 equivalents compared to AgSCF_3_), and thus this compromises its practicability and late-stage potential^16^. To achieve an exclusive benzylic C–H regioselectivity, the best approach may be the generation of a benzylic radical without involving an intermolecular HAT process. This leads to consideration of single-electron oxidation of phenyl rings. As shown in Fig. 1d, the resultant aryl radical cation species^28–36^ will lead to inner-sphere HAT with benzylic C–H bonds giving rise to benzylic radicals. Herein, we report the development of a metal-free, photoredox inner-sphere HAT process which predictably generates, from natural products or drug derivatives, a benzylic radical which can be trifluoromethylthiolated, avoiding the use of oxidants and HAT reagents. The practicality of the method is further illustrated by late-stage trifluoromethylthiolation of benzylic C–H bonds under flow photochemical conditions.

## Results

### Reaction optimization

To test our hypothesis, 2-isopentylbenzo[b]thiophene with Phth-SCF_3_ (2-((trifluoromethyl)thio)isoindoline-1,3-dione) was chosen as the model reaction since the electron-rich thiophene ring can usually support electrophilic trifluoromethylthiolation^37,38^. As shown in Table 1, both solvent and photocatalyst are crucial factors for successful benzylic C–H trifluoromethylthiolation. Among the solvents examined, only MeCN could produce a 75% yield of **3a**. Other solvents gave only trace amounts of the desired product with commercially available 4CzIPN (2,4,5,6-tetra(9*H*-carbazol-9-yl)isophthalonitrile) as the photocatalyst (Table 1, entries 1–5). Notably, the reaction is highly regioselective (benzylic vs methine C–H, see Supplementary Fig. 9)^19^. Replacement of 4CzIPN with other photocatalysts delivered a much lower yield (Table 1, entries 5–10). The amount of Phth-SCF_3_ was further decreased from 1.5 equiv to 1.3 equiv by careful evaluation of reaction and base concentrations, affording the desired product (**3a**) in 81% yield with 98:2 regioselectivity ratio (Table 1, entry 11). Compared with previous work^39^, the use of a slight excess (1.3 equiv) of a trifluoromethylthiolated reagent enhances its synthetic value. In the absence of K_2_CO_3_, a 32% yield of **3a** along with a decreased ratio of 88:12 was obtained (Table 1, entry 12). The role of inorganic base may benefit the deprotonation, generating a benzylic radical species. Control experiments suggested that both photocatalyst and light were crucial for benzylic C(sp^3^)–H trifluoromethylthiolation (Table 1, entries 13–14).

**Table 1:** Optimization of reaction conditions


Entry	PC	Solvent	Yield (%)	3a:3a′
1	4CzIPN	DCM	Trace	–
2	4CzIPN	MeOH	Trace	–
3	4CzIPN	DMF	Trace	–
4	4CzIPN	THF	Trace	–
5	4CzIPN	MeCN	75	97:3
6	Ir[dF(CF_3_)(ppy)]_2_(dtbbpy)PF_6_	MeCN	2	1:1
7	Ru(bpz)_3_(PF_6_)_2_	MeCN	–	–
8	Acr-Mes^+^ ClO_4_^−^	MeCN	Trace	–
9	4CzPN	MeCN	46	92:8
10	DCA	MeCN	32	86:14
11^a^	4CzIPN	MeCN	81 (73)	98:2
12^a,b^	4CzIPN	MeCN	32	88:12
13^a,c^	4CzIPN	MeCN	Trace	–
14^a,d^	4CzIPN	MeCN	0	–

Reaction conditions: **1a** (0.1 mmol), Phth-SCF_3_ (1.5 equiv), PC (2 mol%), K_2_CO_3_ (0.1 equiv), anhydrous MeCN (2 mL), 45 W blue LEDs, 12 h ^a^**1a** (0.2 mmol), K_2_CO_3_ (0.2 equiv), Phth-SCF_3_ (1.3 equiv), anhydrous MeCN (4 mL) ^b^No K_2_CO_3_^c^No photocatalyst. ^d^Under dark condition. The number in parentheses is the isolated yield

### Substrate scope

**Fig. 2:**
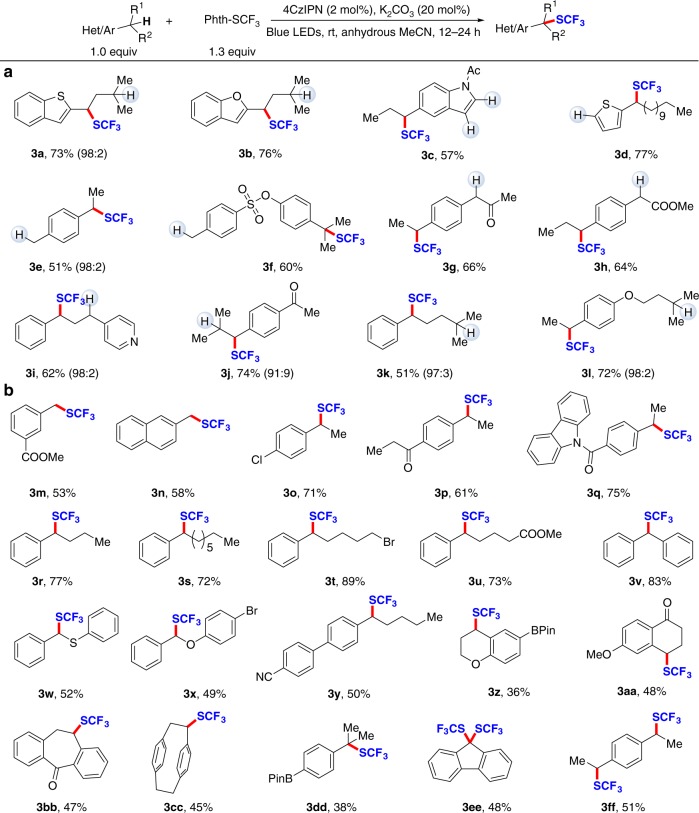
The reaction scope. **a** Substrates with the heteroaromatic ring or competing C–H bond. **b** Substrates with 1°, 2°, 3° benzylic C–H bond. Yields of isolated products are given. The ratio of **3**/**3**′ is given in the parenthesis. **3i** contains a small amount of raw material **1i**, due to the similar Rf value

With the optimal reaction conditions in hand, we investigated the scope of aromatic hydrocarbons (Fig. 2). Several electron-rich five-membered ring heteroaromatic compounds, including thiophene-, furan-, and indole-based substrates (**3a–3d**) are compatible with the reaction. In general, it is challenging to realize site-selective benzylic C–H trifluoromethylthiolation of indole substrates (**3c**) due to the competing trifluoromethylthiolation at C3 position^40,41^. This further indicates the advantages of this inner-sphere radical initiation mechanism. When substrates contain more than one different benzylic C–H bond, the secondary (**3e**), tertiary (**3f**), and less acidic C–H bond (**3g, 3h**) were preferentially trifluoromethylthioylated. Interestingly, with 4-(3-phenylpropyl)-pyridine, excellent regioselectivity was observed, focusing on the C–H bond in the proximity of the more electron-rich phenyl ring rather than the pyridinyl ring (**3i**). Distinguishing between nearly identical C–H bonds with traditional outer-sphere HAT strategy remains a significant challenge. It has been reported that even the outer-sphere HAT mechanism can robustly achieve trifluoromethylthiolation of unactivated methine and methylene C(sp^3^)–H bonds while failing with benzylic C–H bonds^19^. In contrast, our strategy addresses this unresolved problem (**3j–3l**). Also, the predicted regioselectivity is obtained with substrates bearing competitive methine C–H and the α-C–H of a heteroatom (**3l**).

Subsequently, we examined the generality of this protocol with substrates containing primary, secondary, or tertiary benzylic C–H bonds. Substrates bearing electron-withdrawing functional groups on the phenyl rings tolerated the reaction conditions, furnishing the desired products (**3m, 3o–3q**) in satisfactory yields. A wide variety of alkyl arenes are good coupling partners and uniformly afford the desired products (**3r–3cc**) in moderate to good yields with exclusive regioselectivity. The length of an alkyl chain on the aromatic ring has no influence on the regioselectivity and reaction efficiency. Another advantage of this redox-neutral strategy is that it can successfully trifluoromethylthiolate easily oxidized diphenylmethanes and benzylic ethers (**3v–x**). Importantly, PinB-substituted alkyl arenes are tolerated, albeit with moderate isolated yields (**3z, 3dd**), and can participate in a variety of downstream diversification reactions. With 2.5 equivalents of Phth-SCF_3_ reagents, double trifluoromethylthiolation can be realized (**3ee, 3ff**). With fluorenes, this affords direct access to the *gem*-trifluoromethylthiolation product (**3ee**) in moderate yield.

### Late-stage and flow chemistry

**Fig. 3:**
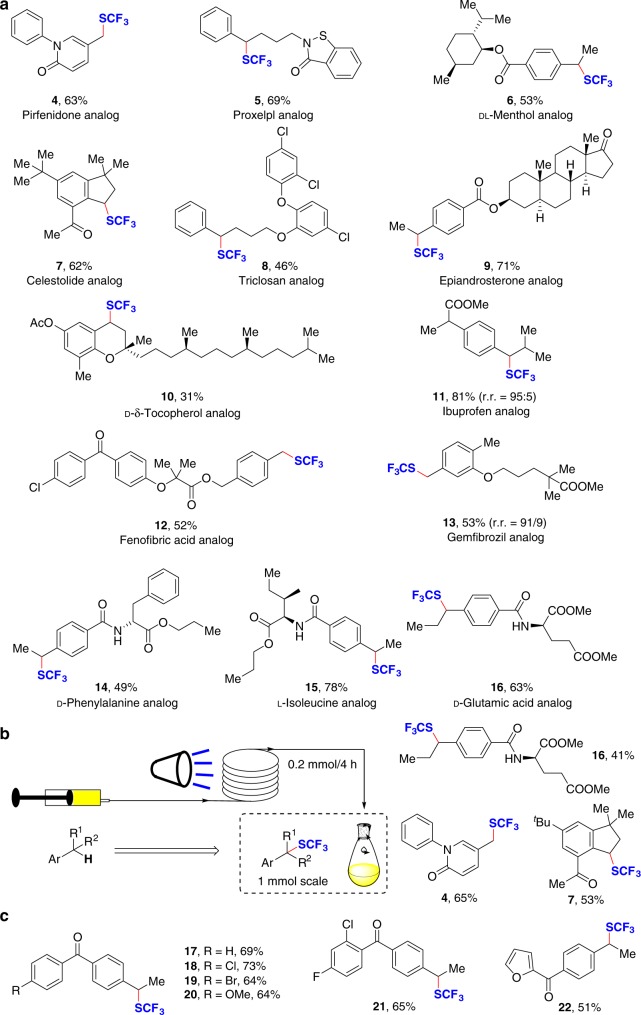
Synthetic application. **a** Late-stage benzylic C−H trifluoromethylthiolation of drugs and complex molecules. **b** Continuous-flow late-stage modification. **c** Synthesis of pesticide analogs

Distinct from previous outer-sphere radical initiation C–H trifluoromethylthiolation reactions, an inner-sphere mechanism can exclusively generate a benzylic radical without depending on external strong oxidants. To demonstrate the generality and practice of the reaction, we applied this redox-neutral strategy to achieve late-stage benzylic trifluoromethylthiolation of biologically important natural products and drugs, with an aim of modularly constructing trifluoromethylthioylated drug candidates (Fig. 3a). A variety of complex molecules can be trifluoromethylthioylated at the benzylic position in satisfactory yield with excellent functional group compatibility. The inner-sphere benzylic radical generation process allows competing C–H bonds little influence in complex molecules. For example, one of the top selling drugs, pirfenidone can undergo benzylic C–H trifluoromethylthiolation exclusively to give the desired product (**4**) in 63% yield. The methyl ester of ibuprofen, an anti-inflammatory drug can incur this transformation at the more electron-rich benzylic C–H bond in 81% yield (**11**). The less sterically hindered benzylic C–H bond in gemfibrozil and d-phenylalanine derivatives can be preferentially trifluoromethylthiolated (**13, 14**). Complex alkyl arenes bearing an amide NH moiety are also good substrates for highly site-selective benzylic C–H trifluoromethylthiolation (**14**–**16**), and late-stage trifluoromethylthiolation can be scaled up to 1 mmol with shorter reaction times by the use of continuous micro-tubing reactors^42,43^, thus enhancing its utility in synthetic applications (Fig. 3b).

### Synthetic application

A large number of benzyl sulfides were synthesized during research of pesticides^44^ and the benzophenone benzyl trifluoromethyl sulfide is a core substructure in such compounds. With our protocol, a series of benzyl trifluoromethyl sulfide analogs were successfully obtained from aryl (4-ethylphenyl)methanones in 51–73% yield under mild reaction conditions (Fig. 3c).

### Mechanistic studies

**Fig. 4:**
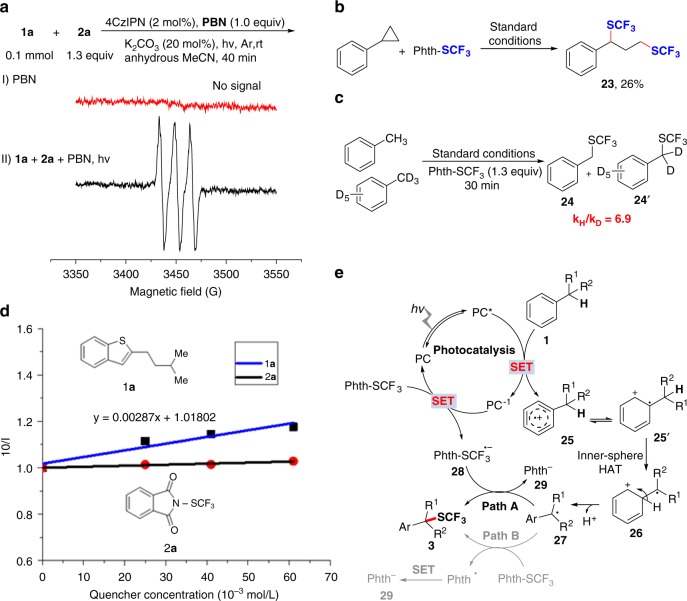
Mechanistic studies. **a** The EPR experiment. **b** The radical clock experiment. **c** KIE experiments. **d** The luminescence quenching experiment. **e** The proposed mechanism

To elucidate the possible reaction mechanism, electron paramagnetic resonance (EPR) experiments with *N*-*tert*-butyl-α-phenylnitrone (PBN) as the electron-spin trapping reagent were carried out. A significant EPR signal was observed for the model reaction, indicating a possible radical pathway (Fig. 4a). To further validate this, a radical clock experiment with cyclopropylbenzene as a substrate was performed and the results (Fig. 4b) clearly demonstrated the involvement of a benzylic radical. In addition, the KIE result (*k*_H_/*k*_D_ = 6.9) suggests that C–H cleavage is involved in the rate-determining step. Luminescence quenching experiments suggest that **1a** may be the quencher in the reductive quenching cycle (Fig. 4d). The quantum yield of a model reaction was determined to be 0.33 and thus a radical chain pathway is less likely.

Accordingly, a plausible mechanism is proposed and is shown in Fig. 4e. 4CzIPN is a commercially available organic photocatalyst, and it is reported that photoexcited 4CzIPN is a strong oxidant (^1/2^*E* = +1.35 V). Although alkyl arenes hold higher oxidative potential^45^, the potential overlap between the excited 4CzIPN and alkyl arenes could promote the single-electron oxidation for irreversible inner-sphere HAT as the driving force (see Supplementary Figs. 31 and 32)^46^. The aryl radical cation species that is formed (**25** or **25**′) will lead to an intramolecular 1,2-HAT with benzylic C–H bonds and subsequent deprotonation^47^ of cyclohexadienyl cation (**26**) with base gives rise to a benzylic radical (**27**). This benzylic radical can couple with a Phth-SCF_3_ anion radical (**28**) to deliver the desired benzylic trifluoromethylthiolation products (**3**) and a Phth anion (**29**). Generation of the Phth anion can further abstract one proton from the cyclohexadienyl cation (**26**) to yield phthalimide and this can explain why a catalytic amount of K_2_CO_3_ can initiate the reaction. Alternatively, the radical addition of benzylic radical (**27**) to electrophilic Phth-SCF_3_ reagent is also a likely candidate^19^ for the generation of trifluoromethylthiolation products (**3**).

## Discussion

In conclusion, we have developed an organophotoredox-catalyzed reaction for site-selective benzylic C–H bond trifluoromethylthiolation of a wide variety of alkyl arenes and heteroarenes through an inner-sphere radical initiation mechanism, affording structurally diverse benzylic trifluoromethyl sulfides with moderate to good yields. The broad scope, excellent functional group compatibility, and predictable regioselectivity allow for efficient late-stage benzylic C–H trifluoromethylthiolation of a variety of drug candidates and complex molecules. We believe that this strategy will expedite precise benzylic C–H functionalization in complex molecules and that it will promote the construction of a library of benzylic trifluoromethyl sulfide leads for drug discovery.

## Methods

### General procedure for benzylic trifluoromethylthiolation

Substrate **1** (0.2 mmol), Phth-SCF_3_ (64.3 mg, 0.26 mmol), 4CzIPN (3.2 mg, 0.004 mmol), and K_2_CO_3_ (5.52 mg, 0.04 mmol) were placed in a transparent Schlenk tube equipped with a stirring bar. The anhydrous MeCN (4.0 mL) was added under argon atmosphere. If the substrate **1** is liquid, anhydrous MeCN and **1** were added in turn. The reaction mixture was stirred under the irradiation of two 45 W blue LEDs (distance app. 4.0 cm from the bulb) at room temperature for 12–24 h. When the reaction finished, the mixture was quenched with water and extracted with ethyl acetate (3 × 10 mL). The organic layers were combined and concentrated under vacuo. The product was purified by flash column chromatography on silica gel (petroleum ether:ethyl acetate).

### General procedure for flow chemistry

Complex molecule (1.0 mmol), Phth-SCF_3_ (321 mg, 1.3 mmol), 4CzIPN (16 mg, 0.02 mmol), and K_2_CO_3_ powder (120 mesh, 27.6 mg, 0.2 mmol) were placed in a sample bottle (20 mL). After placing in the glove box, anhydrous MeCN (20.0 mL) was added and the yellow mixture was then transferred into the syringe (20 mL) in the glove box. Next, the reaction mixture was subjected to the irradiation of three 45 W blue LEDs (distance app. 4.0 cm) with a small fan at room temperature in the mode of perfusion/extraction at the speed of 2.0 mL/h. When the reaction finished, the mixture was quenched with water and extracted with ethyl acetate (3 × 10 mL). The organic layers were combined and concentrated under vacuo. The product was purified by flash column chromatography on silica gel (petroleum ether:ethyl acetate).

## Supplementary information

Supplementary Information

## Data Availability

The authors declare that all other data supporting the findings of this study are available within the article and Supplementary Information files, and also are available from the corresponding author upon reasonable request.
