# Supplementary material for: Late-stage trifluoromethylthiolation of benzylic C-H bonds
Source: Nat Commun. 2019 Oct 25;10:4867. doi: 10.1038/s41467-019-12844-9 (PMC6814834; doi:10.1038/s41467-019-12844-9)
Supplement: Supplementary file 1 — Supplementary Information [file 41467_2019_12844_MOESM1_ESM.pdf]

SUPPLEMENTARY INFORMATION

Late-Stage Trifluoromethylthiolation of Benzylic C-H Bonds

Xu et al.

Supplementary Methods

General information

All the reactions were conducted in oven-dried Schlenk tubes under Argon atmosphere unless otherwise noted. All solvents were obtained from commercial suppliers and used without further purification. Anhydrous MeCN was purified from MeCN (≥99.9%, HPLC) by Solvent Purification System. Reagents were purchased from Energy Chemical, Adamas-beta, and etc. Flash column chromatographic purification of products was accomplished using forced-flow chromatography on Silica Gel (300-400 mesh).

^{1}H NMR, ^{13}C NMR and ^{19}F NMR spectra were recorded on a 400 or 500 MHz spectrometer in $CDCl_3$ (δH = 0.0 ppm, δC = 77.02 ppm as standard). ^{19}F spectra were calibrated in relation to the reference measurement of 1,2-difluorobenzene (- 139 ppm). Data for ^{1}H NMR are reported as follows: chemical shift (ppm, scale), multiplicity, coupling constant (Hz), and integration. Data for ^{13}C NMR are reported in terms of chemical shift (ppm, scale), multiplicity, and coupling constant (Hz). The selectivity of trifluoromethylthiolative products was determined by GC-MS. Gas chromatographic (GC) analyses were performed on a GC equipped with a flameionization detector and an Rtx@-65 (30 m × 0.32 mm ID × 0.25 μm df) column. GC-MS analyses were performed on a GC-MS with an EI mode. High-resolution mass spectra were obtained by ESI on a TOF mass analyzer. And the 45 W blue LEDs light was purchased from Kessil (A360NE/WE).

Optimization of the Trifluoromethylthiolation

Supplementary Table 1. Optimization of reaction conditions[a]

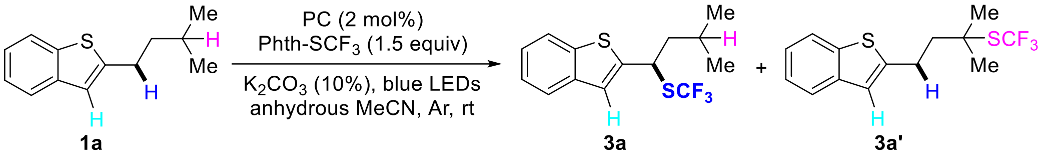

entry	PC	solvent	yield (%)	**3a:3a'**
1	4CzIPN	DCM	trace	-
2	4CzIPN	MeOH	trace	-
3	4CzIPN	DMF	trace	-
4	4CzIPN	THF	trace	-
5	4CzIPN	MeCN	75	97:3
6	$Ir[dF(CF_3)(ppy)]_2(dtbbpy)PF_6$	MeCN	2	1:1
7	$Ru(bpz)_3(PF_6)_2$	MeCN	-	-
8	$Acr\text{-}Mes^+ ClO_4^-$	MeCN	trace	-
9	4CzPN	MeCN	46	92:8

10	DCA	MeCN	32	86:14
11[b]	4CzIPN	MeCN	81 (73)	98:2
12[b,c]	4CzIPN	MeCN	32	88:12
13[b,d]	4CzIPN	MeCN	trace	-
14[b,e]	4CzIPN	MeCN	0	-

[a]reaction conditions: **1a** (0.1 mmol), Phth-SCF_3 (1.5 equiv), PC (2 mol%), K_2CO_3 (0.1 equiv), anhydrous MeCN (2 mL), 45 W blue LEDs, 12 h. [b]**1a** (0.2 mmol), K_2CO_3 (0.2 equiv), Phth-SCF_3 (1.3 equiv), anhydrous MeCN (4 mL). [c]No K_2CO_3. [d]No photocatalyst. [e]Under dark condition. The number in parentheses is the isolated yield.

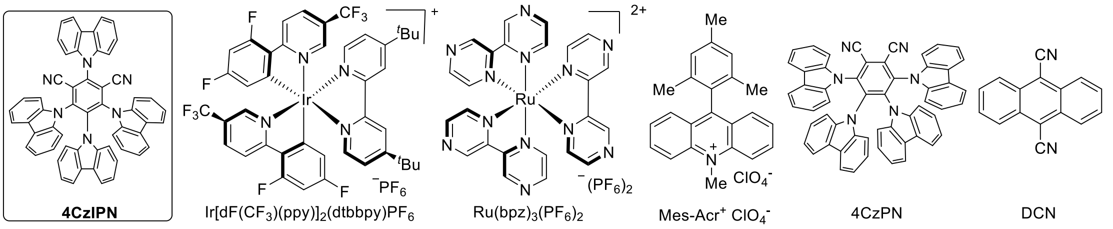

Supplementary Table 2. Screen of base

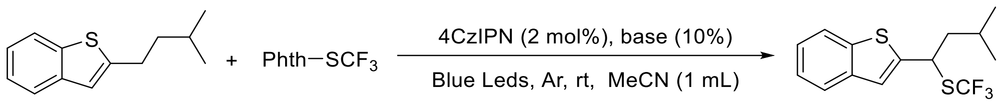

Entry	Base	Yield (%)	Ratio (B/T)
1	K_2CO_3	61	30/1
2	K_2HPO_4	40	14/1
3	$KHCO_3$	50	19/1
4	KOAc	40	16/1
5	KOH	52	17/1
6	Cs_2CO_3	46	22/1
7	K_3PO_4	53	21/1
8	DBU	7	4/1
9	DBN	trace	-
10	Et_3N	20	10/1

Reaction conditions: **1a** (0.1 mmol), Phth-SCF_3 (1.5 equiv), 4CzIPN (2 mol%), base (0.1 equiv), anhydrous MeCN (2 mL), 45 W blue LEDs, 12 h.

General procedure for late-stage trifluoromethylthiolation of benzylic C-H bonds

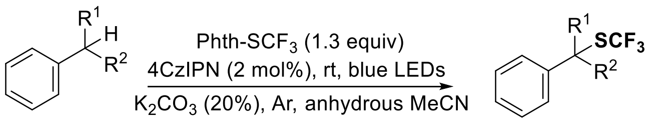

Benzylic C-H substrate **1** (0.2 mmol) $PhthSCF_3$ (64.3 mg, 0.26 mmol), 4CzIPN (3.2 mg, 0.004 mmol), K_2CO_3 (5.52 mg, 0.04 mmol), were placed in a transparent Schlenk tube equipped with a stirring bar. The solvents anhydrous MeCN (4.0 mL) were added under Ar atmosphere. If the benzylic C-H substrate **1** is liquid, anhydrous MeCN and **1** were added in turn. The reaction mixture was stirred under the irradiation of two 45 W blue LEDs (distance app. 4.0 cm from the bulb) at room temperature for 12 – 24 h. When the reaction finished, the mixture was quenched with water and extracted with ethyl acetate (3 x 10 mL). The organic layers were combined and concentrated under vacuo. The product was purified by flash column chromatography on silica gel (petroleum ether: ethyl acetate).

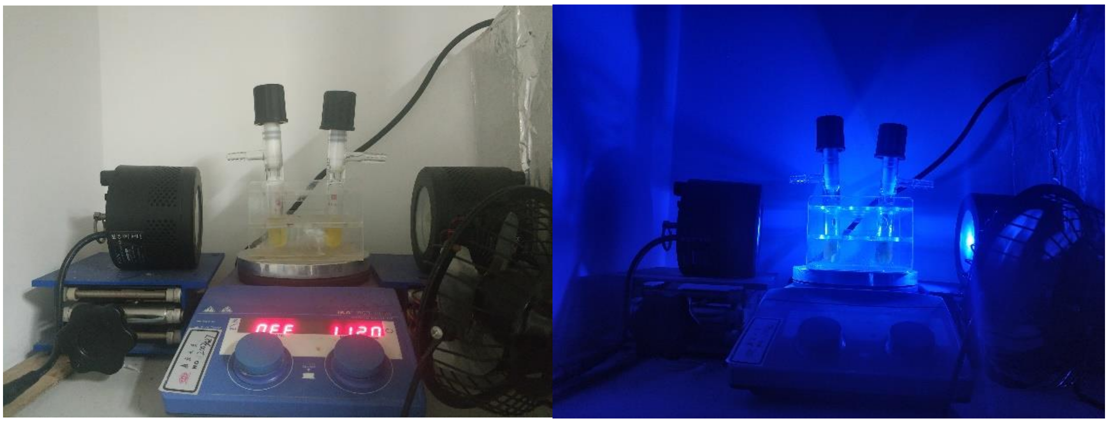

Supplementary Figure 1. Reaction set-up

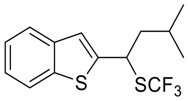

2-(3-methyl-1-((trifluoromethyl)thio)butyl)benzo[b]thiophene (**3a**). According to the general procedure in 0.2 mmol scale using 1.3 equiv $PhthSCF_3$ reagent with reaction time of 12 h; 44.4 mg, 73%, purified by flash chromatography (hexane), colorless oil; Rf = 0.8 (petroleum ether). ^{1}H NMR (400 MHz, $CDCl_3$) δ 7.81 – 7.76 (m, 1H), 7.70 (dd, *J* = 6.9, 1.9 Hz, 1H), 7.32 (pd, *J* = 7.1, 1.4 Hz, 2H), 7.22 (s, 1H), 4.74 (dd, *J* = 9.5, 6.5 Hz, 1H), 1.97 (ddd, *J* = 14.9, 9.4, 5.8 Hz, 1H), 1.87 (ddd, *J* = 14.1, 8.2, 6.5 Hz, 1H), 1.68 (ddq, *J* = 12.8, 8.3, 6.6 Hz, 1H), 0.94 (dd, *J* = 6.6, 5.6 Hz, 6H); ^{19}F NMR (376 MHz, $CDCl_3$) δ -39.98; ^{13}C NMR (101 MHz, $CDCl_3$) δ 145.5, 139.6, 139.1, 130.3 (q, *J* = 307.6 Hz), 124.6, 124.5, 123.6, 122.5, 122.4, 46.0, 44.0, 25.8, 22.6, 21.5. IR (ATR): ν = 3031, 2973, 2933, 1497,1455,1390, 1371, 1094, 1031, 788, 755, 741, 701, 501, 483 cm^{-1}. HRMS (EI) calcd for $C_{14}H_{15}F_3S_2$ (M^+): 304.0567, found 304.0569.

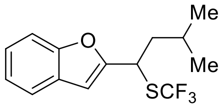

2-(3-methyl-1-((trifluoromethyl)thio)butyl)benzofuran (**3b**). According to the general procedure in 0.2 mmol scale using 1.3 equiv $PhthSCF_3$ reagent with reaction time of 12 h; 44.2 mg, 76%, purified by flash chromatography (hexane), colorless oil; Rf = 0.8 (hexane). ^{1}H NMR (400 MHz, $CDCl_3$) δ 7.52 (dd, *J* = 7.4, 1.3 Hz, 1H), 7.47 (dd, *J* = 8.2, 0.9 Hz, 1H), 7.28 (td, *J* = 8.3, 7.8, 1.5 Hz, 1H), 7.22

(td, J = 7.5, 1.1 Hz, 1H), 6.63 (s, 1H), 4.54 (dd, J = 9.3, 6.8 Hz, 1H), 2.08 (ddd, J = 15.0, 9.2, 6.2 Hz, 1H), 1.85 (dt, J = 14.0, 7.4 Hz, 1H), 1.65 (m, 1H), 0.95 (dd, J = 6.6, 5.4 Hz, 6H); ^{19}F NMR (376 MHz, $CDCl_3$) δ -40.11; ^{13}C NMR (101 MHz, $CDCl_3$) δ 155.4, 154.9, 130.4 (q, J = 307.4 Hz), 128.0, 124.5, 123.0, 121.0, 111.3, 104.5, 42.4, 41.1 (d, J = 1.9 Hz), 25.8, 22.4, 21.7. IR (ATR): ν = 2960, 2934, 2872, 1585, 1469, 1454, 1388, 1370, 1294, 1253, 1148, 1103, 1010, 944, 930, 893, 882, 809, 747, 694 cm^{-1}. HRMS (EI) calcd for $C_{14}H_{15}F_3OS$ (M^+): 288.0796, found 288.0792.

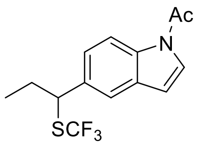

1-(5-(1-((trifluoromethyl)thio)propyl)-1H-indol-1-yl)ethan-1-one (**3c**). According to the general procedure in 0.2 mmol scale using 1.3 equiv $PhthSCF_3$ reagent with reaction time of 12 h; 34.2 mg, 57%, colorless oil; purified by flash chromatography (petroleum ether/ethylacetate 20:1), Rf = 0.3 (petroleum ether/ethylacetate 10:1). ^{1}H NMR (400 MHz, $CDCl_3$) δ 8.40 (d, J = 8.6 Hz, 1H), 7.49 (d, J = 1.8 Hz, 1H), 7.43 (d, J = 3.8 Hz, 1H), 7.29 (dd, J = 8.6, 1.9 Hz, 1H), 6.62 (dd, J = 3.8, 0.8 Hz, 1H), 4.31 (dd, J = 9.1, 6.2 Hz, 1H), 2.63 (s, 3H), 2.06 (ttd, J = 21.5, 14.3, 7.5 Hz, 2H), 0.91 (t, J = 7.3 Hz, 3H); ^{19}F NMR (376 MHz, $CDCl_3$) δ -39.74; ^{13}C NMR (101 MHz, $CDCl_3$) δ 168.5, 135.8, 135.0, 130.7, 130.6 (q, J = 308.4 Hz), 125.9, 124.6, 119.8, 116.8, 109.1, 51.5, 30.1, 23.9, 12.0. IR (ATR): ν = 2972, 2936, 2877, 1706, 1541, 1468, 1442, 1381, 1324, 1291, 1213, 1103, 933, 817, 719, 632, 434 cm^{-1}. HRMS m/z (ESI) calcd for $C_{14}H_{14}F_3NNaOS^+$ $(M + Na)^+$ 324.0640; found: 324.0635.

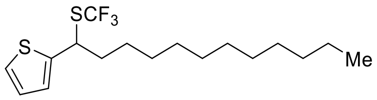

2-(1-((trifluoromethyl)thio)dodecyl)thiophene (**3d**). According to the general procedure in 0.2 mmol scale using 1.3 equiv $PhthSCF_3$ reagent with reaction time of 12 h; 54.6 mg, 77%, purified by flash chromatography (hexane), colorless oil; Rf = 0.7 (petroleum ether). ^{1}H NMR (500 MHz, $CDCl_3$) δ 7.30 (dd, J = 5.0, 3.0 Hz, 1H), 7.15 (dd, J = 2.9, 1.1 Hz, 1H), 7.05 (dd, J = 5.0, 1.4 Hz, 1H), 4.42 (dd, J = 8.5, 6.6 Hz, 1H), 2.03 – 1.89 (m, 2H), 1.39 – 1.17 (m, 18H), 0.88 (t, J = 6.9 Hz, 3H); ^{19}F NMR (471 MHz, $CDCl_3$) δ -39.89; ^{13}C NMR (126 MHz, $CDCl_3$) δ 141.2, 130.6 (q, J = 307.4 Hz), 126.4, 126.3, 122.3, 44.8 (d, J = 1.7 Hz), 36.3, 31.9, 29.6, 29.6, 29.5, 29.3, 29.0, 27.2, 22.7, 14.1. IR (ATR): ν = 2924, 2854, 1466, 1145, 1106, 834, 781, 756, 740, 685, 673, 636 cm^{-1}. HRMS (EI) calcd for $C_{17}H_{27}F_3S_2$ (M^+): 352.1506, found 352.1515.

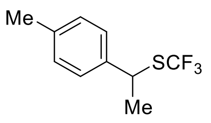

(1-(p-tolyl)ethyl)(trifluoromethyl)sulfane (**3e**). According to the general procedure in 0.2 mmol scale

using 1.3 equiv $PhthSCF_3$ reagent with reaction time of 12 h; 22.5 mg, 51%, purified by flash chromatography (hexane), colorless oil; Rf = 0.7 (petroleum ether). ^{1}H NMR (500 MHz, $CDCl_3$) δ 7.24 (d, *J* = 8.2 Hz, 2H), 7.15 (d, *J* = 7.9 Hz, 2H), 4.50 (q, *J* = 7.1 Hz, 1H), 2.34 (s, 3H), 1.71 (d, *J* = 7.1 Hz, 3H); ^{19}F NMR (471 MHz, $CDCl_3$) δ -40.16; ^{13}C NMR (126 MHz, $CDCl_3$) δ 138.2, 137.8, 130.5 (q, *J* = 307.9 Hz), 129.5, 126.9, 44.3 (d, *J* = 1.8 Hz), 23.1, 21.1. IR (ATR): ν = 2925, 2854, 1515, 1455, 1379, 1145, 1108, 1062, 1048, 1020, 908, 817, 756, 734, 538, 520 cm^{-1}. HRMS (EI) calcd for $C_{10}H_{11}F_3S$ (M^+): 220.0534, found 220.0530.

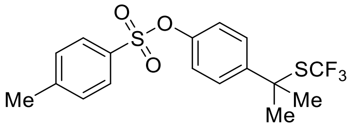

4-(2-((trifluoromethyl)thio)propan-2-yl)phenyl 4-methylbenzenesulfonate (**3f**). According to the general procedure in 0.2 mmol scale using 1.5 equiv $PhthSCF_3$ reagent with reaction time of 12 h; 47.0 mg, 60%, purified by flash chromatography (petroleum/ethylacetate = 20/1), colorless oil; Rf = 0.2 (petroleum ether/ethylacetate = 20/1). ^{1}H NMR (400 MHz, $CDCl_3$) δ 7.68 (d, *J* = 8.3 Hz, 2H), 7.46 (d, *J* = 8.9 Hz, 2H), 7.30 (d, *J* = 8.0 Hz, 2H), 6.96 (d, *J* = 8.9 Hz, 2H), 2.45 (s, 3H), 1.84 (s, 6H); ^{19}F NMR (376 MHz, $CDCl_3$) δ -37.08; ^{13}C NMR (101 MHz, $CDCl_3$) δ 148.7, 145.4, 143.4, 132.2, 130.2 (q, *J* = 310.1Hz), 129.7, 128.5, 127.7, 122.1, 52.0, 30.7 (d, *J* = 1.0 Hz), 21.7. IR (ATR): ν = 2978, 2929, 1598, 1503, 1373, 1180, 1157, 1104, 1084, 1017, 863, 844, 743, 492 cm^{-1}. HRMS m/z (ESI) calcd for $C_{17}H_{17}F_3NaO_3S_2^+$ $(M + Na)^+$ 413.0463; found: 413.0461.

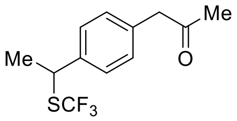

1-(4-(1-((trifluoromethyl)thio)ethyl)phenyl)propan-2-one (**3g**). According to the general procedure in 0.2 mmol scale using 1.3 equiv $PhthSCF_3$ reagent with reaction time of 12 h; 34.6 mg, 66%, purified by flash chromatography (hexane), colorless oil; Rf = 0.5 (petroleum ether/ethylacetate 50:1). ^{1}H NMR (400 MHz, $CDCl_3$) δ 7.32 (d, *J* = 8.2 Hz, 2H), 7.18 (d, *J* = 8.1 Hz, 2H), 4.52 (q, *J* = 7.1 Hz, 1H), 3.70 (s, 2H), 2.16 (s, 3H), 1.72 (dd, *J* = 7.1, 0.9 Hz, 3H); ^{19}F NMR (376 MHz, $CDCl_3$) δ -40.15; ^{13}C NMR (101 MHz, $CDCl_3$) δ 206.0, 140.1, 134.0, 130.4 (q, *J* = 308.4 Hz), 129.9, 127.4, 50.5, 44.2 (d, *J* = 1.9 Hz), 29.4, 23.0. IR (ATR): ν = 2979, 2932, 1714, 1514, 1450, 1423, 1358, 1323, 1229, 1110, 1062, 1049, 1021, 839, 756, 541 cm^{-1}. HRMS m/z (ESI) calcd for $C_{12}H_{13}F_3NaOS^+$ $(M + Na)^+$ 285.0531; found: 285.0525.

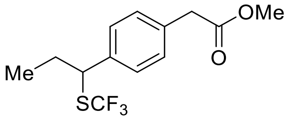

methyl 2-(4-(1-((trifluoromethyl)thio)propyl)phenyl)acetate (**3h**). According to the general procedure in 0.1 mmol scale using 1.3 equiv $PhthSCF_3$ reagent with reaction time of 12 h; 18.9 mg,

64%, purified by flash chromatography (petroleum ether/ethylacetate 50:1), colorless oil; Rf = 0.4 (petroleum ether/ethylacetate 20:1). ^{1}H NMR (400 MHz, $CDCl_3$) δ 7.25 (s, 4H), 4.19 (dd, *J* = 8.8, 6.4 Hz, 1H), 3.70 (s, 3H), 3.62 (s, 2H), 2.10 – 1.89 (m, 2H), 0.92 (t, *J* = 7.3 Hz, 3H); ^{19}F NMR (376 MHz, $CDCl_3$) δ -39.79; ^{13}C NMR (101 MHz, $CDCl_3$) δ 171.9, 139.3, 133.6, 130.5 (q, *J* = 308.05 Hz), 129.6, 127.7, 52.1, 51.0 (d, *J* = 1.7 Hz), 40.8, 29.8, 11.9. IR (ATR): ν = 2972, 2879, 1738, 1515, 1436, 1424, 1382, 1337, 1259, 1222, 1192, 1108, 1020, 810, 756 cm^{-1}. HRMS m/z (ESI) calcd for $C_{13}H_{15}F_3NaO_2S^+$ $(M + Na)^+$ 315.0637; found: 315.0636.

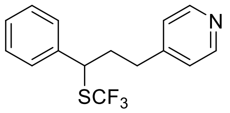

4-(3-phenyl-3-((trifluoromethyl)thio)propyl)pyridine (**3i**). According to the general procedure in 0.2 mmol scale using 1.3 equiv $PhthSCF_3$ reagent with reaction time of 12 h; 36.9 mg, 62%, purified by flash chromatography (petroleum ether/ethylacetate = 10/1), colorless oil; Rf = 0.5 (petroleum ether/ethylacetate = 5/1). ^{1}H NMR (400 MHz, $CDCl_3$) δ^{1}H NMR (400 MHz, $CDCl_3$) δ 8.53 – 8.49 (m, 2H), 7.40 – 7.33 (m, 2H), 7.29 (dd, *J* = 8.1, 1.4 Hz, 2H), 7.23 – 7.16 (m, 1H), 7.10 – 7.01 (m, 2H), 4.25 (dd, *J* = 8.9, 6.4 Hz, 1H), 2.65 – 2.58 (m, 2H), 2.34 – 2.20 (m, 1H), 1.98 (tt, *J* = 9.1, 6.9 Hz, 1H); ^{19}F NMR (376 MHz, $CDCl_3$) δ -39.70; ^{13}C NMR (101 MHz, $CDCl_3$) δ 149.9, 139.6, 130.3 (q, *J* = 308.4 Hz), 129.0, 128.3, 127.4, 126.0, 123.7, 48.7 (d, *J* = 1.7 Hz), 36.8, 32.6. Owing to the extremely similar polarity of **1i** and **3i**, the pure product **3i** could not be obtained. (ATR): ν = 3064, 3028, 2928, 2857, 1734, 1683, 1601, 1559, 1495, 1454, 1415, 1313, 1220, 1108, 1030, 993, 822, 756, 717, 567 cm^{-1}. HRMS m/z (ESI) calcd for $C_{15}H_{15}F_3NS^+$ $(M + H)^+$ 298.0872; found: 298.0879.

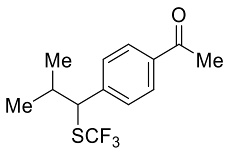

1-(4-(2-methyl-1-((trifluoromethyl)thio)propyl)phenyl)ethan-1-one (**3j**). According to the general procedure in 0.2 mmol scale using 1.3 equiv $PhthSCF_3$ reagent with reaction time of 12 h; 41.2 mg, 74%, purified by flash chromatography (hexane), colorless oil; Rf = 0.8 (petroleum ether). ^{1}H NMR ^{1}H NMR (400 MHz, $CDCl_3$) δ 7.93 (d, *J* = 8.4 Hz, 2H), 7.39 (d, *J* = 8.4 Hz, 2H), 4.13 (d, *J* = 7.7 Hz, 1H), 2.61 (s, 3H), 2.15 (dq, *J* = 13.7, 6.8 Hz, 1H), 1.08 (d, *J* = 6.7 Hz, 3H), 0.90 (d, *J* = 6.7 Hz, 3H); ^{19}F NMR (471 MHz, $CDCl_3$) δ -39.85; ^{13}C NMR (101 MHz, $CDCl_3$) δ 197.5, 146.1, 130.6 (q, *J* = 308.1 Hz), 128.5, 128.3, 33.9, 26.6, 20.5, 20.4. IR (ATR): ν = 2966, 2936, 1686, 1607, 1390, 1360, 1268, 1116, 1106, 755 cm^{-1}. HRMS m/z (ESI) calcd for $C_{13}H_{16}F_3OS^+$ $(M + H)^+$ 277.0868; found: 277.0880.

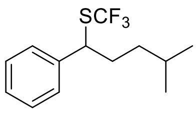

(4-methyl-1-phenylpentyl)(trifluoromethyl)sulfane (**3k**). According to the general procedure in 0.1 mmol scale using 1.3 equiv $PhthSCF_3$ reagent with reaction time of 12 h; 13.4 mg, 51%, purified by flash chromatography (hexane), colorless oil; Rf = 0.8 (petroleum ether); ^{1}H NMR (400 MHz, $CDCl_3$) δ 7.37 – 7.24 (m, 5H), 4.24 (dd, *J* = 8.8, 6.6 Hz, 1H), 2.03 – 1.88 (m, 2H), 1.23 – 1.18 (m, 1H), 1.14 – 1.03 (m, 1H), 0.85 (d, *J* = 2.0 Hz, 3H), 0.84 (d, *J* = 2.0 Hz, 3H), 0.63 (dd, *J* = 12.2, 6.6 Hz, 1H); ^{19}F NMR (376 MHz, $CDCl_3$) δ -39.81; ^{13}C NMR (101 MHz, $CDCl_3$) δ 140.8, 130.6 (q, *J* = 309.4 Hz), 128.7, 127.8, 127.4, 50.0 (d, *J* = 1.4 Hz), 36.3, 34.5, 27.7, 22.5, 22.3. IR (ATR): ν = 2958, 2930, 2872, 1494, 1469, 1454, 1145, 1103, 756, 697 cm^{-1}. HRMS (EI) calcd for $C_{13}H_{17}F_3S$ (M^+): 262.1003, found 262.1007.

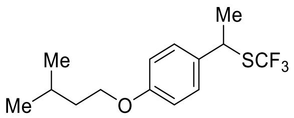

(1-(4-(isopentyloxy)phenyl)ethyl)(trifluoromethyl)sulfane (**3l**). According to the general procedure in 0.2 mmol scale using 1.3 equiv $PhthSCF_3$ reagent with reaction time of 12 h; 42.0 mg, 72%, purified by flash chromatography (hexane), colorless oil; Rf = 0.7 (petroleum ether). ^{1}H NMR (400 MHz, $CDCl_3$) δ 7.25 (d, *J* = 8.7 Hz, 2H), 6.86 (d, *J* = 8.8 Hz, 2H), 4.51 (q, *J* = 7.0 Hz, 1H), 3.97 (t, *J* = 6.7 Hz, 2H), 1.83 (m, 1H), 1.71 (d, *J* = 7.0 Hz, 3H), 1.67 (q, *J* = 6.8 Hz, 2H), 0.96 (d, *J* = 6.6 Hz, 6H); ^{19}F NMR (376 MHz, $CDCl_3$) δ -40.15; ^{13}C NMR (101 MHz, $CDCl_3$) δ 158.8, 132.8, 130.5 (q, *J* = 308.7 Hz), 128.2, 114.7, 66.4, 44.2 (d, *J* = 1.9 Hz), 38.0, 25.1, 23.1, 22.6. IR (ATR): ν = 2958, 2936, 2873, 2344, 1611, 1513, 1252, 1113, 810, 716 cm^{-1}. HRMS (EI) calcd for $C_{14}H_{19}F_3OS$ (M^+): 292.1109, found 292.1115.

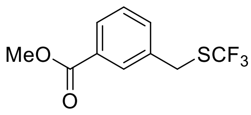

methyl 3-(((trifluoromethyl)thio)methyl)benzoate (**3m**). According to the general procedure in 0.2 mmol scale using 1.3 equiv $PhthSCF_3$ reagent with reaction time of 12 h; 26.5 mg, 53%, purified by flash chromatography (petroleum ether/ethylacetate = 100/1), colorless oil; Rf = 0.4 (petroleum ether/ethylacetate = 50/1). ^{1}H NMR (400 MHz, $CDCl_3$) δ 8.03 (s, 1H), 7.98 (d, *J* = 7.8 Hz, 1H), 7.55 (d, *J* = 7.7 Hz, 1H), 7.43 (t, *J* = 7.7 Hz, 1H), 4.16 (s, 2H), 3.93 (s, 3H); ^{19}F NMR (376 MHz, $CDCl_3$) δ -41.52; ^{13}C NMR (101 MHz, $CDCl_3$) δ 166.5, 135.7, 133.3, 130.9, 130.5 (q, *J* = 308.1 Hz), 130.0, 129.2, 129.0, 52.3, 33.9 (d, *J* = 2.5 Hz). IR (ATR): ν = 2954, 1720, 1448, 1435, 1286, 1202, 1099, 991, 756, 726, 707 cm^{-1}. HRMS (EI) calcd for $C_{10}H_9F_3O_2S$ (M^+): 250.0275, found 250.0273.

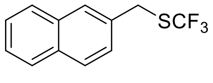

(naphthalen-2-ylmethyl)(trifluoromethyl)sulfane[1] (**3n**). According to the general procedure in 0.2 mmol scale using 1.3 equiv $PhthSCF_3$ reagent with reaction time of 18 h; 28.0 mg, 58%, purified

by flash chromatography (hexane), colorless oil; Rf = 0.8 (PE). ^{1}H NMR (400 MHz, $CDCl_3$) δ 7.88 – 7.77 (m, 4H), 7.53 – 7.43 (m, 3H), 4.29 (s, 2H); ^{19}F NMR (376 MHz, $CDCl_3$) δ -41.52; IR (ATR): ν = 3059, 2929, 1601, 1510, 1108, 817, 752, 474 cm^{-1}. MS (EI) calcd for $C_{12}H_9F_3S$ (M^+): 242.0, found 242.0.

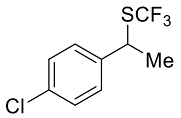

(1-(4-chlorophenyl)ethyl)(trifluoromethyl)sulfane (**3o**). According to the general procedure in 0.2 mmol scale using 1.3 equiv PhthSCF$_3$ reagent with reaction time of 12 h; 34.3 mg, 71%, purified by flash chromatography (hexane), colorless oil; Rf = 0.4 (hexane); ^{1}H NMR (500 MHz, $CDCl_3$) δ 7.30 (q, *J* = 8.7 Hz, 4H), 4.49 (q, *J* = 7.1 Hz, 1H), 1.70 (d, *J* = 7.0 Hz, 3H); ^{19}F NMR (471 MHz, $CDCl_3$) δ -40.14; ^{13}C NMR (126 MHz, $CDCl_3$) δ 140.0, 133.8, 130.3 (q, *J* =308.3 Hz), 129.0, 128.4, 43.8 (d, *J* = 1.8 Hz), 22.9. IR (ATR): ν = 2978, 2931, 1493, 1147, 1109, 1094, 1015, 829, 756, 531 cm^{-1}. HRMS (EI) calcd for $C_9H_8ClF_3S$ (M^+): 239.9987, found 239.9985.

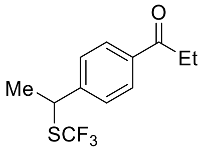

1-(4-(1-((trifluoromethyl)thio)ethyl)phenyl)propan-1-one (**3p**). According to the general procedure in 0.2 mmol scale using 1.3 equiv PhthSCF$_3$ reagent with reaction time of 12 h; 32.2 mg, 61%, purified by flash chromatography (petroleum ether/ethylacetate = 100/1), colorless oil; Rf = 0.8 (petroleum ether/ethylacetate = 20/1); ^{1}H NMR (400 MHz, $CDCl_3$) δ 7.95 (d, *J* = 8.2 Hz, 2H), 7.45 (d, *J* = 8.2 Hz, 2H), 4.55 (q, *J* = 7.1 Hz, 1H), 3.00 (q, *J* = 7.2 Hz, 2H), 1.72 (d, *J* = 7.1 Hz, 3H), 1.23 (t, *J* = 7.2 Hz, 3H); ^{19}F NMR (376 MHz, $CDCl_3$) δ -40.13; ^{13}C NMR (101 MHz, $CDCl_3$) δ 200.1, 146.5, 136.5, 130.2 (q, *J* = 308.7 Hz), 128.6, 127.2, 44.0, 31.8, 22.7, 8.2. IR (ATR): ν = 2980, 2940, 1686, 1608, 1415, 1222, 1106, 952, 800, 756, 573 cm^{-1}. HRMS m/z (ESI) calcd for $C_{12}H_{14}F_3OS^+$ $(M + H)^+$ 263.0712; found: 263.0717.

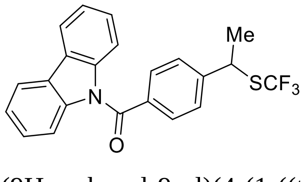

(9H-carbazol-9-yl)(4-(1-((trifluoromethyl)thio)ethyl)phenyl)methanone (**3q**). According to the general procedure in 0.2 mmol scale using 1.3 equiv PhthSCF$_3$ reagent with reaction time of 12 h; 60.3 mg, 75%, purified by flash chromatography (petroleum ether/ethylacetate = 50/1), colorless oil; Rf = 0.4 (petroleum ether/ethylacetate = 20/1); ^{1}H NMR (400 MHz, $CDCl_3$) δ 8.03 – 7.98 (m, 2H), 7.69 (d, *J* = 8.3 Hz, 2H), 7.55 – 7.45 (m, 4H), 7.38 – 7.28 (m, 4H), 4.61 (q, *J* = 7.1 Hz, 1H), 1.76 (d, *J* = 7.1 Hz, 3H); ^{19}F NMR (376 MHz, $CDCl_3$) δ -39.94; ^{13}C NMR (101 MHz, $CDCl_3$) δ

169.0, 146.3, 139.0, 135.4, 130.2 (q, J = 308.7 Hz), 129.5, 127.7, 126.8, 126.1, 123.5, 119.9, 115.8, 44.1 (d, J = 1.8 Hz), 22.5. IR (ATR): ν = 3062, 2976, 2930, 1676, 1443, 1324, 1297, 1104, 838, 751, 722, 617 cm^{-1}. HRMS m/z (ESI) calcd for $C_{22}H_{16}F_3NNaOS^+$ $(M + Na)^+$ 422.0797; found: 422.0799.

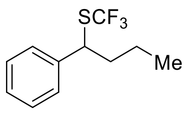

(1-phenylbutyl)(trifluoromethyl)sulfane (**3r**). According to the general procedure in 0.2 mmol scale using 1.3 equiv $PhthSCF_3$ reagent with reaction time of 12 h; 35.8 mg, 77%, purified by flash chromatography (hexane), colorless oil; Rf = 1.0 (hexane). ^1H NMR (400 MHz, $CDCl_3$) δ 7.38 – 7.27 (m, 5H), 4.29 (dd, J = 8.8, 6.7 Hz, 1H), 1.95 (dt, J = 8.8, 6.3 Hz, 2H), 1.34 – 1.26 (m, 2H), 0.90 (t, J = 7.4 Hz, 3H); ^{19}F NMR (376 MHz, $CDCl_3$) δ -39.83; ^{13}C NMR (101 MHz, $CDCl_3$) δ 140.8, 130.6 (q, J = 308.4 Hz), 128.7, 127.8, 127.4, 49.4 (d, J = 1.8 Hz), 38.6, 20.5, 13.5. IR (ATR): ν = 2963, 2935, 2876, 1494, 1455, 1110, 1030, 756, 748, 718, 696 cm^{-1}. HRMS (EI) calcd for $C_{11}H_{13}F_3S$ (M^+): 234.0690, found 234.0700.

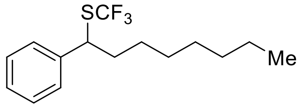

(1-phenyloctyl)(trifluoromethyl)sulfane (**3s**). According to the general procedure in 0.2 mmol scale using 1.3 equiv $PhthSCF_3$ reagent with reaction time of 12 h; 41.9 mg, 72%, purified by flash chromatography (hexane), colorless oil; Rf = 0.9 (hexane); ^1H NMR (500 MHz, $CDCl_3$) δ 7.39 – 7.25 (m, 5H), 4.27 (dd, J = 8.9, 6.5 Hz, 1H), 2.02 – 1.88 (m, 2H), 1.36 – 1.17 (m, 10H), 0.86 (t, J = 7.0 Hz, 3H); ^{19}F NMR (471 MHz, $CDCl_3$) δ -39.80; ^{13}C NMR (126 MHz, $CDCl_3$) δ 140.8, 130.6 (q, J = 307.9 Hz), 128.7, 127.8, 127.4, 49.7 (d, J = 1.5 Hz), 36.6, 31.7, 29.0, 29.0, 27.2, 22.6, 14.1. IR (ATR): ν = 2956, 2932, 2859, 2358, 1455, 1146, 1114, 736, 687 cm^{-1}. HRMS (EI) calcd for $C_{15}H_{21}F_3S$ (M^+): 290.1316, found 290.1322.

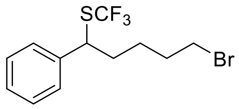

(5-bromo-1-phenylpentyl)(trifluoromethyl)sulfane (**3t**). According to the general procedure in 0.2 mmol scale using 1.3 equiv $PhthSCF_3$ reagent with reaction time of 11 h; 58.1 mg, 89%, purified by flash chromatography (hexane), colorless oil; Rf = 0.5 (hexane); ^1H NMR (400 MHz, $CDCl_3$) δ 7.38 – 7.32 (m, 2H), 7.32 – 7.25 (m, 3H), 4.27 (dd, J = 8.8, 6.6 Hz, 1H), 3.34 (tt, J = 6.8, 3.6 Hz, 2H), 2.06 – 1.93 (m, 2H), 1.84 (tdd, J = 9.4, 7.7, 4.7 Hz, 2H), 1.56 – 1.45 (m, 1H), 1.37 (dddd, J = 13.0, 9.2, 6.6, 3.1 Hz, 1H); ^{19}F NMR (376 MHz, $CDCl_3$) δ -39.77; ^{13}C NMR (101 MHz, $CDCl_3$) δ 140.2, 130.4 (q, J = 308.4 Hz), 128.8, 128.0, 127.3, 49.4, 35.7, 33.0, 32.1, 25.9. IR (ATR): ν = 3031, 2942, 2863, 1494, 1454, 1101, 743, 696 cm^{-1}. HRMS (EI) calcd for $C_{12}H_{14}BrF_3S$ (M^+): 325.9952, found 325.9948.

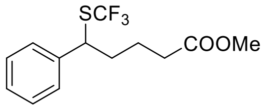

methyl 5-phenyl-5-((trifluoromethyl)thio)pentanoate (**3u**). According to the general procedure in 0.2 mmol scale using 1.3 equiv $PhthSCF_3$ reagent with reaction time of 12 h; 42.6 mg, 73%, purified by flash chromatography (petroleum ether/ethylacetate = 100/1), colorless oil; Rf = 0.8 (petroleum ether/ethylacetate = 20/1); ^1H NMR (400 MHz, $CDCl_3$) δ 7.40 – 7.25 (m, 5H), 4.28 (dd, J = 8.7, 6.8 Hz, 1H), 3.64 (s, 3H), 2.30 (t, J = 7.1 Hz, 2H), 2.01 (ddd, J = 11.7, 9.4, 6.0 Hz, 2H), 1.62 (dddq, J = 34.2, 13.8, 9.4, 6.8, 6.4 Hz, 2H); ^{19}F NMR (376 MHz, $CDCl_3$) δ -39.83; ^{13}C NMR (101 MHz, $CDCl_3$) δ 173.3, 140.1, 130.4 (q, J = 308.4 Hz), 128.8, 128.0, 127.4, 51.6, 49.3, 35.8, 33.3, 22.6. IR (ATR): ν = 3031, 2954, 1735, 1455, 1437, 1102, 756, 697 cm^{-1}. HRMS m/z (ESI) calcd for $C_{13}H_{15}F_3NaO_2S^+$ $(M + Na)^+$ 315.0637; found: 315.0647.

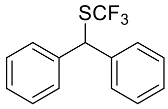

benzhydryl(trifluoromethyl)sulfane (**3v**). According to the general procedure in 0.2 mmol scale using 1.5 equiv $PhthSCF_3$ reagent with reaction time of 12 h; 44.5 mg, 83%, purified by flash chromatography (hexane), colorless oil; Rf = 0.6 (hexane); ^1H NMR (400 MHz, $CDCl_3$) δ 7.42 – 7.38 (m, 4H), 7.37 – 7.32 (m, 4H), 7.30 – 7.25 (m, 2H), 5.69 (s, 1H); ^{19}F NMR (376 MHz, $CDCl_3$) δ -40.81; ^{13}C NMR (101 MHz, $CDCl_3$) δ 139.2, 130.0 (q, J = 309.7 Hz), 128.8, 128.2, 127.9, 53.5. IR (ATR): ν = 3064, 3030, 2928, 1494, 1451, 1106, 747, 695, 590 cm^{-1}. HRMS (EI) calcd for $C_{14}H_{11}F_3S$ (M^+): 268.0534, found 268.0542.

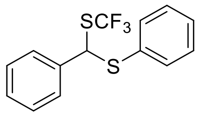

phenyl(phenyl((trifluoromethyl)thio)methyl)sulfane (**3w**). According to the general procedure in 0.1 mmol scale using 1.5 equiv $PhthSCF_3$ reagent with reaction time of 12 h; 15.6 mg, 52%, purified by flash chromatography (hexane), colorless oil; Rf = 0.6 (hexane); ^1H NMR (500 MHz, $CDCl_3$) δ 7.46 (dd, J = 6.3, 2.7 Hz, 2H), 7.41 (d, J = 6.7 Hz, 2H), 7.34 (t, J = 6.9 Hz, 6H), 5.56 (s, 1H); ^{19}F NMR (471 MHz, $CDCl_3$) δ -40.96; ^{13}C NMR (126 MHz, $CDCl_3$) δ 138.3, 133.4, 132.5, 129.4 (q, J = 310.4 Hz), 129.2, 128.9, 128.8, 128.8, 127.5, 55.7 (d, J = 1.5 Hz). IR (ATR): ν = 3062, 3031, 2927, 1583, 1480, 1453, 1440, 1101, 1001, 736, 689, 470 cm^{-1}. HRMS (EI) calcd for $C_{14}H_{11}F_3S_2$ (M^+): 300.0254, found 300.0260.

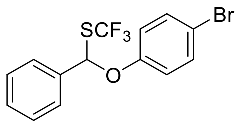

((4-bromophenoxy)(phenyl)methyl)(trifluoromethyl)sulfane (**3x**). According to the general procedure in 0.1 mmol scale using 1.5 equiv PhthSCF$_3$ reagent with reaction time of 12 h; 17.7 mg, 49%, purified by flash chromatography (hexane), colorless oil; Rf = 0.5 (hexane); ^{1}H NMR (400 MHz, CDCl$_3$) δ 7.52 (dd, *J* = 7.6, 1.6 Hz, 2H), 7.45 – 7.38 (m, 3H), 7.38 – 7.33 (m, 2H), 6.81 (d, *J* = 8.9 Hz, 2H), 6.63 (s, 1H); ^{19}F NMR (376 MHz, CDCl$_3$) δ -40.00; ^{13}C NMR (101 MHz, CDCl$_3$) δ 155.9, 135.6, 132.5, 129.7, 129.4 (q, *J* = 310.7 Hz), 129.1, 126.3, 118.8, 115.5, 84.3 (q, *J* = 2.5 Hz). IR (ATR): ν = 3068, 2929, 2350, 1580, 1486, 1220, 1111, 1006, 798, 696 cm^{-1}. HRMS (EI) calcd for $C_{14}H_{10}BrF_3OS$ (M$^+$): 361.9588, found 361.9592.

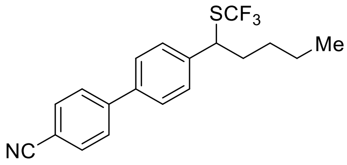

4'-(1-((trifluoromethyl)thio)pentyl)-[1,1'-biphenyl]-4-carbonitrile (**3y**). According to the general procedure in 0.2 mmol scale using 1.3 equiv PhthSCF$_3$ reagent with reaction time of 12 h; 34.7 mg, 50%, purified by flash chromatography (petroleum ether/ethylacetate = 50/1), colorless oil; Rf = 0.6 (petroleum ether/ethylacetate = 20/1); ^{1}H NMR (400 MHz, CDCl$_3$) δ 7.71 (q, *J* = 8.0 Hz, 4H), 7.57 (d, *J* = 7.8 Hz, 2H), 7.42 (d, *J* = 7.8 Hz, 2H), 4.33 (t, *J* = 7.7 Hz, 1H), 1.98 (dq, *J* = 19.0, 6.5, 5.2 Hz, 2H), 1.42 – 1.23 (m, 4H), 0.88 (t, *J* = 6.8 Hz, 3H); ^{19}F NMR (376 MHz, CDCl$_3$) δ -39.72; ^{13}C NMR (101 MHz, CDCl$_3$) δ 144.9, 141.6, 138.6, 132.6, 130.5 (q, *J* = 308.4), 128.2, 127.6, 127.5, 118.9, 111.1, 49.2 (d, *J* = 1.8 Hz), 36.1, 29.4, 22.1, 13.8. IR (ATR): ν = 2959, 2932, 2861, 2227, 1607, 1494, 1100, 1006, 822, 728, 558, 542 cm^{-1}. HRMS m/z (ESI) calcd for $C_{19}H_{18}F_3NNaS^+$ (M + Na)$^+$ 372.1004; found: 372.1016.

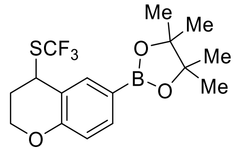

4,4,5,5-tetramethyl-2-(4-((trifluoromethyl)thio)chroman-6-yl)-1,3,2-dioxaborolane (**3z**). According to the general procedure in 0.2 mmol scale using 1.3 equiv PhthSCF$_3$ reagent with reaction time of 12 h; 25.8 mg, 36%, purified by flash chromatography (petroleum ether/ethylacetate = 100/1), colorless oil; Rf = 0.3 (petroleum ether/ethylacetate = 50/1); ^{1}H NMR (400 MHz, CDCl$_3$) δ 7.76 (s, 1H), 7.61 (dd, *J* = 8.2, 1.6 Hz, 1H), 6.80 (d, *J* = 8.3 Hz, 1H), 4.70 (q, *J* = 3.0, 2.5 Hz, 1H), 4.46 – 4.34 (m, 2H), 2.45 (ddt, *J* = 15.2, 11.1, 4.2 Hz, 1H), 2.31 (dd, *J* = 14.8, 2.5 Hz, 1H), 1.37 – 1.30 (m, 12H); ^{19}F NMR (376 MHz, CDCl$_3$) δ -40.02; ^{13}C NMR (126 MHz, CDCl$_3$) δ 157.9, 138.3, 137.6 (q, *J* = 201.6 Hz), 136.4, 130.3 (q, *J* = 309.1 Hz), 117.2, 117.0, 83.8, 62.0, 40.0 (d, *J* = 2.2 Hz), 29.4, 24.9, 24.8. IR (ATR): ν = 2981, 2350, 1610, 1359, 1268, 1129, 1113, 723 cm^{-1}. HRMS (EI) calcd for $C_{16}H_{20}BF_3O_3S$ (M$^+$): 359.1215, found 359.1213.

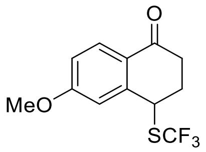

6-methoxy-4-((trifluoromethyl)thio)-3,4-dihydronaphthalen-1(2H)-one (**3aa**). According to the general procedure in 0.2 mmol scale using 1.3 equiv $PhthSCF_3$ reagent with reaction time of 12 h; 26.7 mg, 48%, purified by flash chromatography (petroleum ether/ethylacetate = 20/1), colorless oil; Rf = 0.5 (petroleum ether/ethylacetate = 10/1). ^1H NMR (500 MHz, $CDCl_3$) δ 8.06 – 7.99 (m, 1H), 6.93 (d, J = 7.9 Hz, 2H), 4.80 (t, J = 3.7 Hz, 1H), 3.89 (s, 3H), 3.04 (ddd, J = 17.4, 12.6, 4.8 Hz, 1H), 2.72 – 2.55 (m, 2H), 2.52 – 2.44 (m, 1H); ^{19}F NMR (471 MHz, $CDCl_3$) δ -40.06; ^{13}C NMR (126 MHz, $CDCl_3$) δ 194.8, 164.0, 142.1, 130.3 (q, J = 308.3 Hz), 130.2, 125.9, 115.4, 113.5, 55.7, 44.4 (d, J = 1.8 Hz), 33.8, 29.2. IR (ATR): ν = 2948, 2843, 1680, 1597, 1256, 1100, 1020, 1005, 878, 724, 544 cm^{-1}. HRMS m/z (ESI) calcd for $C_{12}H_{12}F_3O_2S^+$ $(M + H)^+$ 277.0505; found: 277.0500.

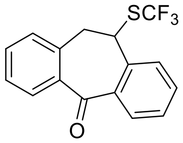

10-((trifluoromethyl)thio)-10,11-dihydro-5H-dibenzo[a,d][7]annulen-5-one (**3bb**). According to the general procedure in 0.2 mmol scale using 1.3 equiv $PhthSCF_3$ reagent with reaction time of 24 h; 29.3 mg, 47%, purified by flash chromatography (petroleum ether/ethylacetate = 50/1), colorless oil; Rf = 0.5 (petroleum ether/ethylacetate = 20/1); ^1H NMR (400 MHz, $CDCl_3$) δ 8.09 (dd, J = 23.4, 7.8 Hz, 2H), 7.52 (td, J = 7.6, 2.3 Hz, 2H), 7.43 (q, J = 7.5 Hz, 3H), 7.29 – 7.24 (m, 1H), 5.00 (d, J = 6.3 Hz, 1H), 3.90 (d, J = 15.7 Hz, 1H), 3.48 (dd, J = 15.7, 6.3 Hz, 1H); ^{19}F NMR (376 MHz, $CDCl_3$) δ -40.23; ^{13}C NMR (101 MHz, $CDCl_3$) δ 192.9, 140.4, 138.4, 137.7, 135.7, 132.9, 132.8, 132.0, 131.2, 130.7, 130.5 (q, J = 130.5 Hz), 129.5, 128.7, 127.8, 48.7 (d, J = 2.1 Hz), 40.2. IR (ATR): ν = 1650, 1599, 1294, 1111, 722, 673, 595 cm^{-1}. HRMS m/z (ESI) calcd for $C_{16}H_{12}F_3OS^+$ $(M + H)^+$ 309.0555; found: 309.0555.

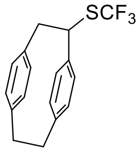

1,4(1,4)-dibenzenacyclohexaphane-2-yl(trifluoromethyl)sulfane (**3cc**). According to the general procedure in 0.2 mmol scale using 5 equiv $PhthSCF_3$ reagent, 0.04 equiv 4CzIPN, and 0.4 equiv K_2CO_3 with reaction time of 24 h; 27.4 mg, 45%, purified by flash chromatography (hexane), white solid; Rf = 0.5 (hexane); ^1H NMR (400 MHz, $CDCl_3$) δ 6.77 (d, J = 8.0 Hz, 1H), 6.55 (h, J = 7.8 Hz, 5H), 6.48 – 6.39 (m, 2H), 4.68 (t, J = 8.4 Hz, 1H), 3.83 (dd, J = 13.7, 9.2 Hz, 1H), 3.23 – 3.13 (m, 2H), 3.06 – 2.94 (m, 2H), 2.73 (dd, J = 13.8, 7.6 Hz, 1H); ^{19}F NMR (376 MHz, $CDCl_3$) δ -40.11; ^{13}C NMR (101 MHz, $CDCl_3$) δ 141.9, 140.6, 137.7, 136.6, 134.6, 134.1, 134.0, 132.4, 132.3, 132.1,

131.8, 130.7, 130.3 (q, J = 308.7 Hz), 48.7 (d, J = 1.0 Hz), 44.5, 35.7, 35.5. IR (ATR): ν = 2925, 2892, 2853, 1498, 1414, 1110, 811, 719, 534 cm^{-1}. HRMS (EI) calcd for $C_{17}H_{15}F_3S$ (M^+): 308.0847, found 308.0839.

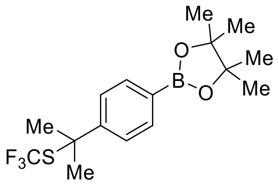

4,4,5,5-tetramethyl-2-(4-(2-((trifluoromethyl)thio)propan-2-yl)phenyl)-1,3,2-dioxaborolane (**3dd**). According to the general procedure in 0.2 mmol scale using 1.3 equiv $PhthSCF_3$ reagent with reaction time of 12 h; 26.2 mg, 38%, purified by flash chromatography (petroleum ether/ethylacetate = 50/1), colorless oil; Rf = 0.3 (petroleum ether/ethylacetate = 20/1); ^{1}H NMR (400 MHz, $CDCl_3$) δ 7.79 (d, J = 7.9 Hz, 2H), 7.55 (d, J = 7.9 Hz, 2H), 1.88 (s, 6H), 1.34 (s, 12H); ^{19}F NMR (376 MHz, $CDCl_3$) δ -36.91; ^{13}C NMR (101 MHz, $CDCl_3$) δ 147.4, 134.8, 130.3 (q, J = 309.4 Hz), 125.5, 83.9, 52.7, 30.7, 24.9. IR (ATR): ν = 2980, 2928, 2351, 1612, 1391, 1362, 1137, 1115, 1094, 859, 751, 659 cm^{-1}. HRMS (EI) calcd for $C_{16}H_{22}BF_3O_2S$ (M^+): 345.1422, found 345.1420.

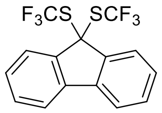

(9H-fluorene-9,9-diyl)bis((trifluoromethyl)sulfane) (**3ee**). According to the general procedure in 0.1 mmol scale using 2.3 equiv $PhthSCF_3$ reagent with reaction time of 36 h in anhydrous MeCN (4 mL); 17.7 mg, 48%, purified by flash chromatography (hexane), white solid; Rf = 0.7 (hexane); ^{1}H NMR (400 MHz, $CDCl_3$) δ 7.77 (dd, J = 15.2, 7.6 Hz, 4H), 7.49 (td, J = 7.5, 1.1 Hz, 2H), 7.41 (td, J = 7.6, 1.2 Hz, 2H); ^{19}F NMR (376 MHz, $CDCl_3$) δ -38.16; ^{13}C NMR (101 MHz, $CDCl_3$) δ 142.9, 138.6, 130.5, 128.3, 128.2 (q, J = 313.4 Hz), 125.7, 120.8, 63.2. IR (ATR): ν = 3064, 2921, 1451, 1145, 1110, 1098, 742 cm^{-1}. HRMS (EI) calcd for $C_{15}H_8F_6S_2$ (M^+): 365.9972, found 365.9977.

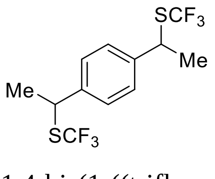

1,4-bis(1-((trifluoromethyl)thio)ethyl)benzene (**3ff**). According to the general procedure in 0.2 mmol scale using 2.5 equiv $PhthSCF_3$ reagent with reaction time of 24 h; 34.2 mg, 51%, purified by flash chromatography (hexane), colorless oil; Rf = 0.5 (hexane); ^{1}H NMR (500 MHz, $CDCl_3$) δ 7.37 (d, J = 1.5 Hz, 1H), 7.33 (d, J = 1.5 Hz, 3H), 4.51 (q, J = 7.1 Hz, 2H), 1.72 (d, J = 7.0 Hz, 6H); ^{19}F NMR (471 MHz, $CDCl_3$) δ -40.16; ^{13}C NMR (126 MHz, $CDCl_3$) δ 141.1, 130.4 (q, J = 307.4 Hz), 127.8, 127.6, 127.5, 44.1 (d, J = 0.8 Hz), 22.9. IR (ATR): ν = 22981, 2359, 1111, 838, 750 cm^{-1}. HRMS (EI) calcd for $C_{12}H_{12}F_6S_2$ (M^+): 334.0285, found 334.0296.

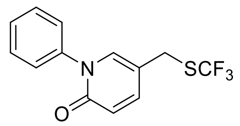

1-phenyl-5-(((trifluoromethyl)thio)methyl)pyridin-2(1H)-one (**4**). According to the general procedure in 0.1 mmol scale using 1.5 equiv $PhthSCF_3$ reagent with reaction time of 24 h; 18.5 mg, 63%, purified by flash chromatography (petroleum ether/ethylacetate = 2/1), colorless oil; Rf = 0.4 (petroleum ether/ethylacetate = 1/1); ^{1}H NMR (400 MHz, $CDCl_3$) δ 7.50 (dd, *J* = 8.3, 6.6 Hz, 2H), 7.47 – 7.39 (m, 2H), 7.39 – 7.32 (m, 3H), 6.69 (d, *J* = 9.5 Hz, 1H), 3.91 (s, 2H); ^{19}F NMR (376 MHz, $CDCl_3$) δ -41.10; ^{13}C NMR (101 MHz, $CDCl_3$) δ 161.6, 140.5, 137.2, 134.3, 130.4 (q, *J* = 308.4 Hz), 129.5, 128.7, 126.5, 122.5, 113.1, 31.2 (q, *J* = 2.5 Hz). IR (ATR): ν = 3067, 2929, 1671, 1591, 1494, 1456, 1268, 1107, 831, 757, 694 cm^{-1}. HRMS m/z (ESI) calcd for $C_{13}H_{11}F_3NOS^+$ $(M + H)^+$ 286.0508; found: 286.0506.

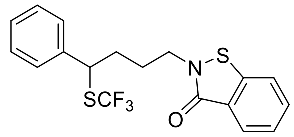

2-(4-phenyl-4-((trifluoromethyl)thio)butyl)benzo[d]isothiazol-3(2H)-one (**5**). According to the general procedure in 0.2 mmol scale using 1.3 equiv $PhthSCF_3$ reagent with reaction time of 12 h; 52.5 mg, 69%, purified by flash chromatography (petroleum ether/ethylacetate = 100/1), colorless oil; Rf = 0.5 (petroleum ether/ethylacetate = 50/1); ^{1}H NMR (400 MHz, $CDCl_3$) δ 7.88 (d, *J* = 8.0 Hz, 1H), 7.76 (d, *J* = 8.2 Hz, 1H), 7.54 – 7.48 (m, 1H), 7.41 – 7.27 (m, 6H), 4.52 (t, *J* = 6.3 Hz, 2H), 4.40 (dd, *J* = 9.1, 6.4 Hz, 1H), 2.32 – 2.11 (m, 2H), 1.99 – 1.77 (m, 2H) ^{19}F NMR (376 MHz, $CDCl_3$) δ -39.73; ^{13}C NMR (101 MHz, $CDCl_3$) δ 162.9, 151.6, 140.1, 130.4 (q, *J* = 308.4 Hz), 128.9, 128.7, 128.1, 127.4, 125.2, 124.4, 123.0, 120.2, 67.7, 49.3, 33.1, 26.8. IR (ATR): ν = 3063, 3031, 2953, 1570, 1507, 1427, 1345, 1103, 712, 653, 497 cm^{-1}. HRMS m/z (ESI) calcd for $C_{18}H_{17}F_3NOS_2^+$ $(M + H)^+$ 384.0698; found: 384.0692.

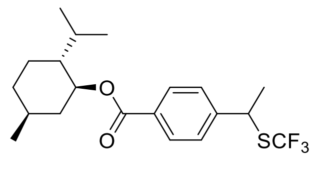

(1S,2R,5S)-2-isopropyl-5-methylcyclohexyl 4-(1-((trifluoromethyl)thio)ethyl)benzoate (**6**). According to the general procedure in 0.2 mmol scale using 1.3 equiv $PhthSCF_3$ reagent with reaction time of 12 h; 41.1 mg, 53%, purified by flash chromatography (petroleum ether/ethylacetate = 50/1), colorless oil; Rf = 0.5 (petroleum ether/ethylacetate = 20/1). ^{1}H NMR (400 MHz, $CDCl_3$) δ 8.03 (d, *J* = 8.4 Hz, 2H), 7.42 (d, *J* = 8.3 Hz, 2H), 4.93 (td, *J* = 10.9, 4.4 Hz, 1H), 4.55 (q, *J* = 7.1 Hz, 1H), 2.11 (ddt, *J* = 9.3, 4.7, 2.4 Hz, 1H), 1.95 (pd, *J* = 7.0, 2.8 Hz, 1H), 1.77 – 1.69 (m, 5H), 1.59 – 1.50 (m, 2H), 1.17 – 1.06 (m, 2H), 0.92 (dd, *J* = 6.8, 4.2 Hz, 7H), 0.79 (d, *J* = 6.9 Hz, 3H); ^{19}F NMR (376 MHz, $CDCl_3$) δ -40.12; ^{13}C NMR (101 MHz, $CDCl_3$) δ 165.6, 146.4 (d, *J* = 2.5 Hz),

130.5, 130.3 (q, J = 308.4 Hz), 130.1, 127.0, 75.0, 47.3, 44.1 (d, J = 1.8 Hz), 41.0, 34.3, 31.5, 26.5, 23.6, 22.8, 22.0, 20.8, 16.5. IR (ATR): ν = 2956, 2929, 2871, 1713, 1611, 1455, 1286, 1105, 982, 774, 756, 708 cm^{-1}. HRMS m/z (ESI) calcd for $C_{20}H_{27}F_3NaO_2S^+$ $(M + Na)^+$ 411.1576; found: 411.1581.

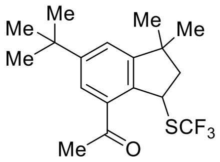

1-(6-(tert-butyl)-1,1-dimethyl-3-((trifluoromethyl)thio)-2,3-dihydro-1H-inden-4-yl)ethan-1-one (**7**). According to the general procedure in 0.2 mmol scale using 1.3 equiv $PhthSCF_3$ reagent with reaction time of 12 h; 42.8 mg, 62%, purified by flash chromatography (petroleum ether/ethylacetate = 50/1), ; Rf = 0.8 (petroleum ether/ethylacetate = 20/1); ^1H NMR (400 MHz, $CDCl_3$) δ 7.69 (d, J = 1.8 Hz, 1H), 7.36 (d, J = 1.8 Hz, 1H), 5.52 (dd, J = 5.8, 2.6 Hz, 1H), 2.63 (s, 3H), 2.48 (d, J = 5.9 Hz, 2H), 1.41 (s, 3H), 1.37 (s, 9H), 1.35 (s, 3H); ^{19}F NMR (376 MHz, $CDCl_3$) δ -41.11; ^{13}C NMR (101 MHz, $CDCl_3$) δ 199.5, 154.8, 153.1, 134.9, 134.5, 130.8 (q, J = 308.7 Hz), 125.8, 123.5, 49.1, 46.4, 43.2, 35.0, 31.4, 31.1, 29.6, 28.4. IR (ATR): ν = 2965, 2870, 2360, 1688, 1464, 1365, 1235, 1112, 764, 755 cm^{-1}. HRMS m/z (ESI) calcd for $C_{18}H_{23}F_3NaOS^+$ $(M + Na)^+$ 367.1314; found: 367.1311.

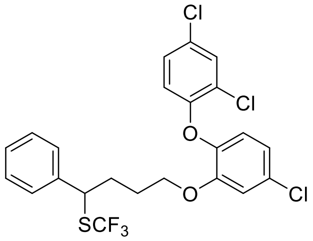

(4-(5-chloro-2-(2,4-dichlorophenoxy)phenoxy)-1-phenylbutyl)(trifluoromethyl)sulfane (**8**). According to the general procedure in 0.2 mmol scale using 1.3 equiv $PhthSCF_3$ reagent with reaction time of 12 h; 47.8 mg, 46%, purified by flash chromatography (petroleum ether/ethylacetate = 50/1), colorless oil; Rf = 0.6 (petroleum ether/ethylacetate = 20/1); ^1H NMR (400 MHz, $CDCl_3$) δ 7.40 (s, 1H), 7.31 (dt, J = 13.9, 7.0 Hz, 3H), 7.21 (d, J = 7.5 Hz, 2H), 7.06 (d, J = 8.8 Hz, 1H), 7.00 – 6.86 (m, 3H), 6.58 (d, J = 8.8 Hz, 1H), 4.21 (t, J = 7.8 Hz, 1H), 3.86 (t, J = 5.8 Hz, 2H), 1.87 (dt, J = 13.7, 7.2 Hz, 2H), 1.72 – 1.50 (m, 2H); ^{19}F NMR (376 MHz, $CDCl_3$) δ -39.79; ^{13}C NMR (101 MHz, $CDCl_3$) δ 152.5, 150.7, 142.7, 140.1, 130.7, 130.4 (q, J = 308.4 Hz), 130.3, 128.8, 128.0, 127.8, 127.6, 127.3, 124.1, 122.4, 121.3, 117.3, 114.7, 68.1, 49.2 (d, J = 2.0 Hz), 32.8, 26.9. IR (ATR): ν = 2930, 2873, 1599, 1471, 1454, 1268, 1230, 1099, 865, 808, 754, 696, 589 cm^{-1}. HRMS m/z (ESI) calcd for $C_{23}H_{18}Cl_3F_3NaO_2S^+$ $(M + Na)^+$ 542.9937; found: 542.9934.

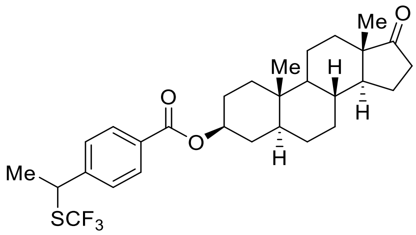

(3S,5S,8R,10S,13S,14S)-10,13-dimethyl-17-oxohexadecahydro-1H-cyclopenta[a]phenanthren-3-yl 4-(1-((trifluoromethyl)thio)ethyl)benzoate (**9**). According to the general procedure in 0.1 mmol scale using 1.5 equiv $PhthSCF_3$ reagent with reaction time of 24 h; 37.1 mg, 71%, purified by flash chromatography (petroleum ether/ethylacetate = 10/1), white solid; Rf = 0.5 (petroleum ether/ethylacetate = 5/1); ^1H NMR (400 MHz, $CDCl_3$) δ 8.01 (d, *J* = 8.3 Hz, 2H), 7.42 (d, *J* = 8.3 Hz, 2H), 4.94 (tt, *J* = 11.3, 4.9 Hz, 1H), 4.55 (q, *J* = 7.1 Hz, 1H), 2.44 (dd, *J* = 19.2, 8.6 Hz, 1H), 2.08 (dt, *J* = 19.2, 9.0 Hz, 1H), 2.01 – 1.90 (m, 2H), 1.85 – 1.45 (m, 12H), 1.40 – 1.25 (m, 6H), 1.17 – 0.98 (m, 2H), 0.90 (s, 3H), 0.87 (s, 3H), 0.76 (ddd, *J* = 11.9, 10.2, 3.9 Hz, 1H); ^{19}F NMR (376 MHz, $CDCl_3$) δ -40.11; ^{13}C NMR (101 MHz, $CDCl_3$) δ 221.2, 165.6, 146.4, 130.5, 130.2 (q, *J* = 308.4 Hz), 130.1, 127.0, 74.3, 54.3, 51.4, 47.8, 44.7, 44.1 (d, *J* = 1.9 Hz), 36.7, 35.9, 35.7, 35.1, 34.0, 31.5, 30.8, 28.3, 27.5, 22.8, 21.8, 20.5, 13.8, 12.3. IR (ATR): ν = 2932, 2855, 1738, 1713, 1451, 1274, 1108, 1013, 775, 709 cm^{-1}. HRMS m/z (ESI) calcd for $C_{29}H_{37}F_3NaO_3S^+$ $(M + Na)^+$ 545.2308; found: 545.2299.

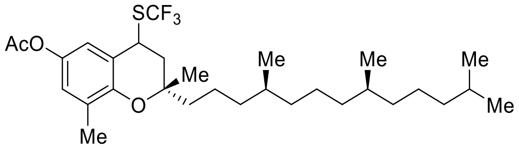

(2S)-2,8-dimethyl-4-((trifluoromethyl)thio)-2-((4S,8S)-4,8,12-trimethyltridecyl)chroman-6-yl acetate (**10**). According to the general procedure in 0.1 mmol scale using 1.5 equiv $PhthSCF_3$ reagent with reaction time of 24 h; 16.9 mg, 31%, purified by flash chromatography (petroleum ether/ethylacetate = 50/1), colorless oil; Rf = 0.6 (petroleum ether/ethylacetate = 20/1); ^1H NMR (400 MHz, $CDCl_3$) δ 7.07 (d, *J* = 2.8 Hz, 1H), 6.79 (d, *J* = 2.8 Hz, 1H), 4.54 (dd, *J* = 9.2, 6.4 Hz, 1H), 2.45 (dd, *J* = 14.2, 6.4 Hz, 1H), 2.27 (s, 3H), 2.21 – 2.16 (m, 1H), 2.15 (s, 3H), 1.51 (dd, *J* = 12.3, 6.0 Hz, 3H), 1.41 – 1.19 (m, 15H), 1.16 – 1.01 (m, 6H), 0.85 (dd, *J* = 11.0, 6.6 Hz, 12H); ^{19}F NMR (376 MHz, $CDCl_3$) δ -40.03; ^{13}C NMR (101 MHz, $CDCl_3$) δ 170.0, 149.9, 142.9, 131.0 (q, *J* = 308.4 Hz), 128.4, 123.8, 119.3, 118.1, 76.9, 41.0, 39.4, 38.4, 37.9, 37.4, 37.3 (d, *J* = 3.7 Hz), 37.2, 32.8, 32.6, 28.0, 25.6, 24.8, 24.4, 22.7, 22.6, 21.1, 21.0, 19.7, 19.6, 16.3. IR (ATR): ν = 2952, 2526, 2868, 1767, 1469, 1199, 1110, 1017, 931, 756 cm^{-1}. HRMS m/z (ESI) calcd for $C_{30}H_{47}F_3NaO_3S^+$ $(M + Na)^+$ 567.3090; found: 567.3089.

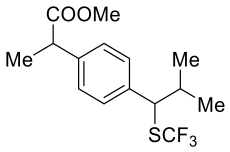

methyl 2-(4-(2-methyl-1-((trifluoromethyl)thio)propyl)phenyl)propanoate (**11**). According to the general procedure in 0.2 mmol scale using 1.3 equiv $PhthSCF_3$ reagent with reaction time of 12 h; 52.2 mg, 81%, purified by flash chromatography (petroleum ether/ethylacetate = 50/1), colorless oil; Rf = 0.5 (petroleum ether/ethylacetate = 20/1); ^1H NMR (500 MHz, $CDCl_3$) δ 7.24 (q, *J* = 8.4 Hz, 4H), 4.07 (d, *J* = 7.3 Hz, 1H), 3.72 (q, *J* = 7.2 Hz, 1H), 3.67 (s, 3H), 2.18 – 2.08 (m, *J* = 6.7 Hz, 1H), 1.49 (d, *J* = 7.2 Hz, 3H), 1.05 (d, *J* = 6.7 Hz, 3H), 0.90 (d, *J* = 6.7 Hz, 3H); ^{19}F NMR (471 MHz, $CDCl_3$) δ -39.90; ^{13}C NMR (126 MHz, $CDCl_3$) δ 174.9 (d, *J* = 1.2 Hz), 139.7 (d, *J* = 1.7 Hz), 139.3, 130.8 (q, *J* = 307.0 Hz), 128.2, 127.4, 56.7 (d, *J* = 0.6 Hz), 52.0, 45.0, 34.0, 20.5, 20.4, 18.5. IR (ATR): ν = 2966, 2876, 1737, 1512, 1333, 1208, 1101, 861, 755, 543 cm^{-1}. HRMS m/z (ESI) calcd for $C_{15}H_{19}F_3NaO_2S^+$ $(M + Na)^+$ 343.0950; found: 343.0952.

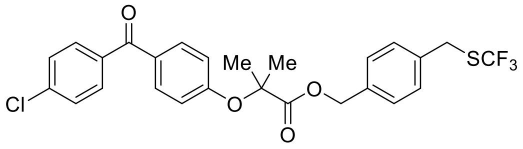

4-(((trifluoromethyl)thio)methyl)benzyl 2-(4-(4-chlorobenzoyl)phenoxy)-2-methylpropanoate (**12**). According to the general procedure in 0.2 mmol scale using 1.3 equiv $PhthSCF_3$ reagent with reaction time of 12 h; 54.3 mg, 52%, purified by flash chromatography (petroleum ether/ethylacetate = 20/1), colorless oil; Rf = 0.4 (petroleum ether/ethylacetate = 10/1); ^1H NMR (400 MHz, $CDCl_3$) δ 7.67 (dd, *J* = 20.6, 8.7 Hz, 4H), 7.44 (d, *J* = 8.5 Hz, 2H), 7.30 – 7.19 (m, 4H), 6.78 (d, *J* = 8.8 Hz, 2H), 5.18 (s, 2H), 4.07 (s, 2H), 1.68 (s, 6H); ^{19}F NMR (376 MHz, $CDCl_3$) δ -41.53; ^{13}C NMR (101 MHz, $CDCl_3$) δ 194.1, 173.4, 159.4, 138.4, 136.3, 135.5, 134.9, 131.9, 131.1, 130.5 (q, *J* = 308.4 Hz), 130.3, 129.0, 128.9, 128.5, 117.2, 79.4, 66.8, 33.8 (q, *J* = 2.4 Hz), 25.4. IR (ATR): ν = 2994, 2943, 1736, 1654, 1597, 1466, 1248, 1104, 926, 740, 478 cm^{-1}. HRMS m/z (ESI) calcd for $C_{26}H_{22}ClF_3NaO_4S^+$ $(M + Na)^+$ 545.0772; found: 545.0771.

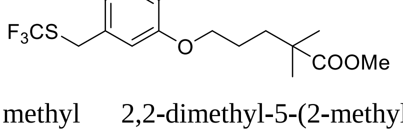

methyl 2,2-dimethyl-5-(2-methyl-5-(((trifluoromethyl)thio)methyl)phenoxy)pentanoate (**13**). According to the general procedure in 0.2 mmol scale using 1.3 equiv $PhthSCF_3$ reagent with reaction time of 12 h; 38.2 mg, 53%, purified by flash chromatography (petroleum ether/ethylacetate = 50/1), colorless oil; Rf = 0.5 (petroleum ether/ethylacetate = 20/1); ^1H NMR (400 MHz, $CDCl_3$) δ 7.12 (d, *J* = 7.6 Hz, 1H), 6.71 (d, *J* = 7.6 Hz, 1H), 6.65 (s, 1H), 4.08 (s, 2H), 3.96 (p, *J* = 2.6 Hz, 2H), 3.67 (s, 3H), 2.33 (s, 3H), 1.74 (d, *J* = 2.9 Hz, 4H), 1.22 (s, 6H); ^{19}F NMR (376 MHz, $CDCl_3$) δ -41.68; ^{13}C NMR (101 MHz, $CDCl_3$) δ 178.3, 156.6, 139.7, 131.2 (q, *J* = 307.7 Hz), 130.1, 121.1,

120.8, 112.3, 68.0, 51.7, 42.1, 37.1, 29.1 (q, J = 2.3 Hz), 25.2, 25.0, 21.6. IR (ATR): ν = 2952, 2873, 1731, 1508, 1268, 1144, 1110, 814, 753, 456 cm^{-1}. HRMS m/z (ESI) calcd for $C_{17}H_{23}F_3NaO_3S^+$ (M + Na)$^+$ 387.1212; found: 387.1212.

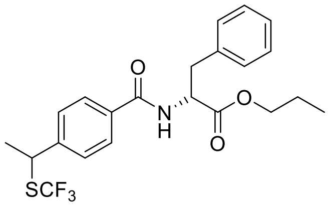

propyl (4-(1-((trifluoromethyl)thio)ethyl)benzoyl)-D-phenylalaninate (**14**). According to the general procedure in 0.2 mmol scale using 1.3 equiv $PhthSCF_3$ reagent with reaction time of 12 h; 43.1 mg, 49%, purified by flash chromatography (petroleum ether/ethylacetate = 10/1), colorless oil; Rf = 0.5 (petroleum ether/ethylacetate = 5/1); ^{1}H NMR (400 MHz, $CDCl_3$) δ 7.73 – 7.68 (m, 2H), 7.43 – 7.37 (m, 2H), 7.32 – 7.24 (m, 3H), 7.18 – 7.09 (m, 2H), 6.63 (d, J = 7.6 Hz, 1H), 5.07 (dt, J = 7.6, 5.7 Hz, 1H), 4.53 (q, J = 7.1 Hz, 1H), 4.18 – 4.04 (m, 2H), 3.33 – 3.18 (m, 2H), 1.70 (d, J = 7.3 Hz, 3H), 1.68 – 1.63 (m, 2H), 0.93 (t, J = 7.4 Hz, 3H); ^{19}F NMR (376 MHz, $CDCl_3$) δ -40.10; ^{13}C NMR (101 MHz, $CDCl_3$) δ 171.6, 166.2, 145.3, 135.8, 133.6, 130.2 (q, J = 308.7 Hz), 129.3, 128.6, 127.6, 127.3, 127.1, 67.2, 53.6, 44.0 (d, J = 2.3 Hz), 37.9, 22.7, 21.8, 10.3. IR (ATR): ν = 3324, 3031, 2970, 2936, 2880, 1738, 1612, 1536, 1497, 1108, 853, 756, 700 cm^{-1}. HRMS m/z (ESI) calcd for $C_{22}H_{25}F_3NO_3S^+$ (M + H)$^+$ 440.1502; found: 440.1499.

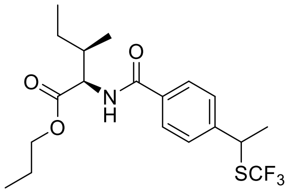

propyl (4-(1-((trifluoromethyl)thio)ethyl)benzoyl)-D-isoleucinate (**15**). According to the general procedure in 0.2 mmol scale using 1.3 equiv $PhthSCF_3$ reagent with reaction time of 12 h; 64.1 mg, 78%, purified by flash chromatography (petroleum ether/ethylacetate = 10/1), colorless oil; Rf = 0.5 (petroleum ether/ethylacetate = 5/1); ^{1}H NMR (400 MHz, $CDCl_3$) δ 7.82 – 7.76 (m, 2H), 7.46 – 7.40 (m, 2H), 6.74 (d, J = 8.4 Hz, 1H), 4.82 (dd, J = 8.5, 4.8 Hz, 1H), 4.55 (q, J = 7.1 Hz, 1H), 4.20 – 4.08 (m, 2H), 2.02 (dqt, J = 9.4, 6.9, 4.7 Hz, 1H), 1.70 (dd, J = 12.4, 7.0 Hz, 5H), 1.63 – 1.48 (m, 1H), 1.27 (dddd, J = 16.4, 12.3, 8.2, 6.3 Hz, 1H), 0.97 (t, J = 7.4 Hz, 9H); ^{19}F NMR (376 MHz, $CDCl_3$) δ -40.13; ^{13}C NMR (101 MHz, $CDCl_3$) δ 172.2, 166.4, 145.3, 133.8, 130.2 (q, J = 308.7 Hz), 127.6, 127.2, 67.0, 56.8, 44.0, 38.3, 25.4, 22.7, 21.9, 15.5, 11.6, 10.4. IR (ATR): ν = 3336, 2968, 2937, 2880, 2350, 1737, 1646, 1612, 1573, 1114, 756 cm^{-1}. HRMS m/z (ESI) calcd for $C_{19}H_{26}F_3NNaO_3S^+$ (M + Na)$^+$ 428.1478; found: 428.1475.

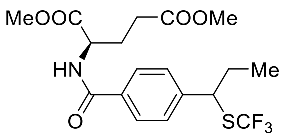

dimethyl (4-(1-((trifluoromethyl)thio)propyl)benzoyl)-D-glutamate (**16**). According to the general procedure in 0.2 mmol scale using 1.3 equiv $PhthSCF_3$ reagent with reaction time of 12 h; 53.2 mg, 63%, purified by flash chromatography (petroleum ether/ethylacetate = 10/1), colorless oil; Rf = 0.4 (petroleum ether/ethylacetate = 3/1); ^{1}H NMR (400 MHz, $CDCl_3$) δ 7.81 (d, *J* = 8.3 Hz, 2H), 7.39 (d, *J* = 8.3 Hz, 2H), 7.04 (d, *J* = 7.5 Hz, 1H), 4.82 (td, *J* = 7.7, 4.9 Hz, 1H), 4.23 (dd, *J* = 8.8, 6.5 Hz, 1H), 3.79 (s, 3H), 3.66 (s, 3H), 2.49 (qt, *J* = 16.9, 7.1 Hz, 2H), 2.33 (dtd, *J* = 14.3, 7.1, 4.9 Hz, 1H), 2.18 (tq, *J* = 14.6, 7.3 Hz, 1H), 2.08 – 1.89 (m, 2H), 0.92 (t, *J* = 7.3 Hz, 3H); ^{19}F NMR (376 MHz, $CDCl_3$) δ -39.75; ^{13}C NMR (101 MHz, $CDCl_3$) δ 173.8, 172.4, 166.6, 144.8, 133.1, 130.3 (q, *J* = 308.4 Hz), 127.8, 127.6, 52.7, 52.3, 52.0, 50.8 (d, *J* = 1.3 Hz), 30.2, 29.6, 27.1, 11.9. IR (ATR): ν = 3339, 2955, 2358, 2329, 1740, 1646, 1537, 1213, 1150, 1114, 764, 750 cm^{-1}. HRMS m/z (ESI) calcd for $C_{18}H_{23}F_3NO_5S^+$ $(M + H)^+$ 422.1244; found: 422.1240.

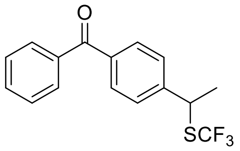

phenyl(4-(1-((trifluoromethyl)thio)ethyl)phenyl)methanone (**17**). According to the general procedure in 0.2 mmol scale using 1.3 equiv $PhthSCF_3$ reagent with reaction time of 12 h; 42.8 mg, 69%, purified by flash chromatography (petroleum ether/ethylacetate = 50/1), colorless oil; Rf = 0.6 (petroleum ether/ethylacetate = 20/1); ^{1}H NMR (400 MHz, $CDCl_3$) δ 7.80 (d, *J* = 7.9 Hz, 4H), 7.62 – 7.57 (m, 1H), 7.52 – 7.45 (m, 4H), 4.58 (q, *J* = 7.1 Hz, 1H), 1.75 (d, *J* = 7.1 Hz, 3H); ^{19}F NMR (376 MHz, $CDCl_3$) δ -40.07; ^{13}C NMR (101 MHz, $CDCl_3$) δ 196.0, 146.1, 137.4, 137.2, 132.5, 130.6, 130.3 (q, *J* = 308.7 Hz), 130.0, 128.3, 127.0, 44.1 (d, *J* = 1.9 Hz), 22.8. IR (ATR): ν = 3062, 2978, 2931, 2350, 1658, 1607, 1276, 1102, 1048, 938, 823, 851, 698, 575 cm^{-1}. HRMS m/z (ESI) calcd for $C_{16}H_{13}F_3NaOS^+$ $(M + Na)^+$ 333.0531; found: 333.0527.

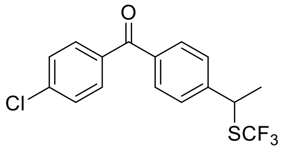

(4-chlorophenyl)(4-(1-((trifluoromethyl)thio)ethyl)phenyl)methanone (**18**). According to the general procedure in 0.2 mmol scale using 1.3 equiv $PhthSCF_3$ reagent with reaction time of 12 h; 50.2 mg, 73%, purified by flash chromatography (petroleum ether/ethylacetate = 50/1), colorless oil; Rf = 0.3 (petroleum ether/ethylacetate = 20/1); ^{1}H NMR (400 MHz, $CDCl_3$) δ 7.76 (dd, *J* = 8.4, 7.2 Hz, 4H), 7.48 (dd, *J* = 8.4, 5.3 Hz, 4H), 4.58 (q, *J* = 7.1 Hz, 1H), 1.75 (d, *J* = 7.1 Hz, 3H); ^{19}F NMR (376 MHz, $CDCl_3$) δ -40.07; ^{13}C NMR (101 MHz, $CDCl_3$) δ 194.7, 146.4, 139.0, 136.8, 135.7, 131.4, 130.5, 130.2 (q, *J* = 308.4 Hz), 128.7, 127.1, 44.1, 22.8. IR (ATR): ν = 2978, 2932, 2358, 1660, 1607, 1589, 1285, 1274, 1111, 928, 856, 768 cm^{-1}. HRMS m/z (ESI) calcd for

$C_{16}H_{12}ClF_3NaOS^+$ $(M + Na)^+$ 367.0142; found: 367.0138.

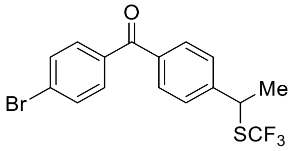

(4-bromophenyl)(4-(1-((trifluoromethyl)thio)ethyl)phenyl)methanone (**19**). According to the general procedure in 0.2 mmol scale using 1.3 equiv $PhthSCF_3$ reagent with reaction time of 12 h; 49.8 mg, 64%, purified by flash chromatography (petroleum ether/ethylacetate = 50/1), colorless oil; Rf = 0.3 (petroleum ether/ethylacetate = 20/1); ^{1}H NMR (400 MHz, $CDCl_3$) δ 7.77 (d, J = 8.3 Hz, 2H), 7.69 – 7.61 (m, 4H), 7.48 (d, J = 8.3 Hz, 2H), 4.58 (q, J = 7.1 Hz, 1H), 1.75 (d, J = 7.0 Hz, 3H); ^{19}F NMR (376 MHz, $CDCl_3$) δ -40.06; ^{13}C NMR (101 MHz, $CDCl_3$) δ 194.9, 146.4, 136.7, 136.1, 131.7, 131.5, 130.5, 130.2 (q, J = 308.7 Hz), 127.7, 127.1, 44.1 (q, J = 2.0 Hz), 22.8. IR (ATR): ν = 2978, 2931, 2351, 1660, 1607, 1585, 1282, 1106, 927, 855, 834, 756, 678 cm^{-1}. HRMS m/z (ESI) calcd for $C_{16}H_{13}BrF_3OS^+$ $(M + H)^+$ 388.9817; found: 388.9813.

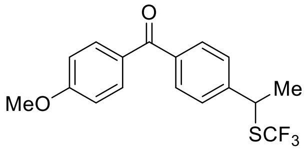

(4-methoxyphenyl)(4-(1-((trifluoromethyl)thio)ethyl)phenyl)methanone (**20**). According to the general procedure in 0.2 mmol scale using 1.3 equiv $PhthSCF_3$ reagent with reaction time of 12 h; 43.4 mg, 64%, purified by flash chromatography (petroleum ether/ethylacetate = 20/1), colorless oil; Rf = 0.4 (petroleum ether/ethylacetate = 10/1); ^{1}H NMR (400 MHz, $CDCl_3$) ^{1}H NMR (400 MHz, $CDCl_3$) δ 7.82 (d, J = 8.8 Hz, 2H), 7.75 (d, J = 8.3 Hz, 2H), 7.46 (d, J = 8.2 Hz, 2H), 6.97 (d, J = 8.9 Hz, 2H), 4.58 (q, J = 7.1 Hz, 1H), 3.89 (s, 3H), 1.75 (d, J = 7.1 Hz, 3H); ^{19}F NMR (376 MHz, $CDCl_3$) δ -40.07; ^{13}C NMR (101 MHz, $CDCl_3$) δ 194.8, 163.3, 145.5, 137.9, 132.5, 130.3, 130.3 (q, J = 308.7 Hz), 130.0, 126.9, 113.6, 55.5, 44.1, 22.8. IR (ATR): ν = 3005, 2360, 1652, 1601, 1276, 1260, 1113, 764, 750 cm^{-1}. HRMS m/z (ESI) calcd for $C_{17}H_{16}F_3O_2S^+$ $(M + H)^+$ 341.0818; found: 341.0818.

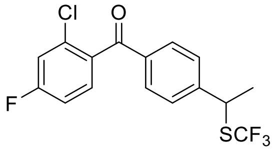

(2-chloro-4-fluorophenyl)(4-(1-((trifluoromethyl)thio)ethyl)phenyl)methanone (**21**). According to the general procedure in 0.2 mmol scale using 1.3 equiv $PhthSCF_3$ reagent with reaction time of 12 h; 47.0 mg, 65%, purified by flash chromatography (petroleum ether/ethylacetate = 50/1), colorless oil; Rf = 0.2 (petroleum ether/ethylacetate = 20/1); ^{1}H NMR (400 MHz, $CDCl_3$) δ 7.78 (d, J = 8.4 Hz, 2H), 7.46 (d, J = 8.3 Hz, 2H), 7.40 (dd, J = 8.5, 5.9 Hz, 1H), 7.22 (dd, J = 8.5, 2.4 Hz, 1H), 7.10

(td, J = 8.2, 2.4 Hz, 1H), 4.56 (q, J = 7.1 Hz, 1H), 1.73 (d, J = 7.1 Hz, 3H); ^{19}F NMR (376 MHz, $CDCl_3$) δ -40.08, -107.53 (td, J = 8.2, 6.0 Hz); ^{13}C NMR (101 MHz, $CDCl_3$) δ 193.6, 163.3 (d, J = 254.2 Hz), 147.6, 134.5 (d, J = 3.8 Hz), 133.0 (d, J = 10.6 Hz), 131.7, 131.0 (d, J = 9.3 Hz), 130.6, 130.2 (q, J = 308.7 Hz), 127.4, 117.7 (d, J = 24.9 Hz), 114.2 (d, J = 21.5 Hz), 44.0 (d, J = 2.1 Hz), 22.7. IR (ATR): ν = 2981, 2932, 2359, 1671, 1600, 1584, 1488, 1286, 1259, 1107, 1017, 938, 854, 831 cm^{-1}. HRMS m/z (ESI) calcd for $C_{16}H_{12}ClF_4OS^+$ $(M + H)^+$ 363.0228; found: 363.0227.

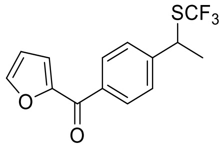

furan-2-yl(4-(1-((trifluoromethyl)thio)ethyl)phenyl)methanone (**22**). According to the general procedure in 0.1 mmol scale using 1.3 equiv $PhthSCF_3$ reagent with reaction time of 12 h; 15.3 mg, 51%, purified by flash chromatography (petroleum ether/ethylacetate = 50/1), colorless oil; Rf = 0.4 (petroleum ether/ethylacetate = 20/1); ^{1}H NMR (400 MHz, $CDCl_3$) δ 7.98 (d, J = 8.3 Hz, 2H), 7.74 – 7.70 (m, 1H), 7.49 (d, J = 8.3 Hz, 2H), 7.26 (d, J = 3.4 Hz, 1H), 6.61 (dd, J = 3.6, 1.7 Hz, 1H), 4.58 (q, J = 7.1 Hz, 1H), 1.75 (d, J = 7.0 Hz, 3H); ^{19}F NMR (376 MHz, $CDCl_3$) δ -40.08; ^{13}C NMR (101 MHz, $CDCl_3$) δ 181.7, 152.3, 147.1, 146.3, 136.8, 130.3 (q, J = 308.7 Hz), 129.9, 127.1, 120.6, 112.3, 44.1 (d, J = 1.9 Hz), 22.8. IR (ATR): ν = 2978, 2930, 2350, 1646, 1608, 1562, 1464, 1311, 1292, 1110, 1015, 853, 766, 757 cm^{-1}. HRMS m/z (ESI) calcd for $C_{14}H_{11}F_3NaO_2S^+$ $(M + Na)^+$ 323.0324; found: 323.0322.

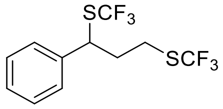

(1-phenylpropane-1,3-diyl)bis((trifluoromethyl)sulfane) (**23**). According to the general procedure in 0.1 mmol scale using 1.3 equiv $PhthSCF_3$ reagent with reaction time of 12 h; 8.2 mg, 26%, purified by flash chromatography (hexane), colorless oil; Rf = 0.7 (hexane); ^{1}H NMR (400 MHz, $CDCl_3$) δ 7.41 – 7.27 (m, 5H), 4.44 (dd, J = 8.9, 6.6 Hz, 1H), 2.88 (ddd, J = 13.9, 7.9, 6.0 Hz, 1H), 2.76 (dt, J = 14.3, 7.6 Hz, 1H), 2.38 (tt, J = 14.5, 6.6 Hz, 2H); ^{19}F NMR (376 MHz, $CDCl_3$) δ -39.81, -40.89; ^{13}C NMR (101 MHz, $CDCl_3$) δ 138.7, 130.8 (q, J = 309.4 Hz), 130.3 (q, J = 308.7 Hz), 129.2, 128.5, 127.3, 47.6, 36.2, 27.3 (d, J = 2.2 Hz). IR (ATR): ν = 3033, 2929, 2359, 1494, 1455, 1099, 754, 697 cm^{-1}. HRMS (EI) calcd for $C_{11}H_{10}F_6S_2$ (M^+): 320.0128, found 320.0130.

The selectivity experiments

Supplementary Table 3. The selectivity between benzylic 1°, 2° and 3° C-H bond

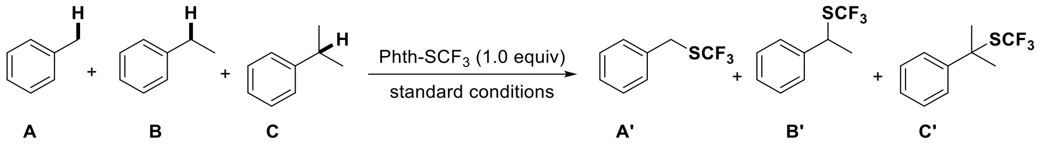

Entry	Reactants	Products	Ratio[b] (GC-MS)
1	A + B + C	A’ + B’ + C’	2.36/50.45/47.19
2	A + B	A’ + B’	5.45/94.55
3	A + C	A’ + C’	2.99/97.01
4	B + C	B’ + C’	52.4/47.6

a. reaction contion: reactants (0.1 mmol), Phth-SCF_3 (0.1 mmol), 4CzIPN (1.6 mg), K_2CO_3 (2.76 mg), anhydrous MeCN (1 mL), under blue LEDs irradiation for 12 h. b. detected by GC-MS.

The TIC of four competing reactions.

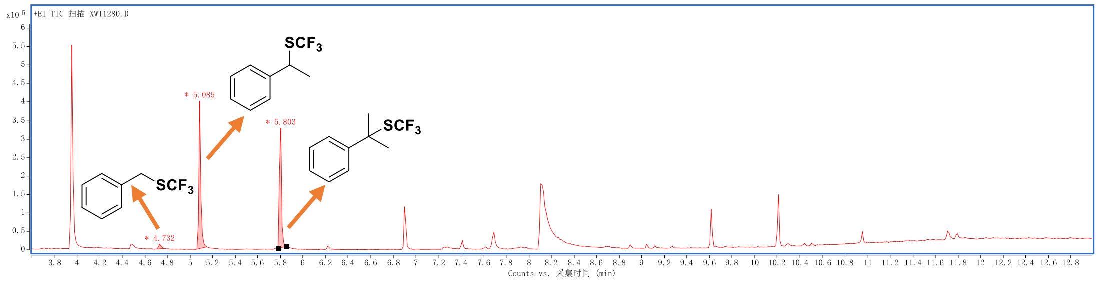

Supplementary Figure 2. TIC 1, entry 1 (A + B + C)

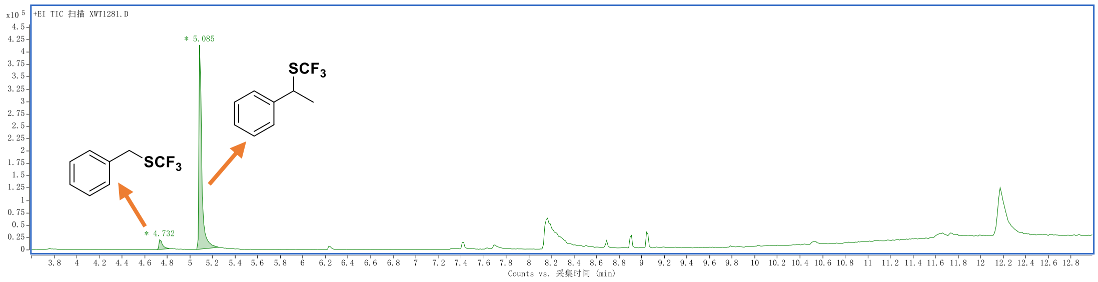

Supplementary Figure 3. TIC 2, entry 2 (A + B)

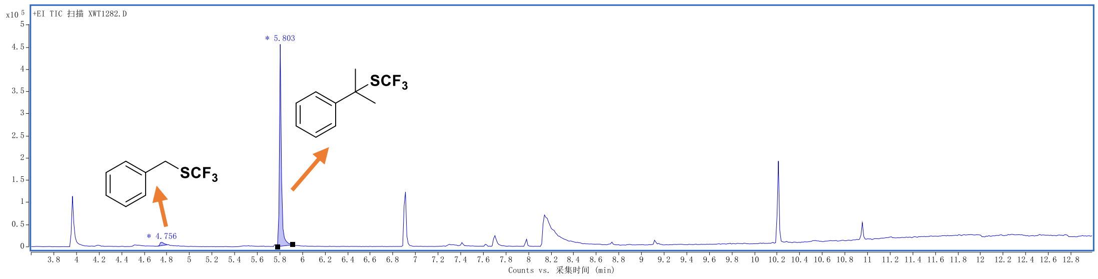

Supplementary Figure 4. TIC 3, entry 3 (A + C)

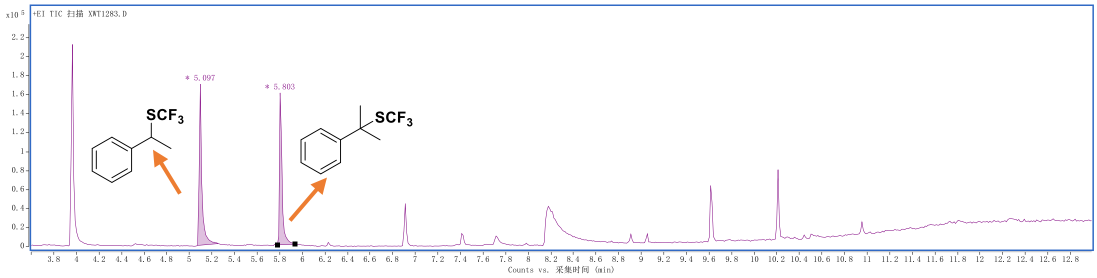

Supplementary Figure 5. TIC 4, entry 4 (B + C)

The mass spectrum of the corresponding trifluoromethylthiolation products (A', B', and C')

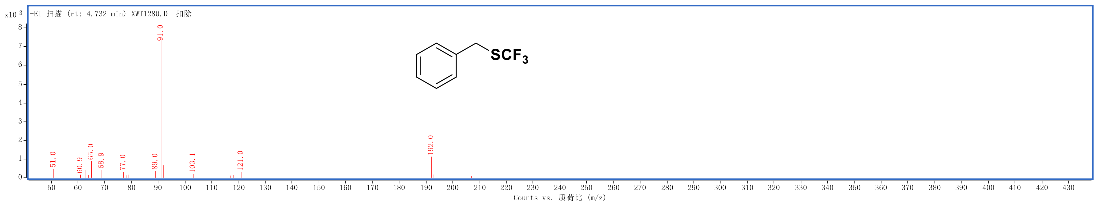

Supplementary Figure 6. RT: 4.732 min, m/z 192.0, benzyl(trifluoromethyl)sulfane (A')

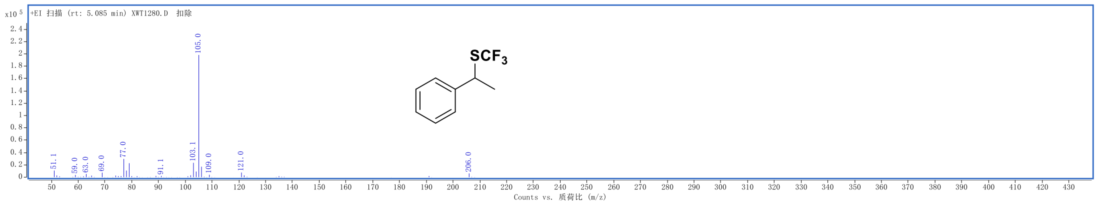

Supplementary Figure 7. RT: 5.085 min, m/z 206.0, (1-phenylethyl)(trifluoromethyl)sulfane (B')

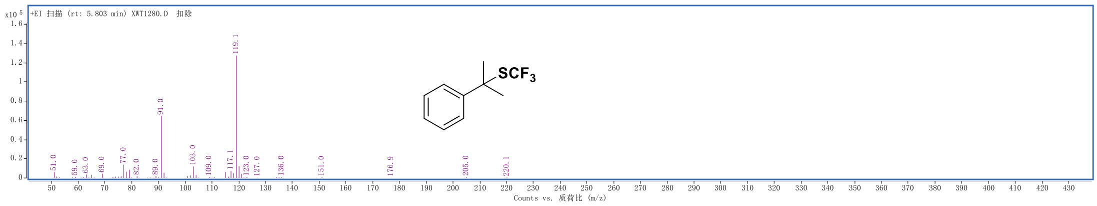

Supplementary Figure 8. RT: 5.803 min, m/z 220.1, (2-phenylpropan-2-yl)(trifluoromethyl)sulfane (C')

The parallel experiments under the present condition and the previous condition

The selected examples **1a** and **1k** were conducted under the optimal conditions and the previous condition, respectively. To our delight, the present method shows an excellent selectivity and efficiency for benzylic C-H trifluoromethylthiolation of **1a** (Fig. S9-a). In addition, **1k** could undergo C(sp^3)-H trifluoromethylthiolation, but with a much lower conversion and selectivity than the present condition (Fig. S9-b). These experiments perfectly exhibit the advantages in compatibility and selectivity by inner-sphere radical initiation mechanism in comparison with outer-sphere radical initiation mechanism [Qing's work: *Org. Lett.* **16**, 3372-3375 (2014); Glorius's work: *J. Am.*

Chem. Soc. **138**, 16200-16203 (2016)].

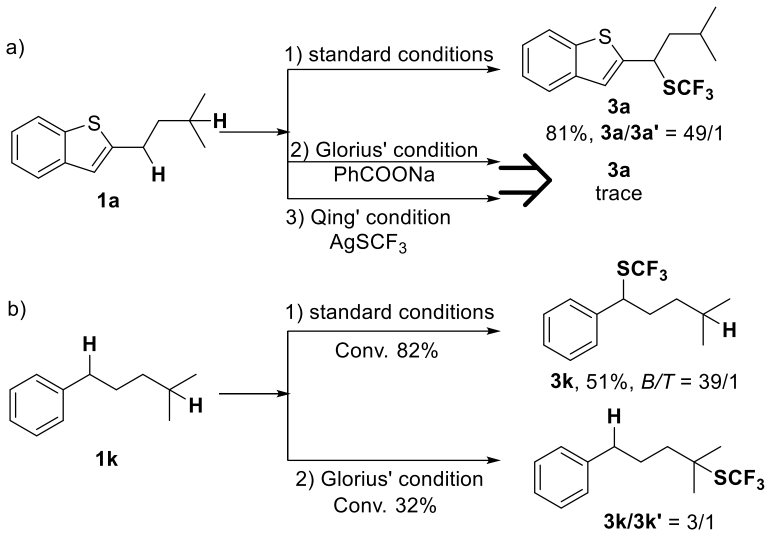

Supplementary Figure 9. The parallel experiments under the present condition and the previous condition

GC and GC-MS data:

a) GC method:

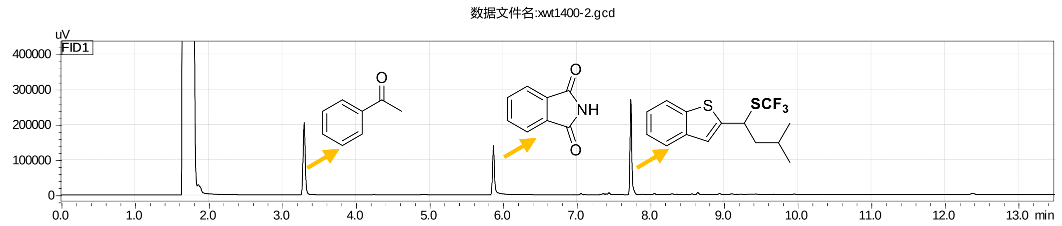

Supplementary Figure 10. standard conditions (a1)

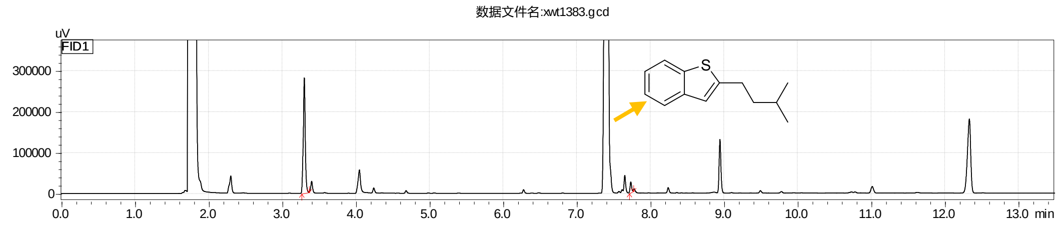

Supplementary Figure 11. Qing's condition (a2)

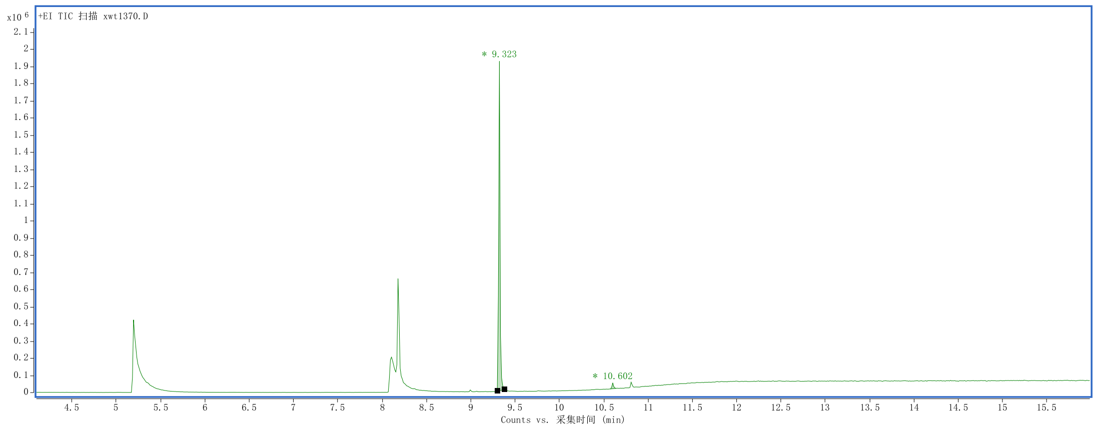

Supplementary Figure 12. Glorius' condition (a3)

b)

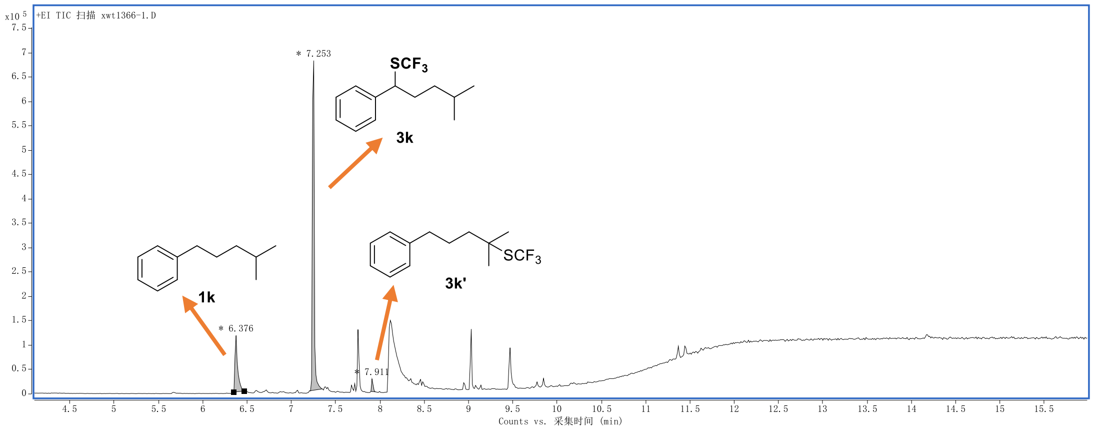

Supplementary Figure 13. standard condition (b1)

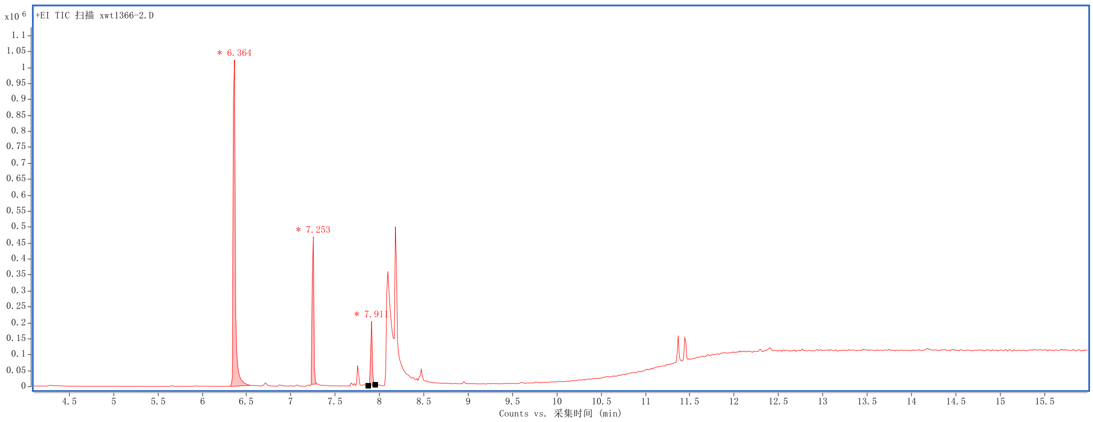

Supplementary Figure 14. Glorius' condition (b2)

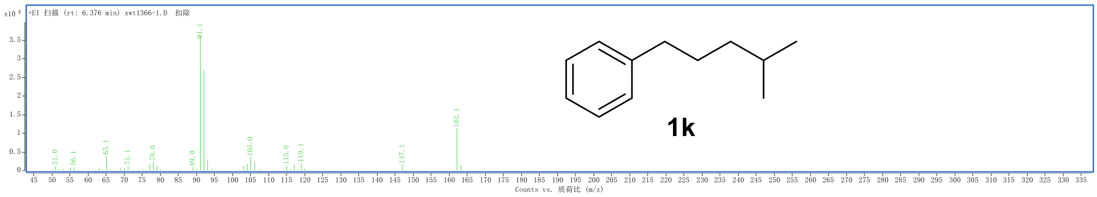

Supplementary Figure 15. RT: 6.376 min, m/z 162.0, (4-methylpentyl)benzene (**1k**)

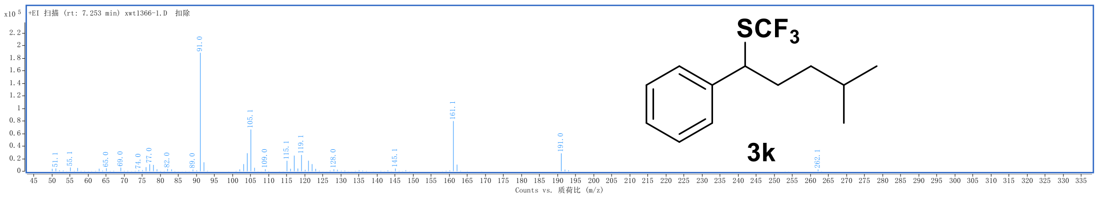

Supplementary Figure 16. RT: 7.253 min, m/z 262.1, (4-methyl-1-phenylpentyl)(trifluoromethyl)sulfane (**3k**)

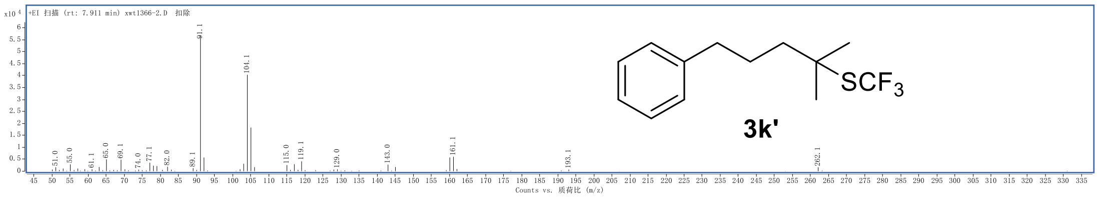

Supplementary Figure 17. RT: 7.911 min, m/z 262.1, (2-methyl-5-phenylpentan-2-yl)(trifluoromethyl)sulfane

Flow chemistry

General procedure. To further demonstrate the present protocol's value in synthesis application for medicinal chemists, we selected several complex molecules (like pirfenidone, used to treat idiopathic pulmonary fibrosis, ranked 126 of the top-selling 200 pharmaceutical products) to react in continuous flow in 1 mmol scale. To our delight, the corresponding lata-stage trifluoromethylation products (**5**, **7**, **14**) were afforded in a good yield in a shorter time (0.2 mmol/4 h).

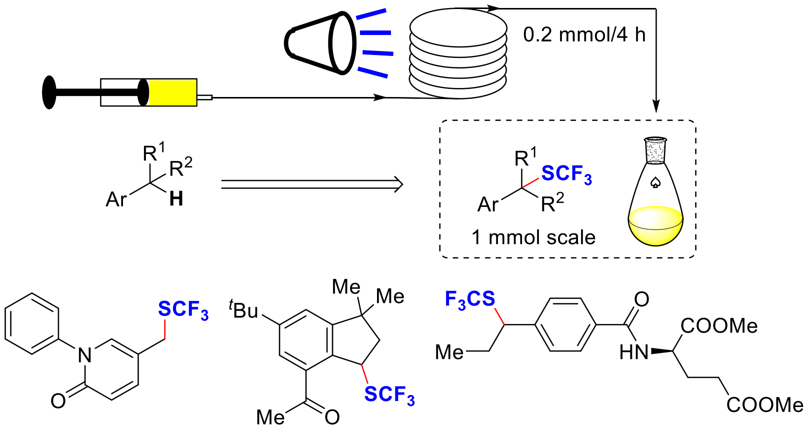

4, 65% **7**, 53% **17**, 41%

Supplementary Figure 18. Continuous-flow synthesis.

Complex molecule (1.0 mmol), PhthSCF$_3$ (321 mg, 1.3 mmol), 4CzIPN (16 mg, 0.02 mmol), and K$_2$CO$_3$ (27.6 mg, 0.2 mmol) were placed in a sample bottle (20 mL). After placed in the glove box, anhydrous MeCN (20.0 mL) was added and the yellow mixture was then transferred into the syringe (20 mL) in the glove box. Next, the reaction mixture was flowing under the irradiation of three 45 W blue LEDs (distance app. 4.0 cm) with a small fan at room temperature in the mode of perfusion/extraction at the speed of 2.0 mL/h. When the reaction finished, the mixture was quenched with water and extracted with ethyl acetate (3 x 10 mL). The organic layers were combined and concentrated under vacuo. The product was purified by flash column chromatography on silica gel (petroleum ether: ethyl acetate). Note that anhydrous K$_2$CO$_3$ was pulverized into powder (120 mesh) for use.

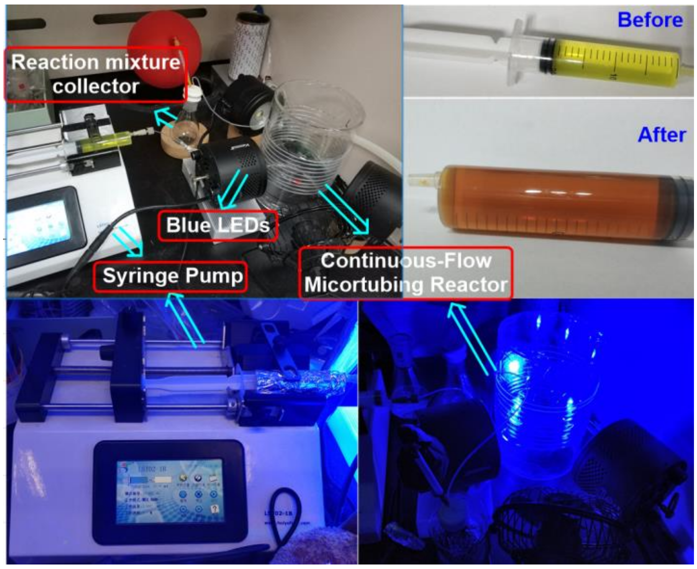

Supplementary Figure 19. Reaction set-up

Investigation of the reaction mechanism

Radical trap experiments: The reaction was completely inhibited by TEMPO, BHT, and PBN. These results indicated that the reaction probably proceeded via a free radical process.

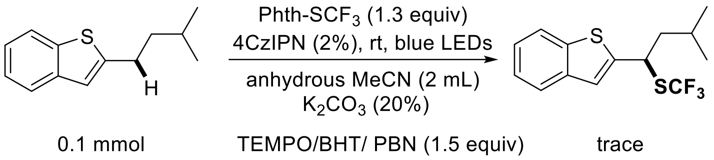

Supplementary Figure 20. Radical trap experiments.

EPR experiments

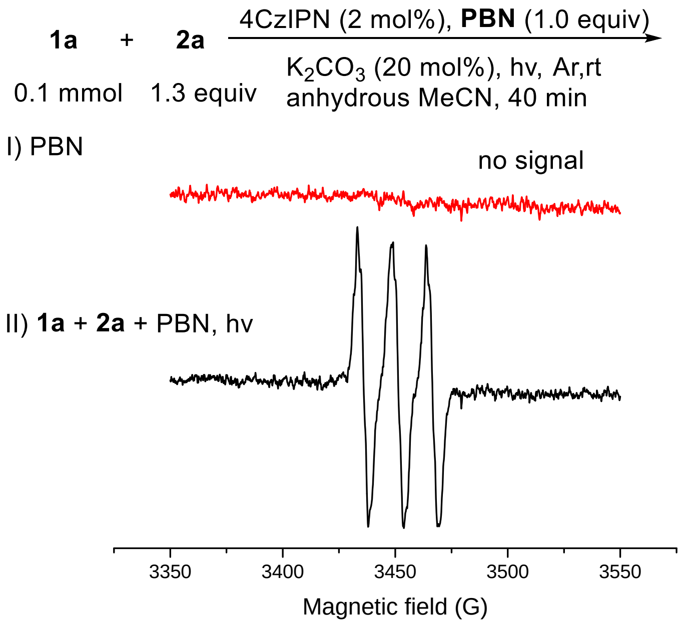

Supplementary Figure 21. EPR experiments.

To elucidate the possible reaction mechanism, electron paramagnetic resonance (EPR)

experiments with N-tert-Butyl-α-phenylnitrone (PBN) as electron-spin trapping reagent were carried out. As shown in Fig S20-II, a significant EPR signal was observed for the model reaction, indicating a possible radical pathway.

Radical clock experiment

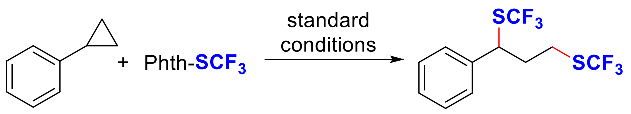

23, 26%

According to the general procedure in 0.1 mmol scale using 1.3 equiv PhthSCF$_3$ reagent with reaction time of 12 h, cyclopropylbenzene went through cycle-opening process to deliver **24** in 26% isolated yield (based on cyclopropylbenzene). The results clearly demonstrated the generation of benzylic radical.

^{1}H NMR (400 MHz, CDCl$_3$) δ 7.41 – 7.27 (m, 5H), 4.44 (dd, J = 8.9, 6.6 Hz, 1H), 2.88 (ddd, J = 13.9, 7.9, 6.0 Hz, 1H), 2.76 (dt, J = 14.3, 7.6 Hz, 1H), 2.38 (tt, J = 14.5, 6.6 Hz, 2H); ^{19}F NMR (376 MHz, CDCl$_3$) δ -39.81, -40.89; ^{13}C NMR (101 MHz, CDCl$_3$) δ 138.7, 130.8 (q, J = 309.4 Hz), 130.3 (q, J = 308.7 Hz), 129.2, 128.5, 127.3, 47.6, 36.2, 27.3 (d, J = 2.2 Hz). IR (ATR): ν = 3033, 2929, 2359, 1494, 1455, 1099, 754, 697 cm^{-1}. HRMS (EI) calcd for $C_{11}H_{10}F_6S_2$ (M^+): 320.0128, found 320.0130.

KIE experiment

Parallel competition

Following the general procedure, D$_8$-toluene (0.1 mmol), Phth-SCF$_3$ (0.1 mmol), 4CzIPN (2 mol%, 1.6 mg), K_2CO_3 (20 mol% 2.76 mg), anhydrous MeCN (1 mL) were successively added to a 10 mL transparent Schlenk tube under Ar atmosphere, equipped with a stir bar. In another reaction Schlenk tube, toluene (0.1 mmol) was used instead of D$_8$-toluene. The two reactions were stirred under blue LEDs irradiation for 30 min. Then acetophenone (0.1 mmol), used as as an internal standard, was added after the reaction, respectively. GC-MS was using the diluent mixture. The ratio of **24**:[D7]-**24** = 6.9/1 was determined by GC-MS (Method 3).

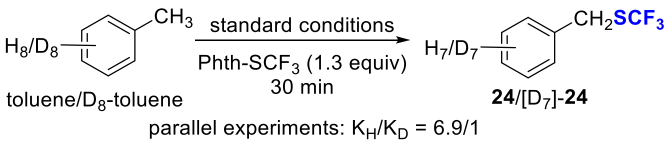

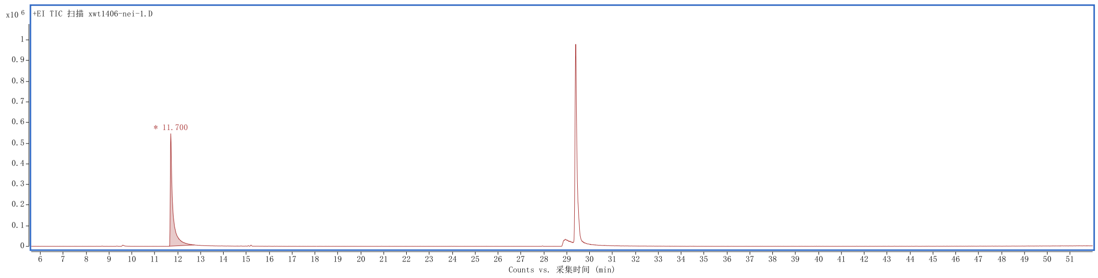

Supplementary Figure 22. TIC for D$_8$-toluene

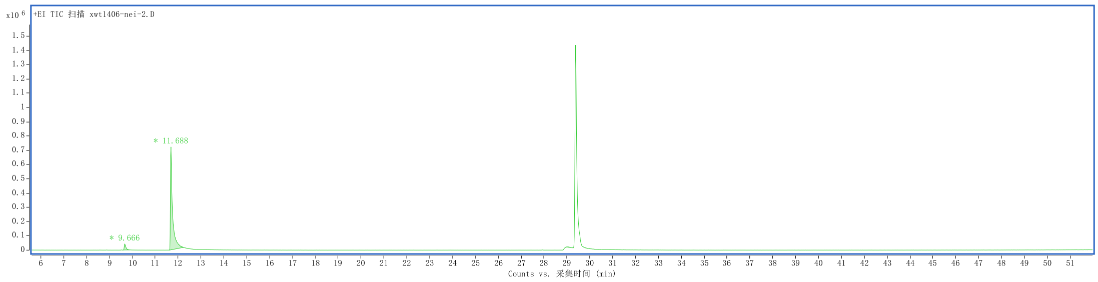

Supplementary Figure 23. TIC for toluene

Entry	RT (min)	Area	Ratio
1 (D$_8$-toluene)	9.557	26758.23	0.006303
	11.700	4245286.96	
2 (toluene)	9.666	211701.72	0.0435329
	11.688	4863024.72	

KIE = K_H/K_D = 0.0435329/0.006303 = 6.9/1

Intermolecular competition

Following the general procedure, toluene (0.1 mmol), D$_8$-toluene (0.1 mmol), Phth-SCF$_3$ (0.1 mmol), 4CzIPN (2 mol%, 1.6 mg), K_2CO_3 (20 mol% 2.76 mg), anhydrous MeCN (1 mL) were successively added to a 10 mL transparent Schlenk tube under Ar atmosphere, equipped with a stir bar. The mixture was stirred under blue LEDs irradiation for 30 min. GC-MS was using the diluent mixture. The ratio of **24**:[D7]-**24** = 24.7/1 was determined by GC-MS (Method 3).

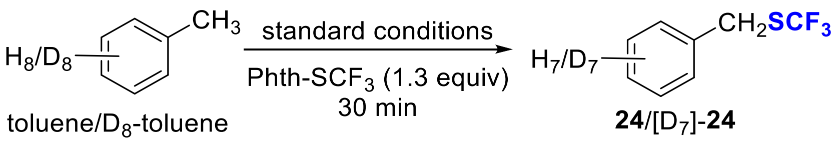

intermolecular competition: **24**/[D$_7$]-**24** = 24.7/1

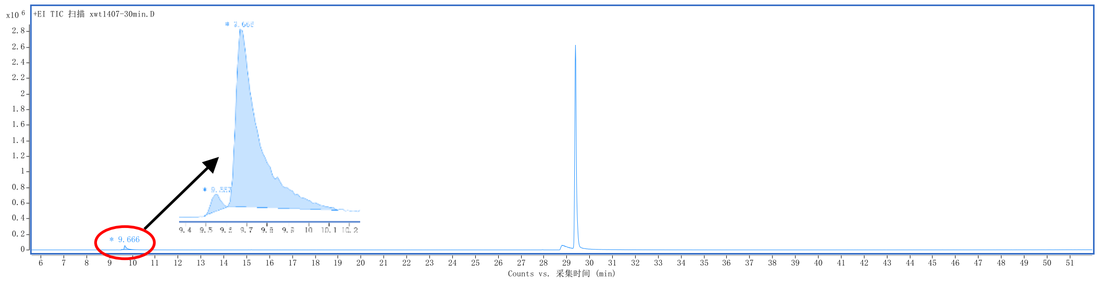

Supplementary Figure 24. TIC for intermolecular competition

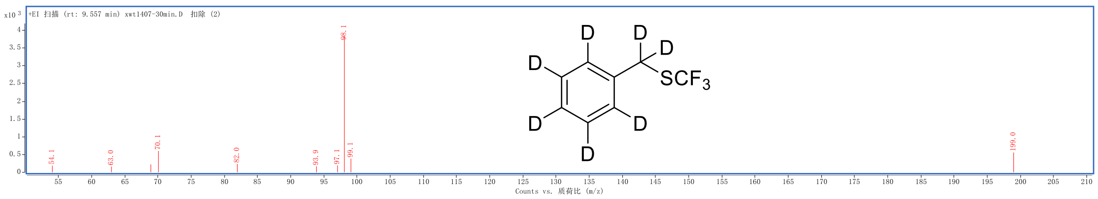

Supplementary Figure 25. TIC for D$_8$-toluene RT: 9.557 min, m/z 199.0, ((phenyl-d5)methyl-d2)(trifluoromethyl)sulfane, [D7]-**24**

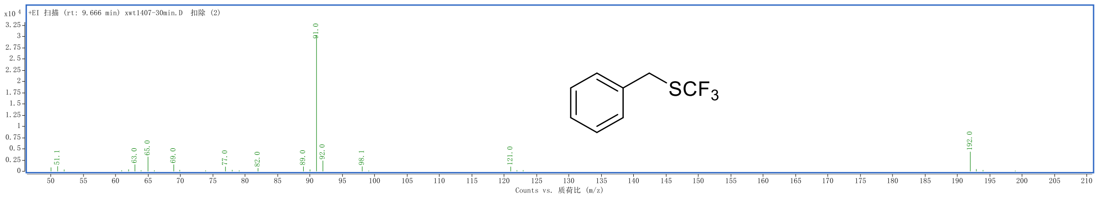

Supplementary Figure 26. TIC for D_8-toluene RT: 9.666 min, m/z 192.0, benzyl(trifluoromethyl)sulfane, **24**

Luminescence quenching experiment

The luminescence quenching experiment was taken using a F-7000 FL Spectrophotometer (Hitachi, Japan). The experiments were carried out in 5 x 10^{-7} mol/L of 4CzIPN in anhydrous degassed CH_3CN at 25 °C. The excitation wavelength was 435 nm and the emission intensity was collected at 540 nm. The concentrations of quencher (**1a**, 2-isopentylbenzo[b]thiophene) in CH_3CN were 0 mmol/L, 25 mmol/L, 41 mmol/L, 61 mmol/L. The concentrations of quencher (**2a**, Phth-SCF_3) in CH_3CN were 0 mmol/L, 25 mmol/L, 41 mmol/L, 61 mmol/L. Note that all the experiments were conducted under Argon atmosphere.

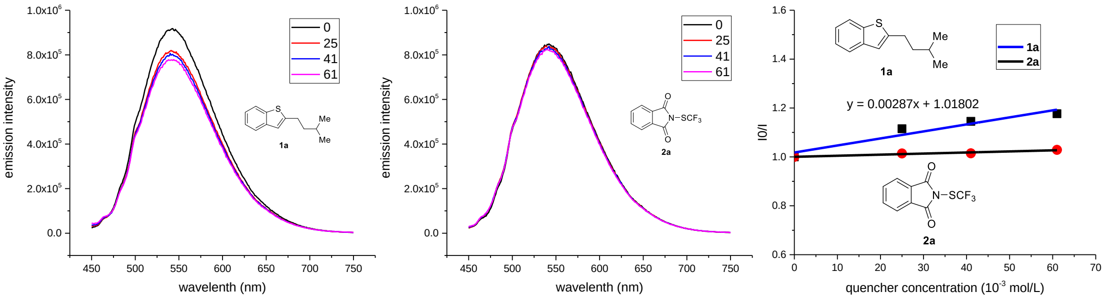

Supplementary Figure 27. The data of fluorescence quenching of 4CzIPN by **1a** and $PhthSCF_3$.

To determine whether a reductive or oxidative quenching cycle is operative in the reaction, fluorescence quenching studies were conducted. Based on the above data, photoexcited 4CzIPN* can be quenched by **1a**, involved a reductive quenching cycle.

Quantum yield measurement

The quantum yield (ф) was determined by the known ferrioxalate actinometry method. A ferrioxalate actinometry solution was prepared by following the Hammond variation of the Hatchard and Parker procedure outlined in Handbook of Photochemistry. Hence, the irradiated light intensity was estimated to 3.93 x 10^{-8} einstein S^{-1} by using $K_3[Fe(C_2O_4)_3]$ as an actinometer. For seven clean tubes, according to the general procedure, the 0.1 mmol scale model reaction solution was irradiated with two 45 W blue LEDs for specified time intervals (10 min, 20 min, 30 min, 40 min, 50 min, 60 min and 70 min). The moles of products formed were determined by GC yield with acetophenone as reference standard. The number of moles of products (y axis) per unit time is related to the number of photons (x axis, calculated from the light intensity). The slope gives the quantum yield (Φ) of the

photoreaction, 0.33 (33%). Thus, a radical chain pathway may be ruled out.

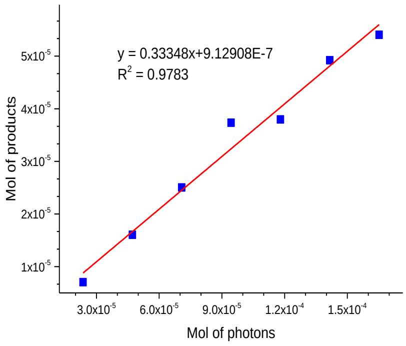

Supplementary Figure 28. The UV-Vis spectra and data of quantum yield measurement.

Cyclic Voltammetry (CV) measurements

Cyclic Voltammetry were collected using CHI660E from Shanghai Chenhua Instruments Limited (SCHI). Sample 0.001 M and tetrabutylammonium hexafluorophosphate 0.1 M in anhydrous MeCN were used for tests. Measurements were run using glassy carbon working electrode, platinum wire counter electrode, and 0.01 M $AgNO_3$ silver-silver chloride reference electrode in a scan rate of 0.1 V/s. In addition, ferrocene was used as an internal reference to SCE.

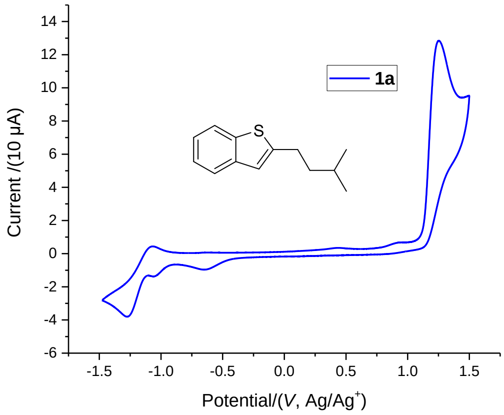

Supplementary Figure 29. The CV data of 2-isopentylbenzo[b]thiophene The oxidation potential is $E_{1/2}^{oxidation}$ = 1.368 V vs. SCE.

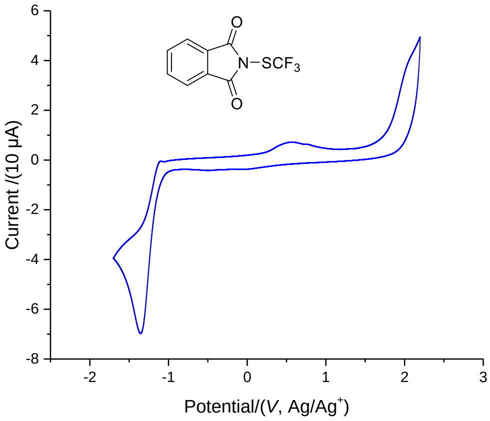

Supplementary Figure 30. The CV data of 2-((trifluoromethyl)thio)isoindoline-1,3-dione The

reductive potential is $E_{1/2}^{reduction}$ = -0.80 V vs. SCE.

In order to elucidate the possibility of SET between 4CzIPN and alkyl arenes, we again gained the CV data of 4CzIPN and toluene. To our delight, the small part overlap between the excited 4CzIPN[2] with Toluene was observed. Thus, the first sluggish oxidation of alkyl arenes is reasonable, which is consist with the luminescence experiments. $E^{1/2}(P^*/P^-) = E_{0\text{-}0} + E_{1/2}(P/P^-)$, $E_{0\text{-}0}$ = 2.53 V

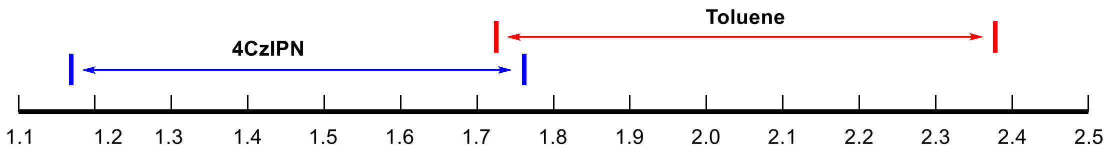

V vs SCE

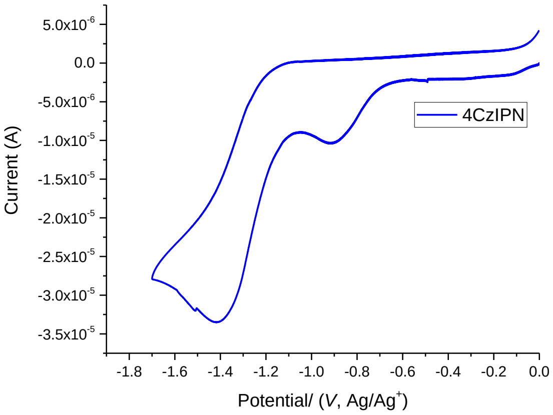

Supplementary Figure 31. In a scan rate of 0.1 V/s.

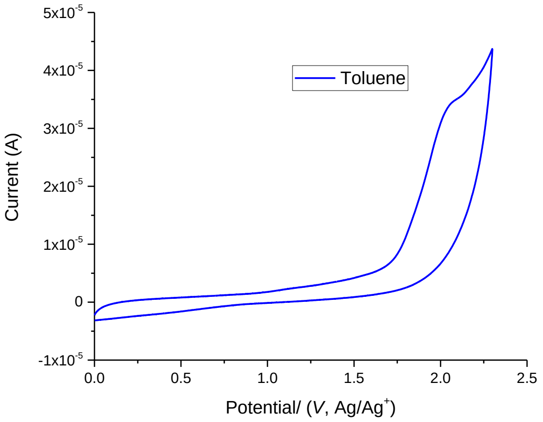

Supplementary Figure 32. In a scan rate of 0.02 V/s.

UV-Vis absorption study

All the UV spectra were measured in MeCN (I-V).

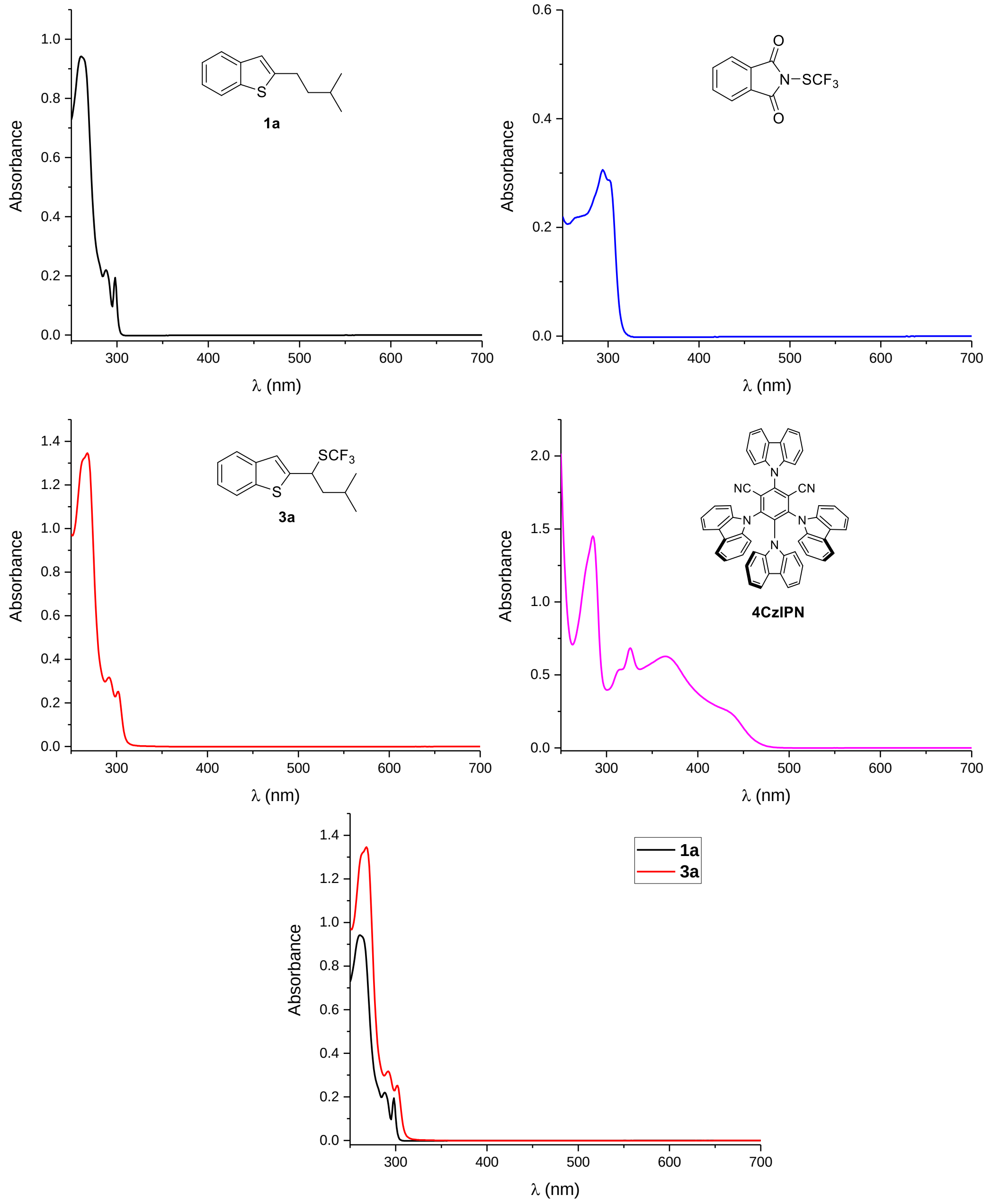

Supplementary Figure 33. The UV spectra (I-V)

It is also interesting to find that the introduction of SCF_3 into **1a** could make the the max UV-Vis absorption peak red-shift (V). It is clear to see that photocatalyst 4CzIPN has a visible light absorption range from 400-470 nm (our blue LEDs, IV). All the other compounds (**1a**, Phth-SCF_3, **3a**) don't have obvious absorption in visible-light region (>400 nm).

GC-MS Method

Method 1: Oven Method, Initial temperature: 50.0 ℃, Initial hold time: 1.00 min, Number of ramps: 1, Ramp 01 rate: 20.0 ℃/min, Ramp 01 final temperature: 250.0 ℃, Ramp 01 hold time: 5.00 min, Front Method, S/SL mode: Split, Temperature: 250 ℃, Split flow enable: On, Split flow: 60 mL/min, Purge flow: 3.0 mL/min, Constant septum purge: On, Carrier mode: Constant Flow, Carrier flow: 1.200 mL/min. Mass Method, MS transfer line temperature: 250 ℃, Ion source temperature: 230 ℃,

Ionization mode: EI, Start time: 4.0 minute, Start mass: 50 amu, End mass: 500 amu, Scan time: 0.2 sec;

Method 2: Oven Method , Initial temperature: 50.0 ℃, Initial hold time: 1.00 min, Number of ramps: 1, Ramp 01 rate: 10.0 ℃/min, Ramp 01 final temperature: 200.0 ℃, Ramp 01 hold time: 4.00 min, Front Method, S/SL mode: Split, Temperature: 200 ℃, Split flow enable: On, Split flow: 60 mL/min, Purge flow: 3.0 mL/min, Constant septum purge: On, Carrier mode: Constant Flow, Carrier flow: 1.200 mL/min. Mass Method, MS transfer line temperature: 200 ℃, Ion source temperature: 230 ℃, Ionization mode: EI, Start time: 4.0 minute, Start mass: 50 amu, End mass: 500 amu, Scan time: 0.2 sec;

Method 3: Oven Method , Initial temperature: 50.0 ℃, Initial hold time: 1.00 min, Number of ramps: 2, Ramp 01 rate: 5.0 ℃/min, Ramp 01 final temperature: 200.0 ℃, Ramp 01 hold time: 5.00 min; Ramp 02 rate: 20 ℃/min, Ramp 02 final temperature: 250.0 ℃, Ramp 01 hold time: 11.50 min, Front Method, S/SL mode: Split, Temperature: 250 ℃, Split flow enable: On, Split flow: 60 mL/min, Purge flow: 3.0 mL/min, Constant septum purge: On, Carrier mode: Constant Flow, Carrier flow: 1.200 mL/min. Mass Method, MS transfer line temperature: 250 ℃, Ion source temperature: 230 ℃, Ionization mode: EI, Start time: 5.0 minute, Start mass: 50 amu, End mass: 500 amu, Scan time: 0.2 sec;

Preparation of substrates

Procedure A:

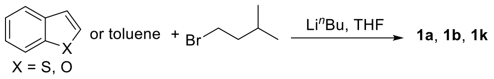

The following reactants were prepared from the corresponding arenes according to procedure A.[3]

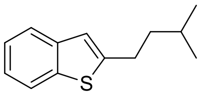

2-isopentylbenzo[b]thiophene, 20 mmol scale, colorless oil, 2.4 g, 59%, Rf = 0.9 (petroleum ether); ^{1}H NMR (500 MHz, $CDCl_3$) δ 7.74 (d, *J* = 7.9 Hz, 1H), 7.64 (d, *J* = 7.7 Hz, 1H), 7.30 – 7.26 (m, 1H), 7.22 (td, *J* = 7.7, 7.2, 1.2 Hz, 1H), 6.98 (s, 1H), 2.92 – 2.87 (m, 2H), 1.69 – 1.60 (m, 3H), 0.95 (d, *J* = 6.3 Hz, 6H); ^{13}C NMR (126 MHz, $CDCl_3$) δ 147.0, 140.2, 139.2, 124.0, 123.3, 122.6, 122.1, 120.3, 40.2, 28.7, 27.5, 22.4.

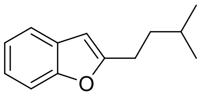

2-isopentylbenzofuran, 20 mmol scale, colorless oil, 1.3 g, 35%, Rf = 0.9 (petroleum ether); ^{1}H NMR (400 MHz, $CDCl_3$) δ 7.49 – 7.45 (m, 1H), 7.40 (dt, *J* = 7.7, 1.1 Hz, 1H), 7.22 – 7.13 (m, 2H), 6.36 (d, *J* = 1.0 Hz, 1H), 2.81 – 2.73 (m, 2H), 1.64 (ddt, *J* = 9.8, 5.3, 3.1 Hz, 3H), 0.95 (d, *J* = 6.2 Hz, 6H); ^{13}C NMR (101 MHz, $CDCl_3$) δ 159.9, 154.6, 129.0, 123.0, 122.3, 120.1, 110.7, 101.6, 36.6, 27.6, 26.4, 22.4.

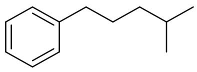

(4-methylpentyl)benzene, 10 mmol scale, colorless oil, 0.8 g, 49%, Rf = 0.9 (petroleum ether); ^{1}H NMR (400 MHz, $CDCl_3$) δ 7.27 (t, *J* = 7.5 Hz, 2H), 7.17 (d, *J* = 7.4 Hz, 3H), 2.61 – 2.55 (m, 2H), 1.66 – 1.50 (m, 3H), 1.27 – 1.18 (m, 2H), 0.87 (d, *J* = 6.6 Hz, 6H); ^{13}C NMR (101 MHz, $CDCl_3$) δ 143.0, 128.4, 128.2, 125.6, 38.7, 36.3, 29.4, 27.9, 22.6.

Procedure B:

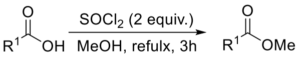

The following reactants were prepared from the corresponding carboxylic acids according to procedure B. To a stirred solution of the corresponding carboxylic acid in MeOH (30 mL), $SOCl_2$ (0.75 mL, 10 mmol) was added dropwise. Then, the reaction was stirred with a reflux condenser at 90 ^{o}C for 3 h. After the reaction cooling to the room temperature, the reaction mixture was concentrated in vacuo to remove CH_3OH and thionylchloride to afford the crude ester, which was next purified by column chromatography for use.

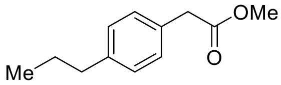

methyl 2-(4-propylphenyl)acetate, 5 mmol scale, colorless oil, 0.722 g, 75%, Rf = 0.5 (petroleum ether/ethylacetate 20:1), ^{1}H NMR (400 MHz, $CDCl_3$) δ 7.18 (d, *J* = 8.1 Hz, 2H), 7.12 (d, *J* = 8.1 Hz, 2H), 3.67 (s, 3H), 3.58 (s, 2H), 2.56 (dd, *J* = 8.5, 6.8 Hz, 2H), 1.68 – 1.55 (m, 2H), 0.93 (t, *J* = 7.3 Hz, 3H); ^{13}C NMR (101 MHz, $CDCl_3$) δ 172.1, 141.4, 131.1, 129.0, 128.6, 51.9, 40.7, 37.6, 24.5, 13.8.

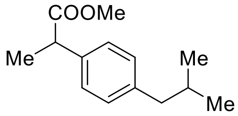

methyl 2-(4-isobutylphenyl)propanoate, 5 mmol scale, colorless oil, 0.95 g, 80%, Rf = 0.5 (petroleum ether/ethylacetate 20:1); ^{1}H NMR (500 MHz, $CDCl_3$) δ 7.19 (d, *J* = 8.1 Hz, 2H), 7.09 (d, *J* = 8.1 Hz, 2H), 3.65 (s, 3H), 2.44 (d, *J* = 7.2 Hz, 2H), 1.84 (m, 1H), 1.48 (d, *J* = 7.1 Hz, 3H), 0.89 (d, *J* = 6.7 Hz, 6H); ^{13}C NMR (126 MHz, $CDCl_3$) δ 175.2, 140.5, 137.7, 129.3, 127.1, 51.9, 45.0, 45.0, 30.2, 22.4, 18.6.

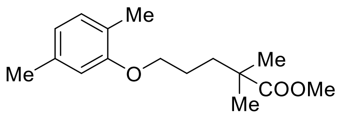

methyl 5-(2,5-dimethylphenoxy)-2,2-dimethylpentanoate, 10 mmol scale, colorless oil, 2.6 g, 98%, Rf = 0.5 (petroleum ether/ethylacetate 20:1), ^{1}H NMR (400 MHz, $CDCl_3$) δ 6.98 (d, *J* = 7.5 Hz, 1H),

6.64 (d, J = 7.5 Hz, 1H), 6.59 (s, 1H), 3.89 (t, J = 2.3 Hz, 2H), 3.65 (s, 3H), 2.29 (s, 3H), 2.17 (s, 3H), 1.71 (d, J = 2.9 Hz, 4H), 1.21 (s, 6H); ^{13}C NMR (101 MHz, $CDCl_3$) δ 178.1, 156.8, 136.3, 130.2, 123.4, 120.6, 111.8, 67.7, 51.6, 42.0, 37.0, 25.1, 25.1, 21.3, 15.7.

Procedure C:

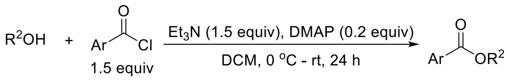

The following reactants were prepared from the corresponding alcohols and 4-ethylbenzoyl chloride according to procedure C.

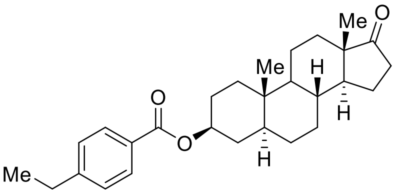

(3S,5S,8R,10S,13S,14S)-10,13-dimethyl-17-oxohexadecahydro-1H-cyclopenta[a]phenanthren-3-yl 4-ethylbenzoate, 5 mmol scale, white solid, 1.22 g, 58%, Rf = 0.5 (petroleum ether/ethylacetate 20:1); ^{1}H NMR (400 MHz, $CDCl_3$) δ 7.95 (d, J = 8.2 Hz, 2H), 7.25 (d, J = 8.2 Hz, 2H), 4.93 (tt, J = 11.3, 4.9 Hz, 1H), 2.69 (q, J = 7.7 Hz, 2H), 2.44 (dd, J = 19.3, 8.5 Hz, 1H), 2.17 – 2.01 (m, 1H), 1.93 (ddd, J = 14.5, 8.9, 6.1 Hz, 2H), 1.85 – 1.72 (m, 4H), 1.70 – 1.21 (m, 14H), 1.06 (dtd, J = 35.7, 12.9, 12.2, 4.5 Hz, 2H), 0.89 (s, 3H), 0.86 (s, 3H), 0.75 (ddd, J = 11.9, 10.2, 3.9 Hz, 1H); ^{13}C NMR (101 MHz, $CDCl_3$) δ 221.3, 166.2, 149.5, 130.3, 129.7, 128.3, 128.0, 127.8, 73.9, 54.3, 51.4, 47.8, 44.7, 36.8, 35.9, 35.7, 35.0, 34.1, 31.5, 30.8, 28.9, 28.3, 27.5, 21.8, 20.5, 15.3, 13.8, 12.3.

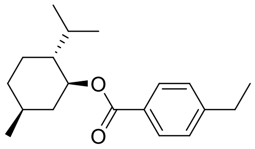

(1S,2R,5S)-2-isopropyl-5-methylcyclohexyl 4-ethylbenzoate, 5 mmol scale, white solid, 0.498 g, 34%, Rf = 0.3 (petroleum ether/ethylacetate 50:1); ^{1}H NMR (400 MHz, $CDCl_3$) δ 7.97 (d, J = 8.3 Hz, 2H), 7.25 (d, J = 8.3 Hz, 2H), 4.93 (td, J = 10.9, 4.4 Hz, 1H), 2.69 (q, J = 7.6 Hz, 2H), 2.17 – 2.08 (m, 1H), 1.97 (pd, J = 7.0, 2.8 Hz, 1H), 1.78 – 1.68 (m, 2H), 1.55 (dddd, J = 14.9, 13.2, 7.5, 3.2 Hz, 2H), 1.24 (t, J = 7.6 Hz, 3H), 1.19 – 1.02 (m, 2H), 0.92 (dd, J = 6.8, 3.9 Hz, 7H), 0.79 (d, J = 7.0 Hz, 3H); ^{13}C NMR (101 MHz, $CDCl_3$) δ 166.0, 149.4, 129.6, 128.3, 127.8, 74.5, 47.3, 41.0, 34.3, 31.4, 28.9, 26.5, 23.6, 22.0, 20.7, 16.5, 15.3.

Procedure D:

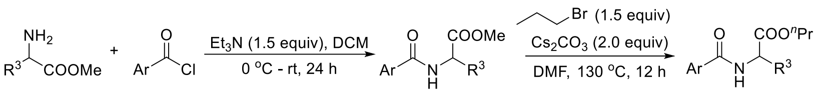

The following reactants were prepared from the corresponding amino acid methyl esters and

benzoyl chlorides according to procedure D.

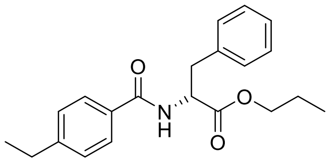

propyl (4-ethylbenzoyl)-D-phenylalaninate, 3.2 mmol scale, colorless oil, 0.120 g, 12%, Rf = 0.6 (petroleum ether/ethylacetate 5:1); ^{1}H NMR (400 MHz, $CDCl_3$) δ 7.65 (d, *J* = 8.3 Hz, 2H), 7.31 – 7.19 (m, 5H), 7.18 – 7.10 (m, 2H), 6.66 (d, *J* = 7.6 Hz, 1H), 5.07 (dt, *J* = 7.6, 5.7 Hz, 1H), 4.15 – 4.03 (m, 2H), 3.33 – 3.16 (m, 2H), 2.67 (q, *J* = 7.6 Hz, 2H), 1.65 (h, *J* = 7.1 Hz, 2H), 1.23 (t, *J* = 7.6 Hz, 3H), 0.92 (t, *J* = 7.4 Hz, 3H); ^{13}C NMR (101 MHz, $CDCl_3$) δ 171.7, 166.7, 148.3, 135.9, 131.3, 129.3, 128.4, 128.0, 127.0, 127.0, 67.1, 53.5, 37.9, 28.7, 21.8, 15.2, 10.3.

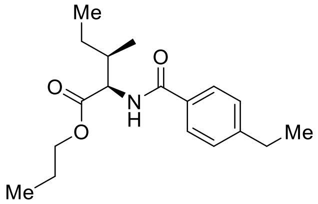

propyl (4-ethylbenzoyl)-D-isoleucinate, 4.0 mmol scale, colorless oil, 0.122 g, 10%, Rf = 0.6 (petroleum ether/ethylacetate 5:1); ^{1}H NMR (400 MHz, $CDCl_3$) δ 7.73 (d, *J* = 8.2 Hz, 2H), 7.26 (d, *J* = 8.2 Hz, 2H), 6.70 (d, *J* = 8.4 Hz, 1H), 4.82 (dd, *J* = 8.5, 4.8 Hz, 1H), 4.19 – 4.07 (m, 2H), 2.69 (q, *J* = 7.6 Hz, 2H), 2.02 (ddd, *J* = 9.1, 4.6, 2.2 Hz, 1H), 1.70 (h, *J* = 7.1 Hz, 2H), 1.53 (dtd, *J* = 14.6, 7.3, 4.7 Hz, 1H), 1.25 (t, *J* = 7.6 Hz, 4H), 0.97 (t, *J* = 7.4 Hz, 9H).

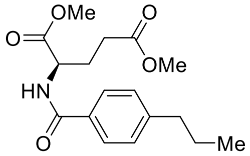

dimethyl (4-propylbenzoyl)-D-glutamate, 3.0 mmol scale, white solid, 0.7 g, 73%, Rf = 0.3 (petroleum ether/ethylacetate 3:1); ^{1}H NMR (400 MHz, $CDCl_3$) δ 7.74 (d, *J* = 8.2 Hz, 2H), 7.22 (d, *J* = 8.2 Hz, 3H), 4.81 (td, *J* = 8.0, 4.8 Hz, 1H), 3.75 (d, *J* = 4.2 Hz, 3H), 3.63 (d, *J* = 3.7 Hz, 3H), 2.61 (dd, *J* = 8.8, 6.4 Hz, 2H), 2.56 – 2.39 (m, 2H), 2.31 (dtd, *J* = 14.5, 7.3, 4.9 Hz, 1H), 2.13 (dq, *J* = 14.5, 7.6 Hz, 1H), 1.64 (h, *J* = 7.3 Hz, 2H), 0.93 (t, *J* = 7.3 Hz, 3H); ^{13}C NMR (101 MHz, $CDCl_3$) δ 173.4, 172.3, 167.0, 146.7, 130.8, 128.4, 127.0, 52.3, 52.0, 51.7, 37.7, 30.1, 27.0, 24.1, 13.5.

Procedure E

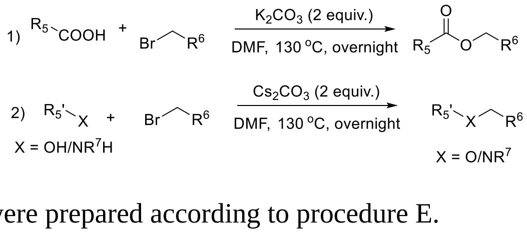

The following reactants were prepared according to procedure E.

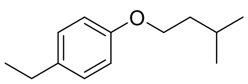

1-ethyl-4-(isopentyloxy)benzene, 5 mmol scale, colorless oil, 0.85 g, 88%, Rf = 0.7 (petroleum ether/ethylacetate 20:1); ^{1}H NMR (400 MHz, $CDCl_3$) δ 7.09 (d, *J* = 8.6 Hz, 2H), 6.81 (d, *J* = 8.6 Hz, 2H), 3.95 (t, *J* = 6.7 Hz, 2H), 2.58 (q, *J* = 7.6 Hz, 2H), 1.83 (m, 1H), 1.66 (q, *J* = 6.7 Hz, 2H), 1.20 (t, *J* = 7.6 Hz, 3H), 0.95 (d, *J* = 6.6 Hz, 6H); ^{13}C NMR (101 MHz, $CDCl_3$) δ 157.2, 136.2, 128.7, 114.4, 66.4, 38.2, 28.0, 25.1, 22.6, 15.9.

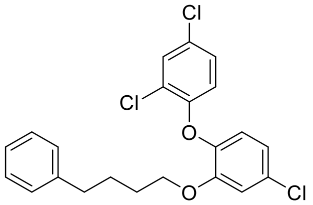

2,4-dichloro-1-(4-chloro-2-(4-phenylbutoxy)phenoxy)benzene, 5 mmol scale, colorless oil, 1.3 g, 62%, Rf = 0.5 (petroleum ether/ethylacetate 20:1); ^{1}H NMR (500 MHz, $CDCl_3$) δ 7.33 (d, *J* = 2.5 Hz, 1H), 7.23 (t, *J* = 7.5 Hz, 2H), 7.13 (t, *J* = 7.4 Hz, 1H), 7.09 – 7.03 (m, 2H), 6.99 (dd, *J* = 8.8, 2.5 Hz, 1H), 6.94 – 6.84 (m, 3H), 6.55 (d, *J* = 8.8 Hz, 1H), 3.82 (t, *J* = 6.0 Hz, 2H), 2.50 (t, *J* = 7.6 Hz, 2H), 1.59 (dq, *J* = 12.2, 6.1 Hz, 2H), 1.49 (dt, *J* = 14.8, 7.1 Hz, 2H); ^{13}C NMR (126 MHz, $CDCl_3$) δ 152.5, 150.9, 142.6, 141.8, 130.5, 129.9, 128.2, 128.2, 127.4, 127.3, 125.6, 124.0, 122.3, 120.7, 117.3, 114.5, 68.6, 35.2, 28.4, 27.3.

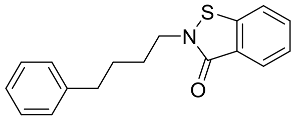

2-(4-phenylbutyl)benzo[d]isothiazol-3(2H)-one, 5 mmol scale, colorless oil, 0.716 g, 51%, Rf = 0.5 (petroleum ether/ethylacetate 50:1); ^{1}H NMR (400 MHz, $CDCl_3$) δ 7.88 (d, *J* = 8.0 Hz, 1H), 7.71 (d, *J* = 8.2 Hz, 1H), 7.46 (ddd, *J* = 8.1, 6.9, 1.2 Hz, 1H), 7.33 (t, *J* = 7.5 Hz, 1H), 7.27 (t, *J* = 7.5 Hz, 2H), 7.22 – 7.13 (m, 3H), 4.54 (t, *J* = 6.3 Hz, 2H), 2.70 (t, *J* = 7.5 Hz, 2H), 1.95 – 1.78 (m, 4H); ^{13}C NMR (101 MHz, $CDCl_3$) δ 163.1, 151.5, 142.1, 128.5, 128.4, 128.3, 125.7, 125.4, 124.2, 123.0, 120.0, 68.5, 35.5, 28.6, 27.8.

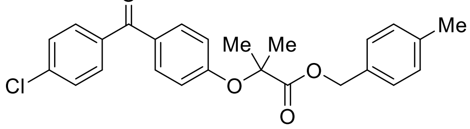

4-methylbenzyl 2-(4-(4-chlorobenzoyl)phenoxy)-2-methylpropanoate, 5 mmol scale, white solid, 1.7 g, 80%, Rf = 0.3 (petroleum ether/ethylacetate 20:1); ^{1}H NMR (500 MHz, $CDCl_3$) δ 7.69 (d, *J* = 8.5 Hz, 2H), 7.63 (d, *J* = 8.8 Hz, 2H), 7.45 (d, *J* = 8.4 Hz, 2H), 7.14 (d, *J* = 8.1 Hz, 2H), 7.10 (d, *J* = 7.8 Hz, 2H), 6.76 (d, *J* = 8.9 Hz, 2H), 5.16 (s, 2H), 2.30 (s, 3H), 1.66 (s, 6H); ^{13}C NMR (126 MHz, $CDCl_3$) δ 194.2, 173.4, 159.5, 138.4, 138.3, 136.4, 132.1, 131.9, 131.1, 130.2, 129.2, 128.6,

128.5, 117.2, 79.4, 67.3, 25.4, 21.2.

Procedure F:

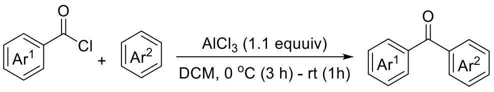

The following reactants were prepared from the corresponding arenes and benzoyl chlorides according to procedure F.

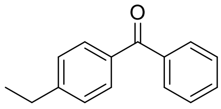

(4-ethylphenyl)(phenyl)methanone, 10 mmol scale, colorless oil, 0.512 g, 25%, Rf = 0.5 (petroleum ether/ethylacetate 20:1); ^{1}H NMR (400 MHz, $CDCl_3$) δ 7.83 – 7.72 (m, 4H), 7.61 – 7.54 (m, 1H), 7.48 (t, *J* = 7.5 Hz, 2H), 7.31 (d, *J* = 8.3 Hz, 2H), 2.74 (q, *J* = 7.6 Hz, 2H), 1.29 (t, *J* = 7.6 Hz, 3H); ^{13}C NMR (101 MHz, $CDCl_3$) δ 196.5, 149.4, 137.9, 135.1, 132.1, 130.4, 129.9, 128.2, 127.8, 29.0, 15.3.

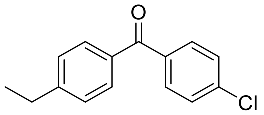

(4-chlorophenyl)(4-ethylphenyl)methanone, 10 mmol scale, colorless oil, 1.5 g, 61%, Rf = 0.4 (petroleum ether/ethylacetate 50:1); ^{1}H NMR (400 MHz, $CDCl_3$) δ 7.73 (dd, *J* = 11.0, 8.4 Hz, 4H), 7.45 (d, *J* = 8.5 Hz, 2H), 7.31 (d, *J* = 8.2 Hz, 2H), 2.74 (q, *J* = 7.6 Hz, 2H), 1.28 (t, *J* = 7.6 Hz, 3H); ^{13}C NMR (101 MHz, $CDCl_3$) δ 195.2, 149.7, 138.6, 136.2, 134.7, 131.4, 130.3, 128.5, 127.9, 29.0, 15.2.

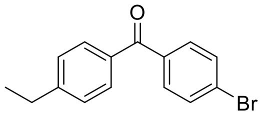

(4-bromophenyl)(4-ethylphenyl)methanone, 10 mmol scale, colorless oil, 1.5 g, 61%, Rf = 0.4 (petroleum ether/ethylacetate 50:1); ^{1}H NMR (400 MHz, $CDCl_3$) δ 7.72 (d, *J* = 8.2 Hz, 2H), 7.67 (d, *J* = 8.6 Hz, 2H), 7.62 (d, *J* = 8.6 Hz, 2H), 7.31 (d, *J* = 8.2 Hz, 2H), 2.74 (q, *J* = 7.6 Hz, 2H), 1.28 (t, *J* = 7.6 Hz, 3H); ^{13}C NMR (101 MHz, $CDCl_3$) δ 195.4, 149.8, 136.7, 134.7, 131.5, 131.5, 130.3, 127.9, 127.2, 29.0, 15.3.

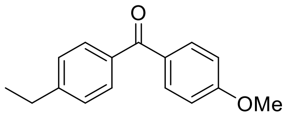

(4-ethylphenyl)(4-methoxyphenyl)methanone, 10 mmol scale, colorless oil, 1.6 g, 66%, Rf = 0.5 (petroleum ether/ethylacetate 20:1); ^{1}H NMR (400 MHz, $CDCl_3$) δ 7.82 (d, *J* = 8.9 Hz, 2H), 7.70 (d, *J* = 8.2 Hz, 2H), 7.29 (d, *J* = 8.2 Hz, 2H), 6.95 (d, *J* = 8.9 Hz, 2H), 3.87 (s, 3H), 2.73 (q, *J* = 7.6

Hz, 2H), 1.28 (t, *J* = 7.6 Hz, 3H); ^{13}C NMR (101 MHz, $CDCl_3$) δ 195.3, 163.0, 148.8, 135.7, 132.4, 130.4, 130.0, 127.6, 113.4, 55.4, 28.9, 15.3.

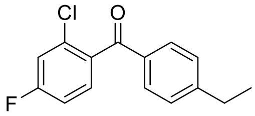

(2-chloro-4-fluorophenyl)(4-ethylphenyl)methanone, 5 mmol scale, colorless oil, 0.884 g, 67%, Rf = 0.5 (petroleum ether/ethylacetate 20:1); ^{1}H NMR (400 MHz, $CDCl_3$) δ 7.72 (d, *J* = 8.3 Hz, 2H), 7.37 (dd, *J* = 8.5, 6.0 Hz, 1H), 7.29 (d, *J* = 8.4 Hz, 2H), 7.21 (dd, *J* = 8.6, 2.4 Hz, 1H), 7.08 (td, *J* = 8.2, 2.4 Hz, 1H), 2.72 (q, *J* = 7.6 Hz, 2H), 1.27 (t, *J* = 7.6 Hz, 3H); ^{19}F NMR (376 MHz, $CDCl_3$) δ -108.21 (td, *J* = 8.2, 6.1 Hz); ^{13}C NMR (101 MHz, $CDCl_3$) δ 193.9, 163.1 (d, *J* = 253.2 Hz), 151.0, 135.0 (d, *J* = 3.6 Hz), 134.1, 132.8 (d, *J* = 10.4 Hz), 130.7 (d, *J* = 9.3 Hz), 130.3, 128.2, 117.5 (d, *J* = 24.8 Hz), 114.1 (d, *J* = 21.6 Hz), 29.0, 15.1.

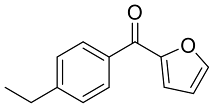

(4-ethylphenyl)(furan-2-yl)methanone, 10 mmol scale, colorless oil, 1.440 g, 72%, Rf = 0.5 (petroleum ether/ethylacetate 20:1);^{1}H NMR (400 MHz, $CDCl_3$) δ 7.91 (d, *J* = 8.2 Hz, 2H), 7.67 (dd, *J* = 1.7, 0.8 Hz, 1H), 7.30 (d, *J* = 8.1 Hz, 2H), 7.21 (dd, *J* = 3.6, 0.8 Hz, 1H), 6.56 (dd, *J* = 3.6, 1.7 Hz, 1H), 2.70 (q, *J* = 7.6 Hz, 2H), 1.26 (t, *J* = 7.6 Hz, 3H); ^{13}C NMR (101 MHz, $CDCl_3$) δ 182.0, 152.2, 149.3, 146.7, 134.6, 129.3, 127.7, 120.0, 111.9, 28.7, 15.0.

Others[4,5]:

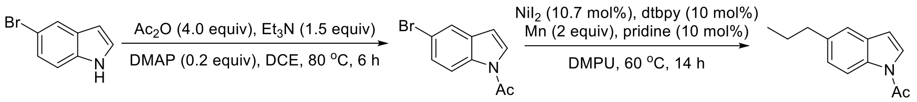

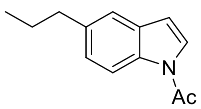

1-(5-propyl-1H-indol-1-yl)ethan-1-one, 10 mmol scale, white solid, 1.0 g, 51%, Rf = 0.3 (petroleum ether/ethylacetate 10:1); ^{1}H NMR (400 MHz, $CDCl_3$) δ 8.31 (d, *J* = 8.5 Hz, 1H), 7.37 (d, *J* = 3.8 Hz, 1H), 7.35 (s, 1H), 7.17 (dd, *J* = 8.6, 1.7 Hz, 1H), 6.57 (dd, *J* = 3.8, 0.7 Hz, 1H), 2.67 (dd, *J* = 8.4, 6.8 Hz, 2H), 2.61 (s, 3H), 1.68 (m, *J* = 7.4 Hz, 2H), 0.95 (t, *J* = 7.3 Hz, 3H); ^{13}C NMR (101 MHz, $CDCl_3$) δ 168.4, 138.2, 133.9, 130.6, 125.9, 125.2, 120.2, 116.1, 109.1, 38.0, 25.0, 23.9, 13.8.

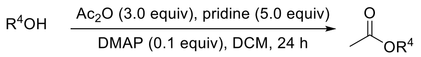

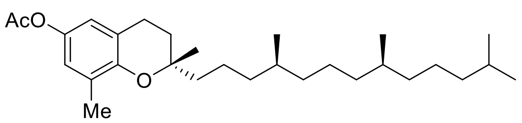

(S)-2,8-dimethyl-2-((4S,8S)-4,8,12-trimethyltridecyl)chroman-6-yl acetate, 2.5 mmol scale, light yellow oil, 0.772 g, 69%, Rf = 0.5 (petroleum ether/ethylacetate 10:1); ^{1}H NMR (500 MHz, $CDCl_3$) δ 6.66 (d, *J* = 2.8 Hz, 1H), 6.60 (d, *J* = 2.8 Hz, 1H), 2.78 – 2.65 (m, 2H), 2.23 (s, 3H), 2.13 (s, 3H), 1.74 (ddt, *J* = 38.4, 13.3, 6.9 Hz, 2H), 1.60 – 1.50 (m, 3H), 1.48 – 1.20 (m, 15H), 1.17 – 1.02 (m, 6H), 0.89 – 0.82 (m, 12H); ^{13}C NMR (126 MHz, $CDCl_3$) δ 170.1, 149.7, 142.4, 127.2, 121.1, 120.8, 119.0, 76.0, 40.1, 39.3, 37.4, 37.4, 37.2, 32.7, 32.6, 30.9, 27.9, 24.8, 24.4, 24.2, 22.7, 22.6, 22.4, 21.0, 20.9, 19.7, 19.6, 16.1.

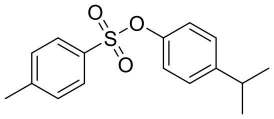

4-isopropylphenyl 4-methylbenzenesulfonate, 10 mmol scale, colorless oil, 2.1178 g, 73%, Rf = 0.7 (petroleum ether/ethylacetate 10:1); ^{1}H NMR (400 MHz, $CDCl_3$) δ 7.71 (d, *J* = 8.3 Hz, 2H), 7.30 (d, *J* = 7.9 Hz, 2H), 7.12 (d, *J* = 8.5 Hz, 2H), 6.88 (d, *J* = 8.7 Hz, 2H), 2.86 (hept, *J* = 6.9 Hz, 1H), 2.44 (s, 3H), 1.20 (d, *J* = 6.9 Hz, 6H); ^{13}C NMR (101 MHz, $CDCl_3$) δ 147.7, 147.5, 145.2, 132.6, 129.7, 128.5, 127.5, 122.0, 33.6, 23.9, 21.7. (Ref. *Synthesis* **2015**, *47*, 2578.)

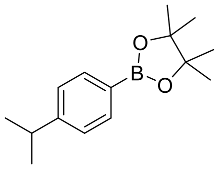

2-(4-isopropylphenyl)-4,4,5,5-tetramethyl-1,3,2-dioxaborolane, 5 mmol scale, white solid, 2.27 g, 92%, Rf = 0.4 (petroleum ether/ethylacetate 20:1); ^{1}H NMR (400 MHz, $CDCl_3$) δ 7.75 (d, *J* = 7.9 Hz, 2H), 7.24 (d, *J* = 8.0 Hz, 2H), 2.91 (hept, *J* = 6.8 Hz, 1H), 1.33 (s, 12H), 1.25 (d, *J* = 6.9 Hz, 6H); ^{13}C NMR (101 MHz, $CDCl_3$) δ 152.3, 134.9, 125.9, 83.6, 34.3, 24.8, 23.8.

NMR spectra of products

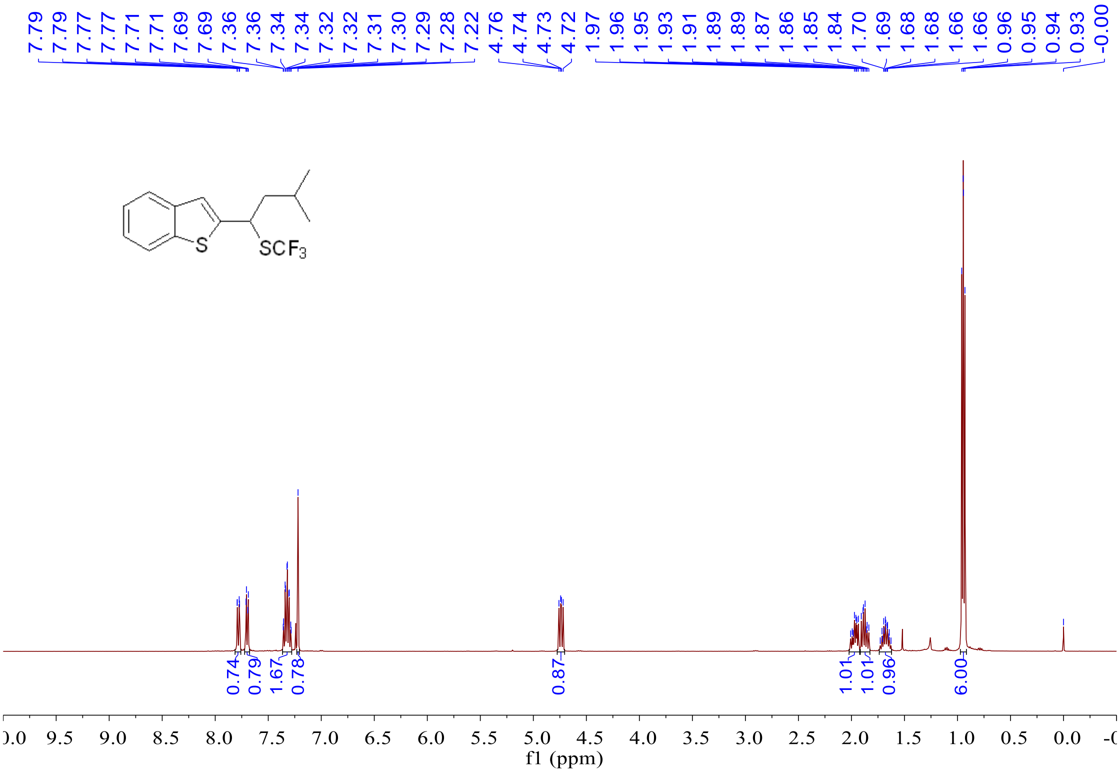

Supplementary Figure 34. ^{1}H NMR spectra for compound **3a**

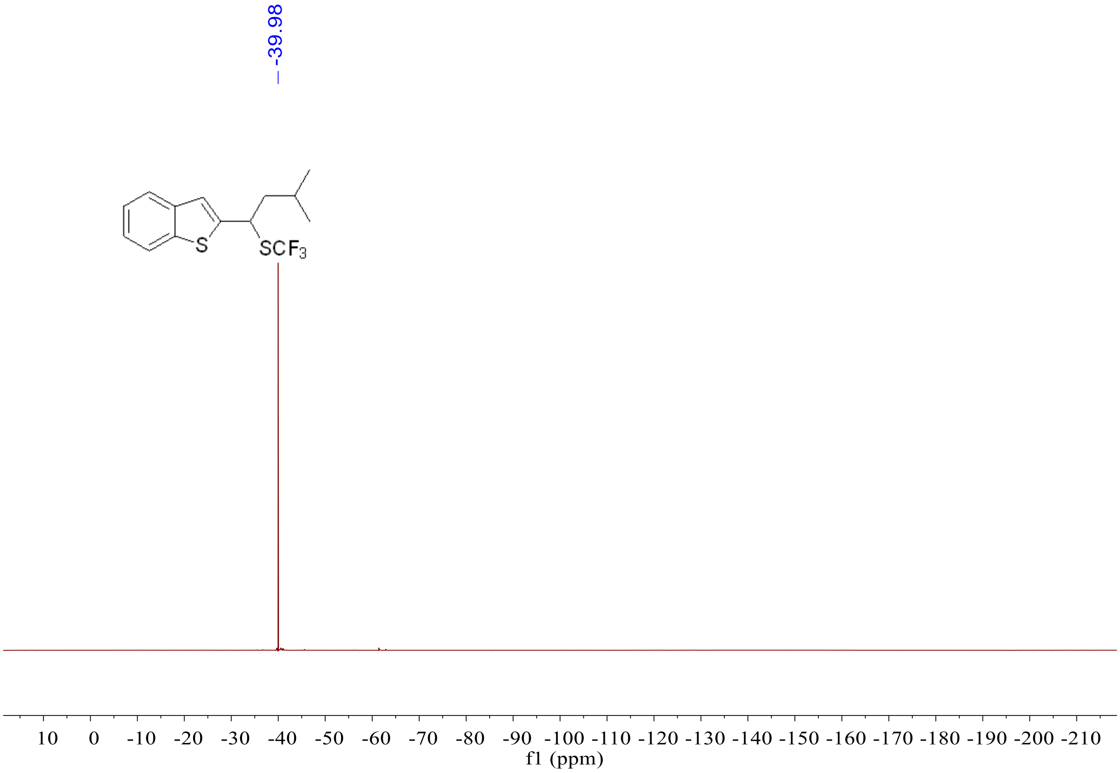

Supplementary Figure 35. ^{19}F NMR spectra for compound **3a**

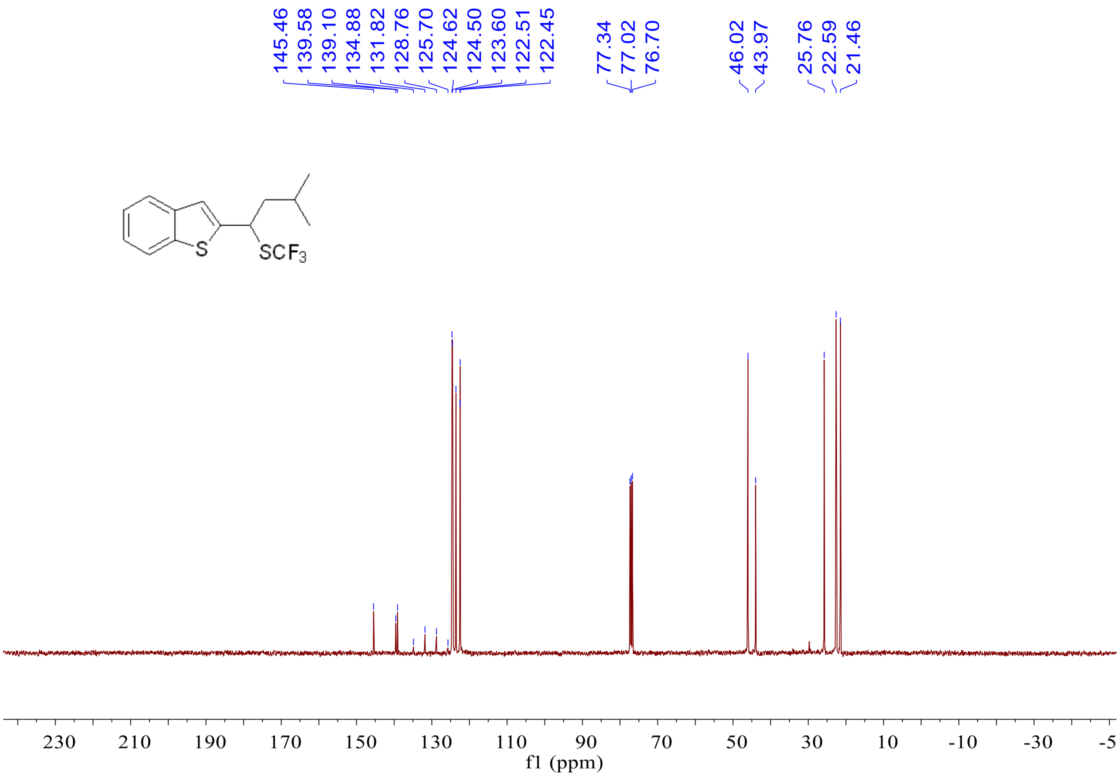

Supplementary Figure 36. ^{13}C NMR spectra for compound **3a**

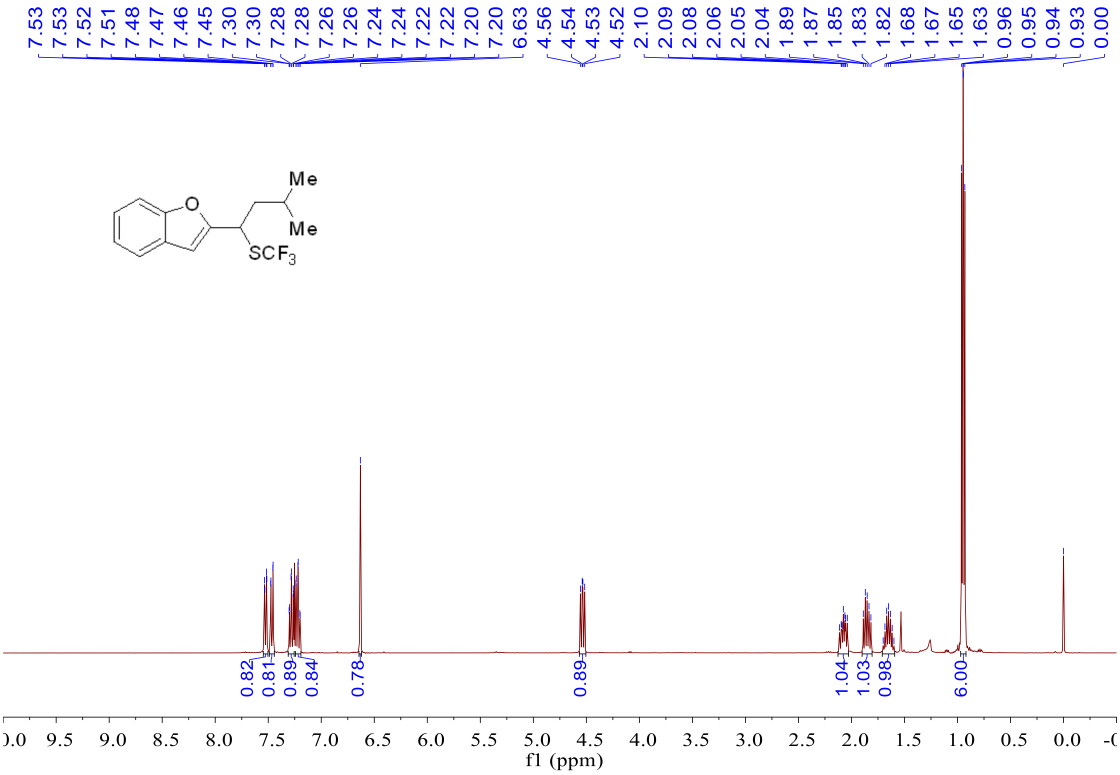

Supplementary Figure 37. ^{1}H NMR spectra for compound **3b**

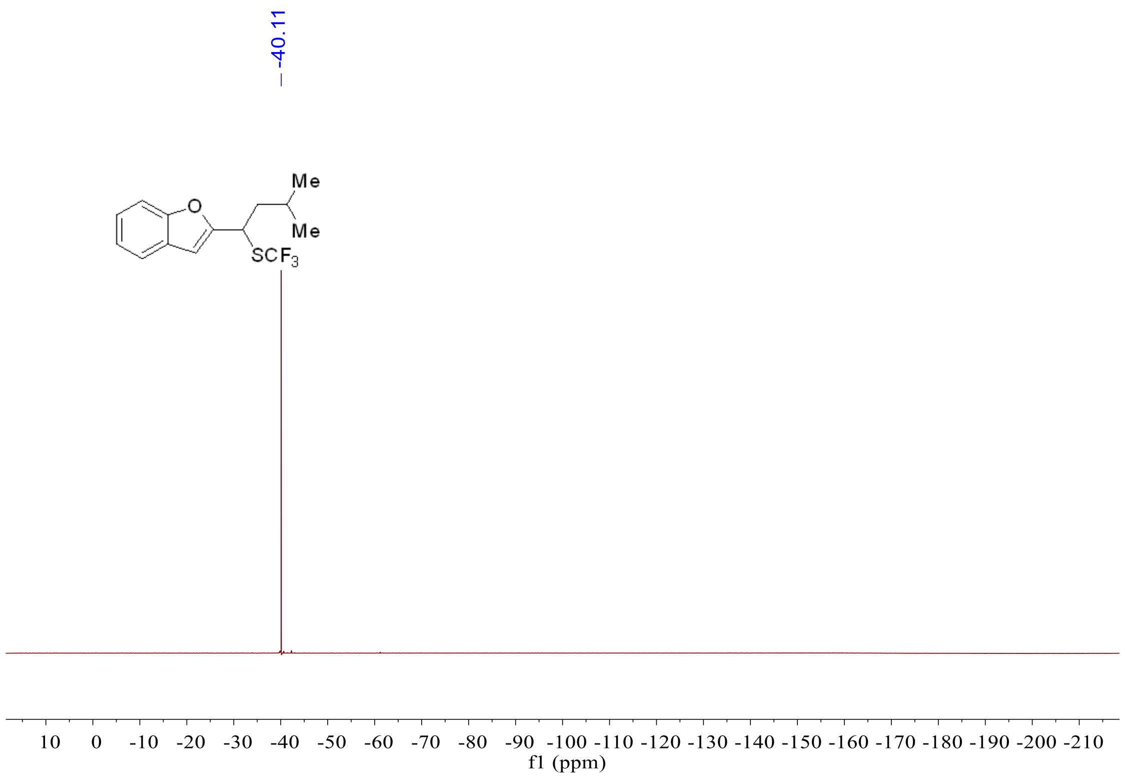

Supplementary Figure 38. ^{19}F NMR spectra for compound **3b**

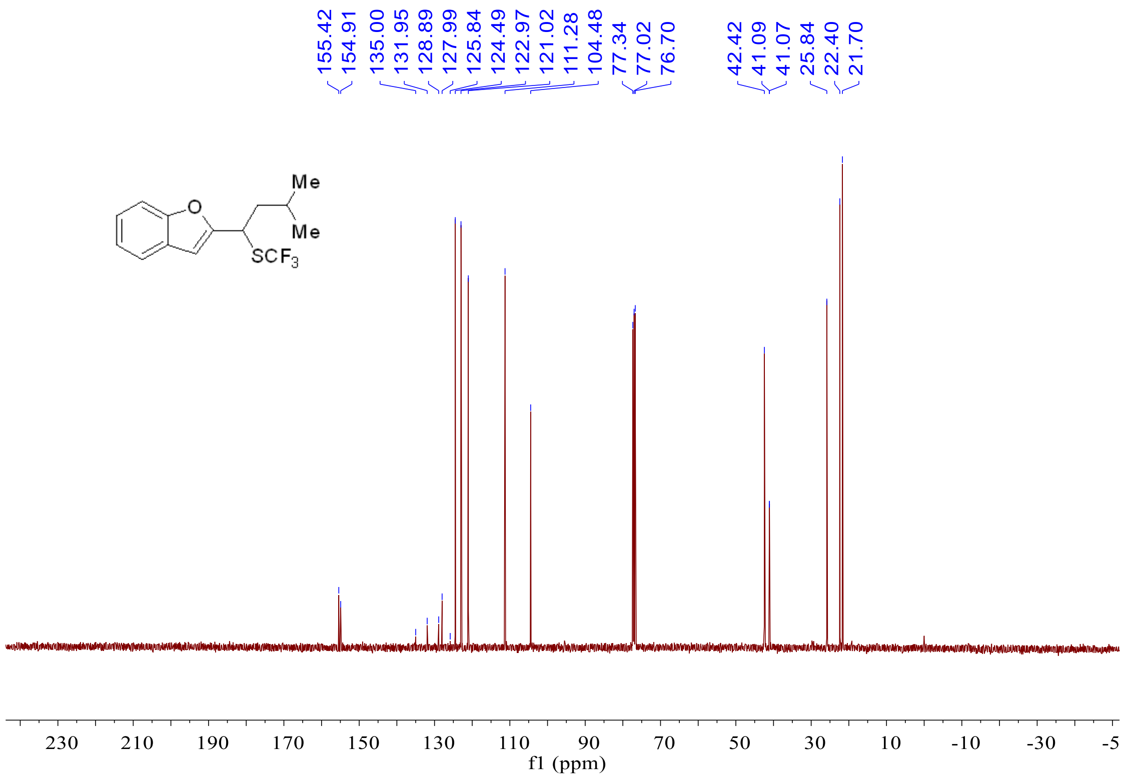

Supplementary Figure 39. ^{13}C NMR spectra for compound **3b**

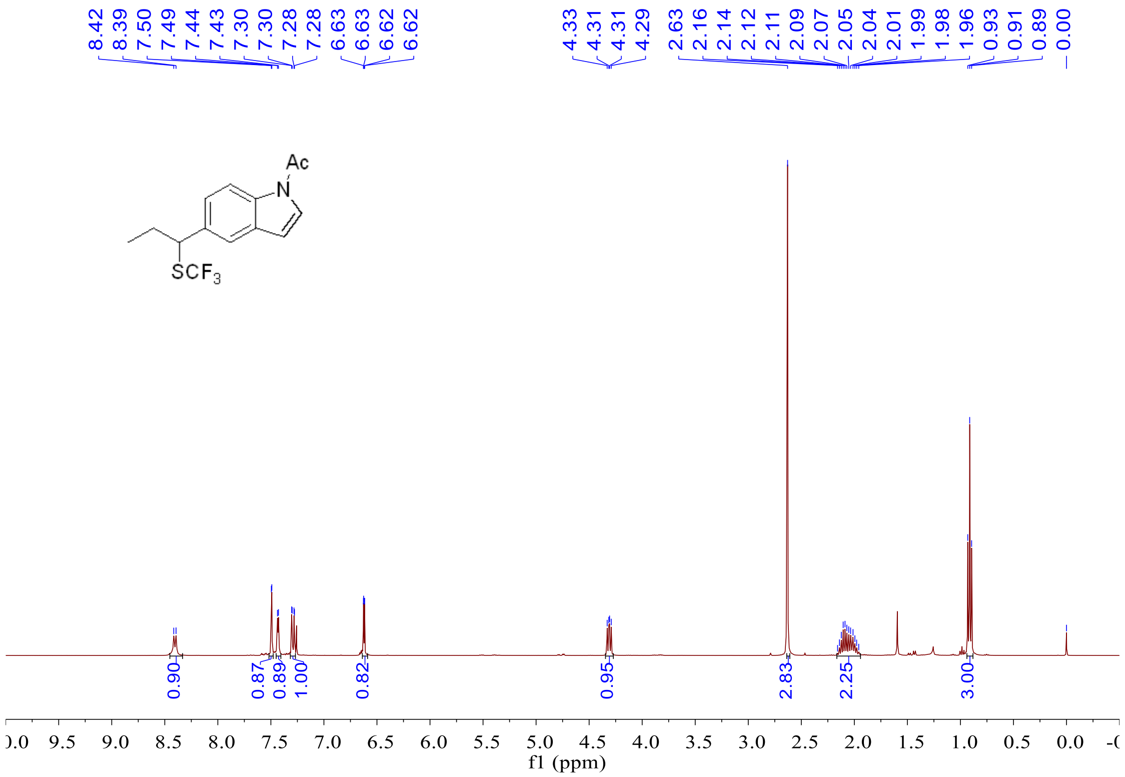

Supplementary Figure 40. ^{1}H NMR spectra for compound **3c**

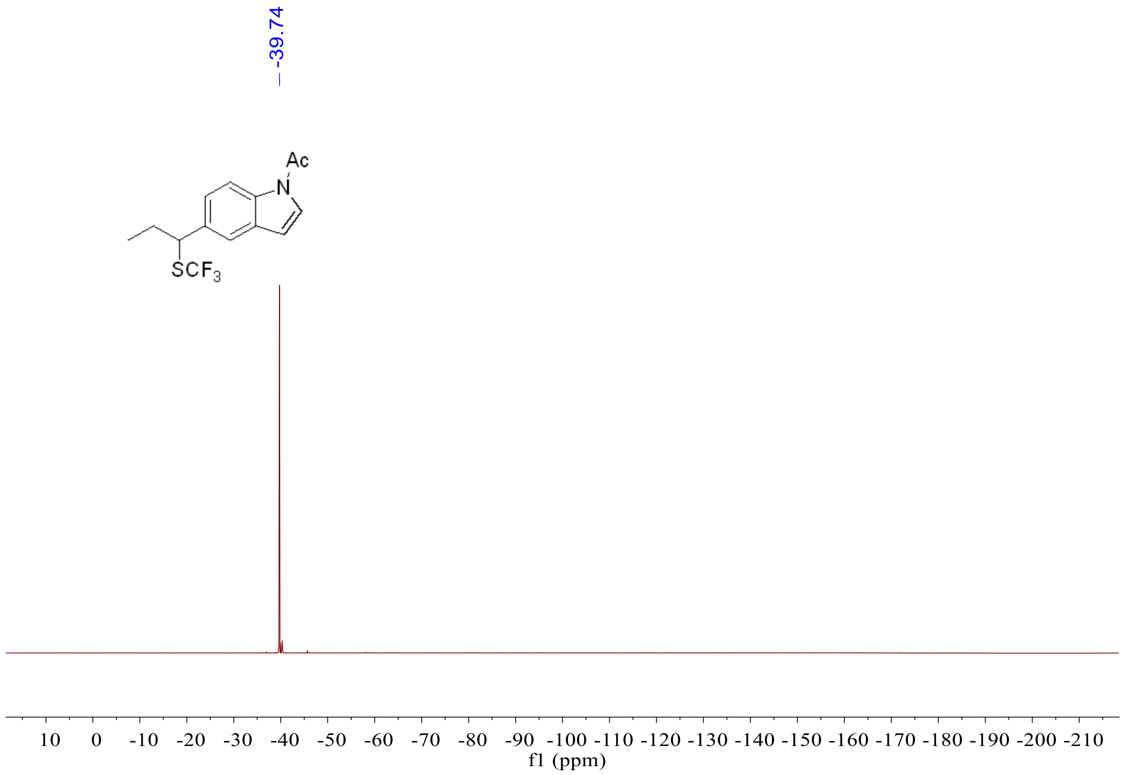

Supplementary Figure 41. ^{19}F NMR spectra for compound **3c**

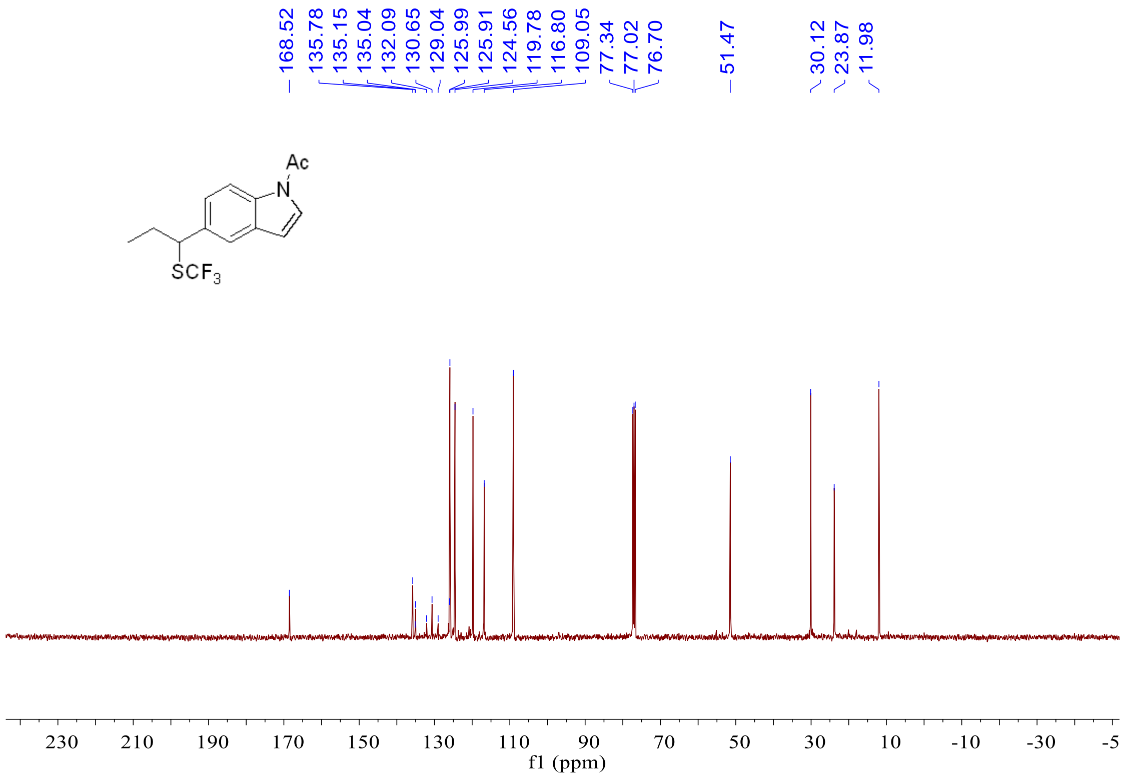

Supplementary Figure 42. ^{13}C NMR spectra for compound **3c**

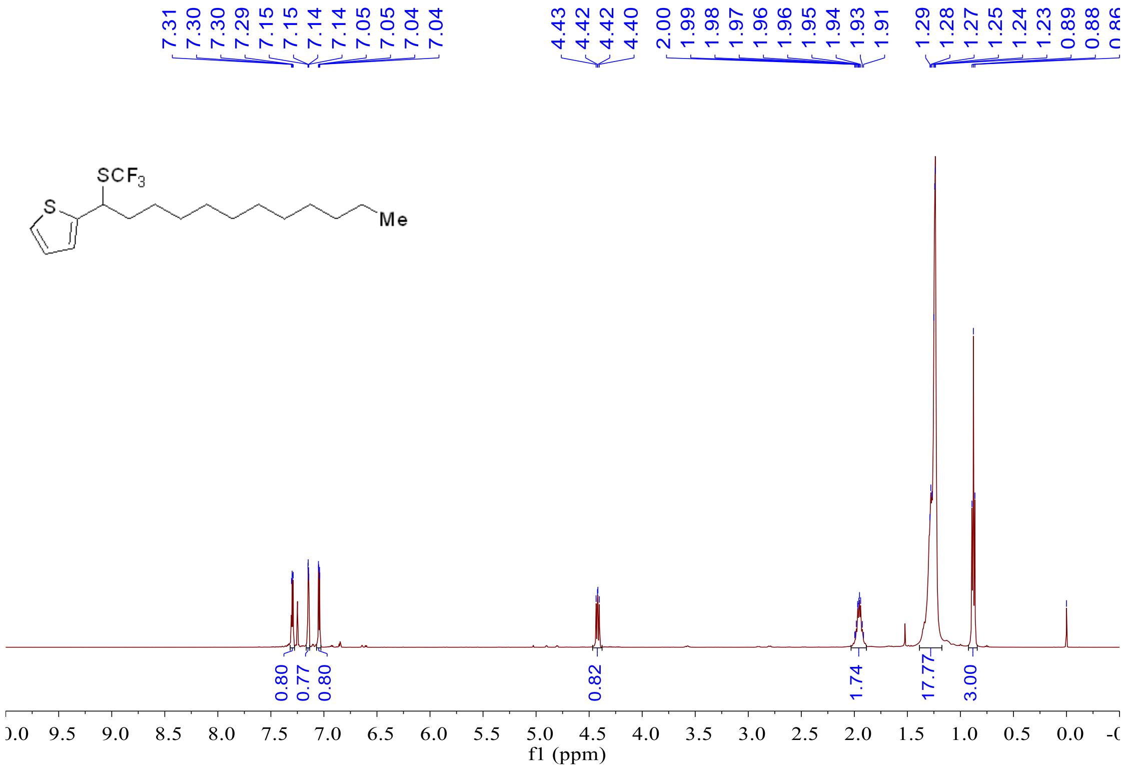

Supplementary Figure 43. ^{1}H NMR spectra for compound **3d**

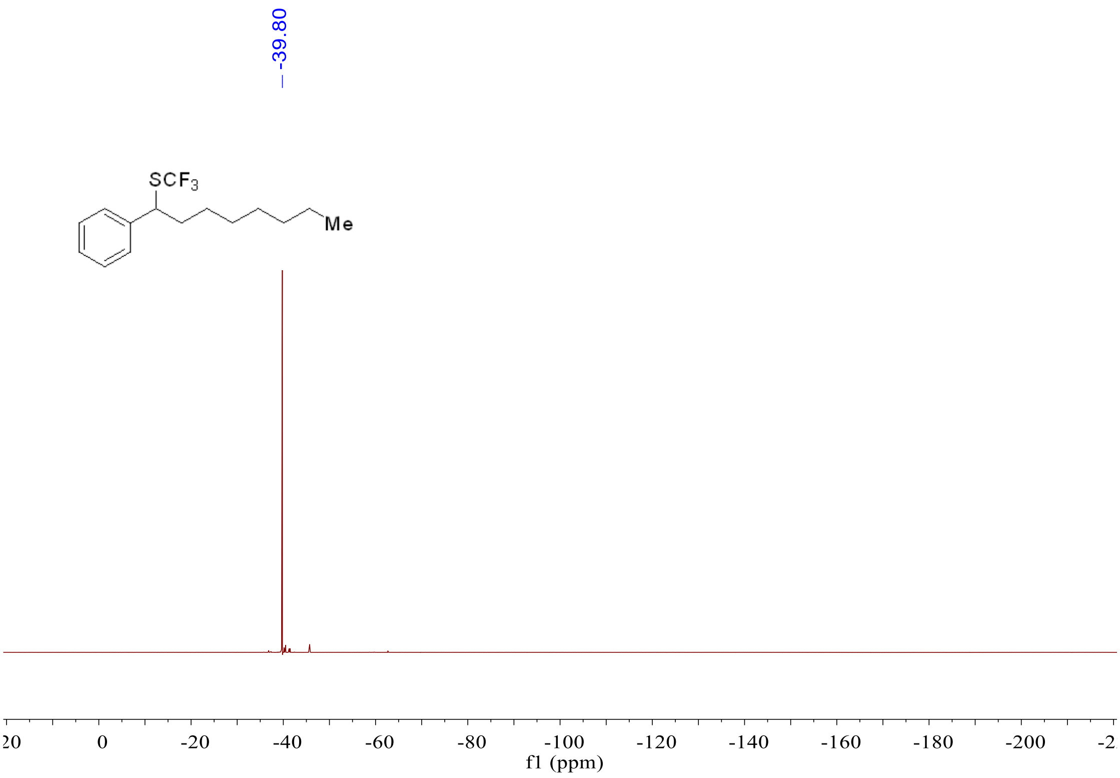

Supplementary Figure 44. ^{19}F NMR spectra for compound **3d**

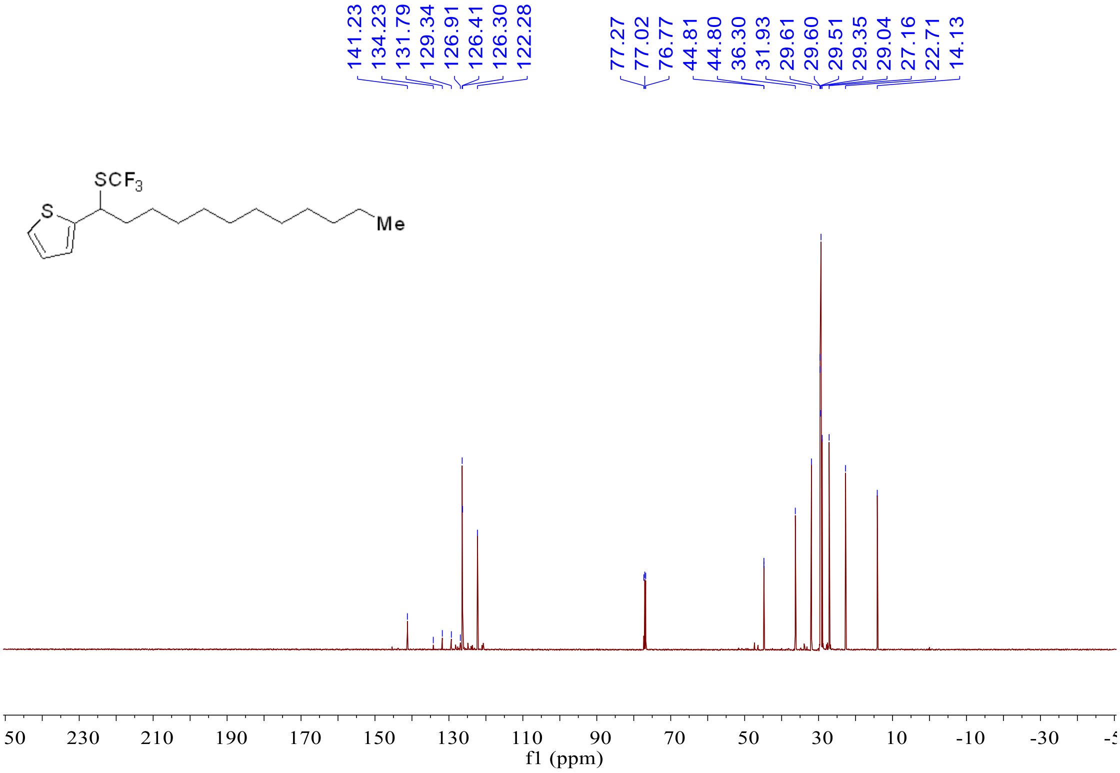

Supplementary Figure 45. ^{13}C NMR spectra for compound **3d**

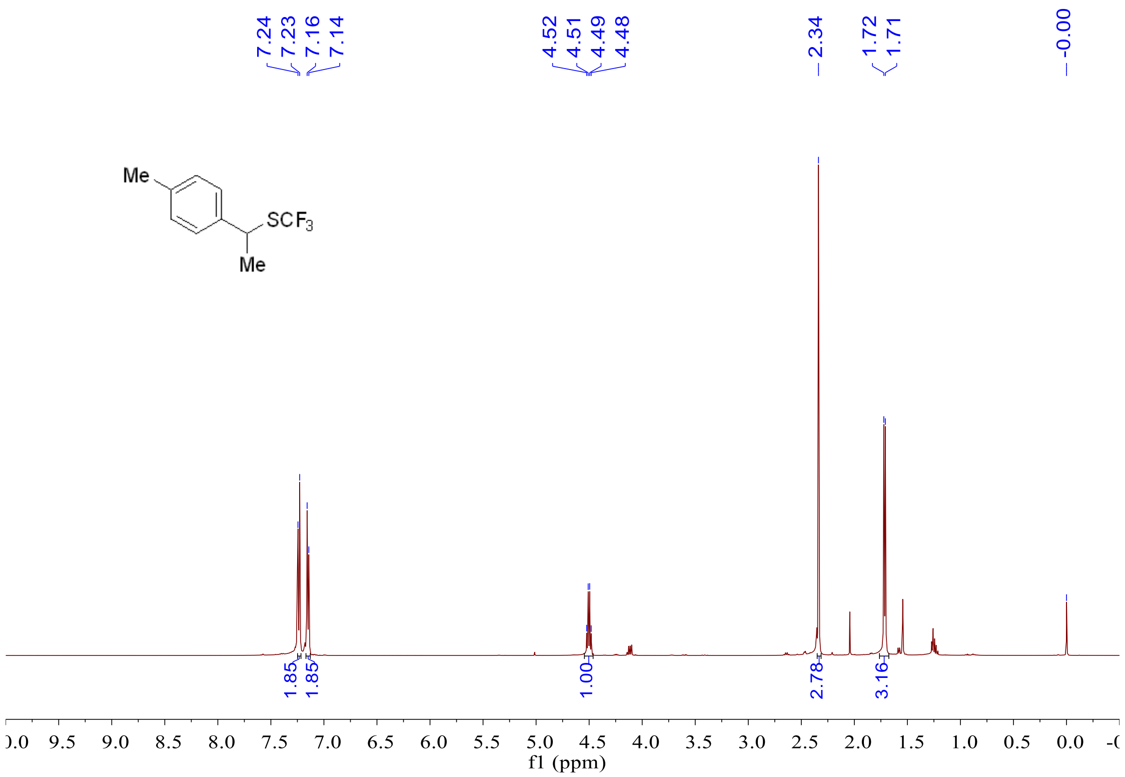

Supplementary Figure 46. ^{1}H NMR spectra for compound **3e**

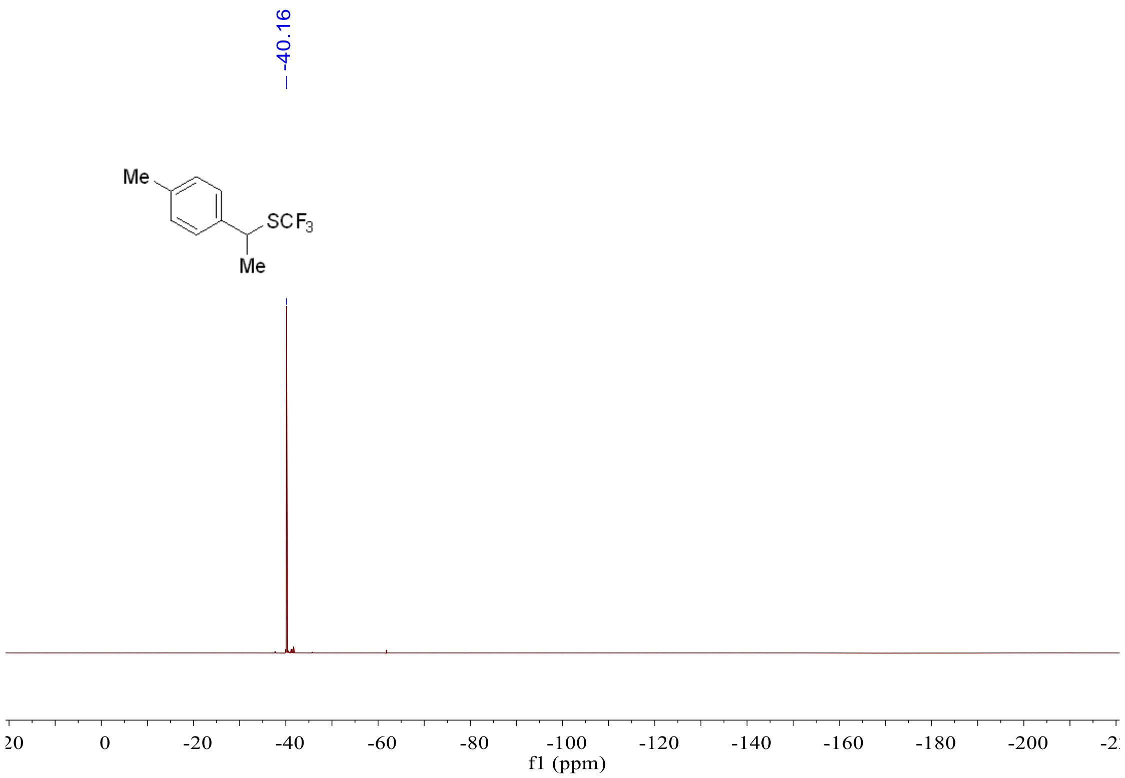

Supplementary Figure 47. ^{19}F NMR spectra for compound **3e**

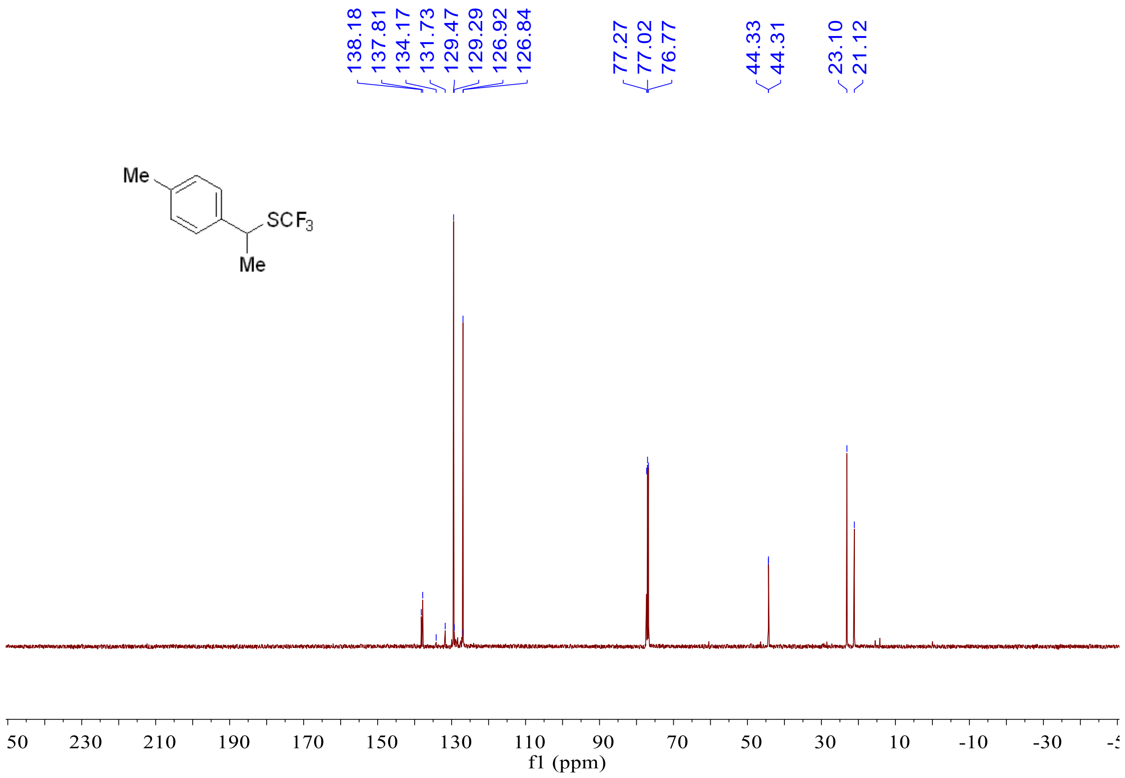

Supplementary Figure 48. ^{13}C NMR spectra for compound **3e**

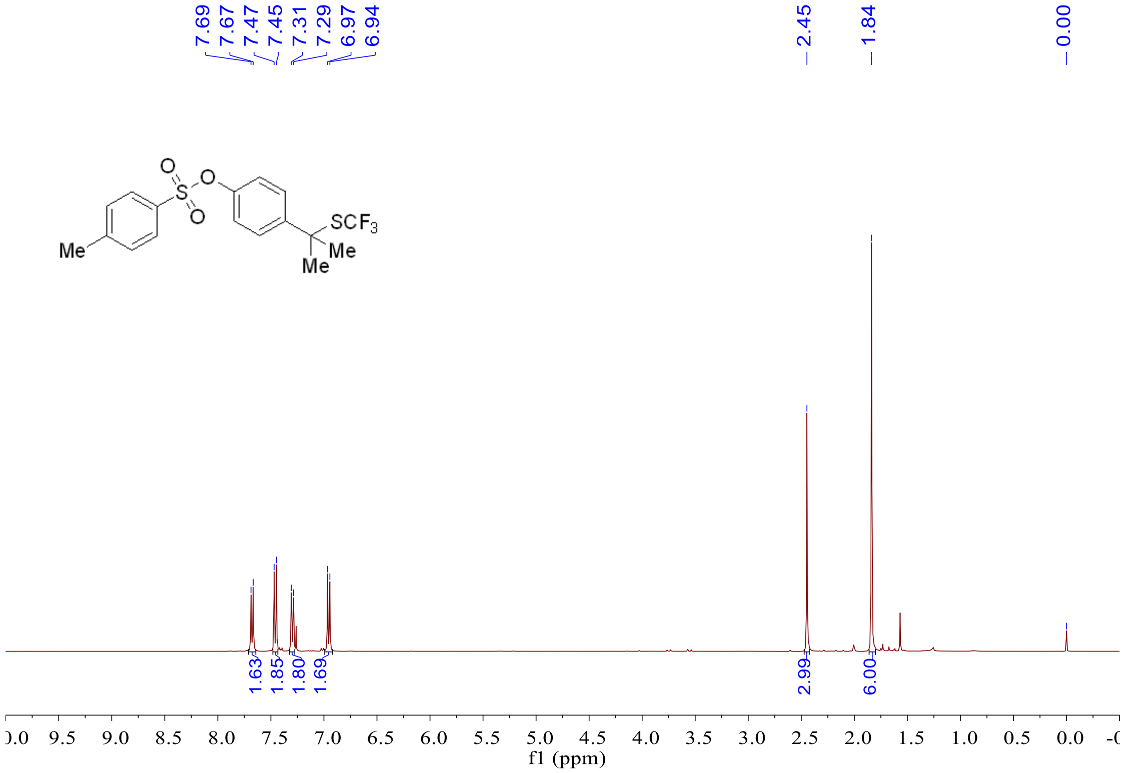

Supplementary Figure 49. ^{1}H NMR spectra for compound **3f**

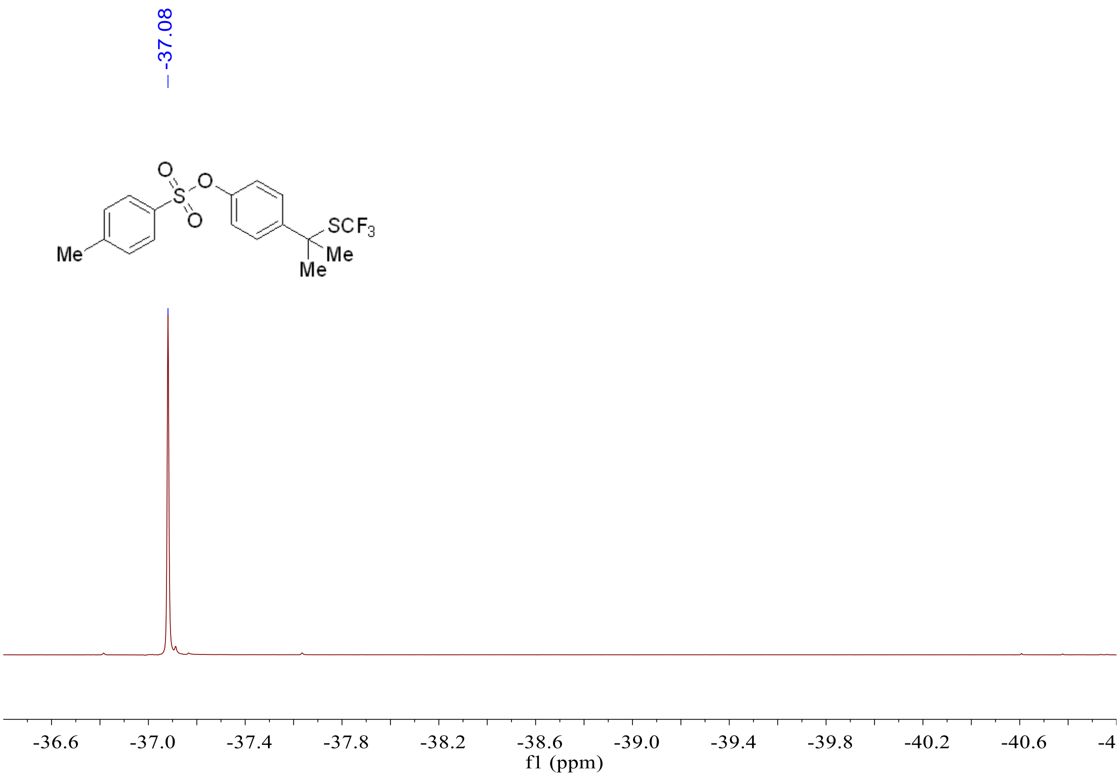

Supplementary Figure 50. ^{19}F NMR spectra for compound **3f**

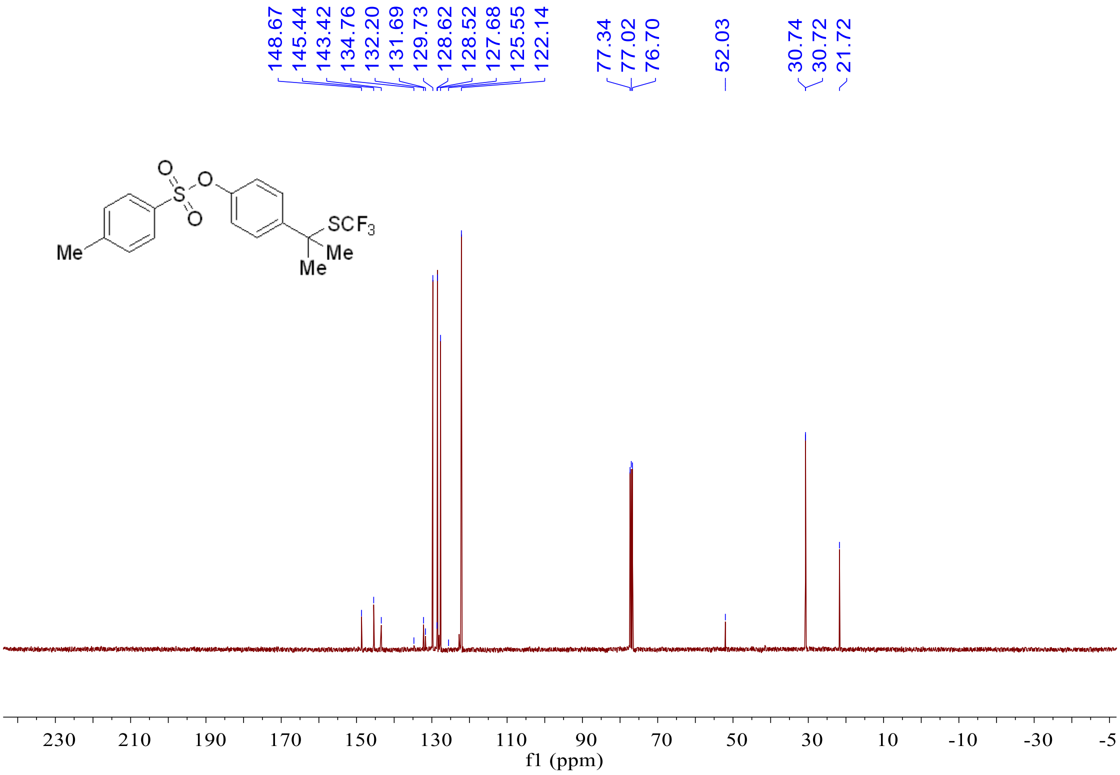

Supplementary Figure 51. ^{13}C NMR spectra for compound **3f**

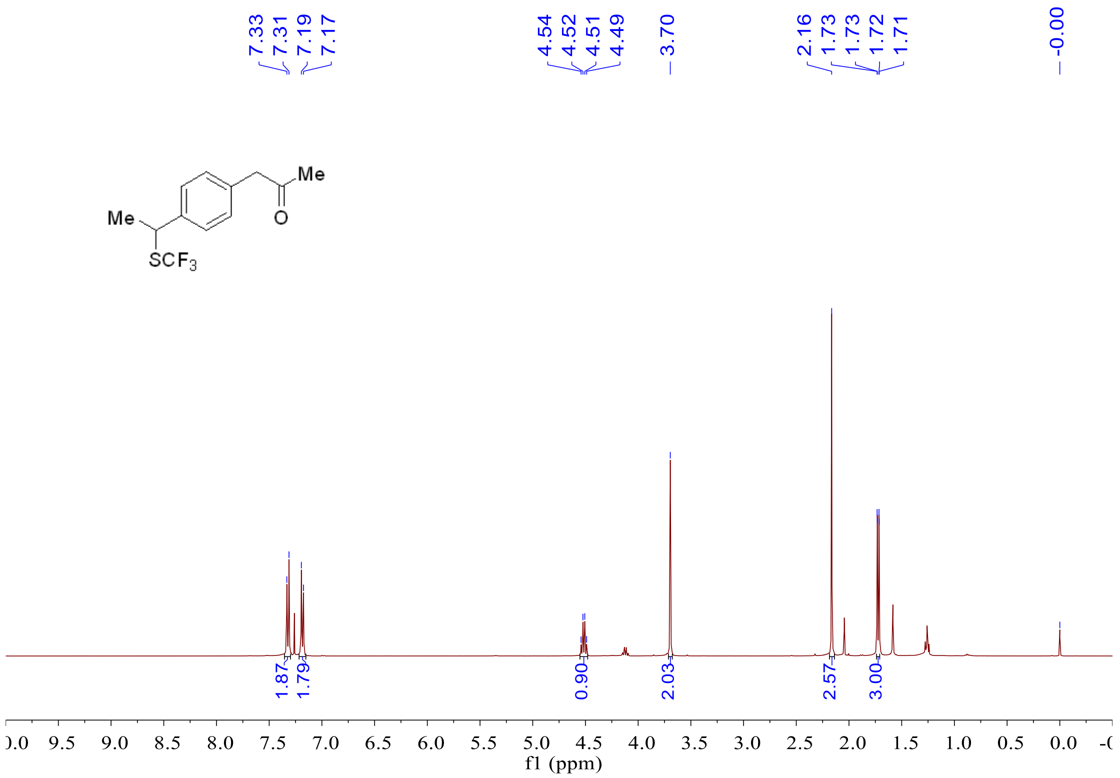

Supplementary Figure 52. ^{1}H NMR spectra for compound **3g**

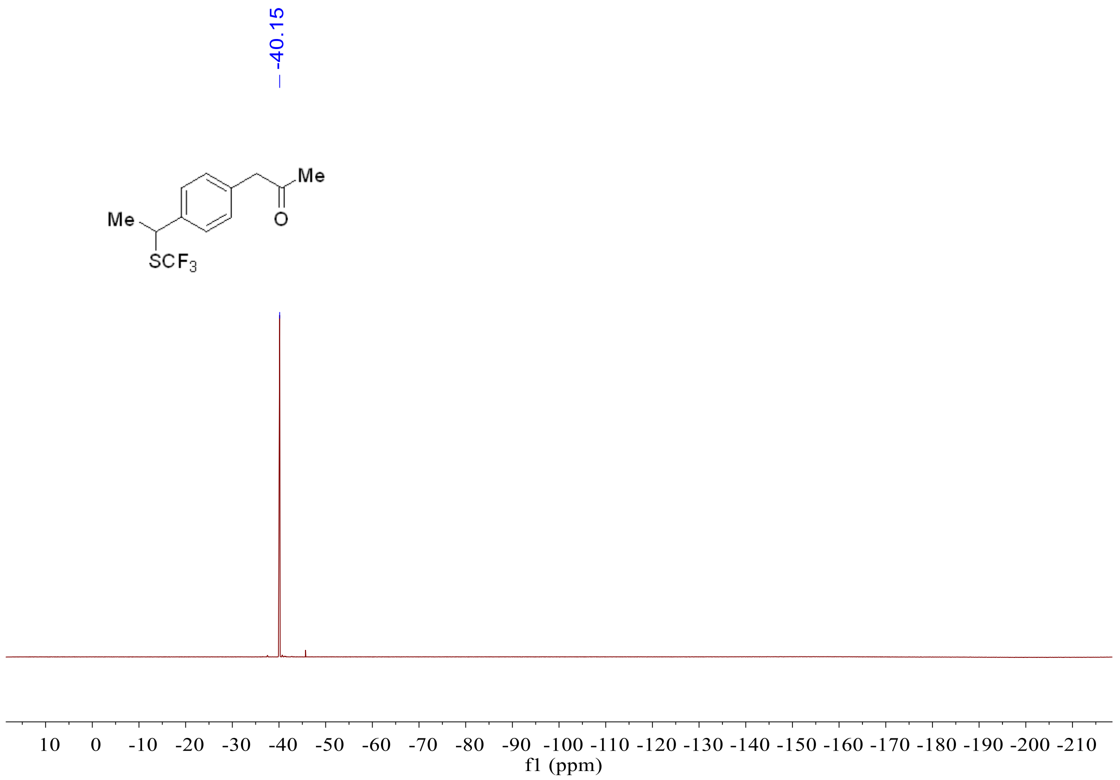

Supplementary Figure 53. ^{19}F NMR spectra for compound **3g**

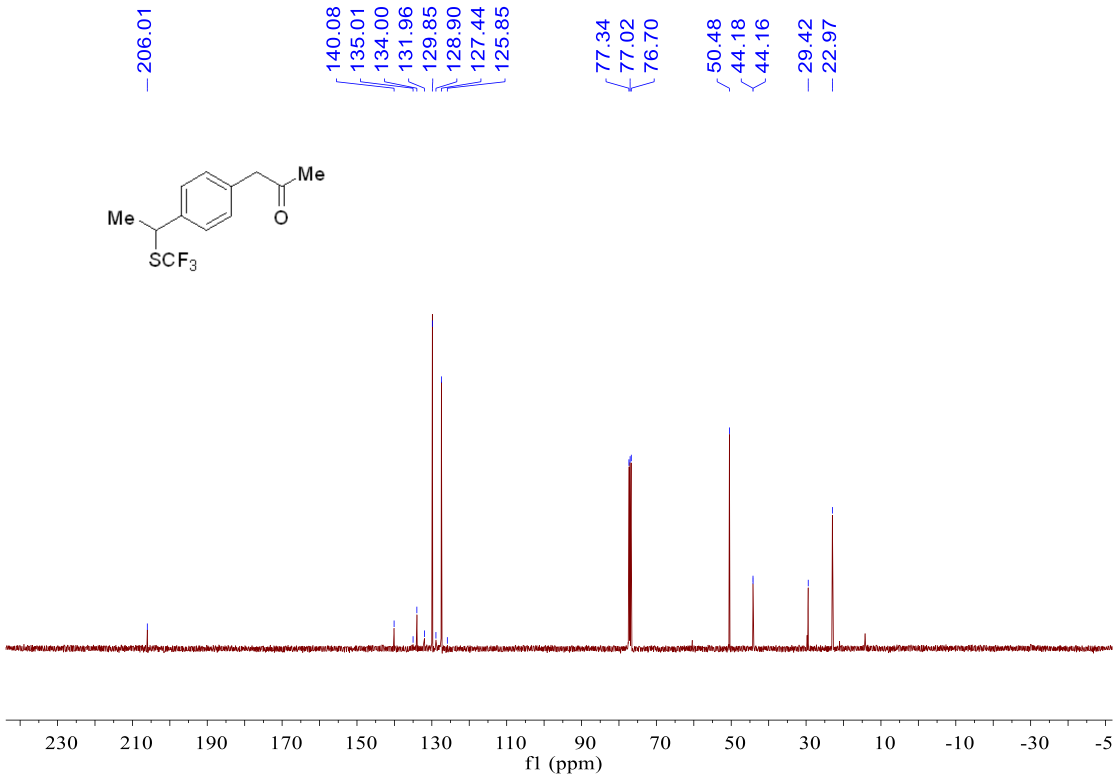

Supplementary Figure 54. ^{13}C NMR spectra for compound **3g**

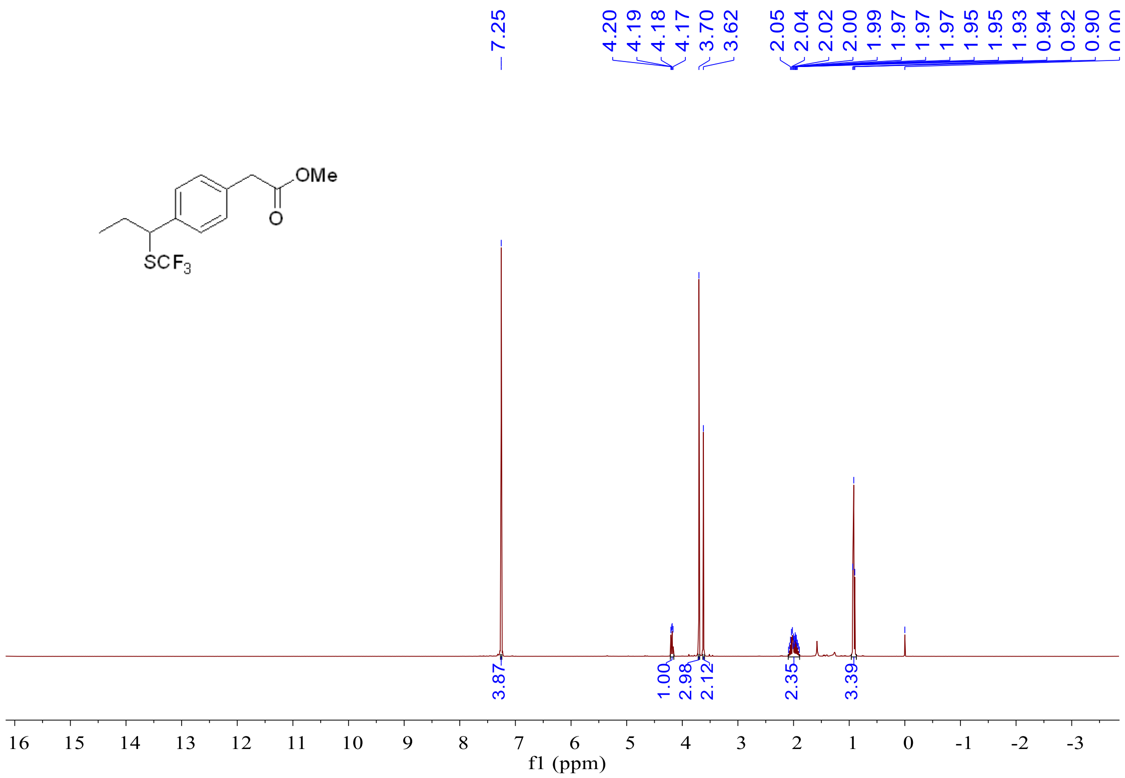

Supplementary Figure 55. ^{1}H NMR spectra for compound **3h**

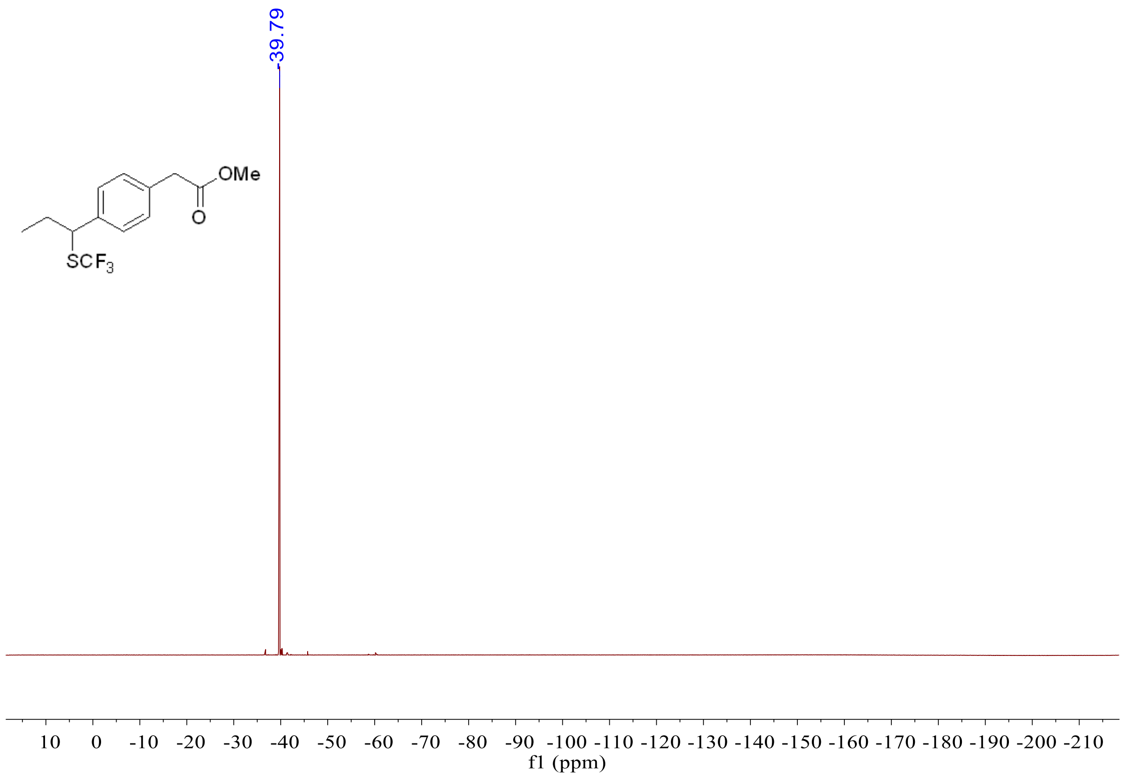

Supplementary Figure 56. ^{19}F NMR spectra for compound **3h**

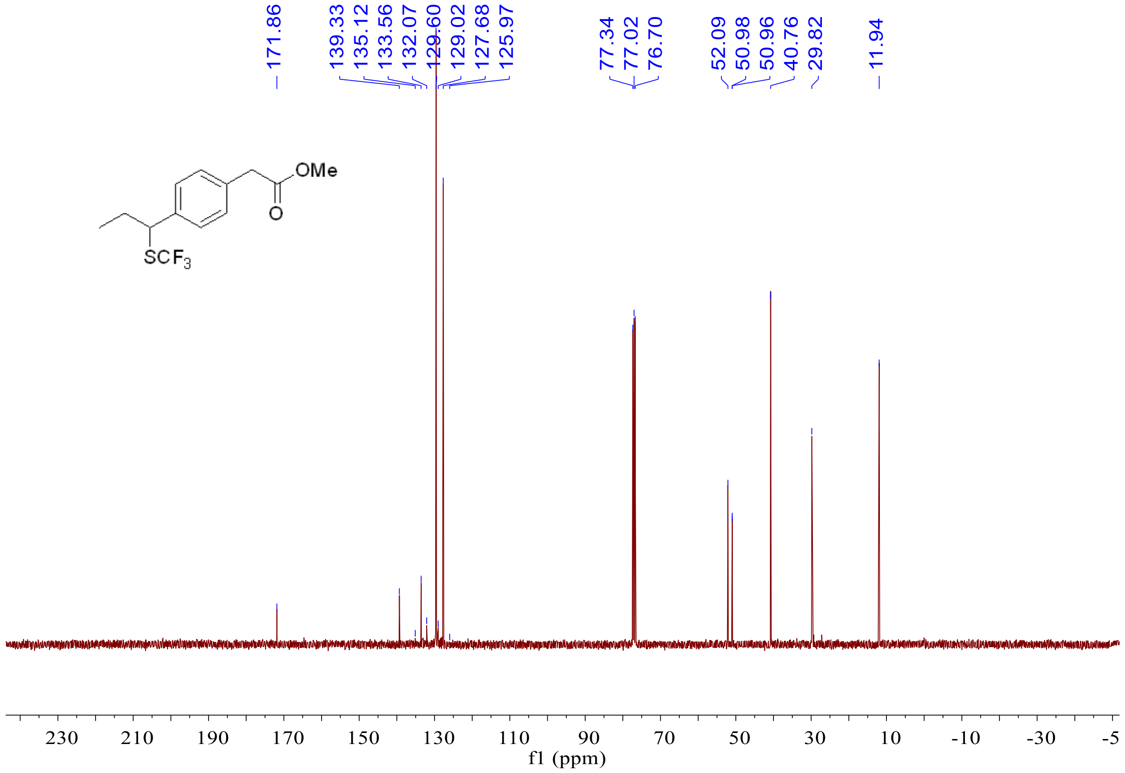

Supplementary Figure 57. ^{13}C NMR spectra for compound **3h**

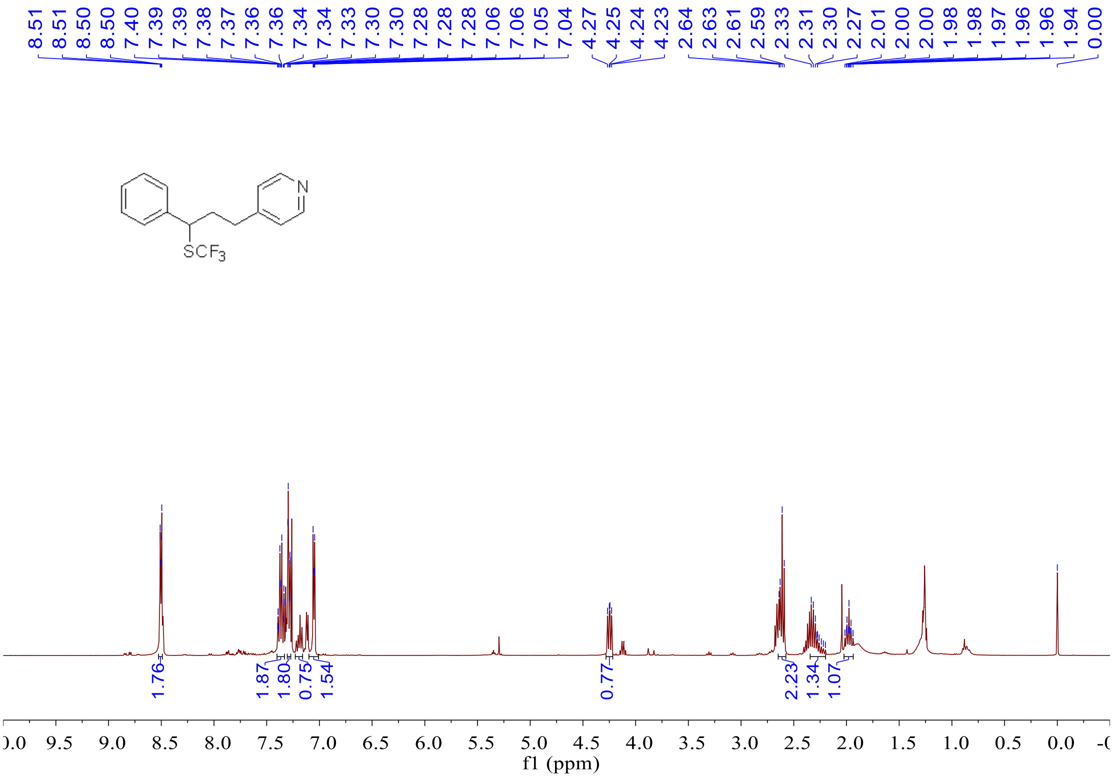

Supplementary Figure 58. ^{1}H NMR spectra for compound **3i**

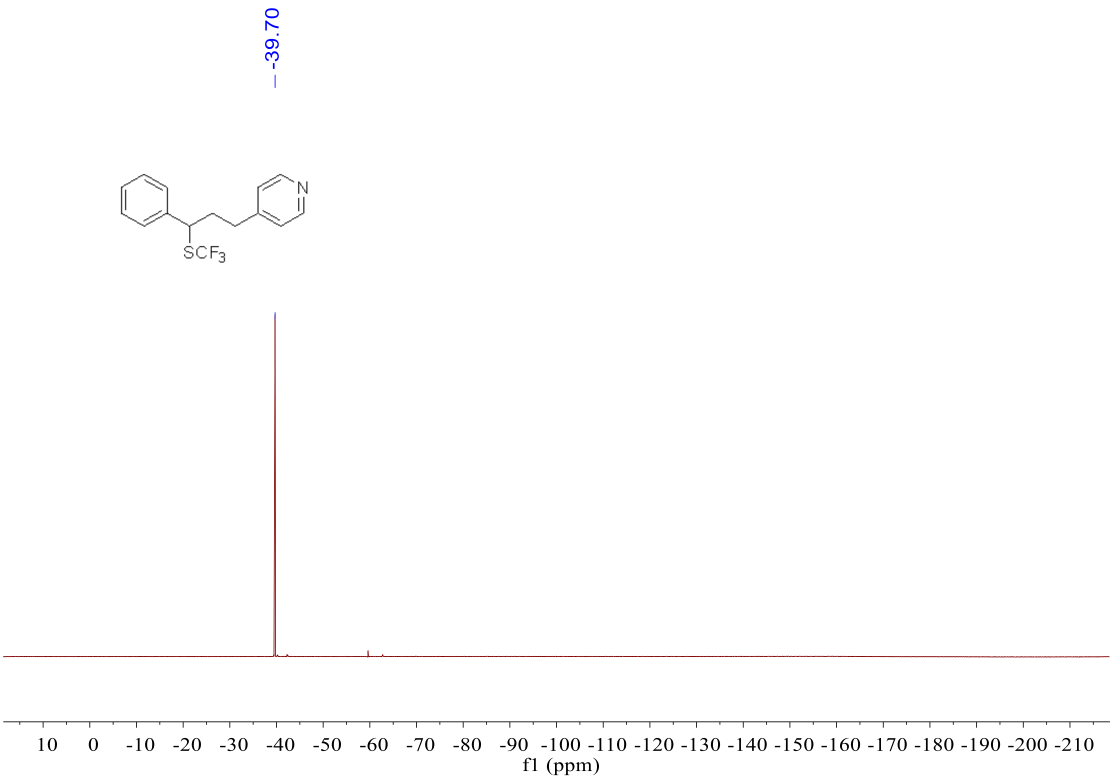

Supplementary Figure 59. ^{19}F NMR spectra for compound **3i**

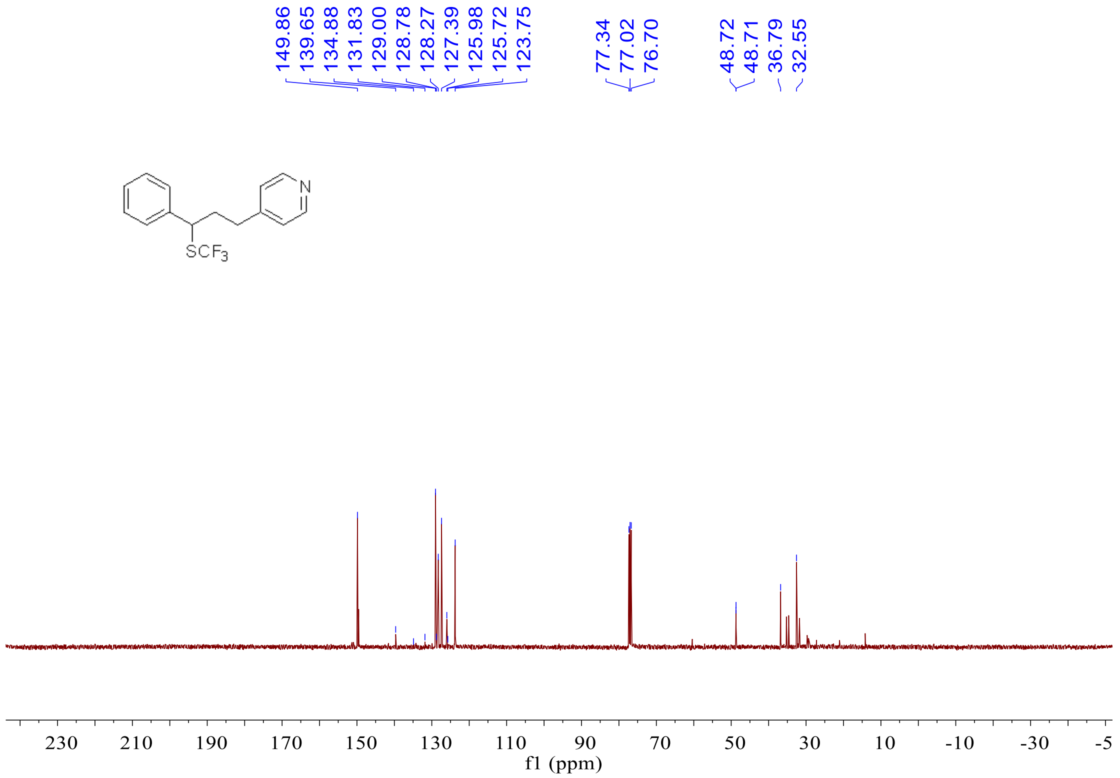

Supplementary Figure 60. ^{13}C NMR spectra for compound **3i**

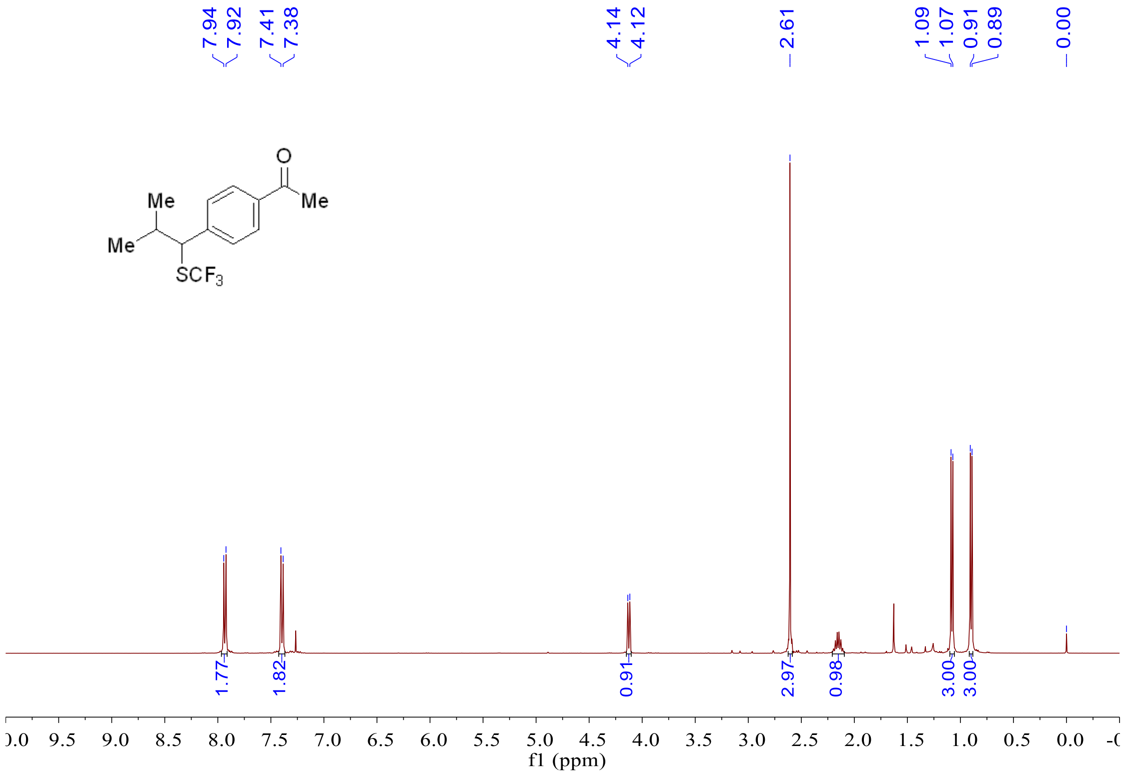

Supplementary Figure 61. ^{1}H NMR spectra for compound **3j**

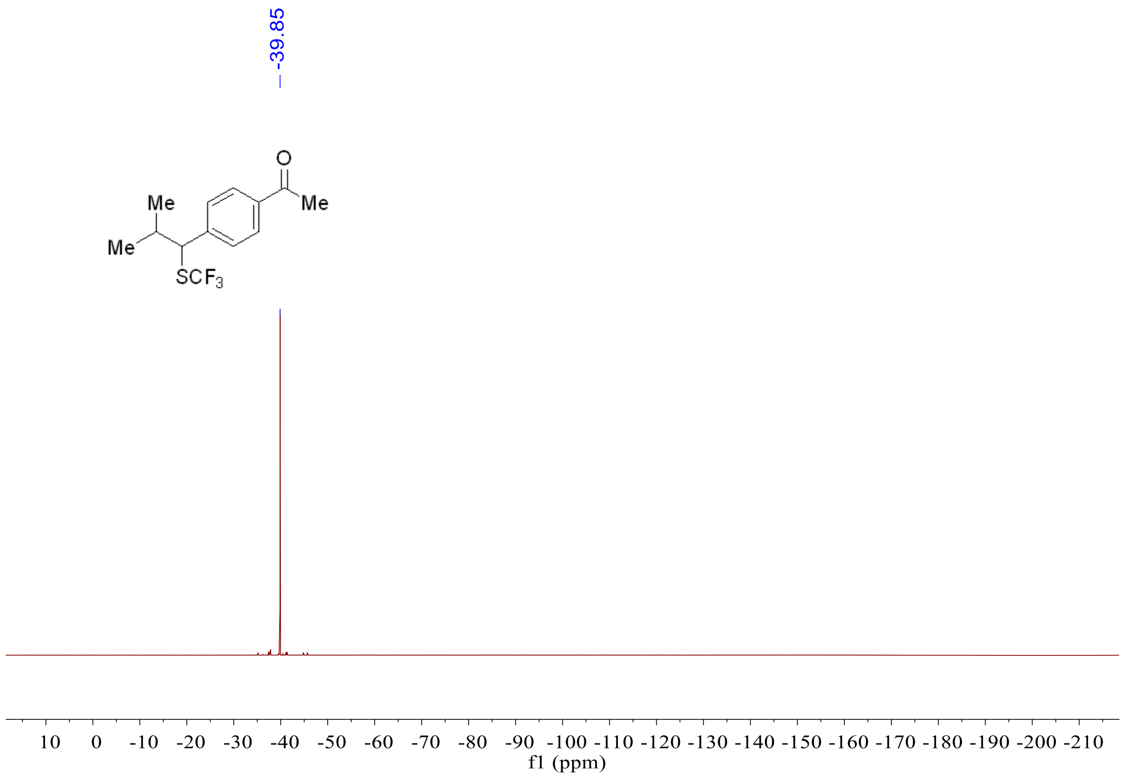

Supplementary Figure 62. ^{19}F NMR spectra for compound **3j**

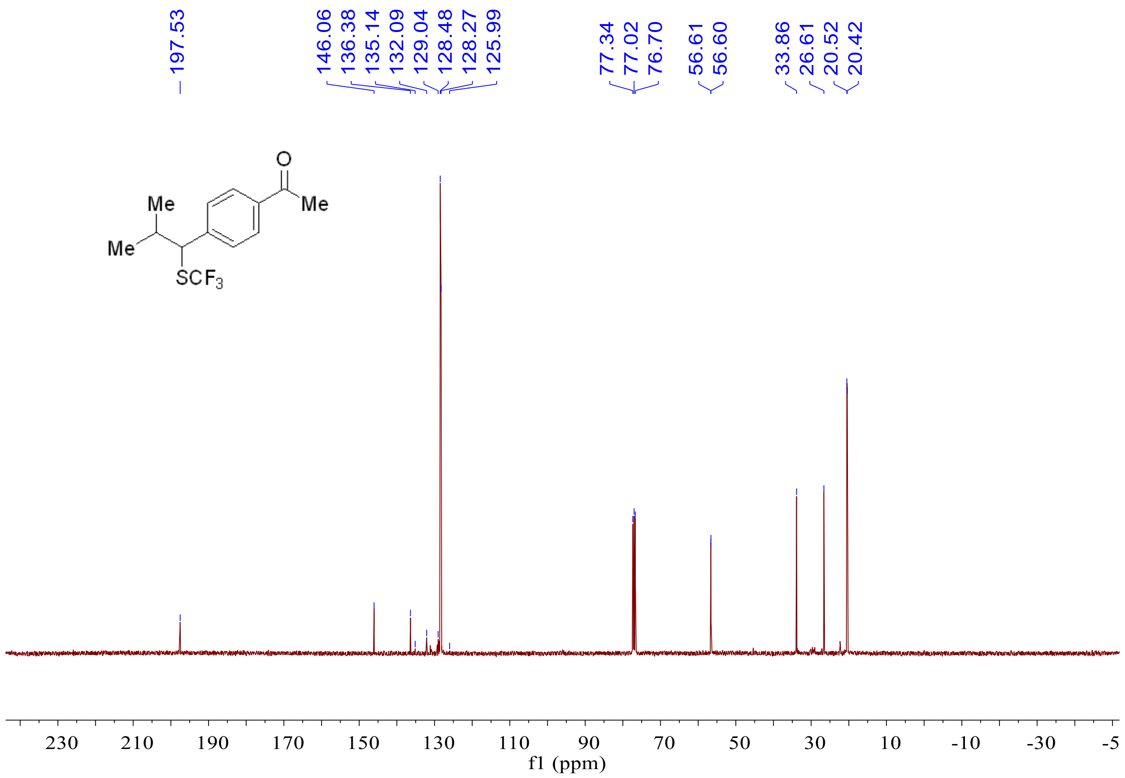

Supplementary Figure 63. ^{13}C NMR spectra for compound **3j**

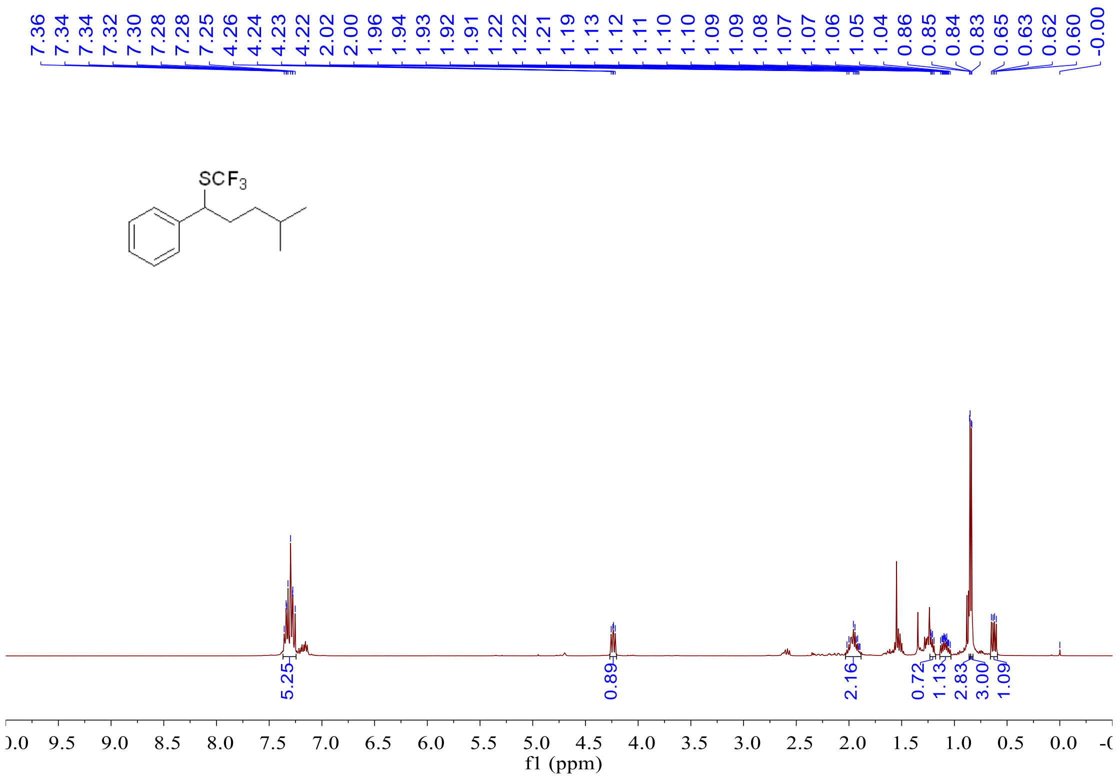

Supplementary Figure 64. ^{1}H NMR spectra for compound **3k**

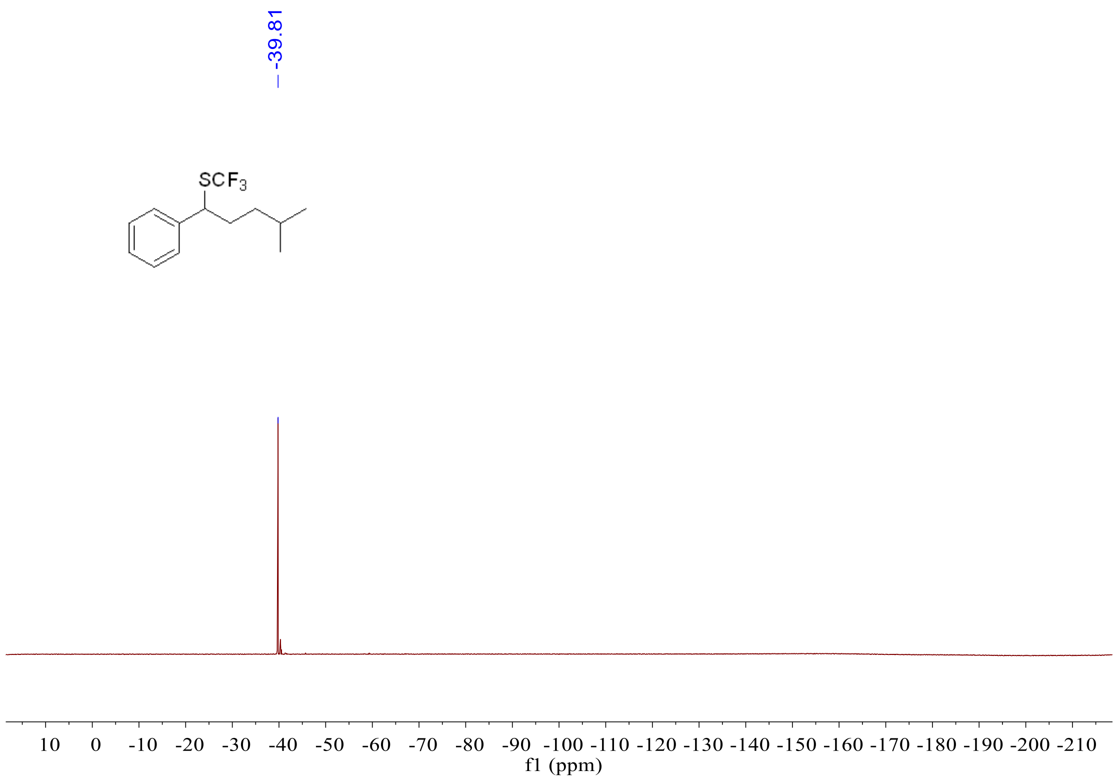

Supplementary Figure 65. ^{19}F NMR spectra for compound **3k**

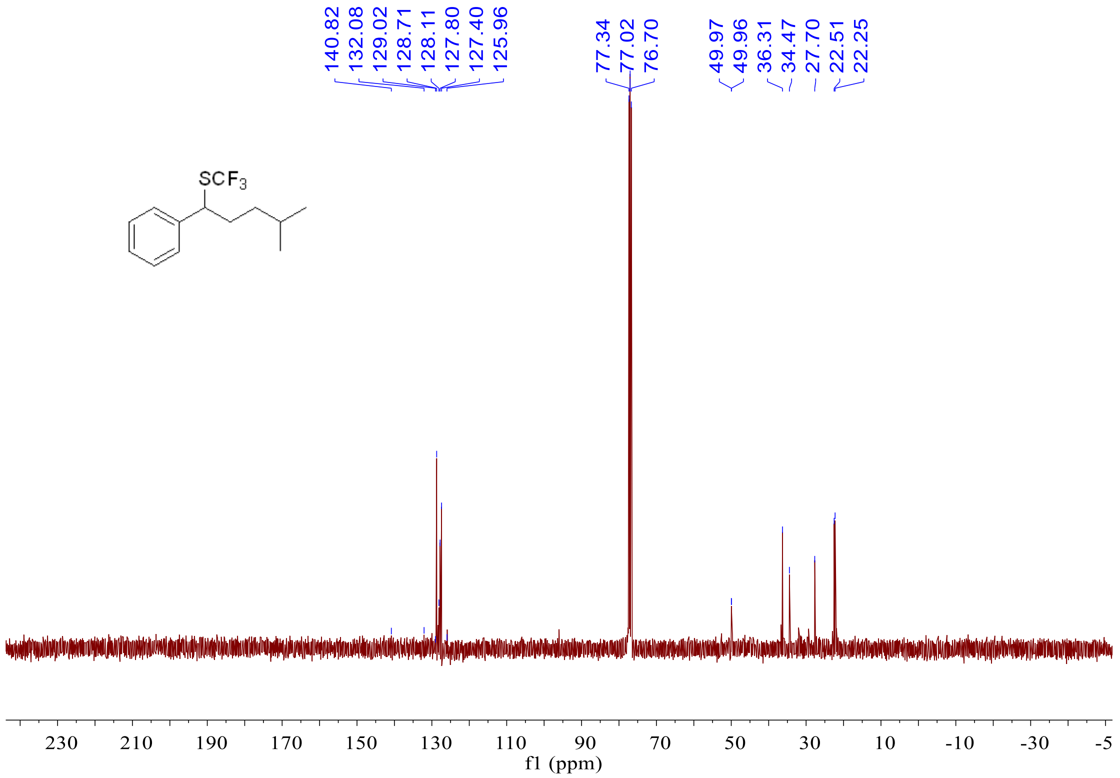

Supplementary Figure 66. ^{13}C NMR spectra for compound **3k**

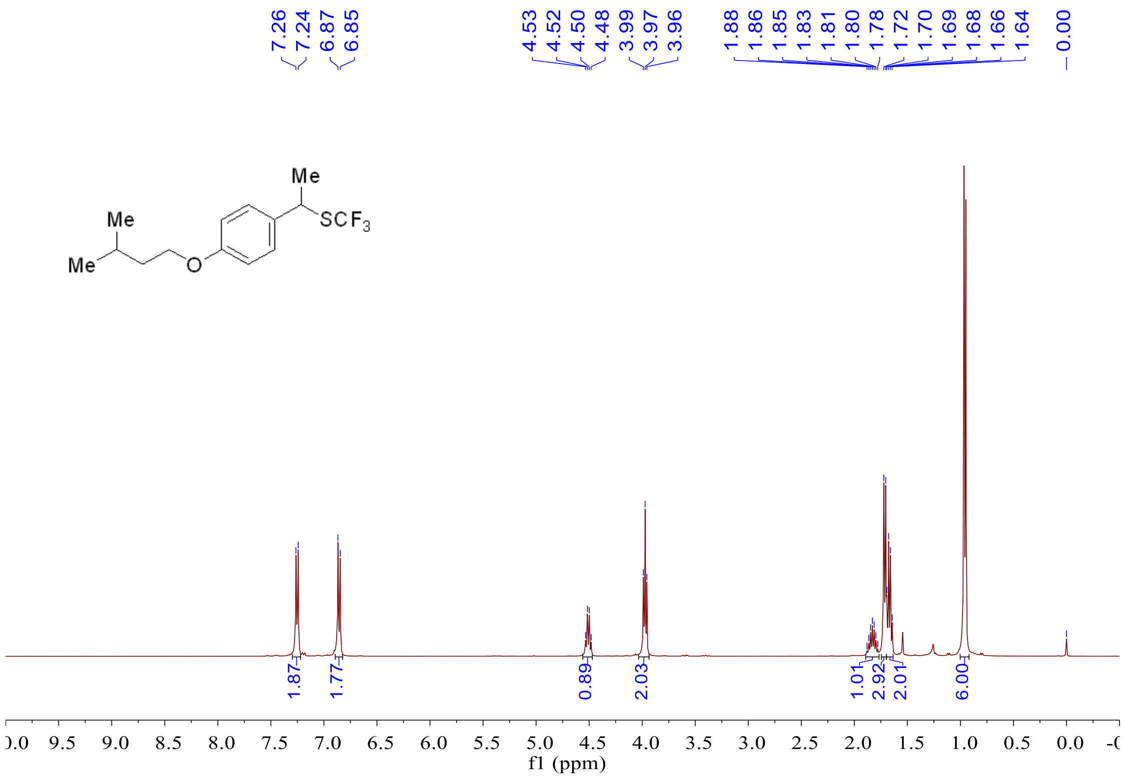

Supplementary Figure 67. ^{1}H NMR spectra for compound **3l**

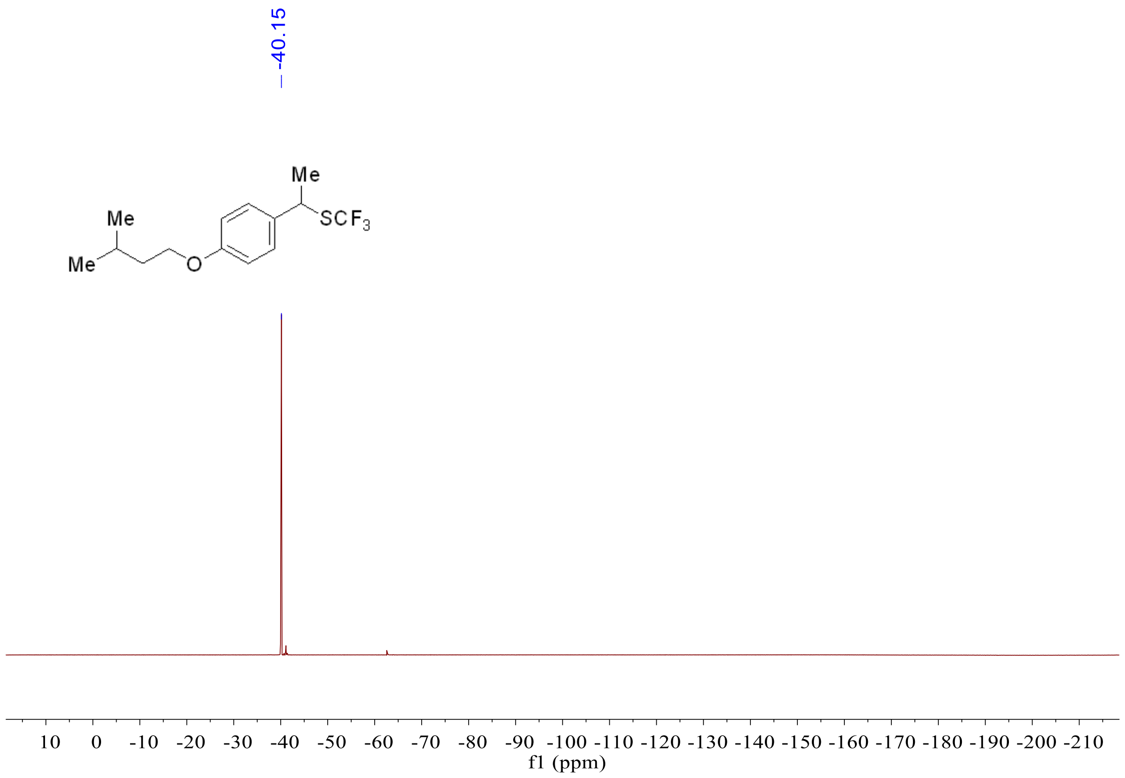

Supplementary Figure 68. ^{19}F NMR spectra for compound **3l**

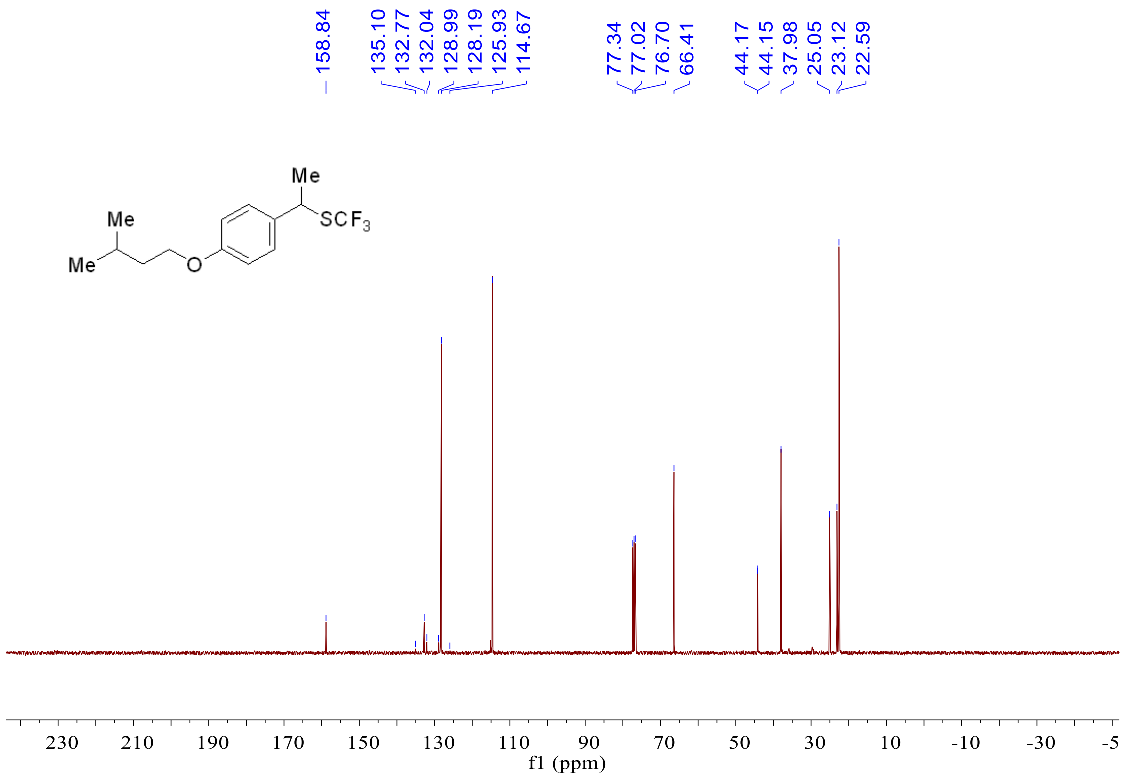

Supplementary Figure 69. ^{13}C NMR spectra for compound **3l**

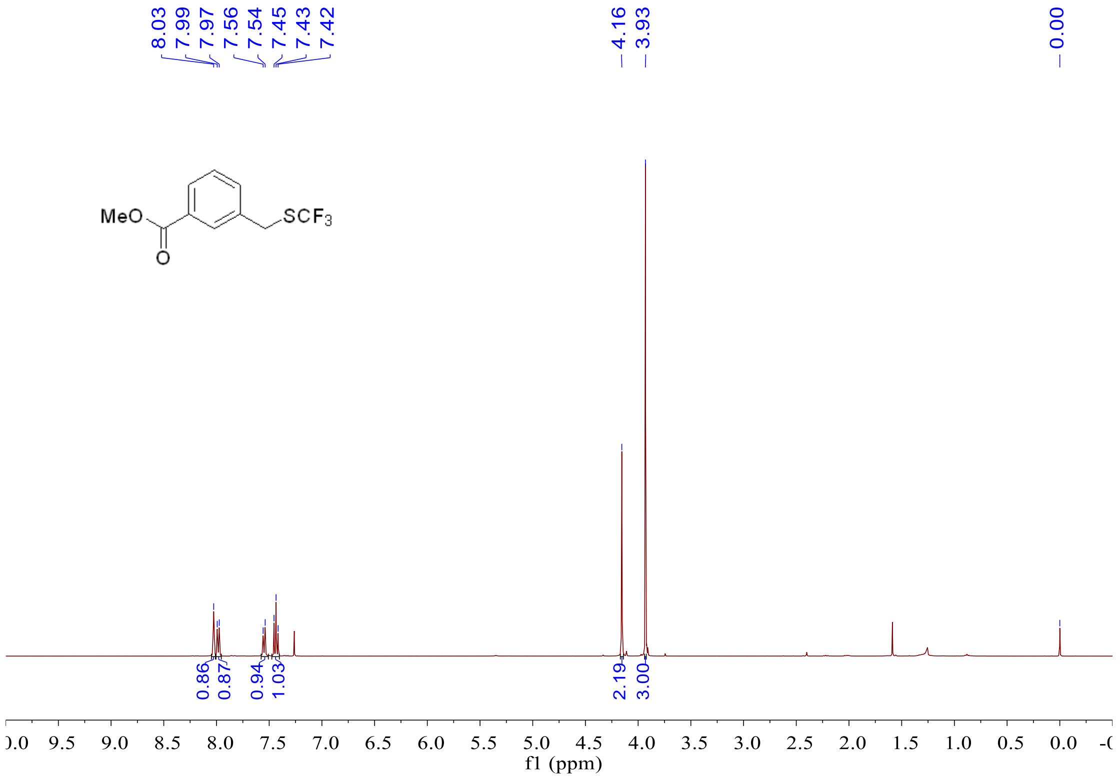

Supplementary Figure 70. ^{1}H NMR spectra for compound **3m**

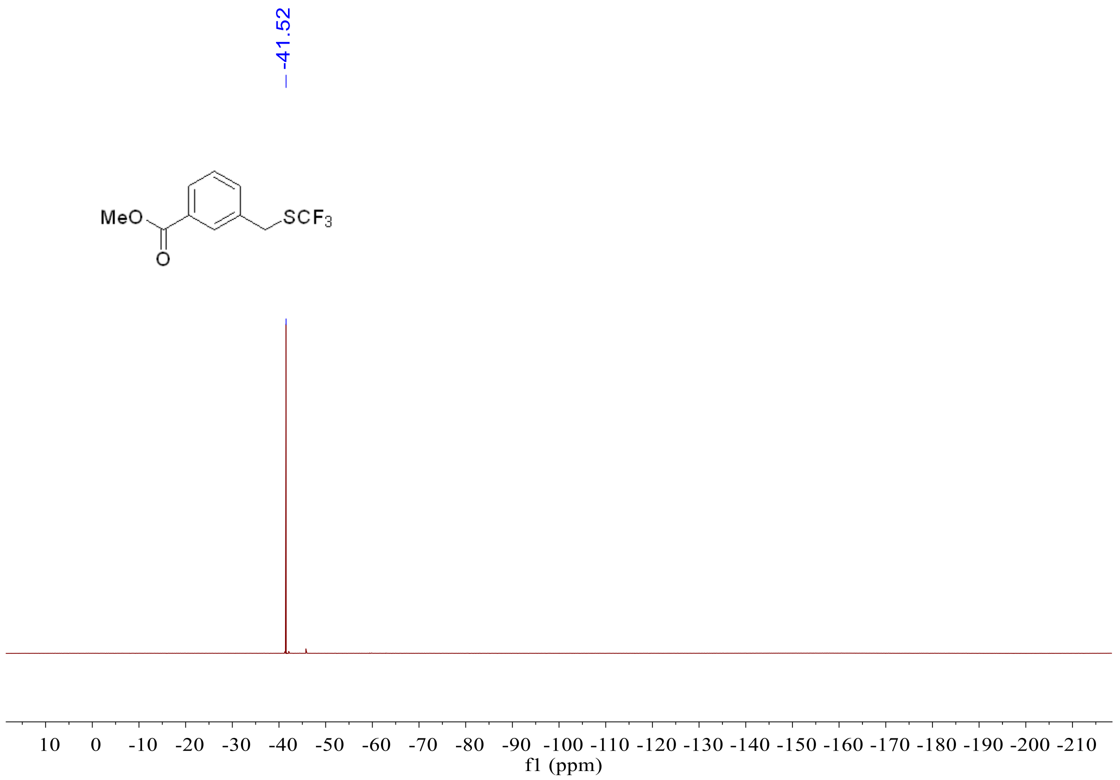

Supplementary Figure 71. ^{19}F NMR spectra for compound **3m**

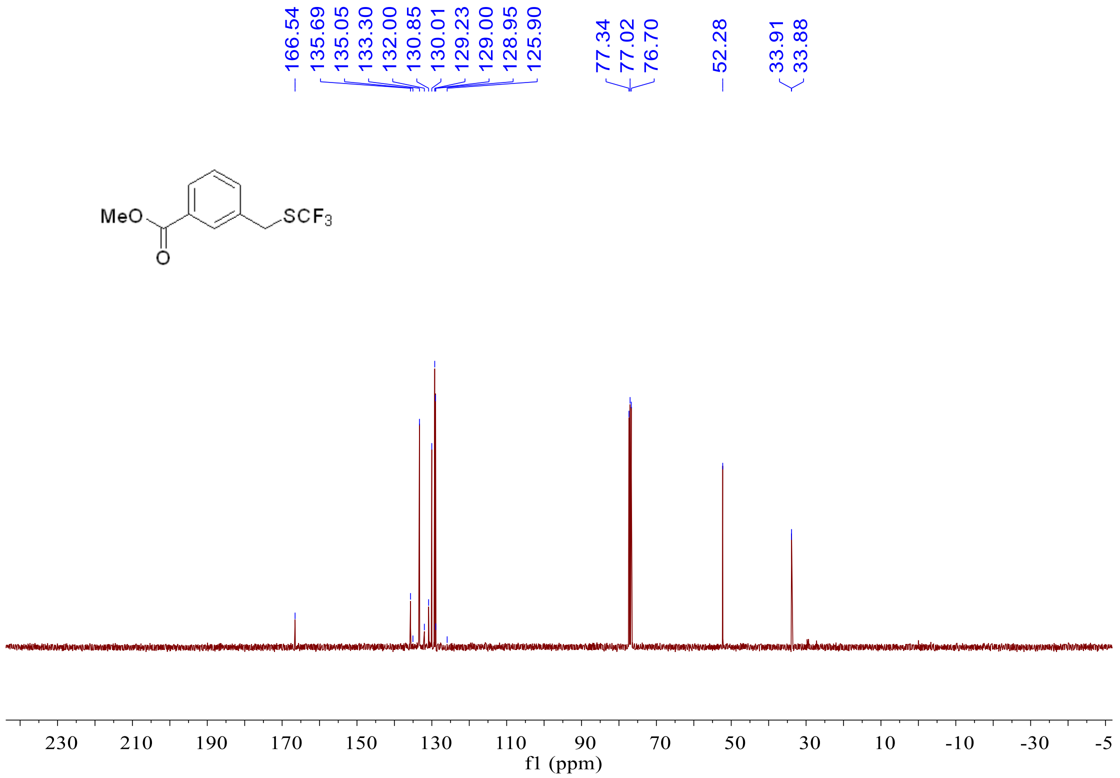

Supplementary Figure 72. ^{13}C NMR spectra for compound **3m**

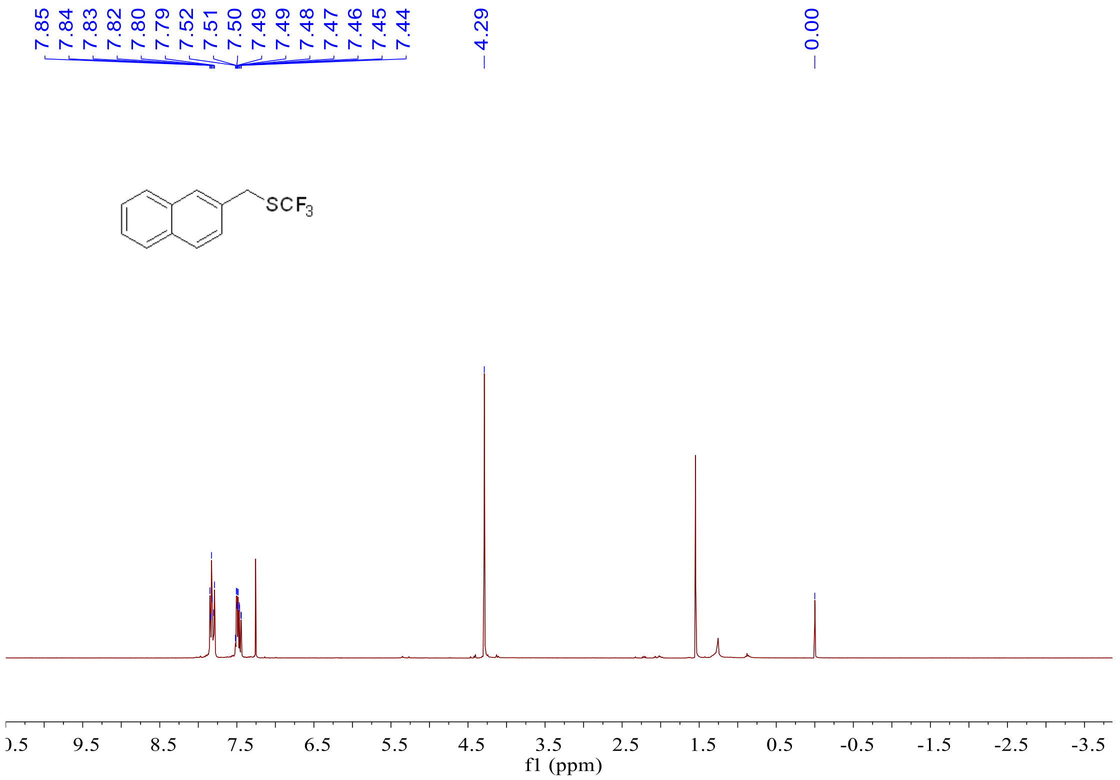

Supplementary Figure 73. ^{1}H NMR spectra for compound **3n**

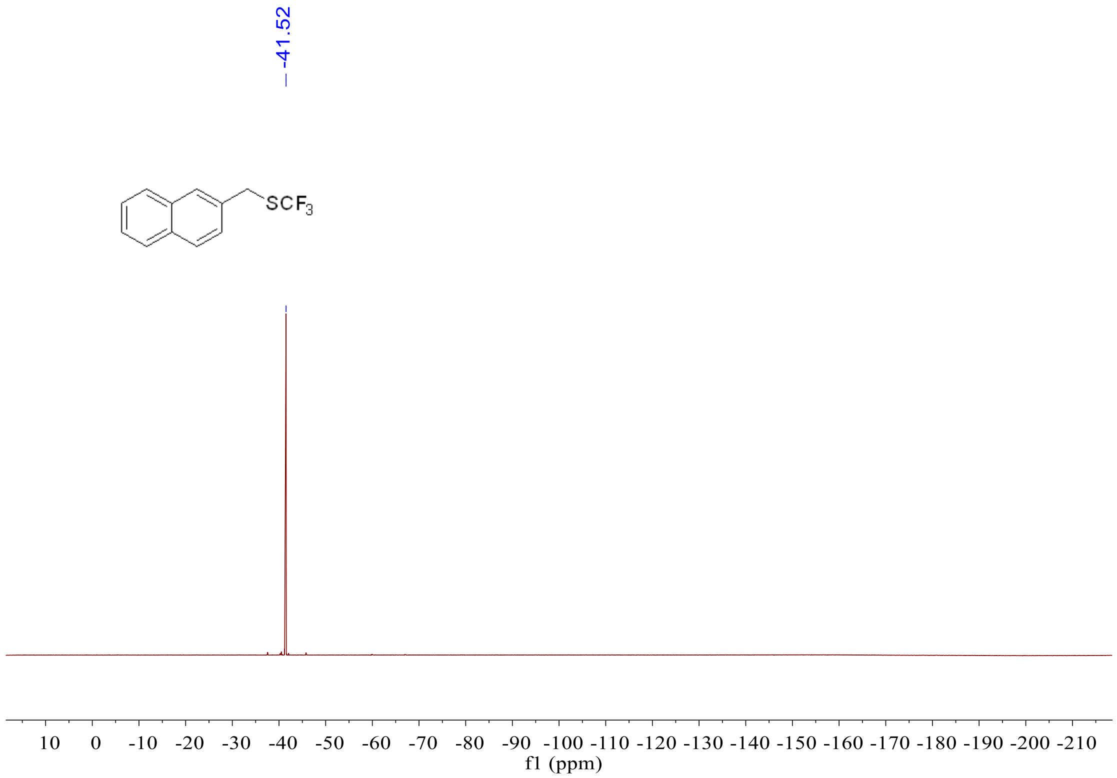

Supplementary Figure 74. ^{19}F NMR spectra for compound **3n**

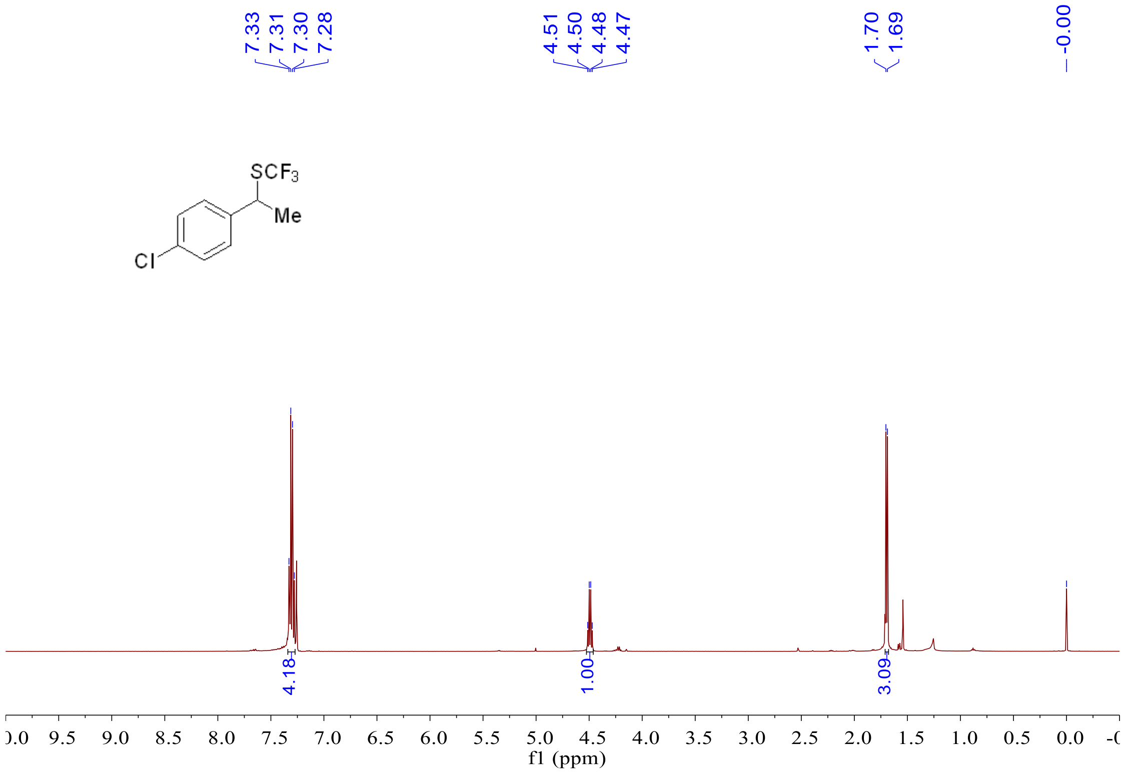

Supplementary Figure 75. ^{1}H NMR spectra for compound **3o**

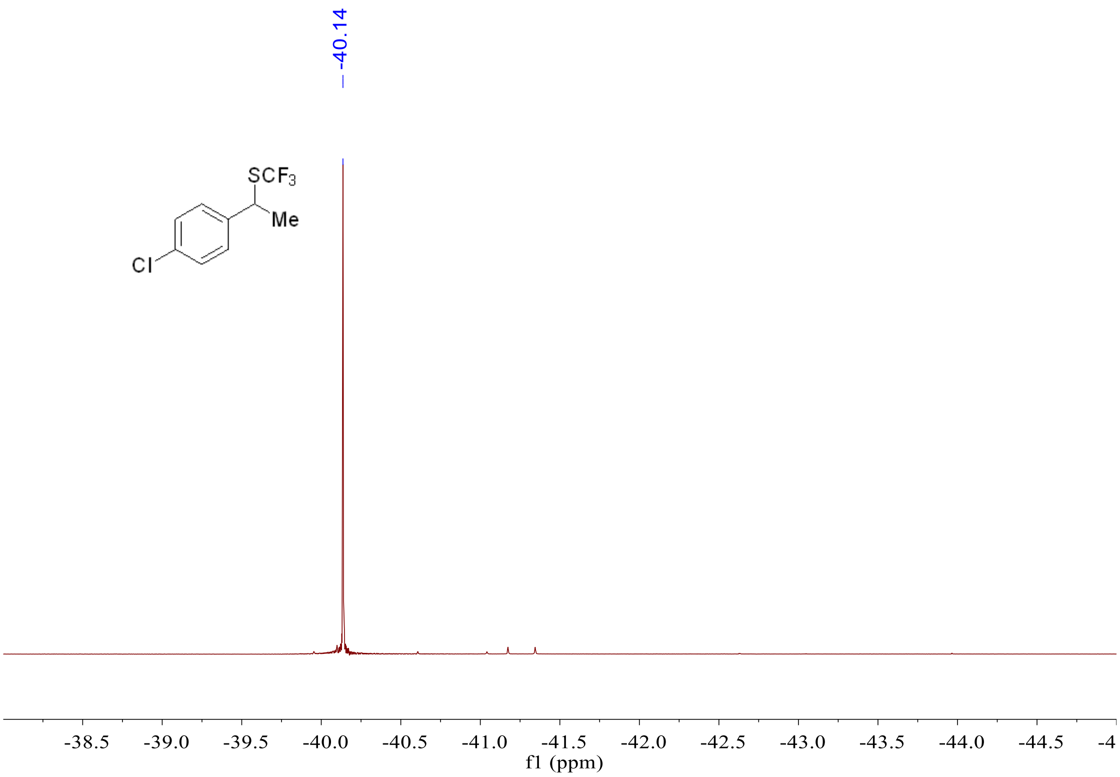

Supplementary Figure 76. ^{19}F NMR spectra for compound **3o**

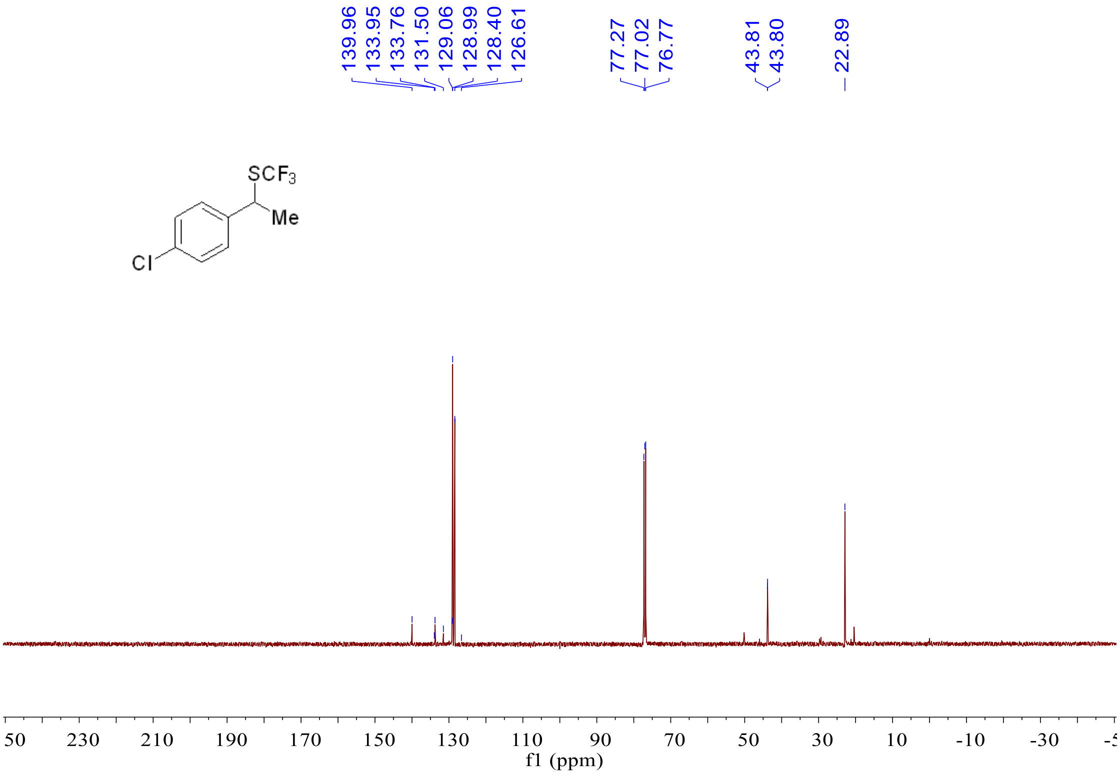

Supplementary Figure 77. ^{13}C NMR spectra for compound **3o**

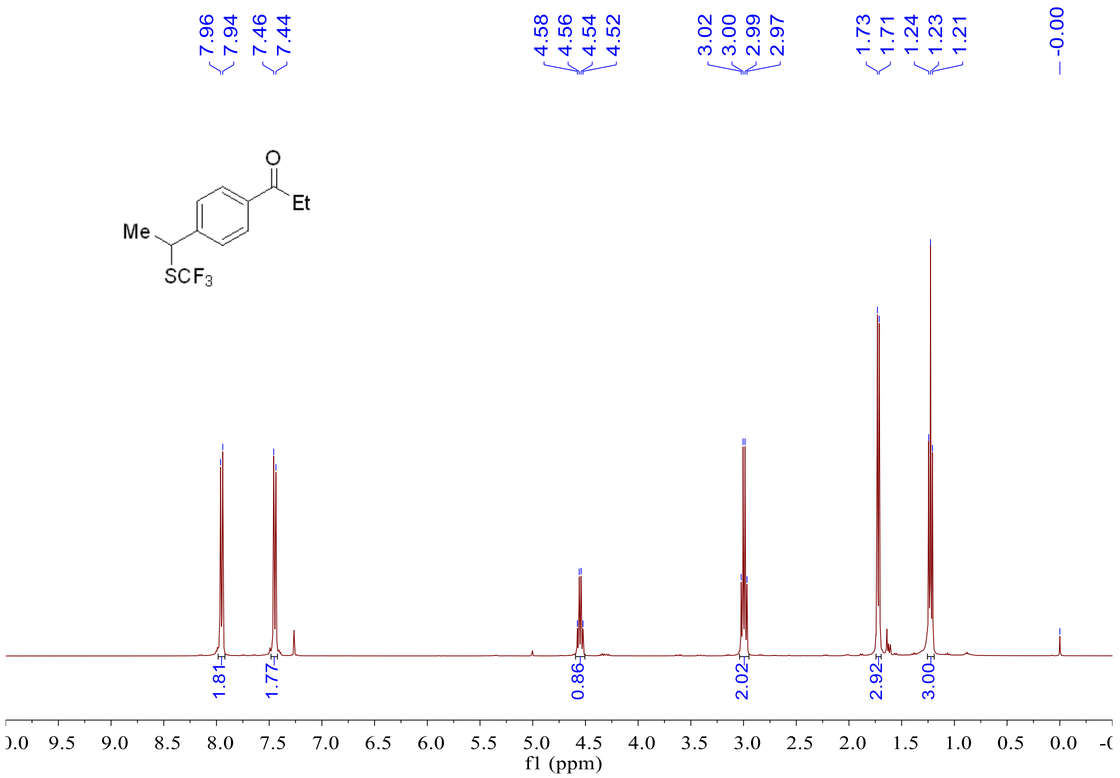

Supplementary Figure 78. ^{1}H NMR spectra for compound **3p**

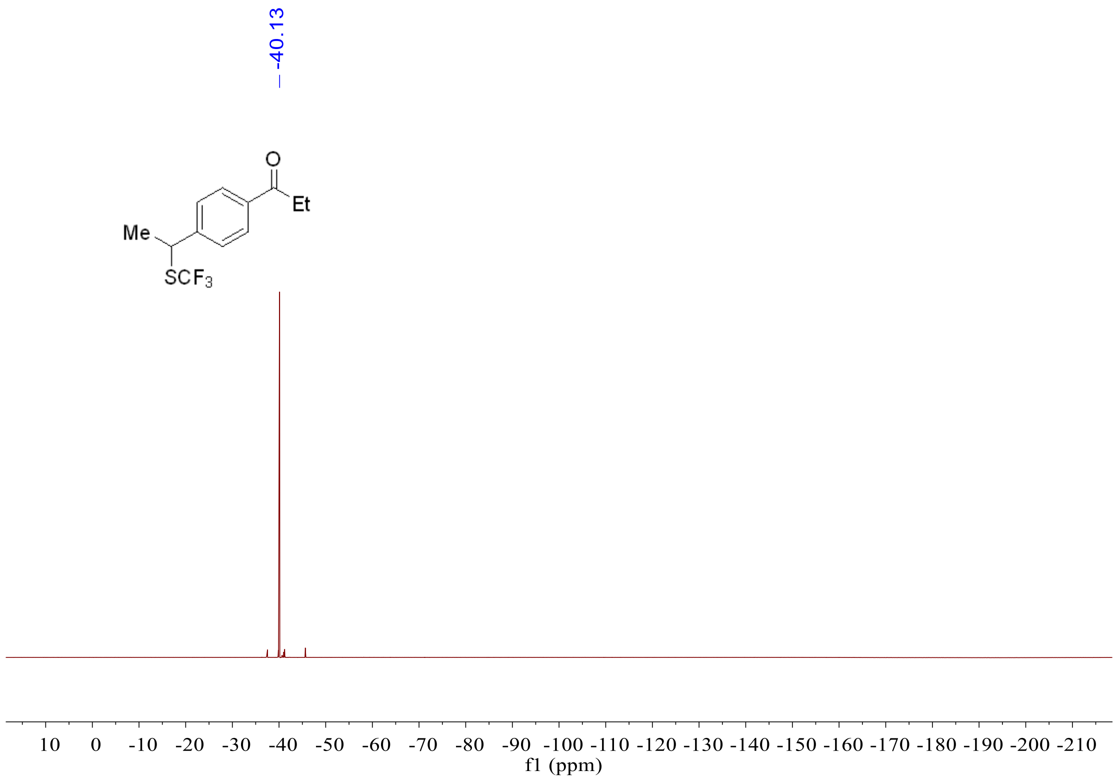

Supplementary Figure 79. ^{19}F NMR spectra for compound **3p**

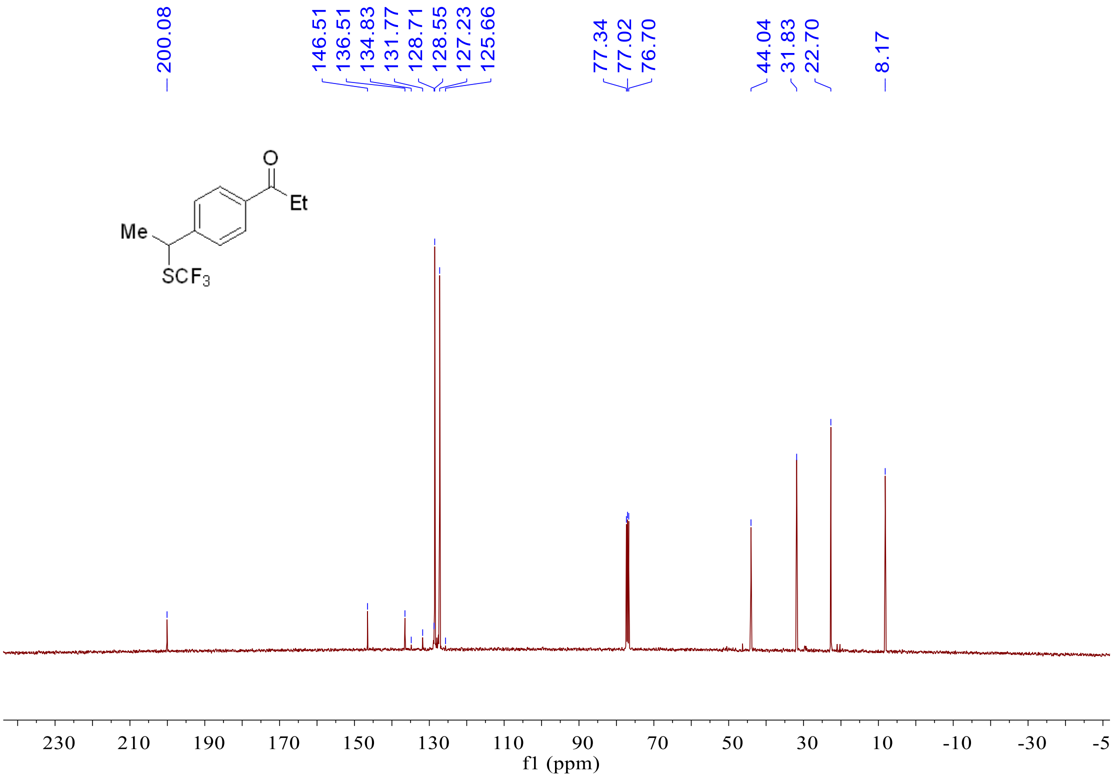

Supplementary Figure 80. ^{13}C NMR spectra for compound **3p**

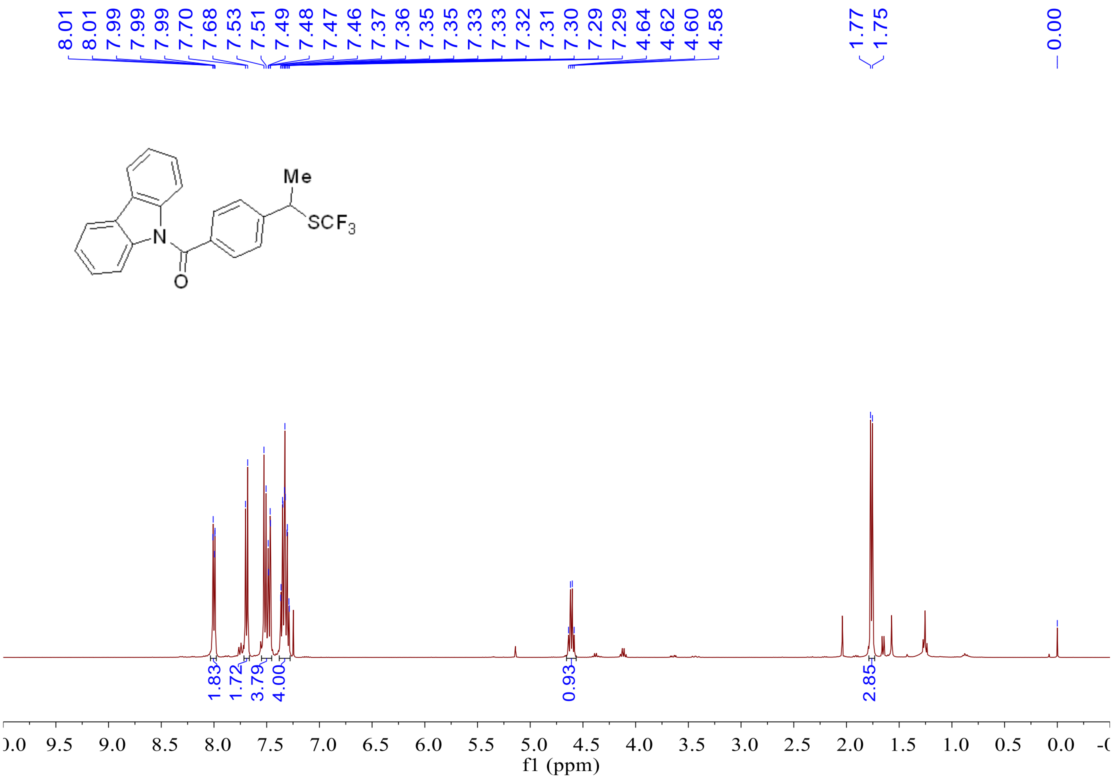

Supplementary Figure 81. ^{1}H NMR spectra for compound **3q**

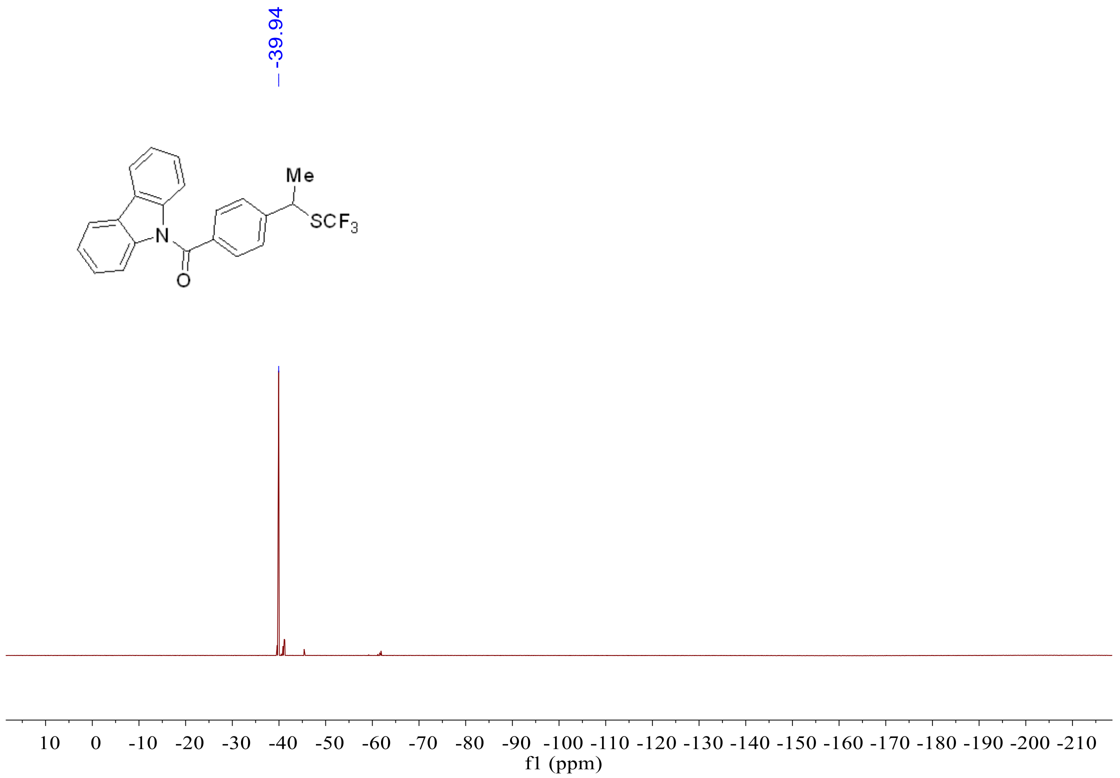

Supplementary Figure 82. ^{19}F NMR spectra for compound **3q**

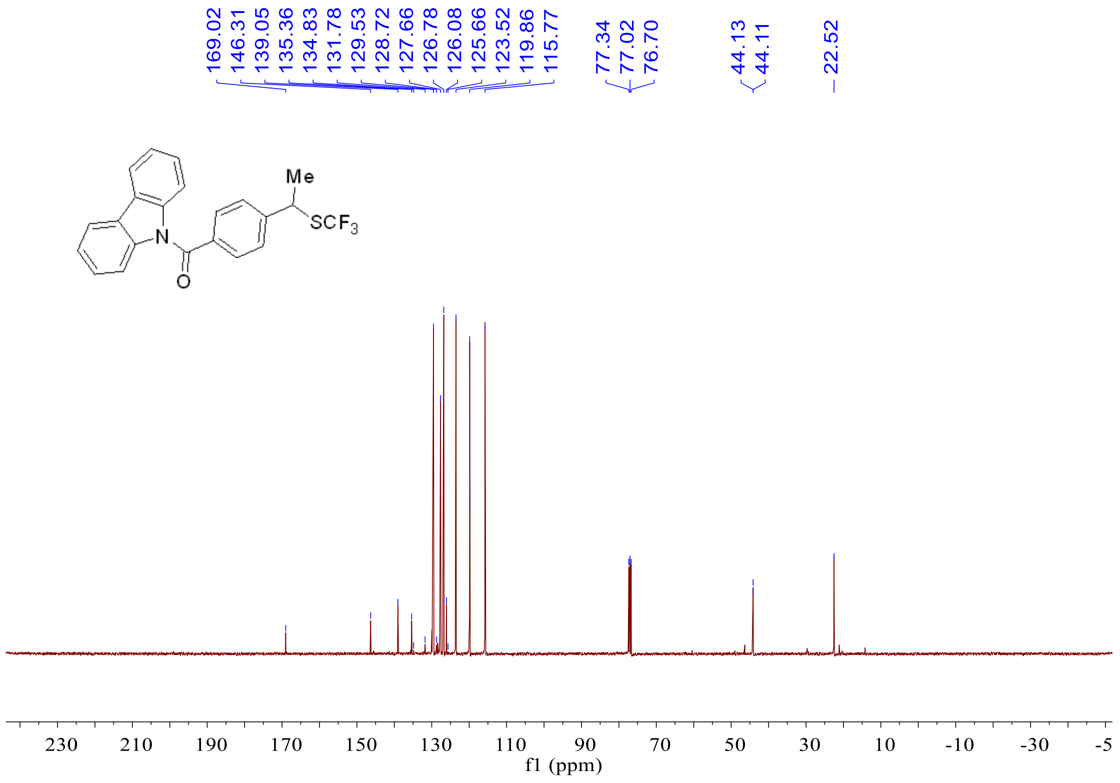

Supplementary Figure 83. ^{13}C NMR spectra for compound **3q**

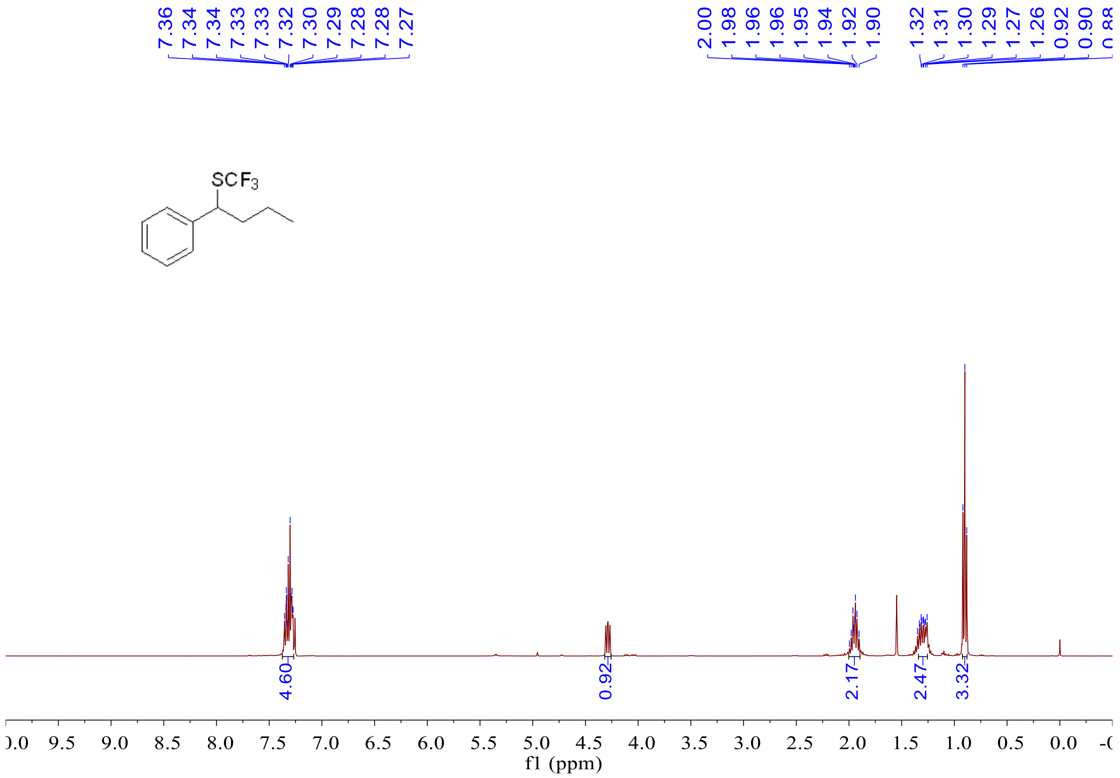

Supplementary Figure 84. ^{1}H NMR spectra for compound **3r**

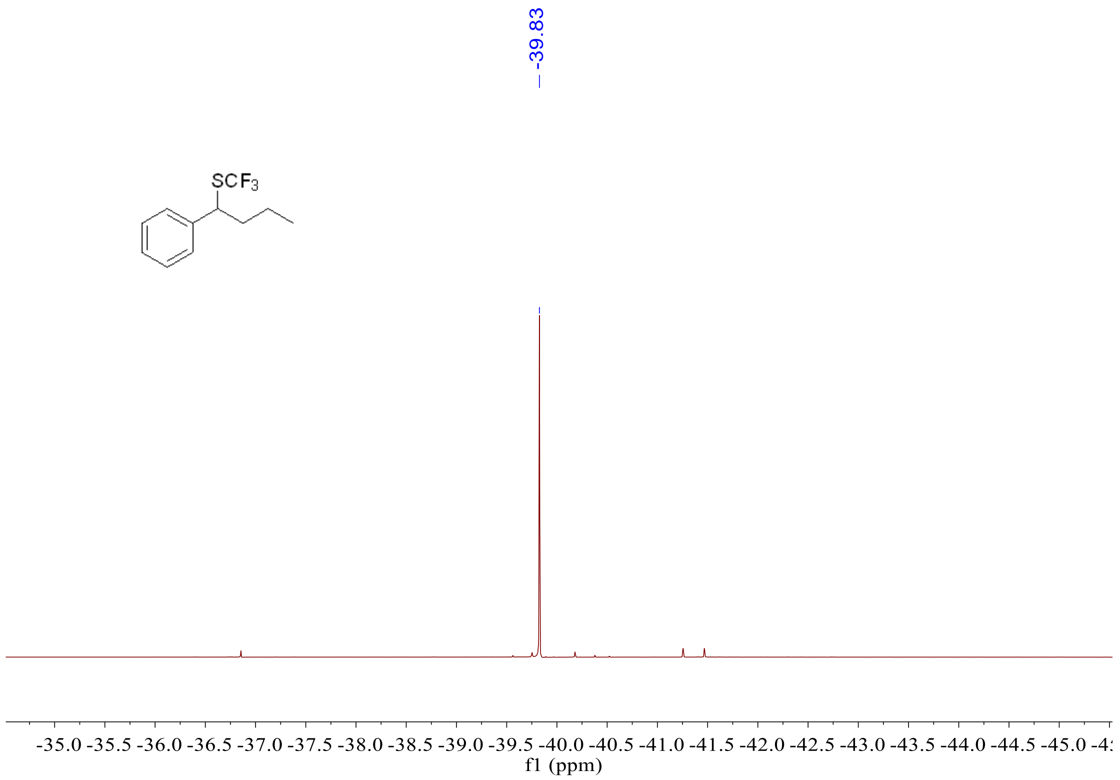

Supplementary Figure 85. ^{19}F NMR spectra for compound **3r**

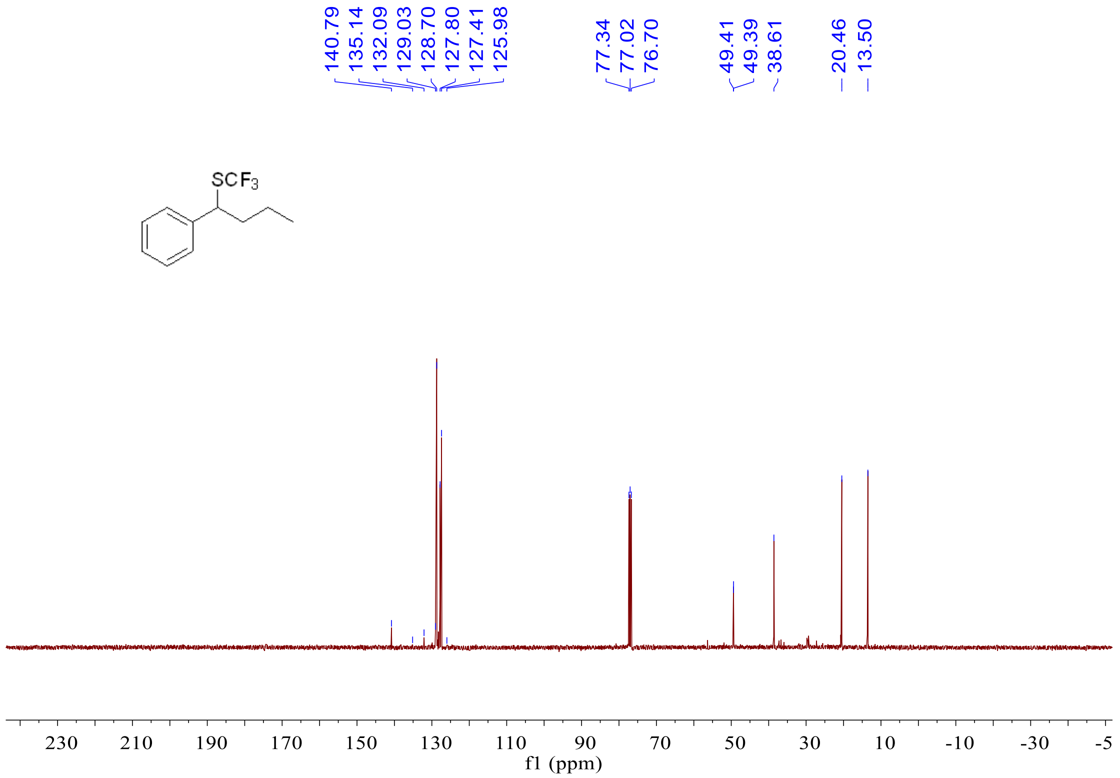

Supplementary Figure 86. ^{13}C NMR spectra for compound **3r**

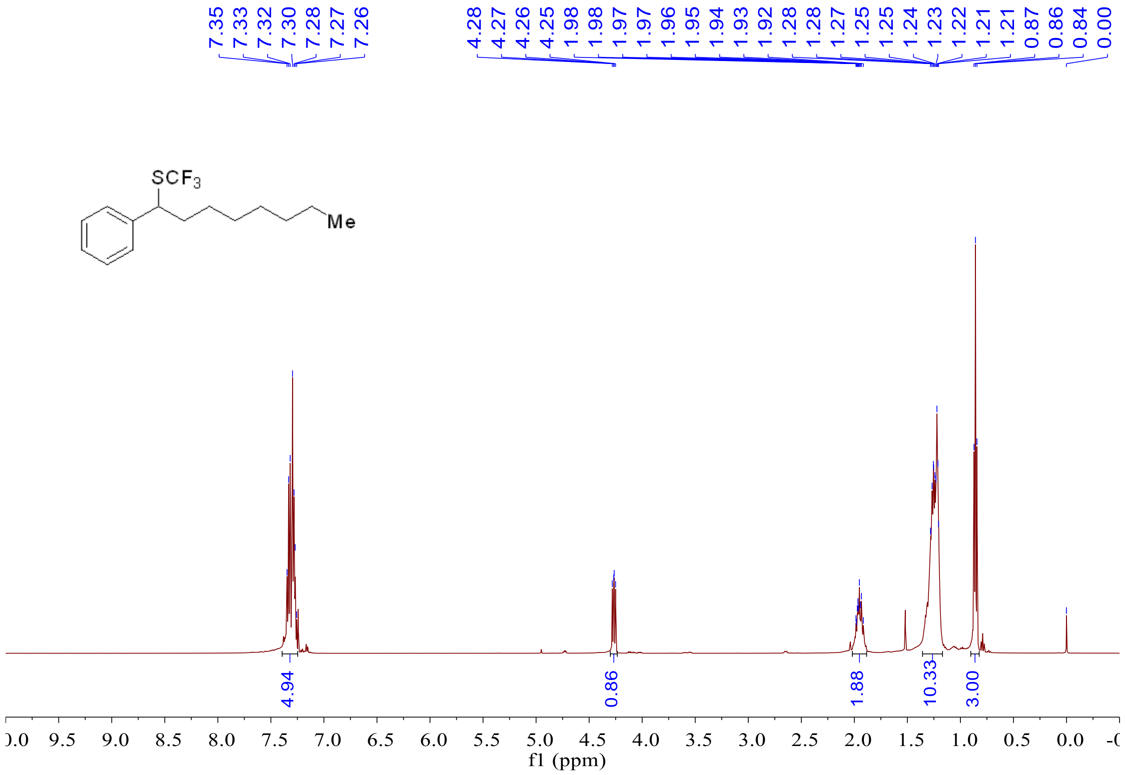

Supplementary Figure 87. ^{1}H NMR spectra for compound **3s**

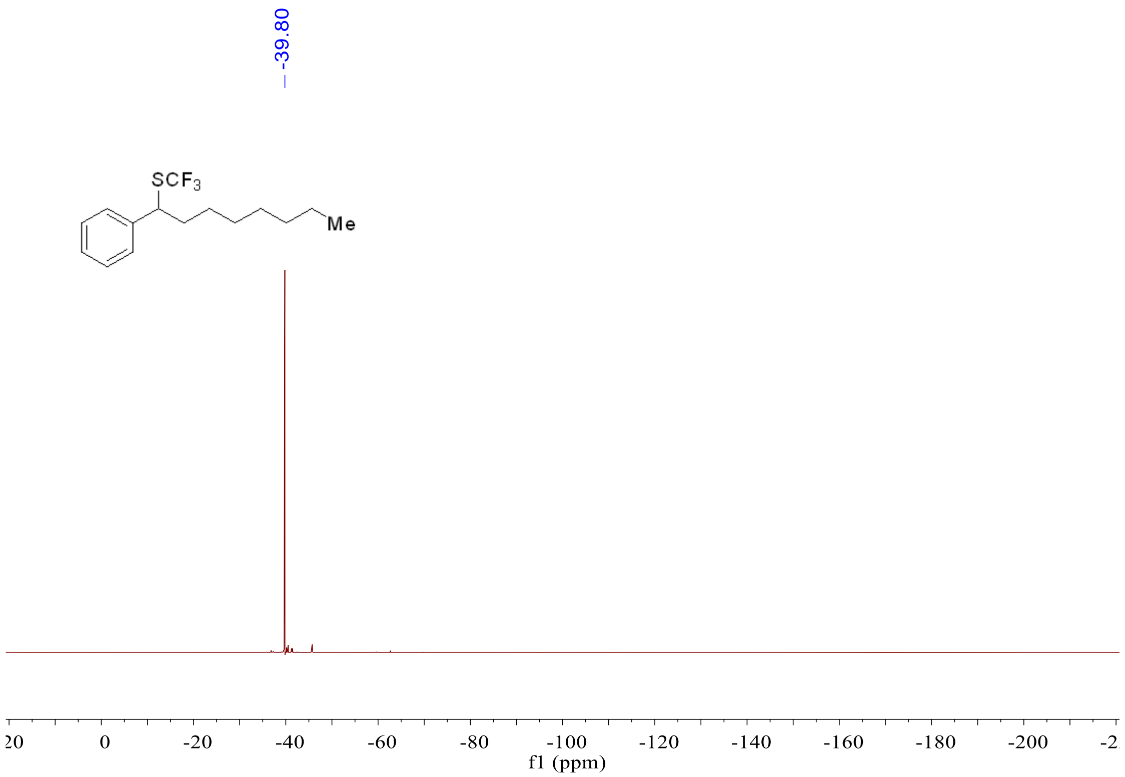

Supplementary Figure 88. ^{19}F NMR spectra for compound **3s**

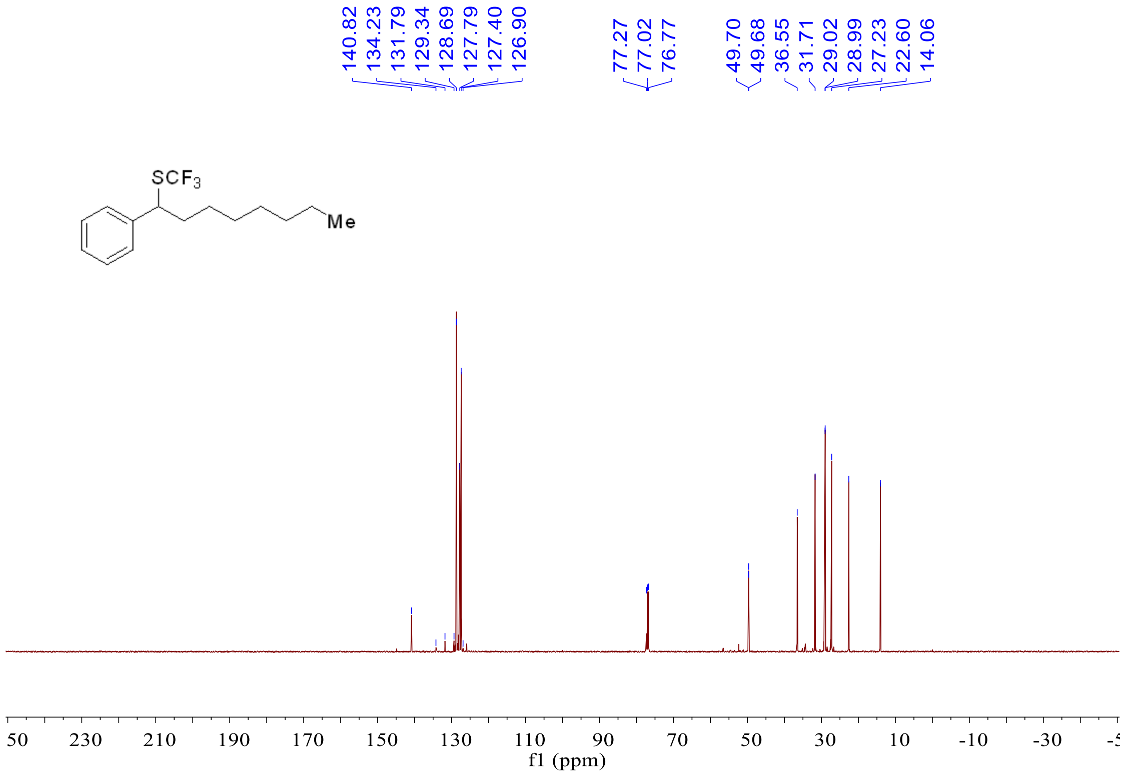

Supplementary Figure 89. ^{13}C NMR spectra for compound **3s**

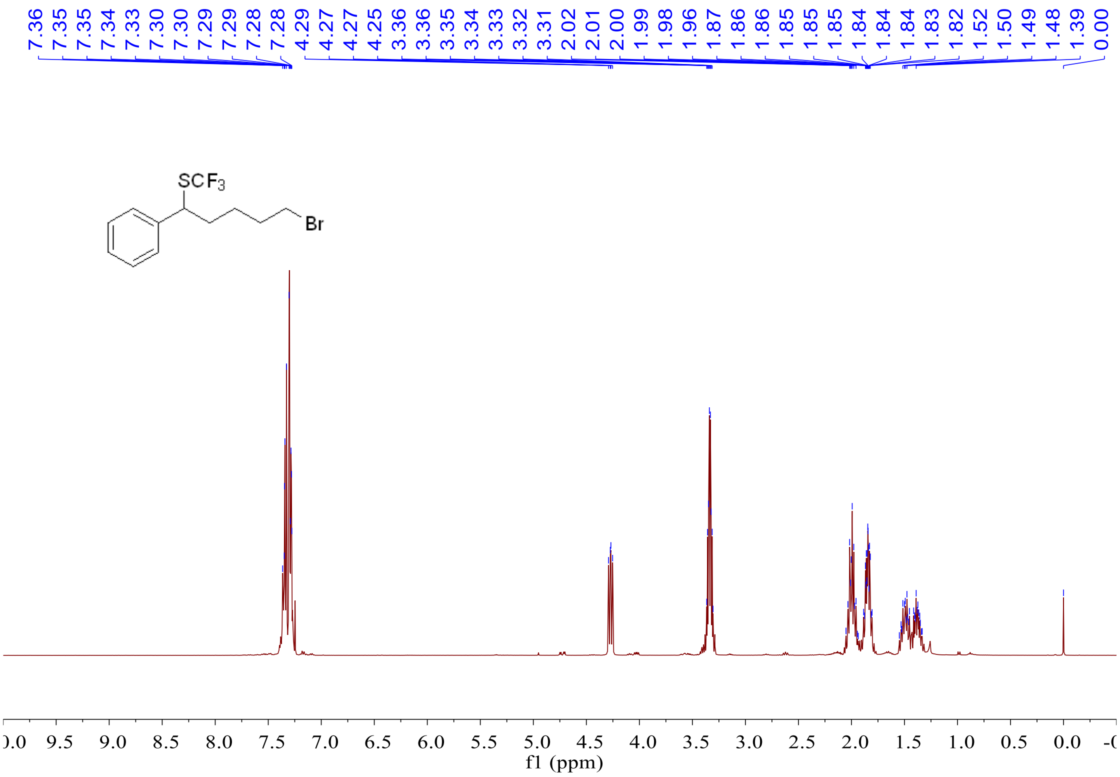

Supplementary Figure 90. ^{1}H NMR spectra for compound **3t**

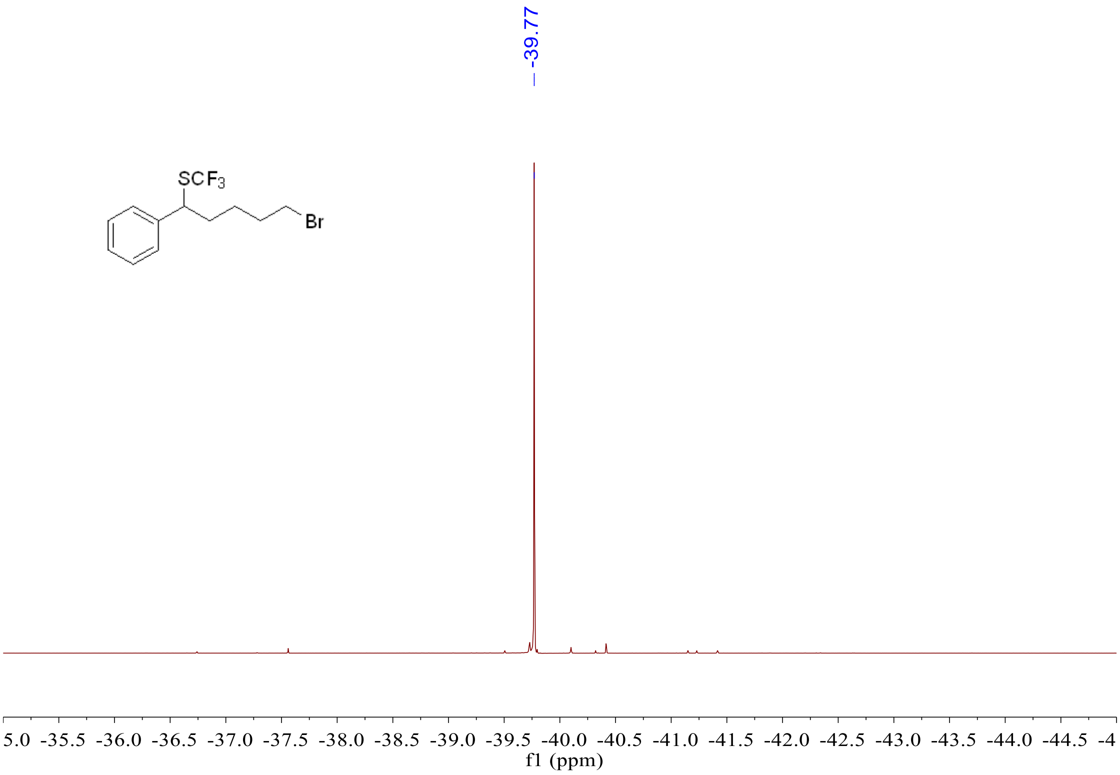

Supplementary Figure 91. ^{19}F NMR spectra for compound **3t**

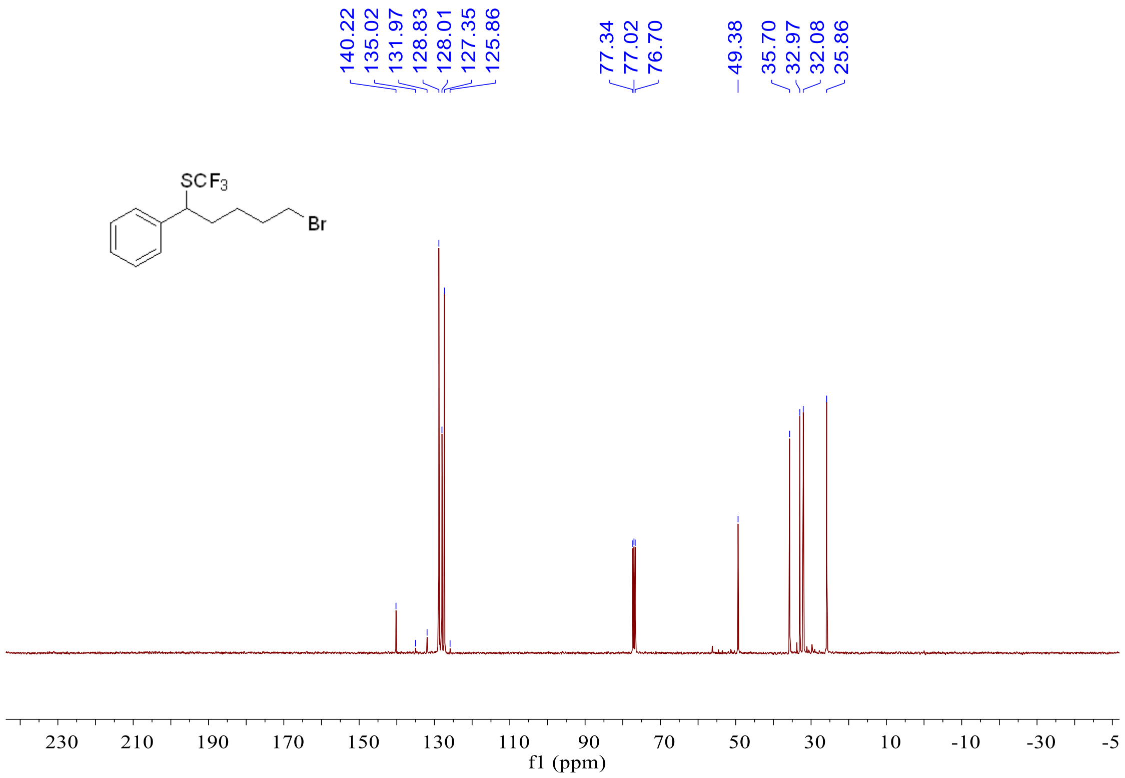

Supplementary Figure 92. ^{13}C NMR spectra for compound **3t**

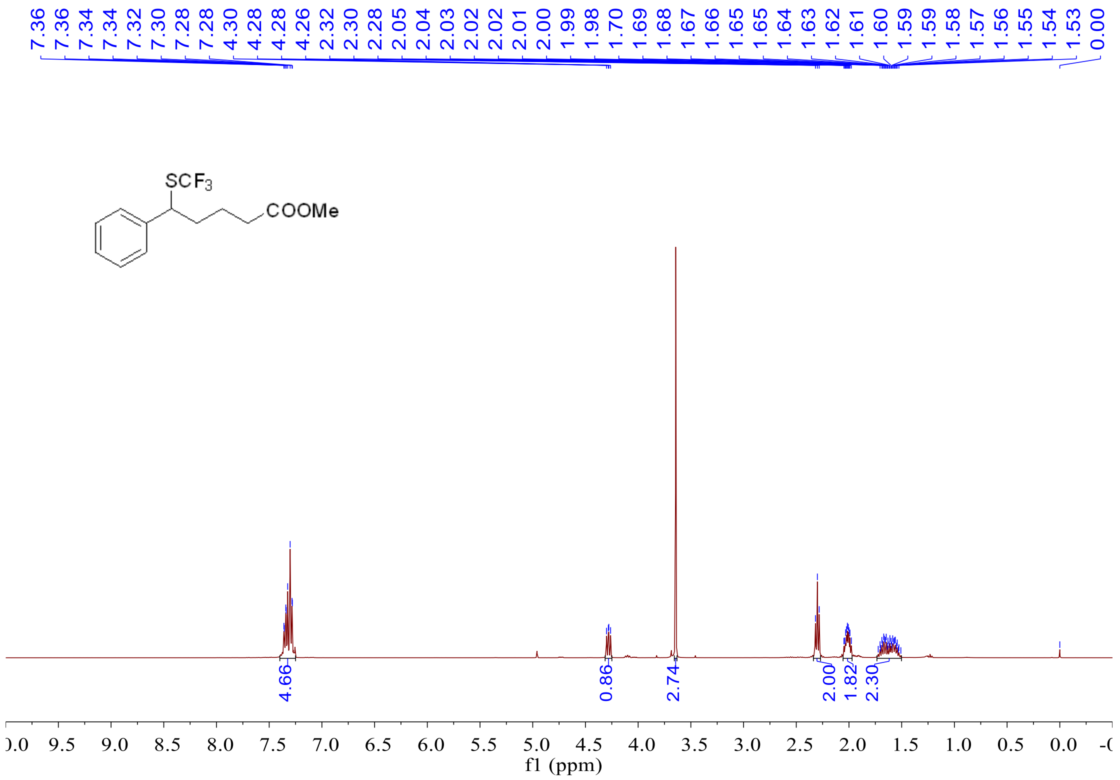

Supplementary Figure 93. ^{1}H NMR spectra for compound **3u**

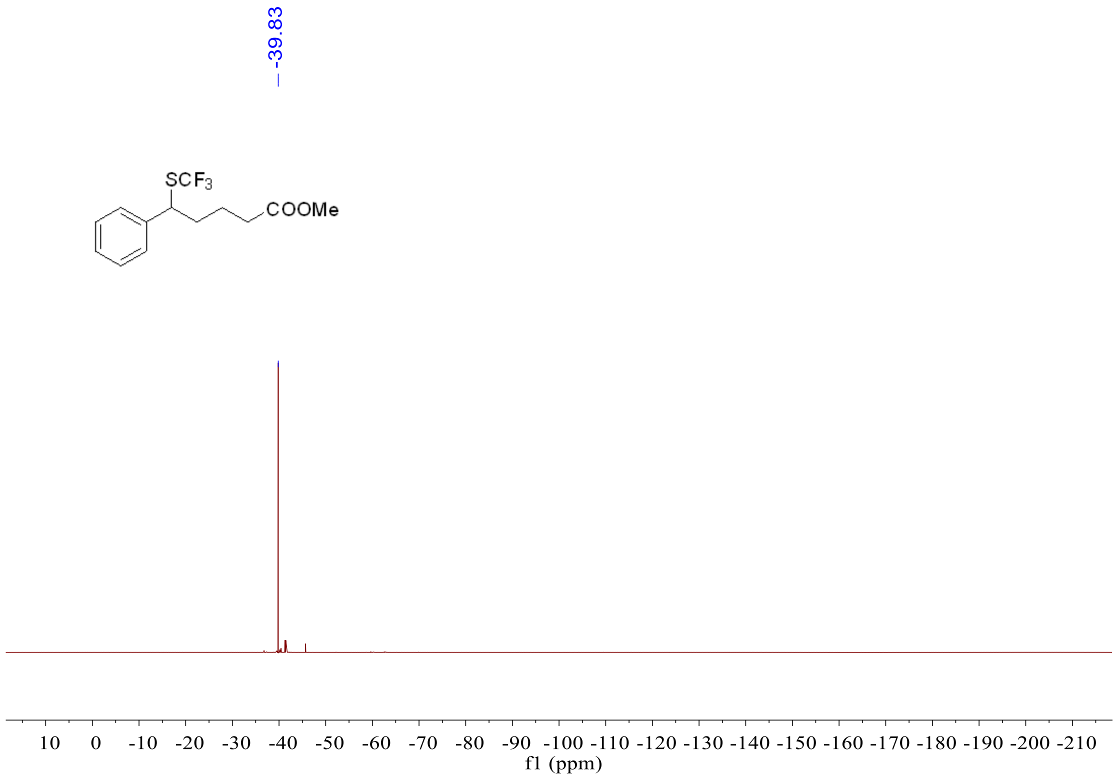

Supplementary Figure 94. ^{19}F NMR spectra for compound **3u**

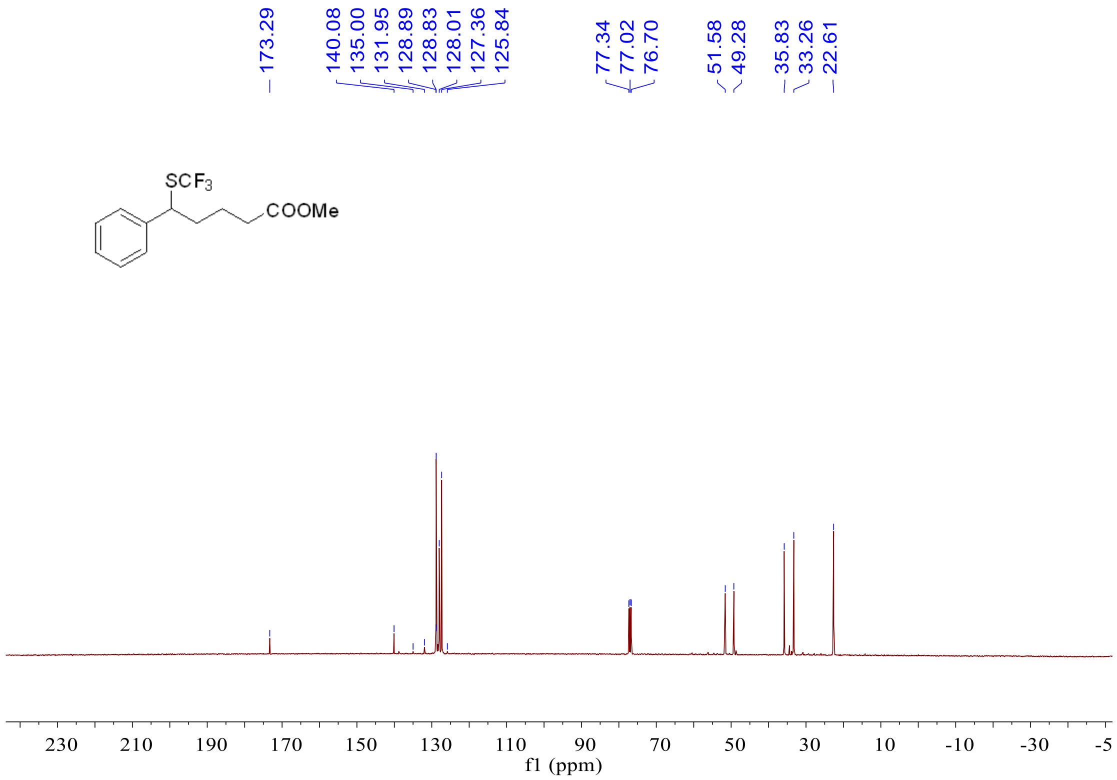

Supplementary Figure 95. ^{13}C NMR spectra for compound **3u**

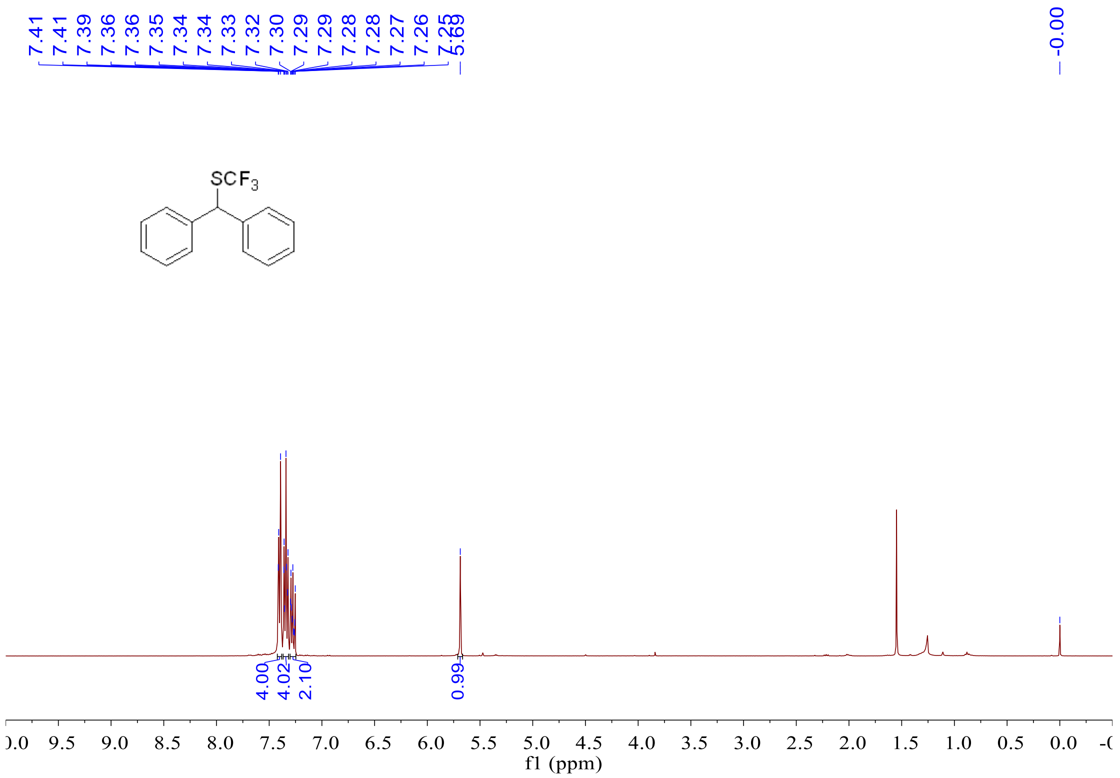

Supplementary Figure 96. ^{1}H NMR spectra for compound **3v**

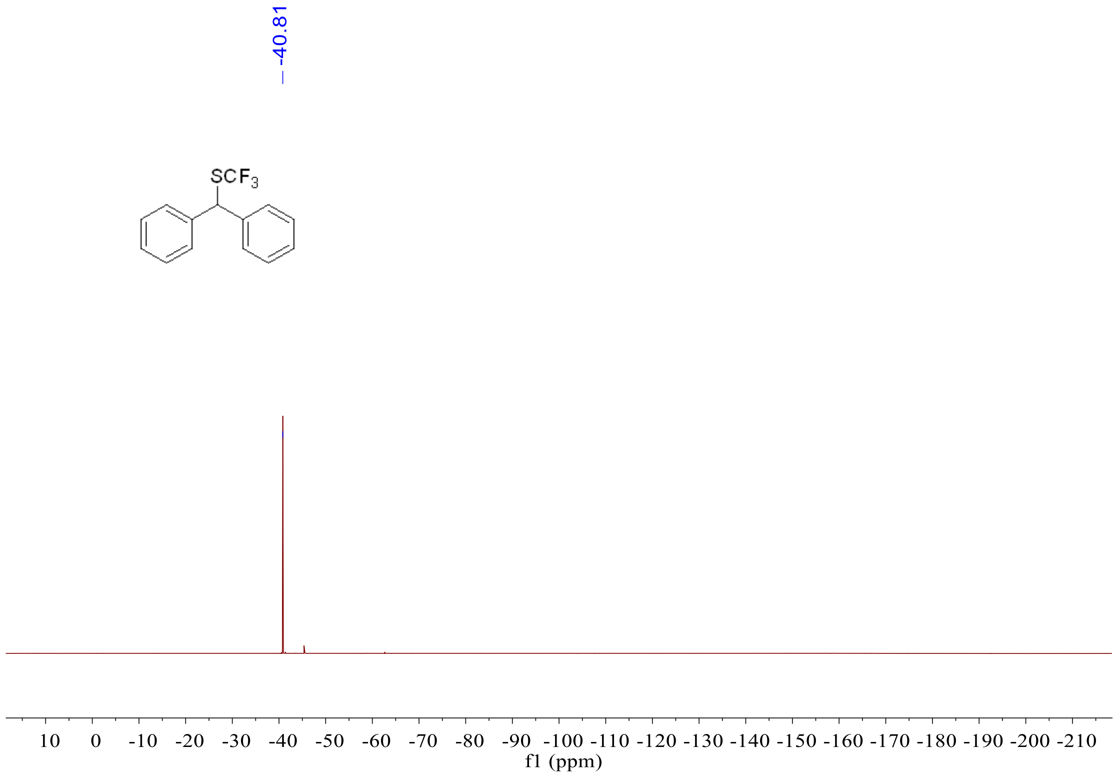

Supplementary Figure 97. ^{19}F NMR spectra for compound **3v**

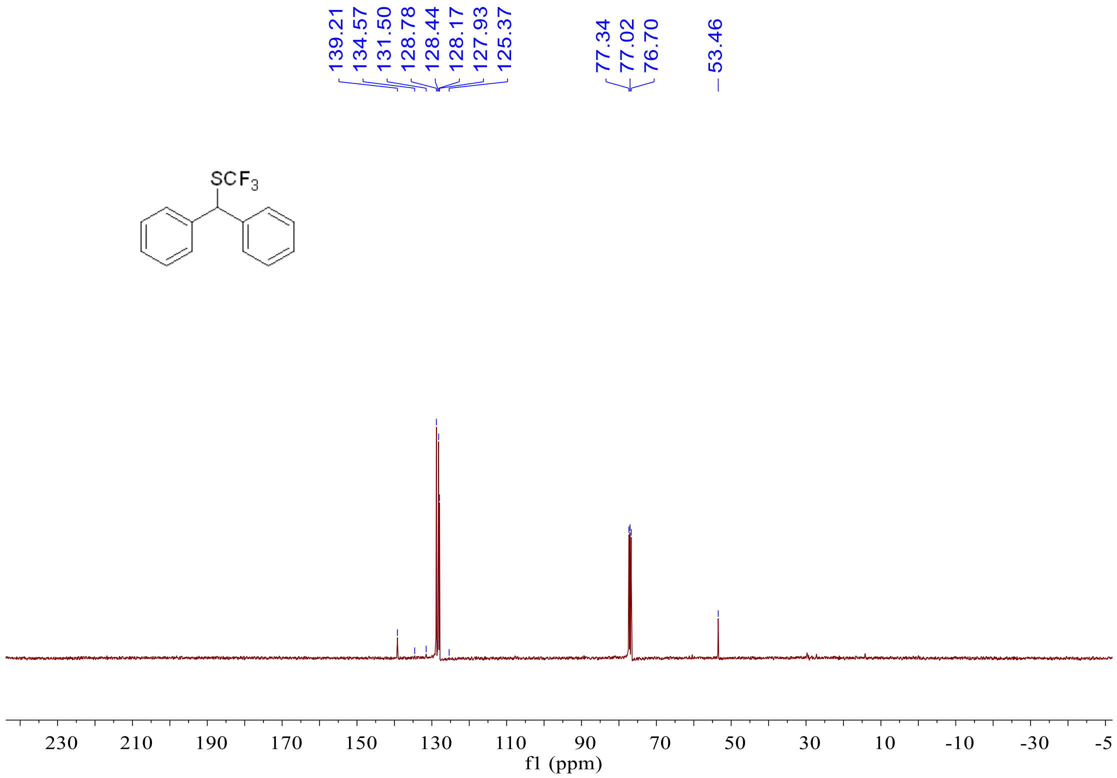

Supplementary Figure 98. ^{13}C NMR spectra for compound **3v**

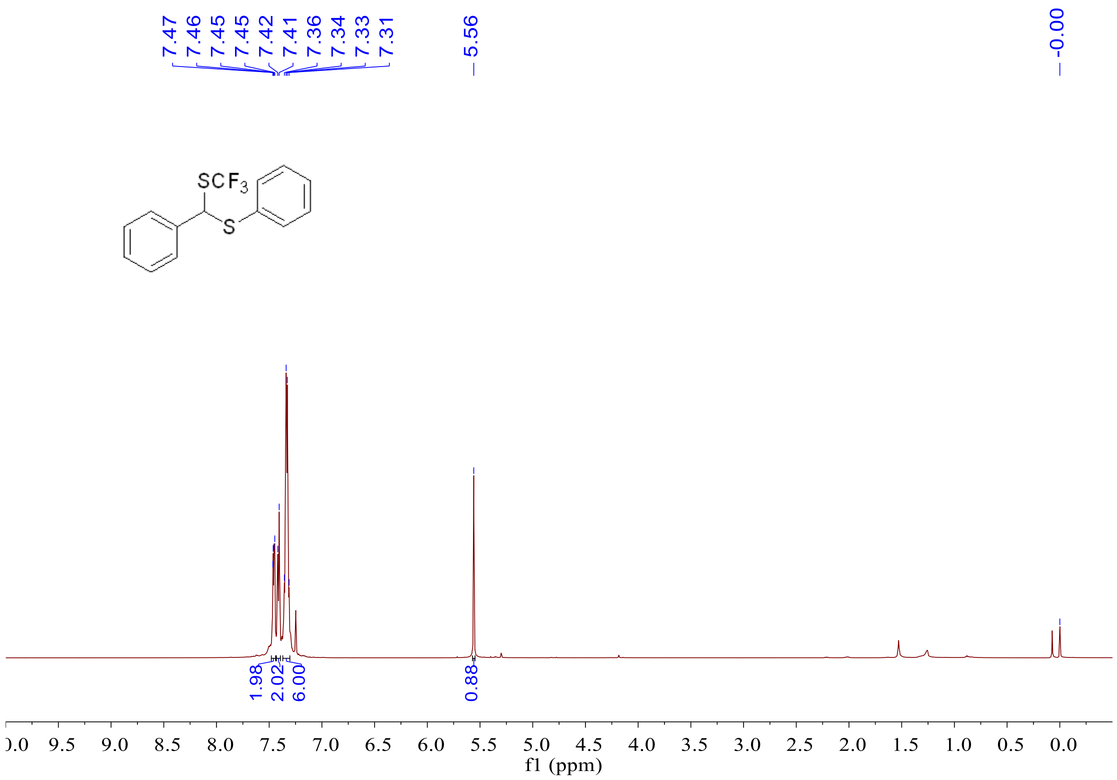

Supplementary Figure 99. ^{1}H NMR spectra for compound **3w**

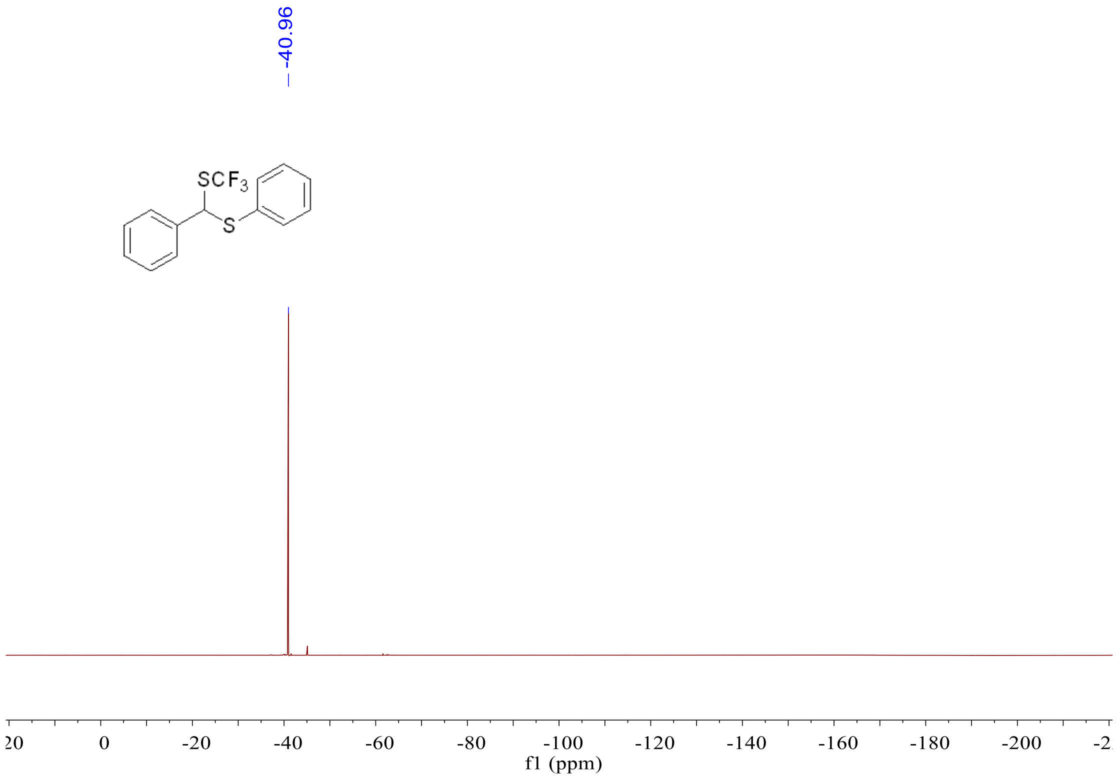

Supplementary Figure 100. ^{19}F NMR spectra for compound **3w**

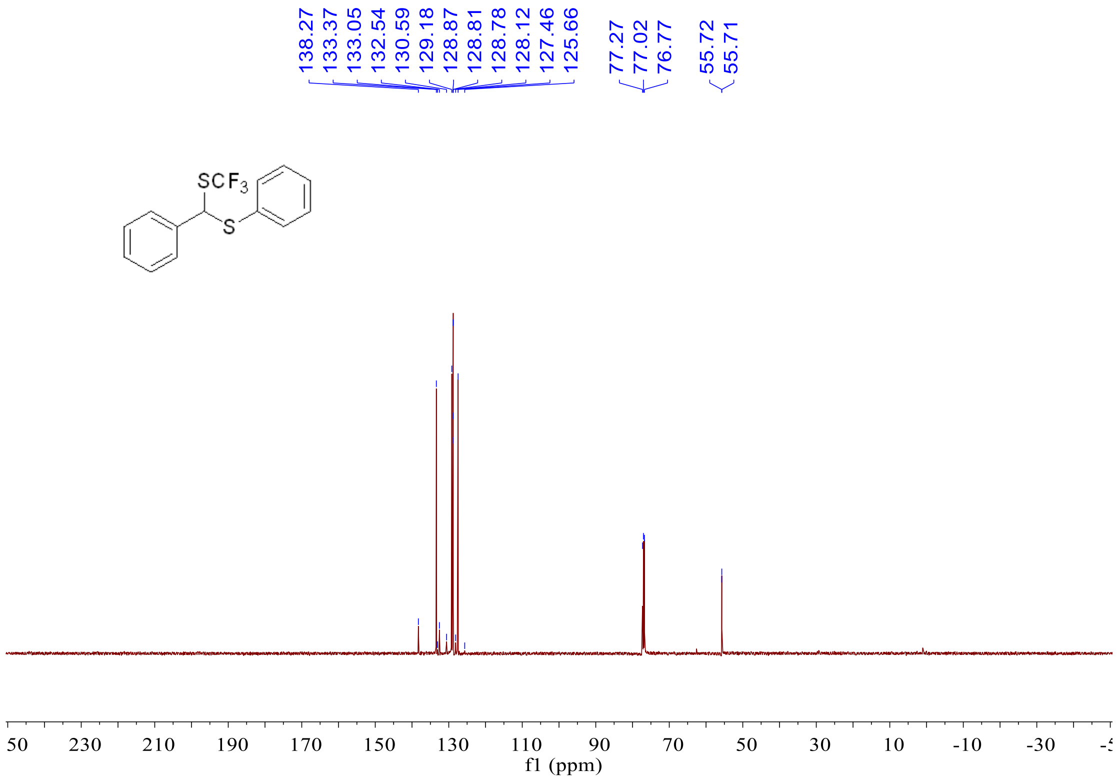

Supplementary Figure 101. ^{13}C NMR spectra for compound **3w**

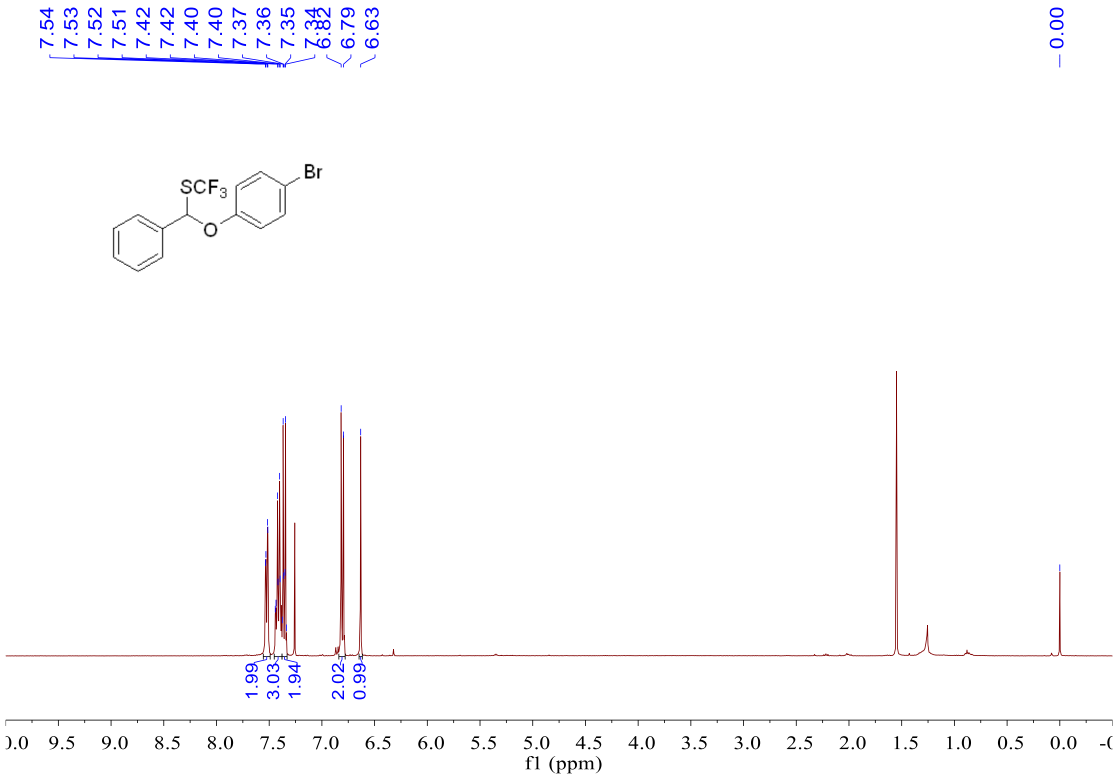

Supplementary Figure 102. ^{1}H NMR spectra for compound **3x**

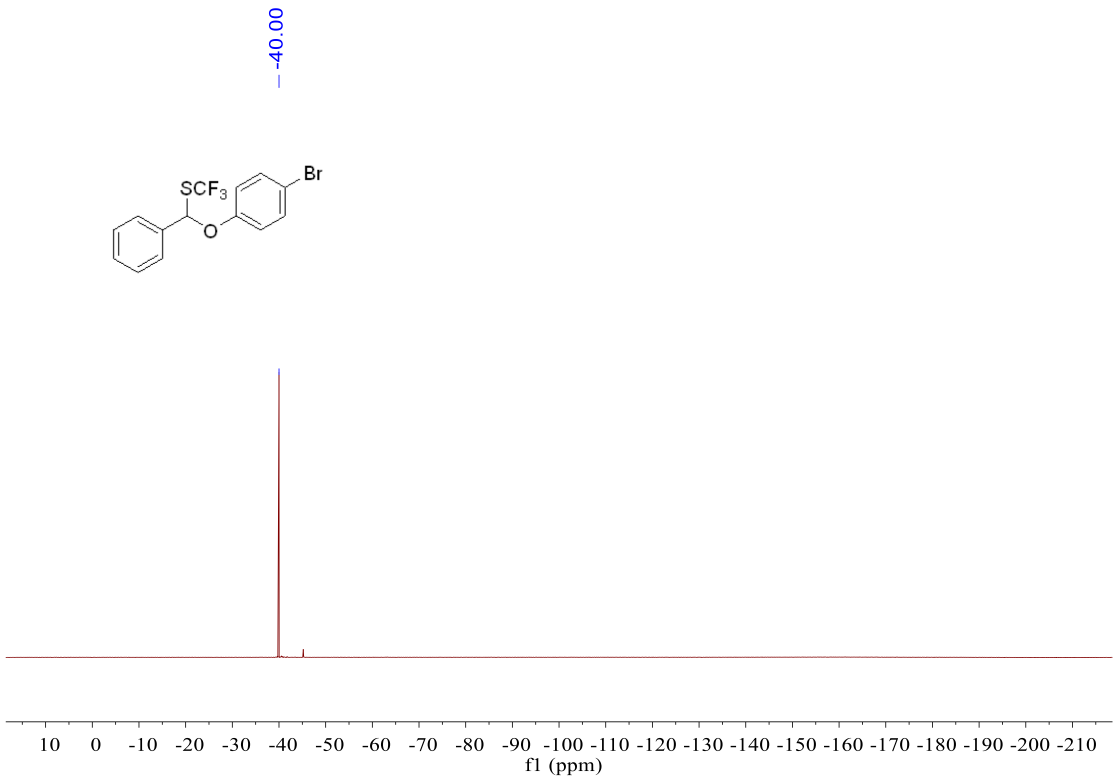

Supplementary Figure 103. ^{19}F NMR spectra for compound **3x**

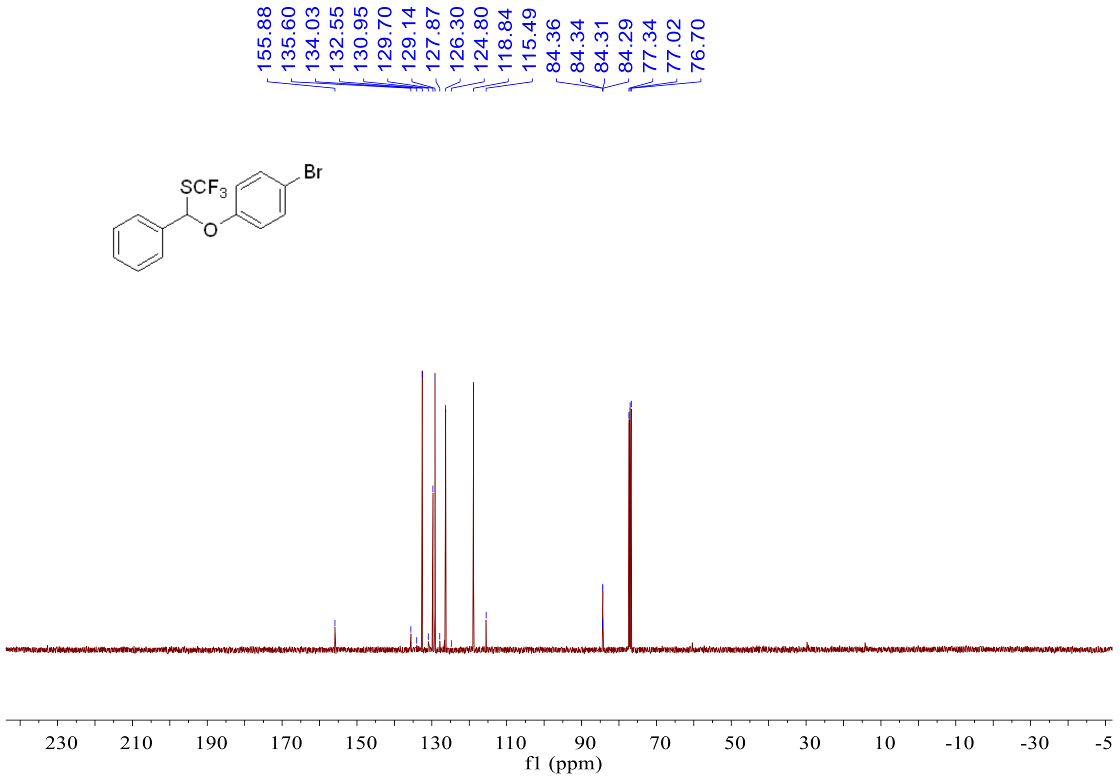

Supplementary Figure 104. ^{13}C NMR spectra for compound **3x**

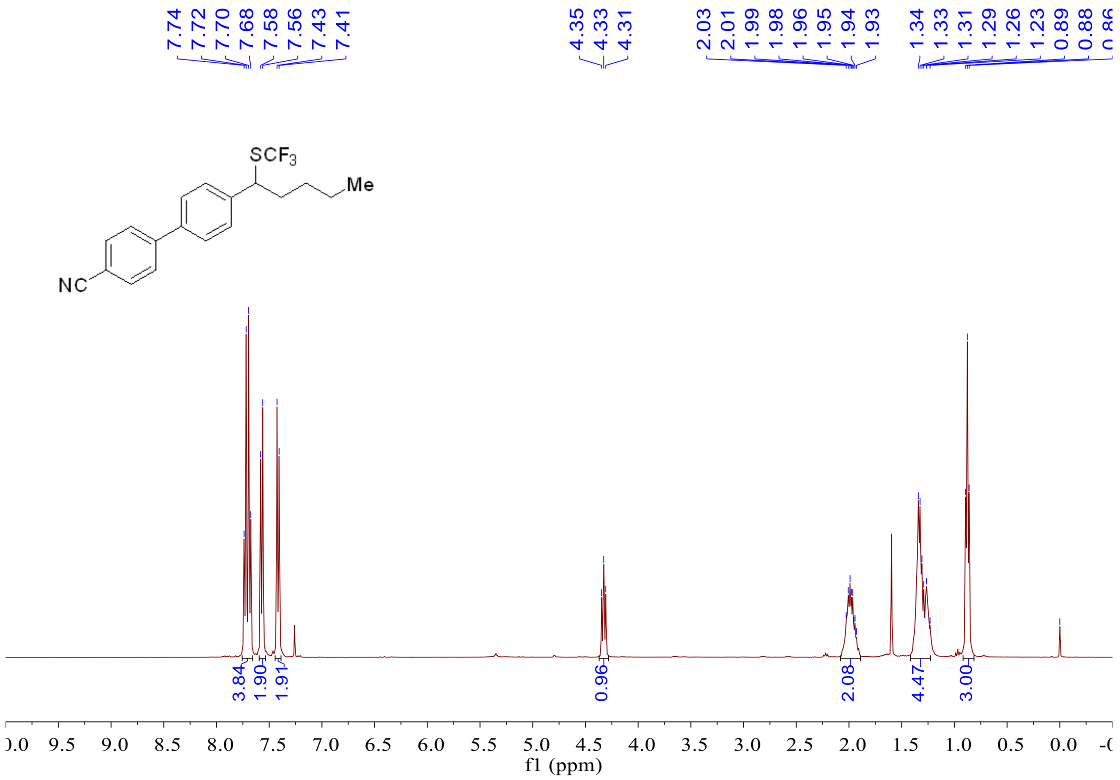

Supplementary Figure 105. ^{1}H NMR spectra for compound **3y**

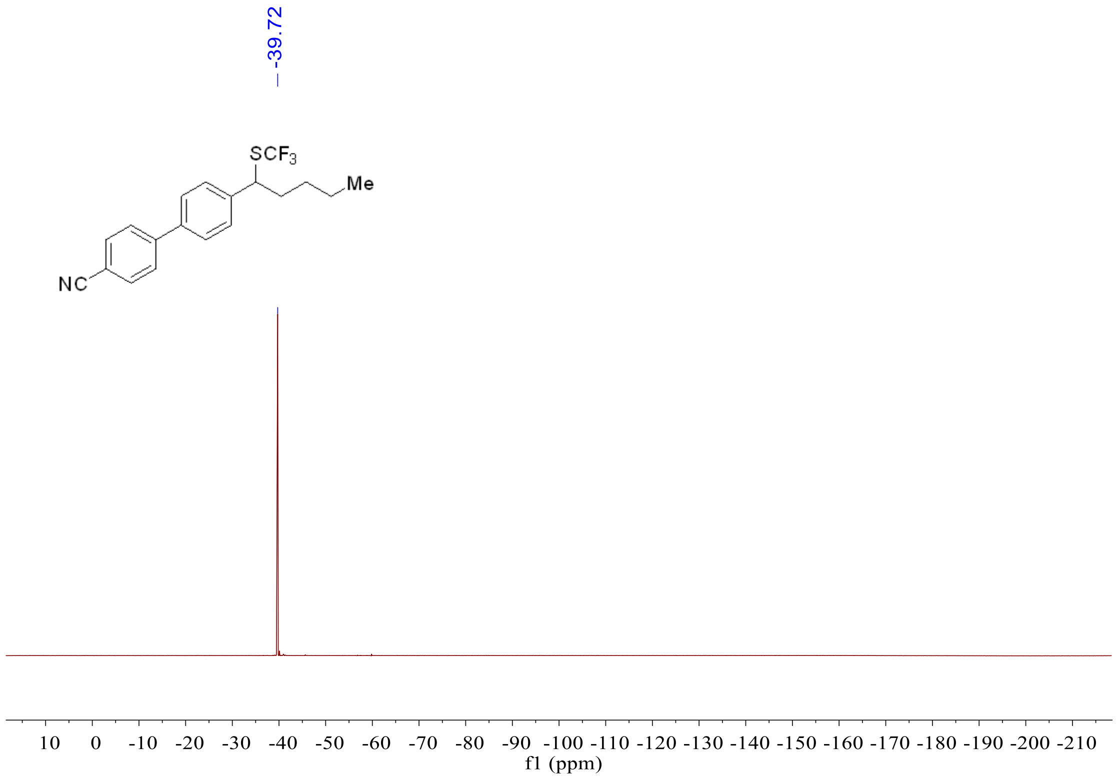

Supplementary Figure 106. ^{19}F NMR spectra for compound **3y**

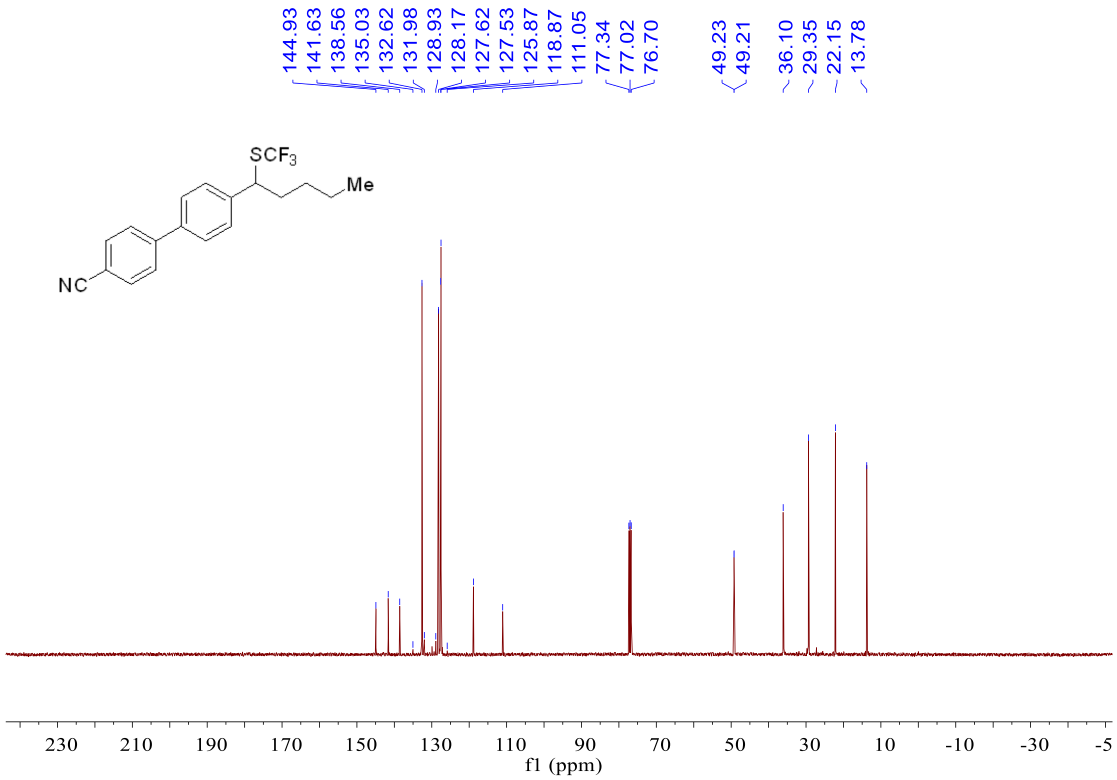

Supplementary Figure 107. ^{13}C NMR spectra for compound **3y**

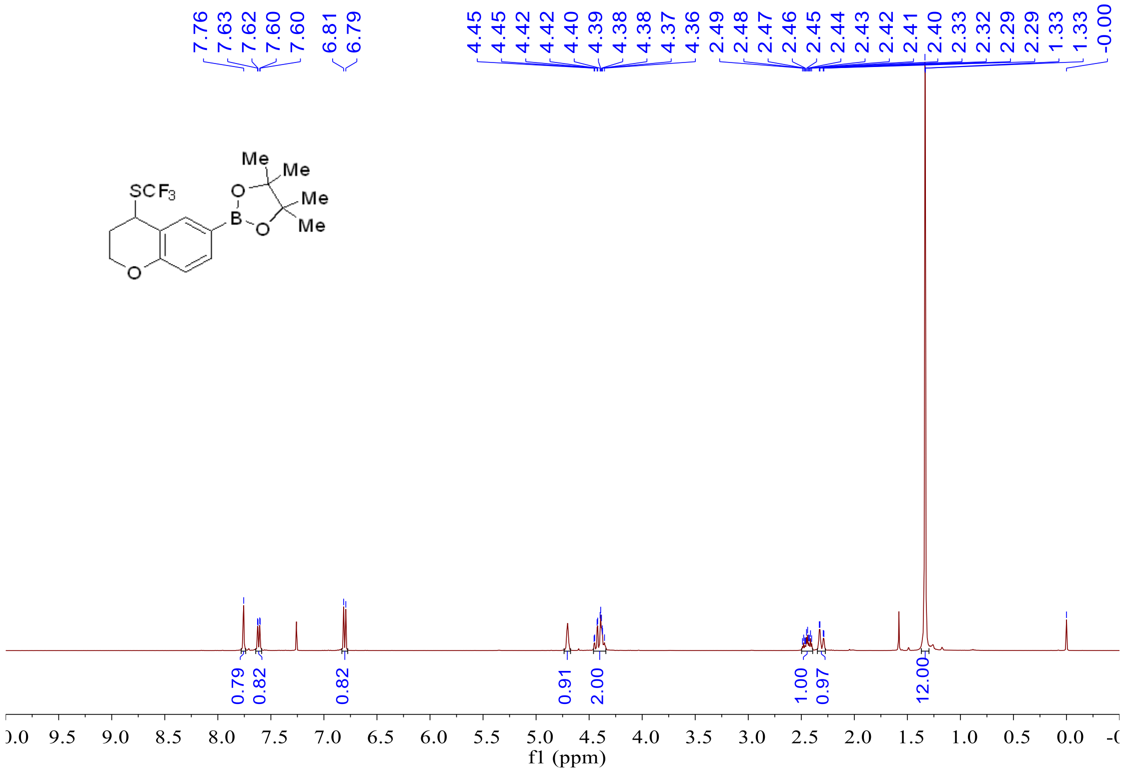

Supplementary Figure 108. ^{1}H NMR spectra for compound **3z**

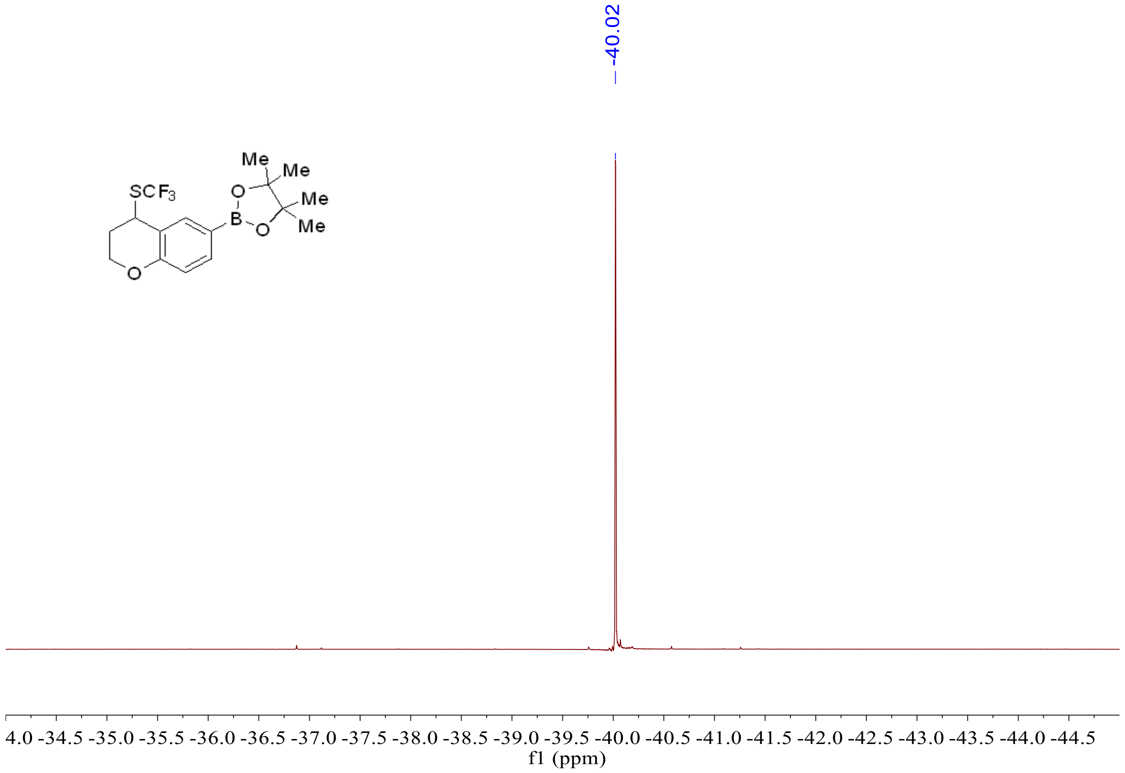

Supplementary Figure 109. ^{19}F NMR spectra for compound **3z**

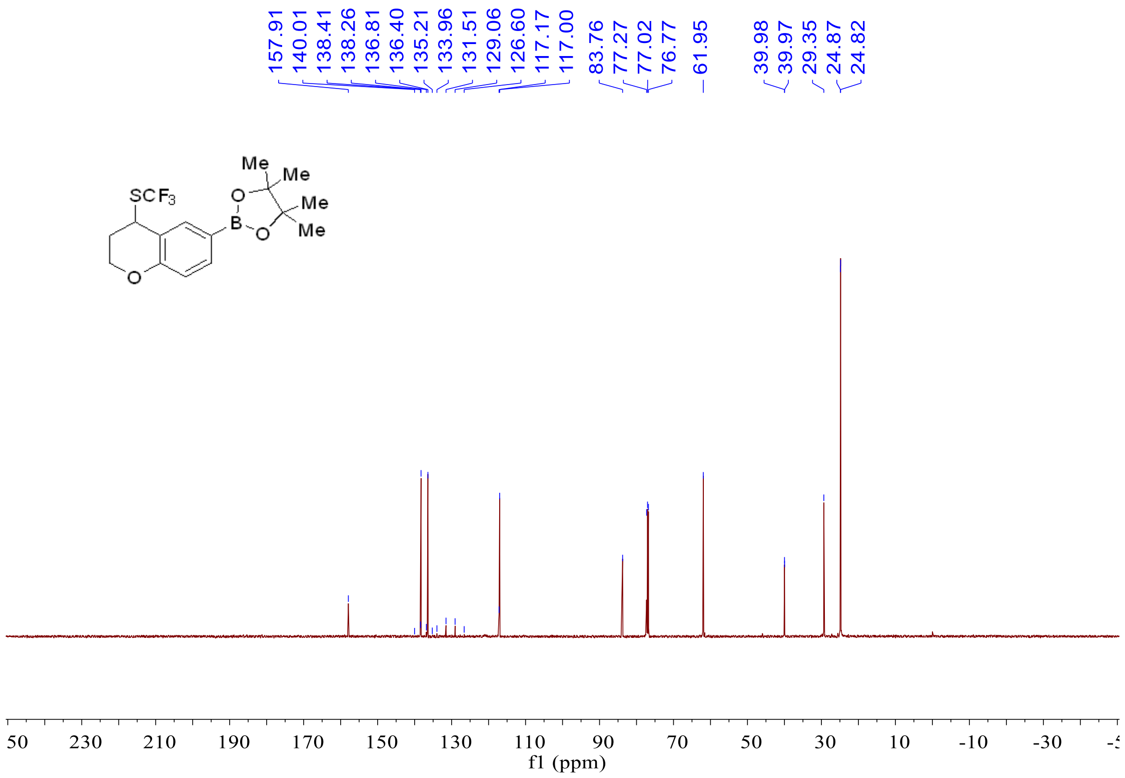

Supplementary Figure 110. ^{13}C NMR spectra for compound **3z**

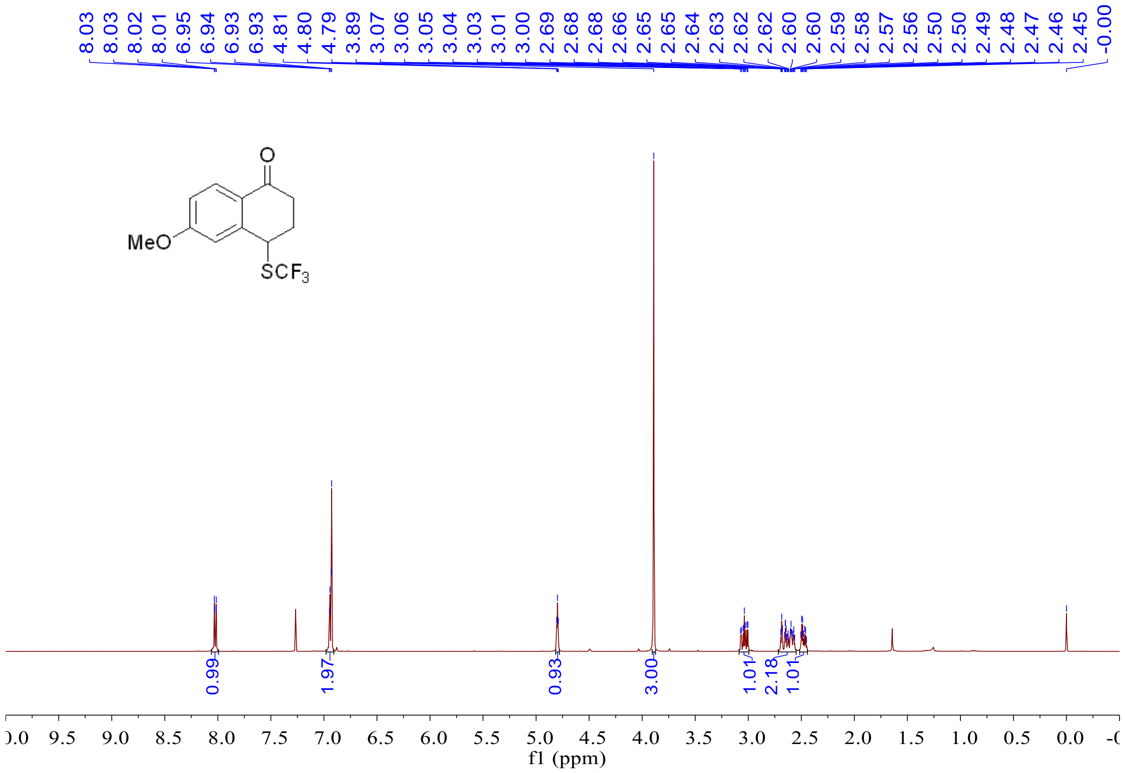

Supplementary Figure 111. ^{1}H NMR spectra for compound **3aa**

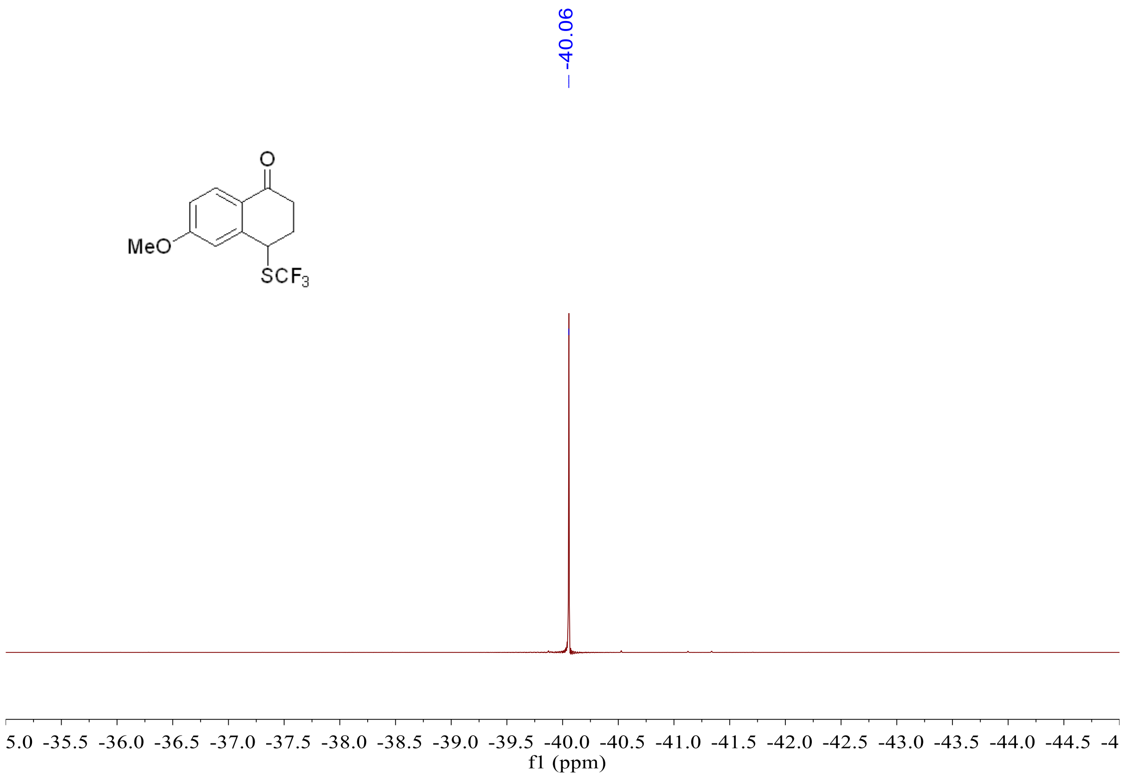

Supplementary Figure 112. ^{19}F NMR spectra for compound **3aa**

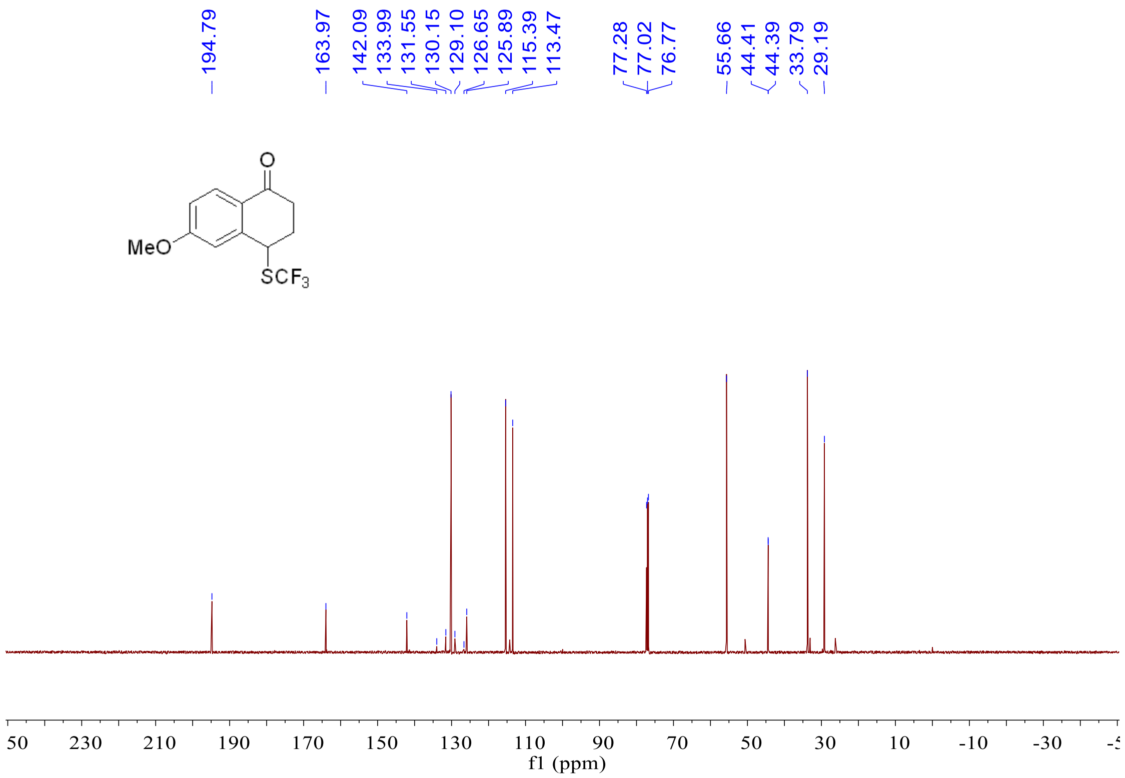

Supplementary Figure 113. ^{13}C NMR spectra for compound **3aa**

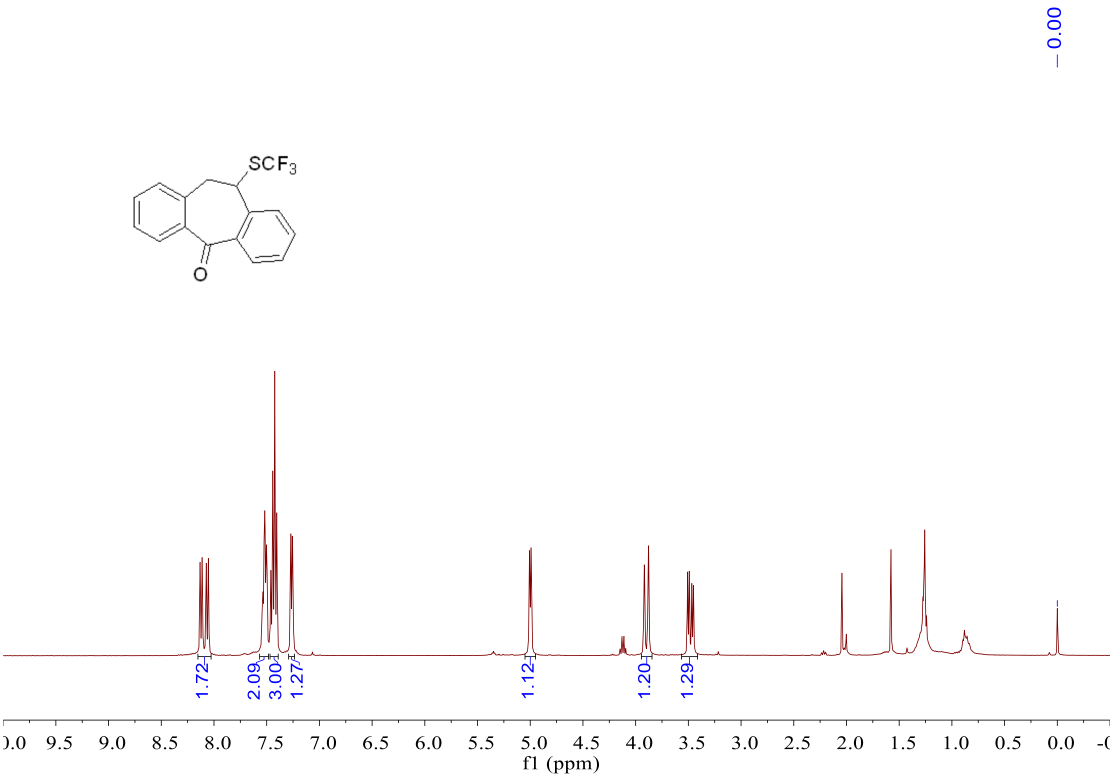

Supplementary Figure 114. ^{1}H NMR spectra for compound **3bb**

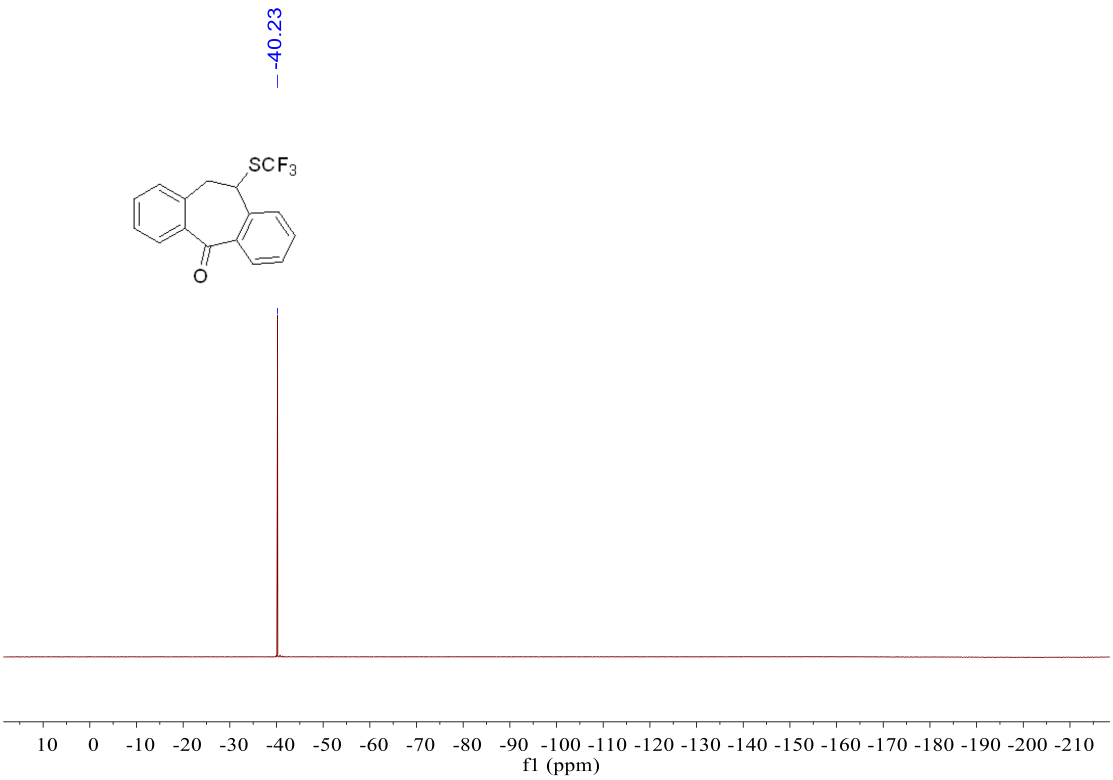

Supplementary Figure 115. ^{19}F NMR spectra for compound **3bb**

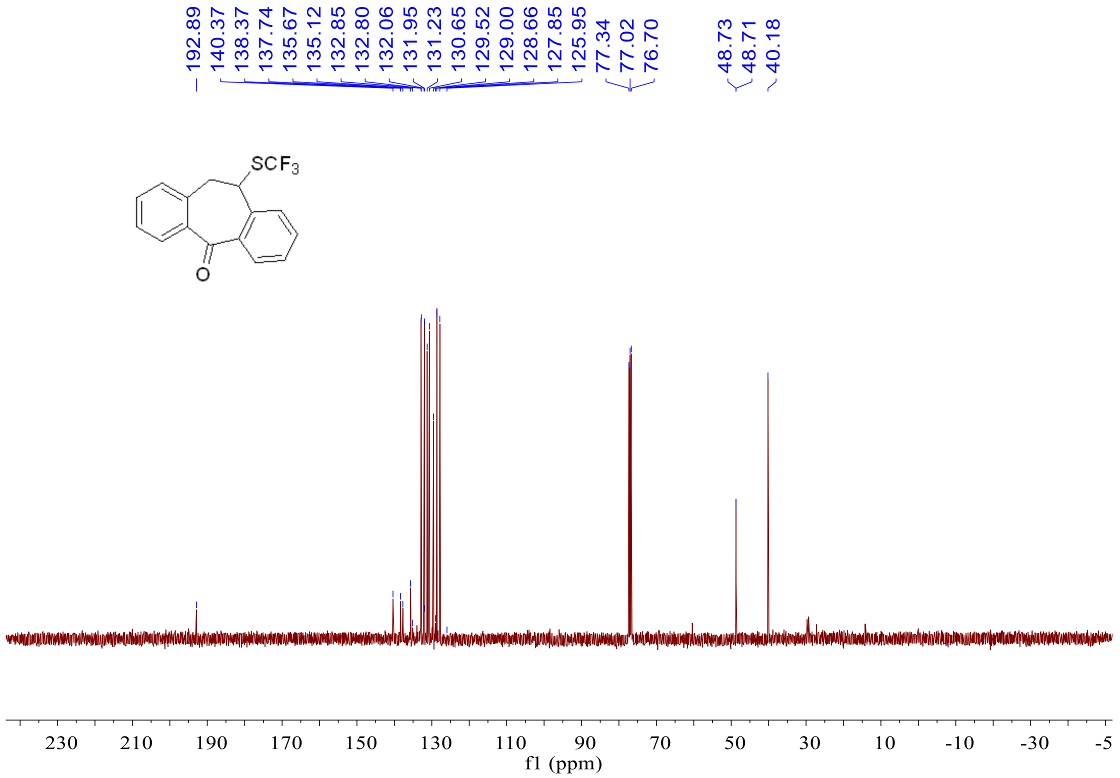

Supplementary Figure 116. ^{13}C NMR spectra for compound **3bb**

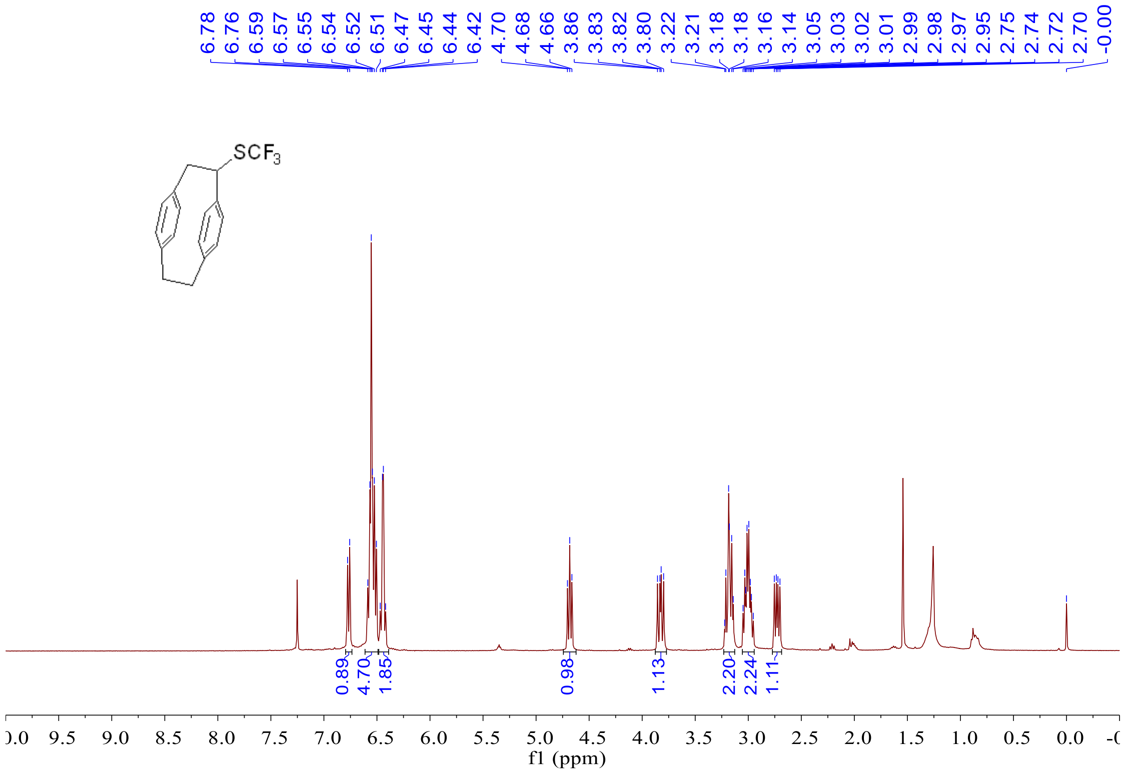

Supplementary Figure 117. ^{1}H NMR spectra for compound **3cc**

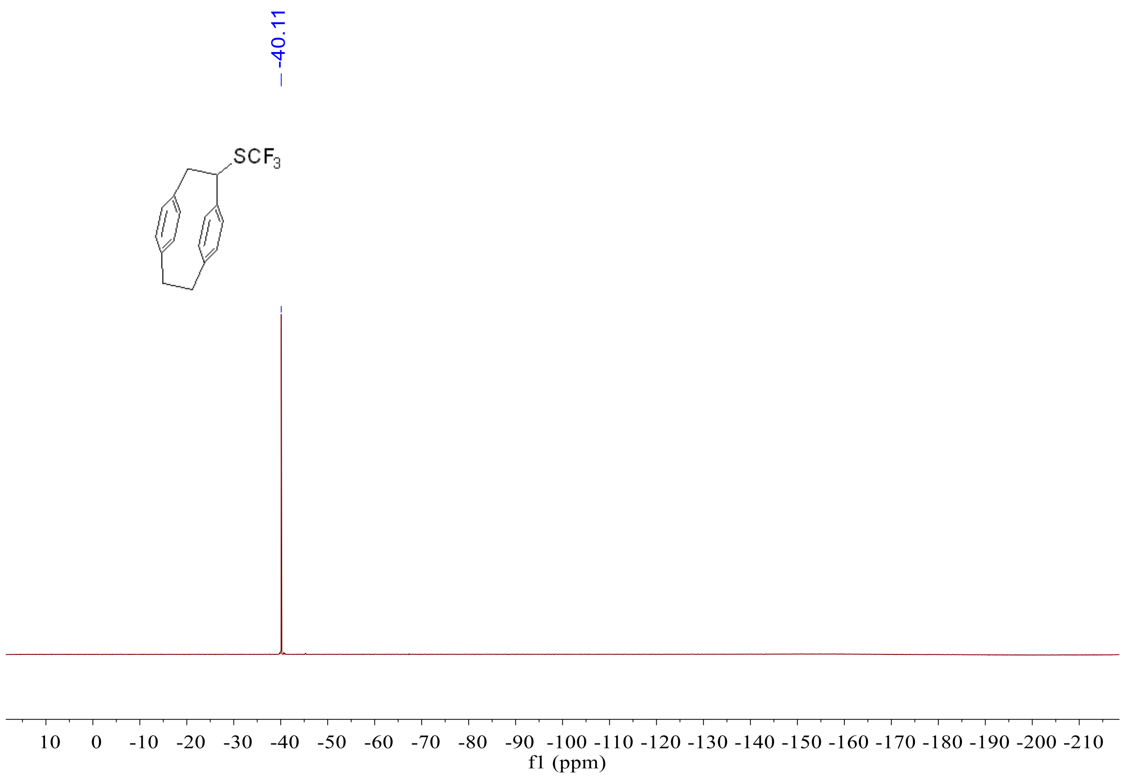

Supplementary Figure 118. ^{19}F NMR spectra for compound **3cc**

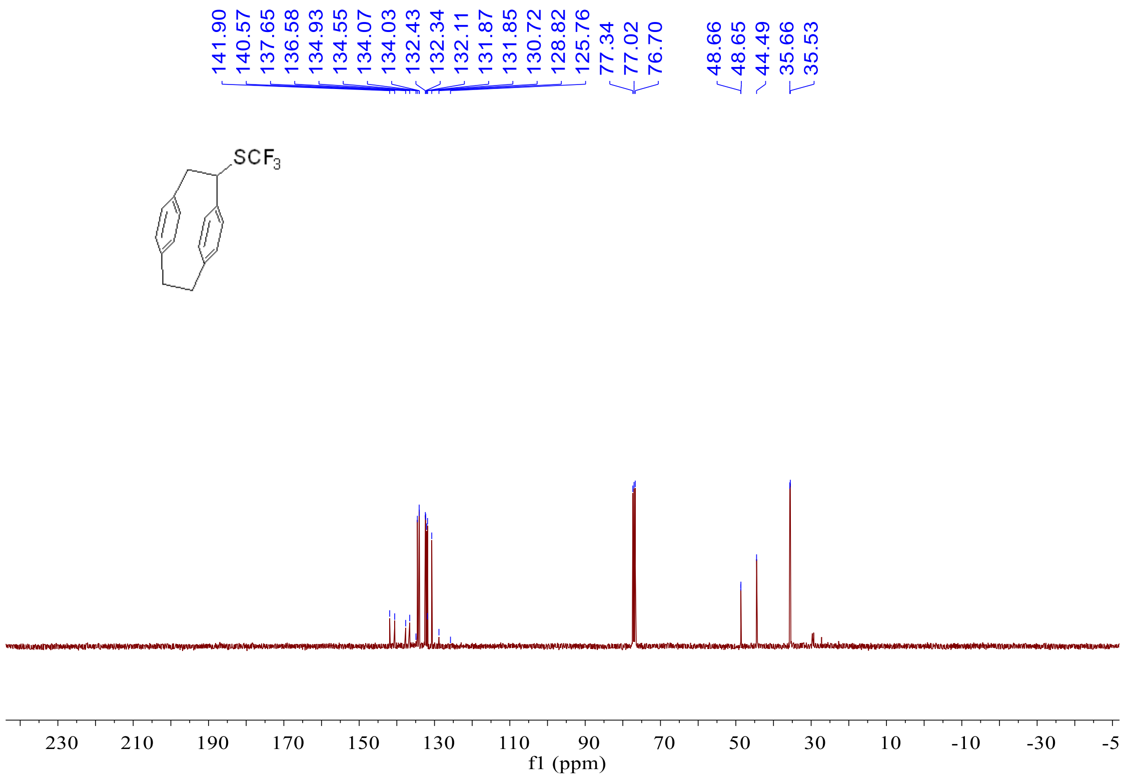

Supplementary Figure 119. ^{13}C NMR spectra for compound **3cc**

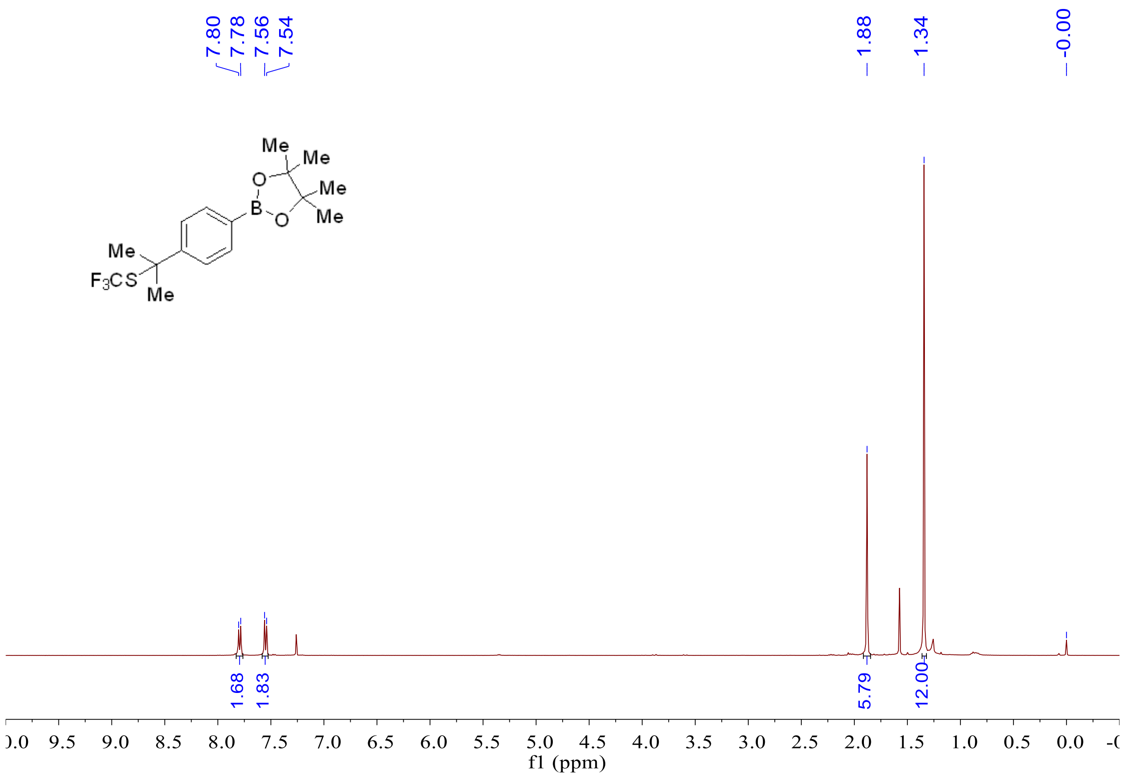

Supplementary Figure 120. ^{1}H NMR spectra for compound **3dd**

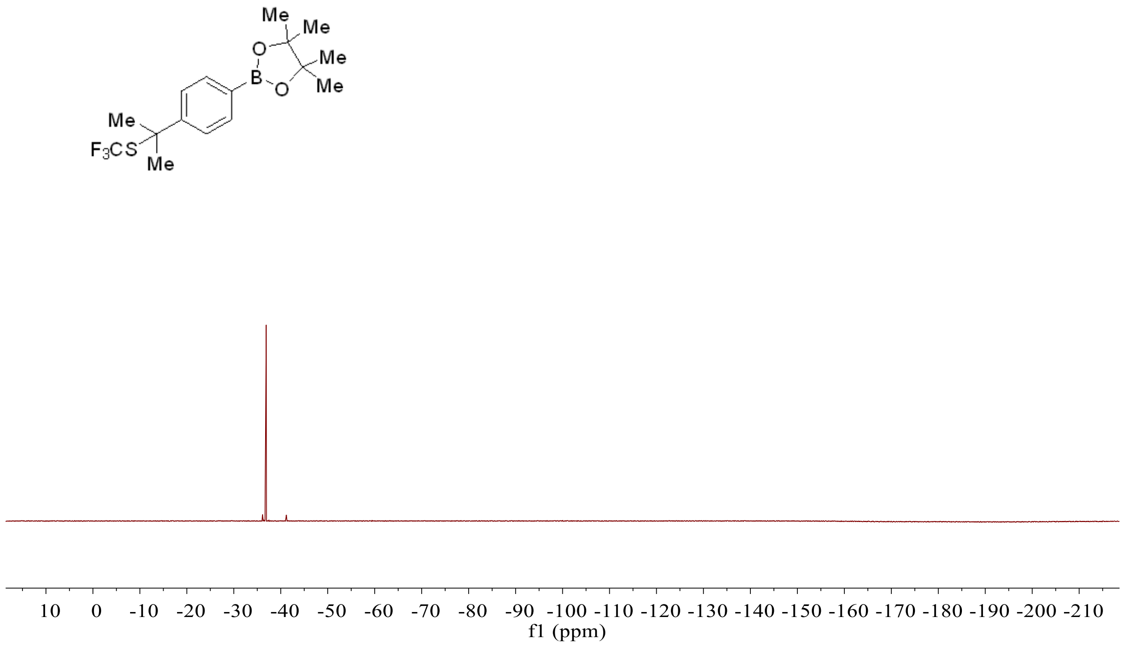

Supplementary Figure 121. ^{19}F NMR spectra for compound **3dd**

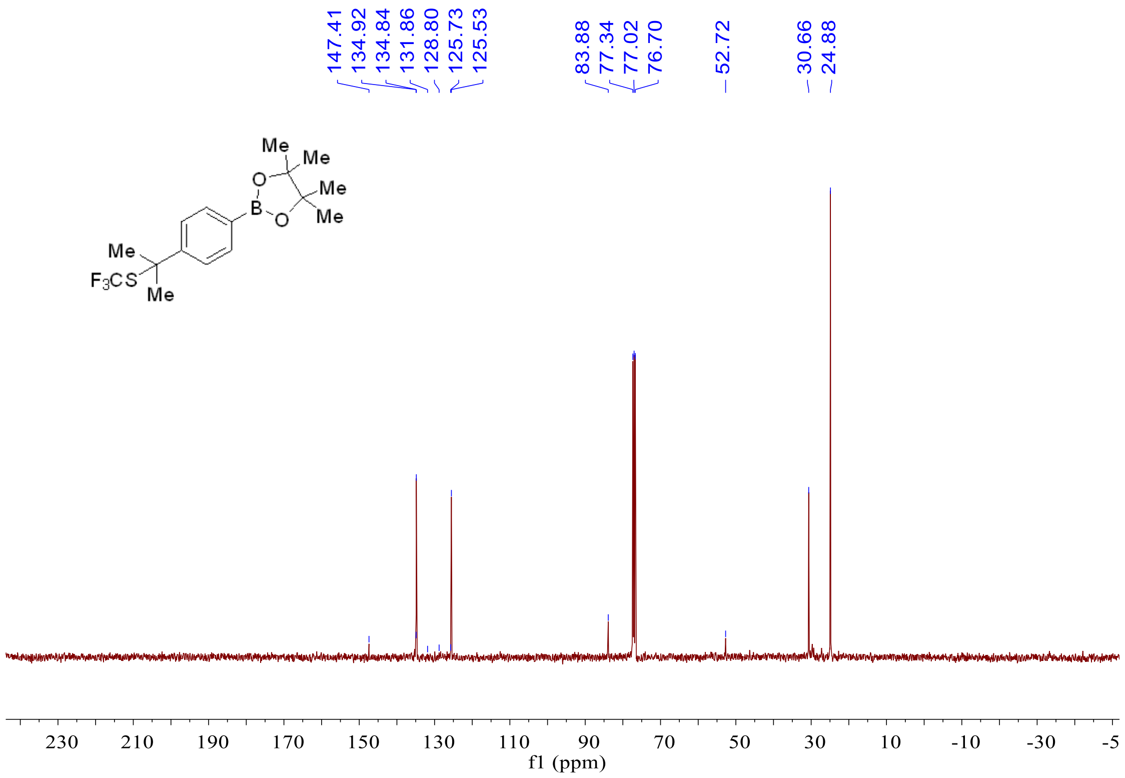

Supplementary Figure 122. ^{13}C NMR spectra for compound **3dd**

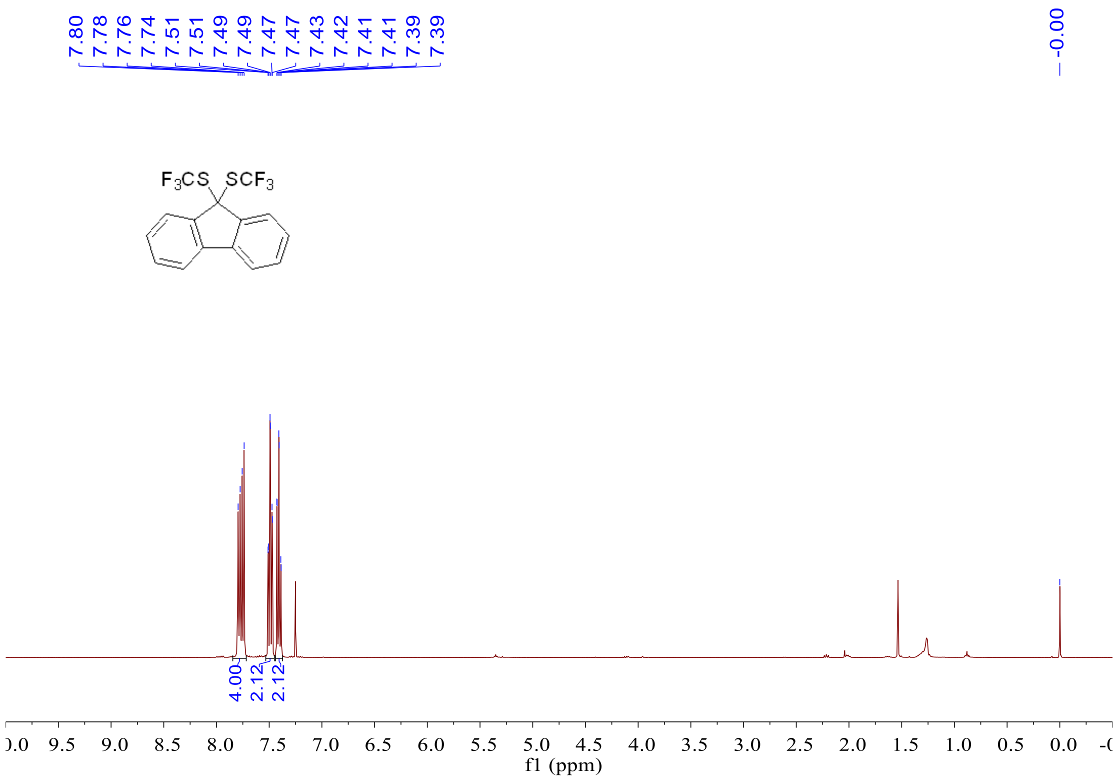

Supplementary Figure 123. ^{1}H NMR spectra for compound **3ee**

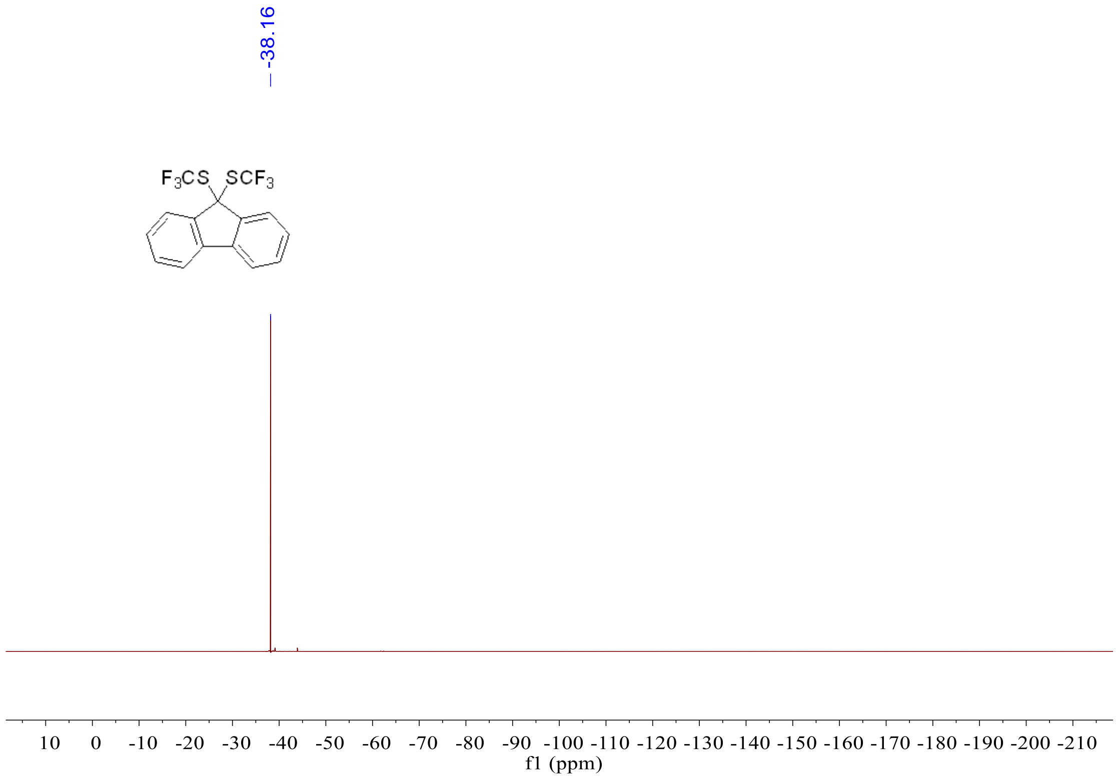

Supplementary Figure 124. ^{19}F NMR spectra for compound **3ee**

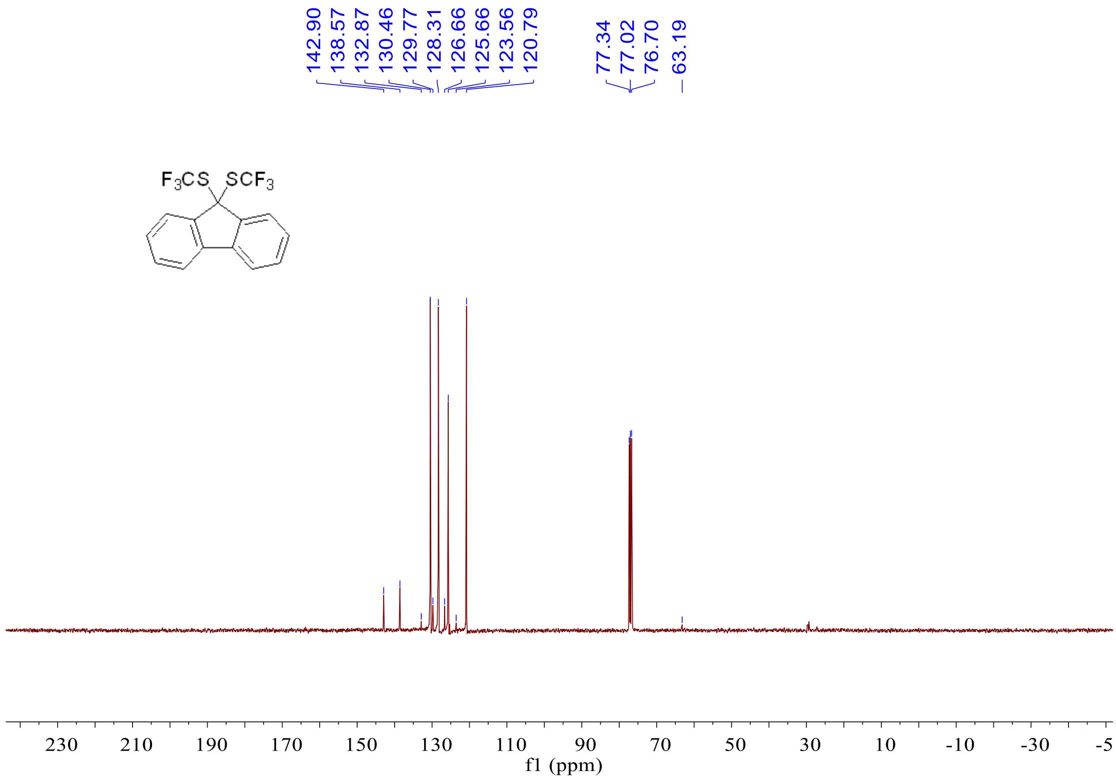

Supplementary Figure 125. ^{13}C NMR spectra for compound **3ee**

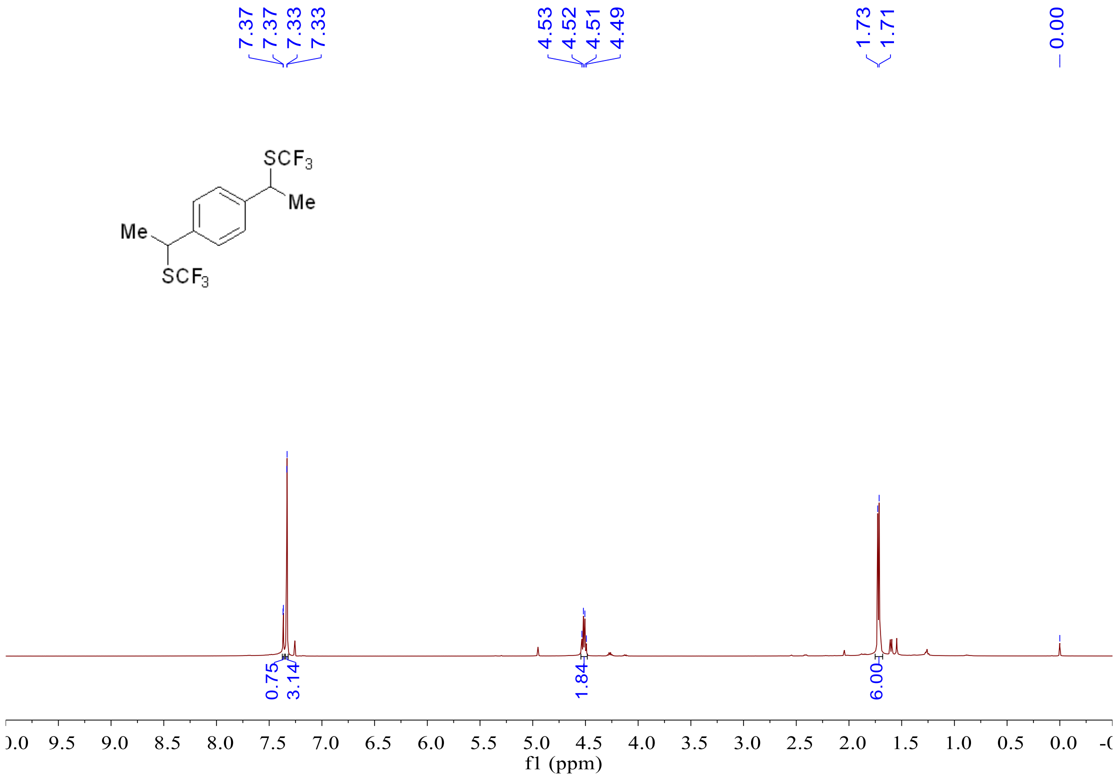

Supplementary Figure 126. ^{1}H NMR spectra for compound **3ff**

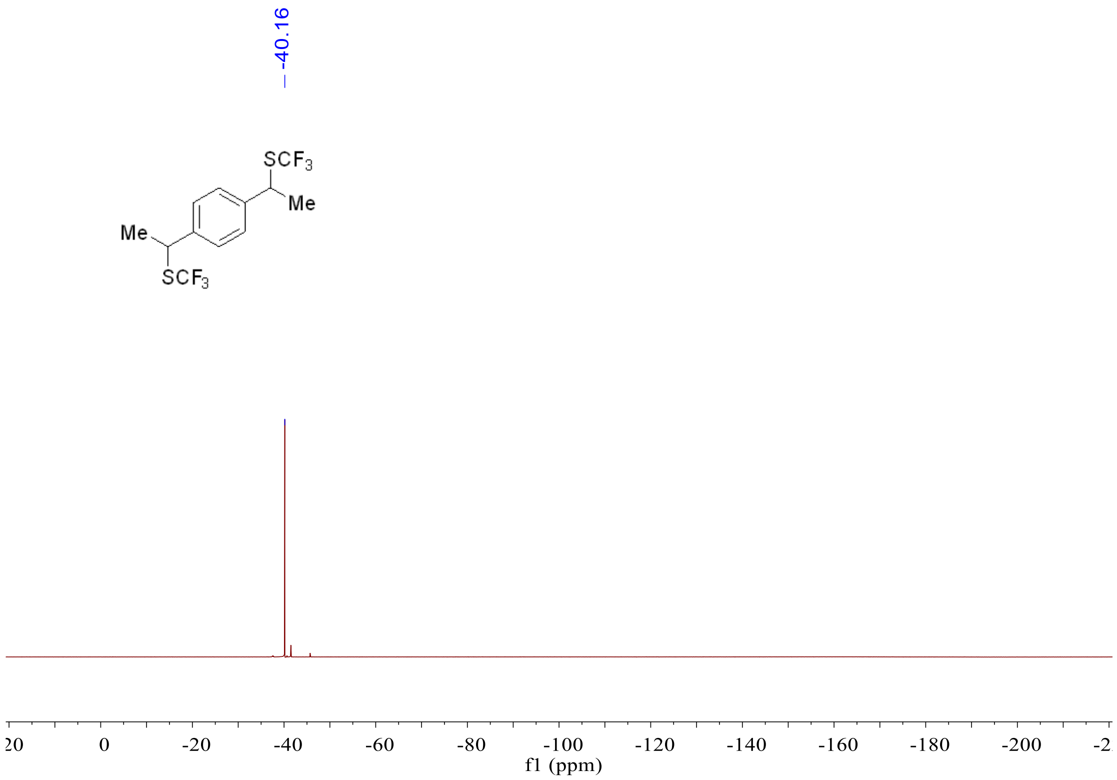

Supplementary Figure 127. ^{19}F NMR spectra for compound **3ff**

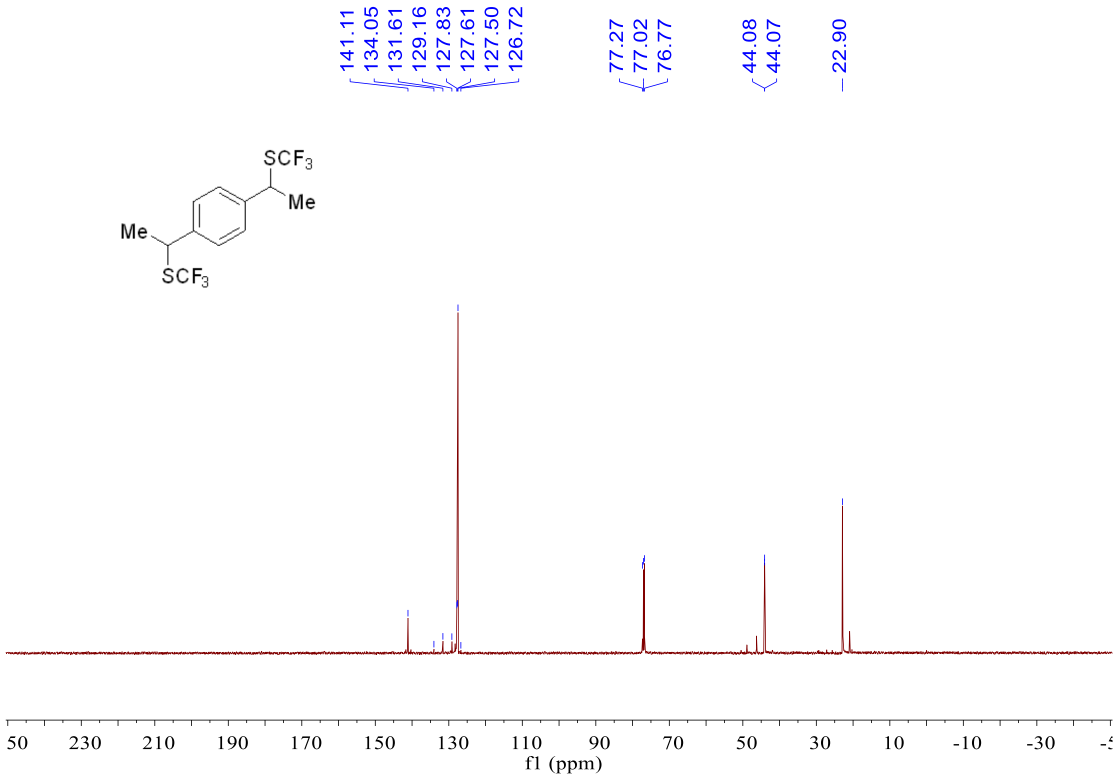

Supplementary Figure 128. ^{13}C NMR spectra for compound **3ff**

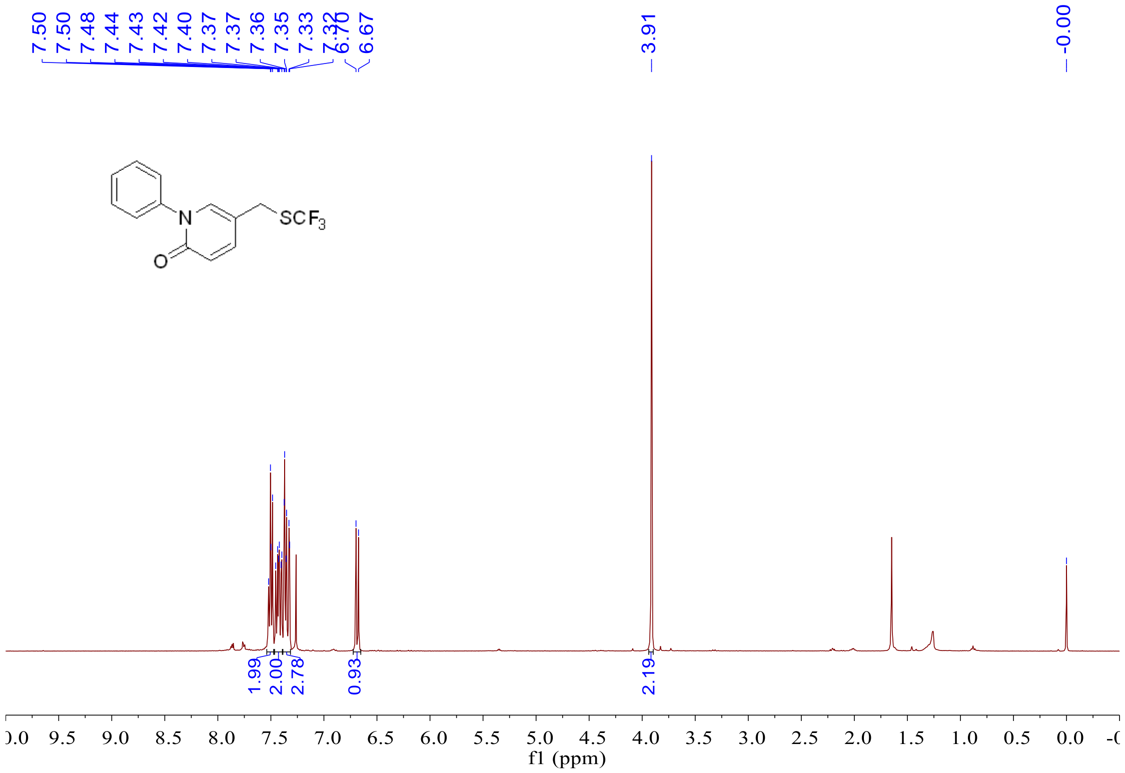

Supplementary Figure 129. ^{1}H NMR spectra for compound **4**

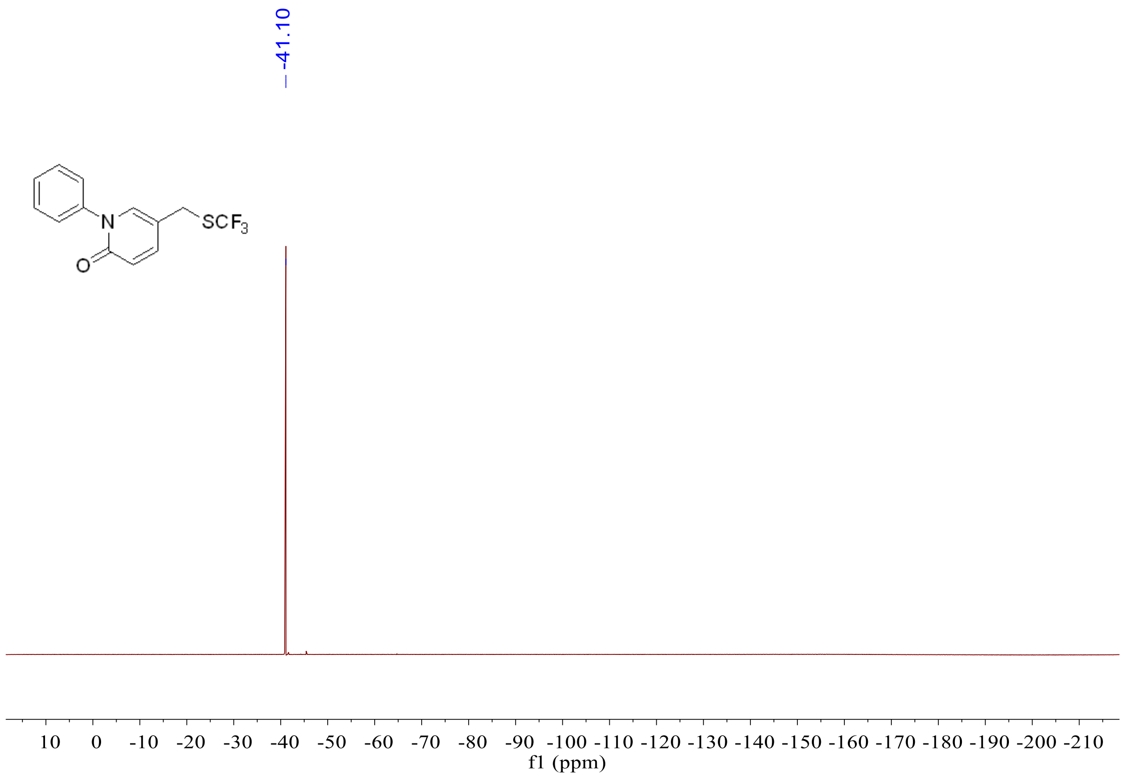

Supplementary Figure 130. ^{19}F NMR spectra for compound **4**

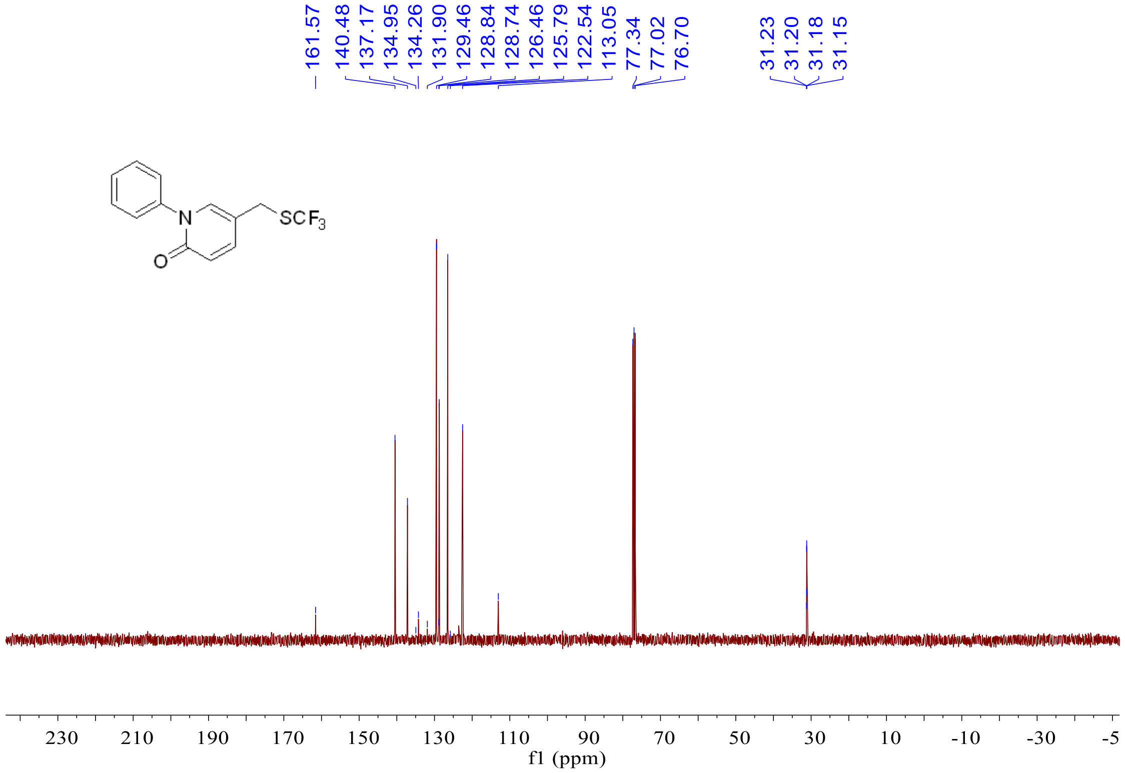

Supplementary Figure 131. ^{13}C NMR spectra for compound **4**

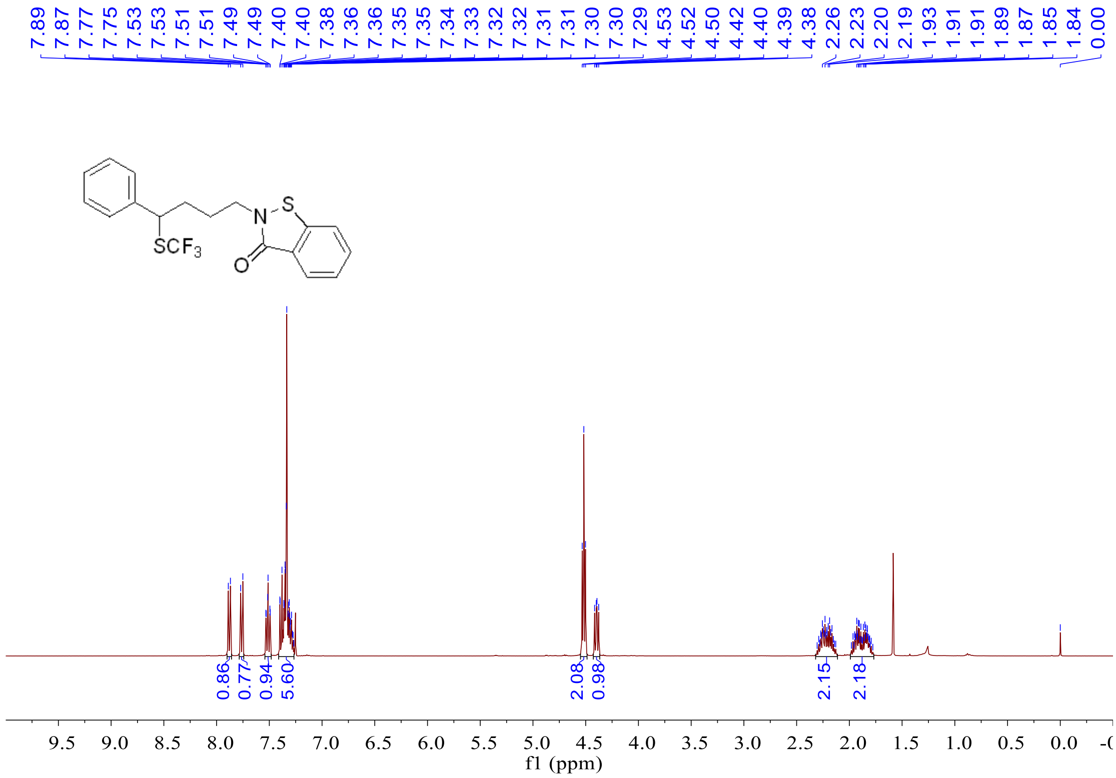

Supplementary Figure 132. ^{1}H NMR spectra for compound **5**

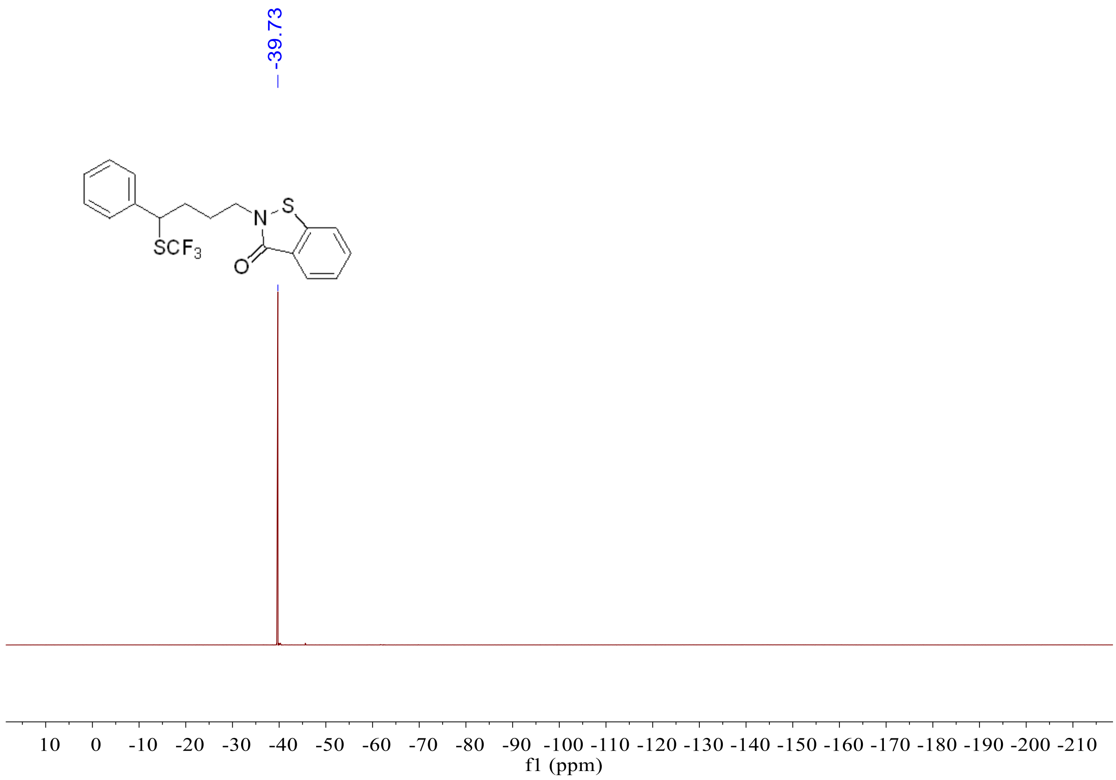

Supplementary Figure 133. ^{19}F NMR spectra for compound **5**

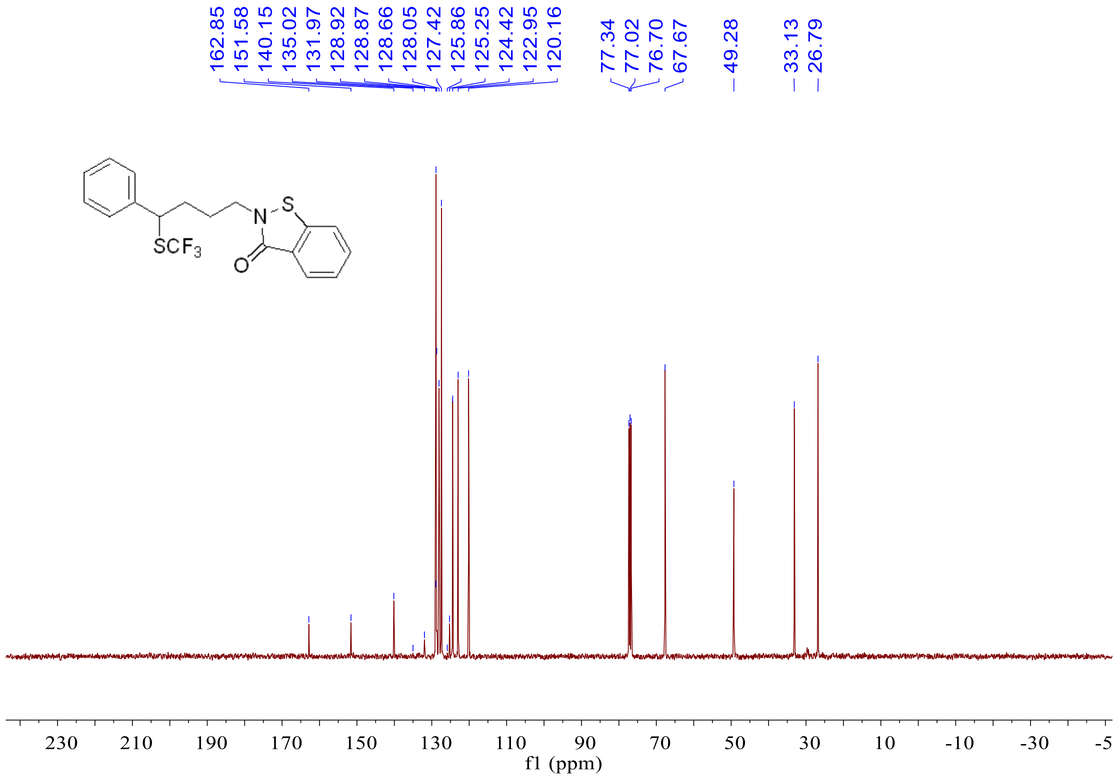

Supplementary Figure 134. ^{13}C NMR spectra for compound **5**

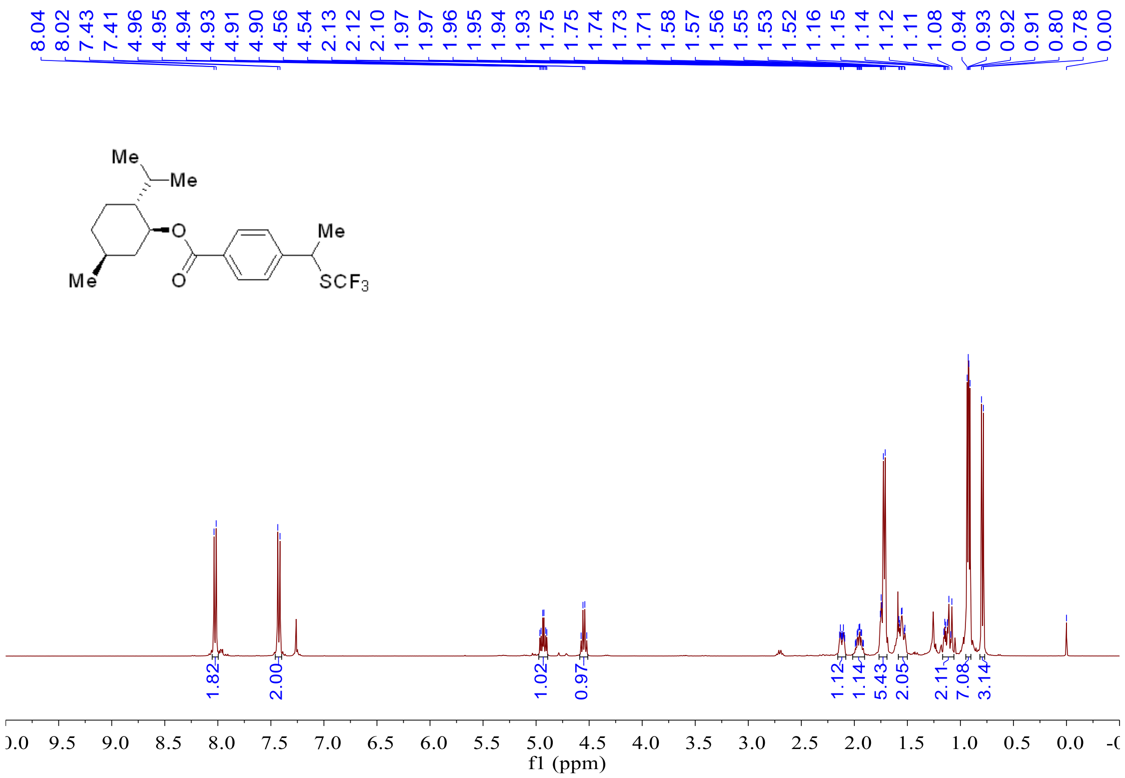

Supplementary Figure 135. ^{1}H NMR spectra for compound **6**

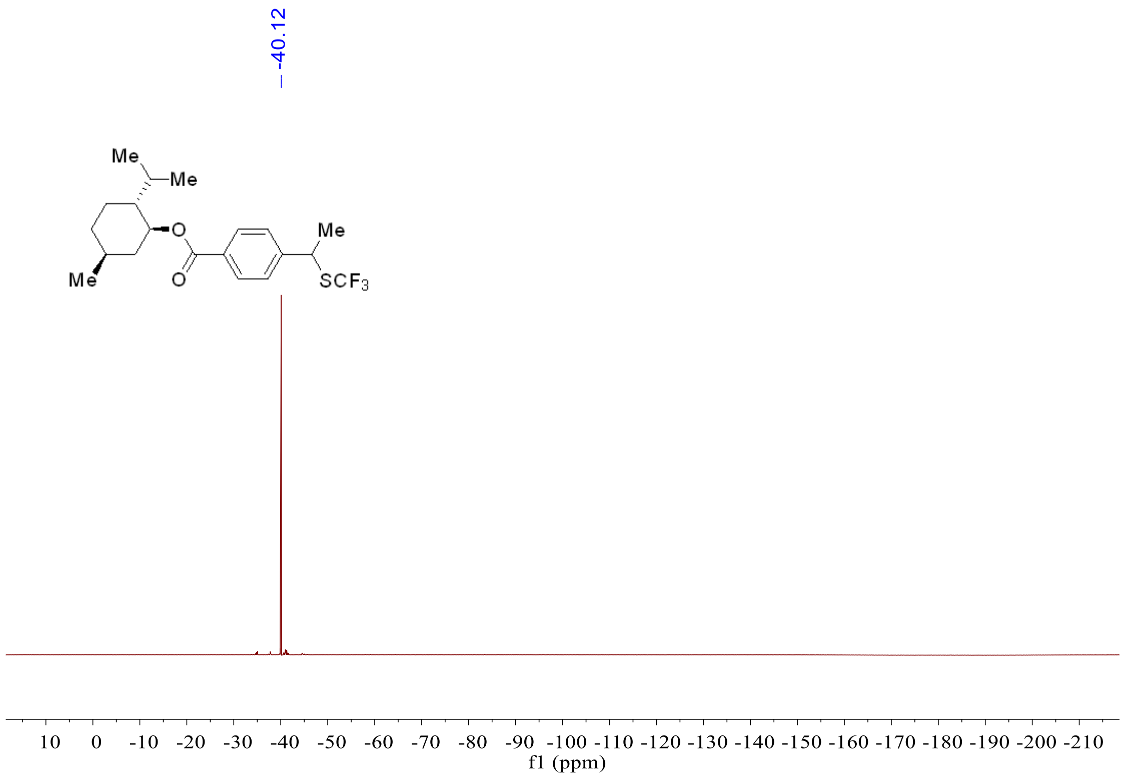

Supplementary Figure 136. ^{19}F NMR spectra for compound **6**

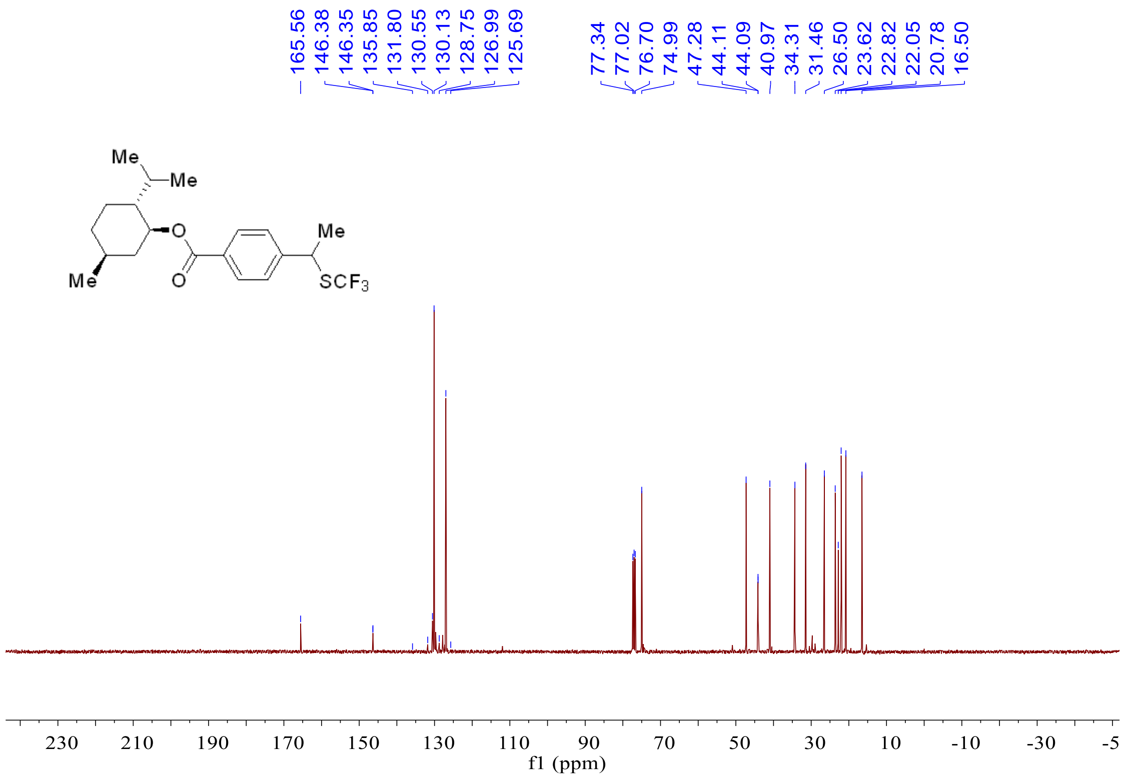

Supplementary Figure 137. ^{13}C NMR spectra for compound **6**

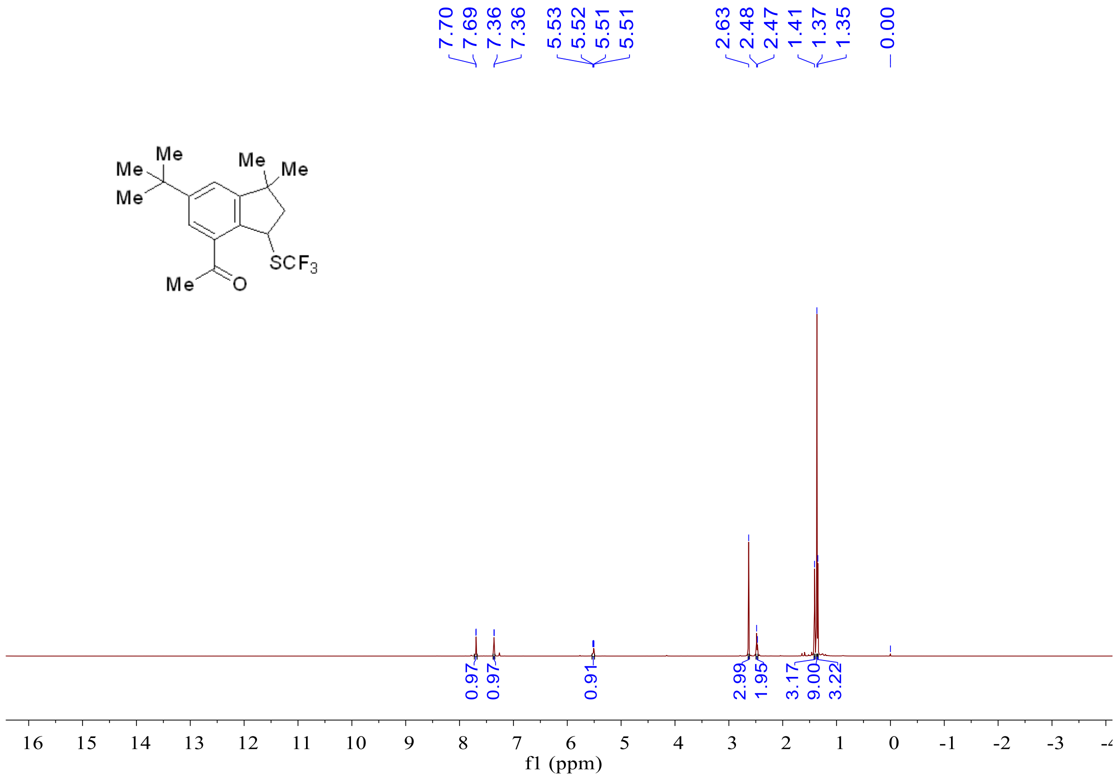

Supplementary Figure 138. ^{1}H NMR spectra for compound **7**

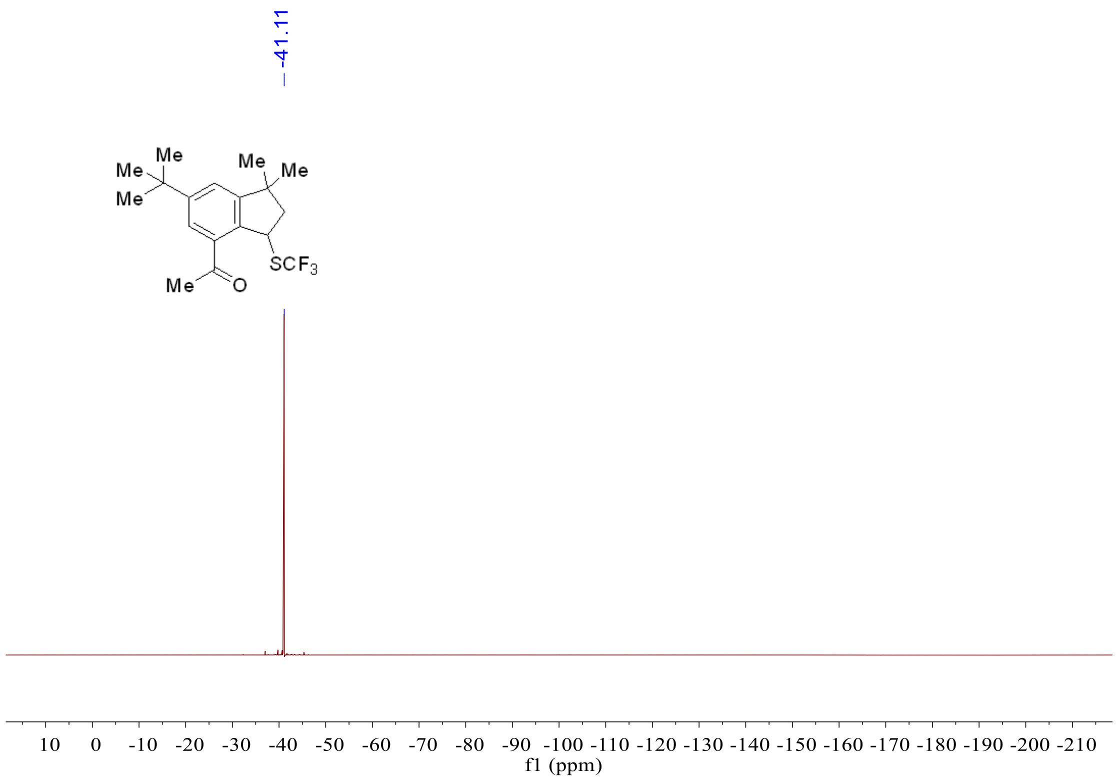

Supplementary Figure 139. ^{19}F NMR spectra for compound **7**

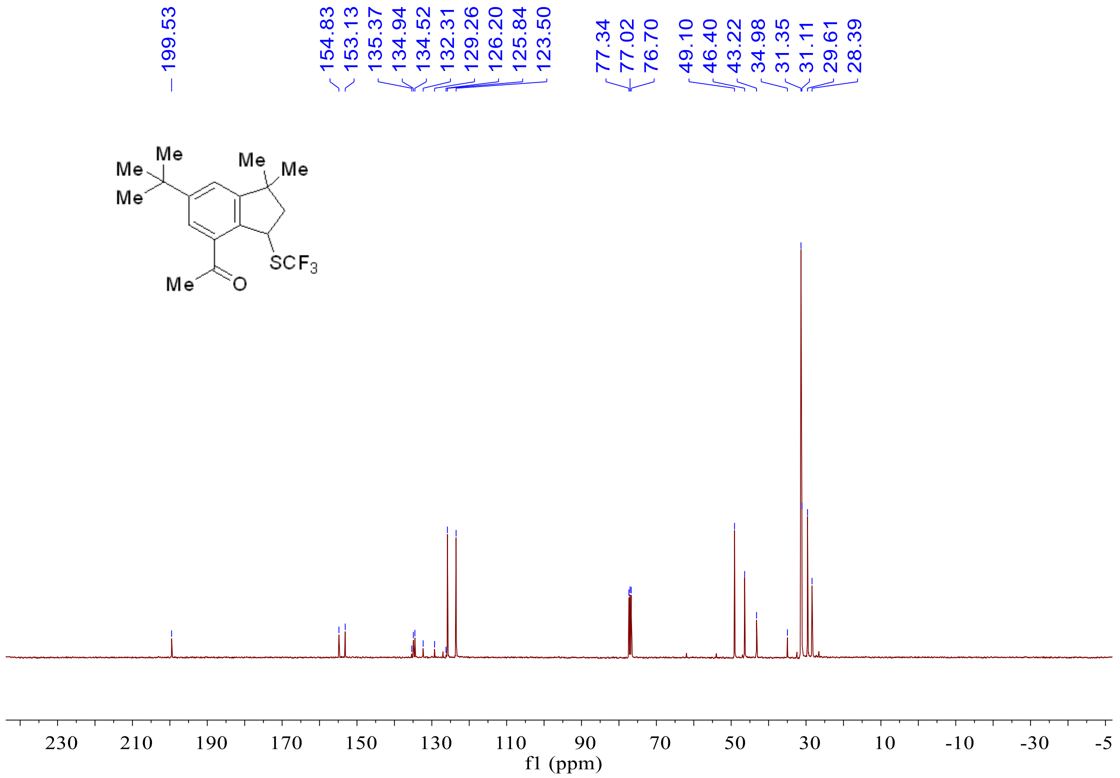

Supplementary Figure 140. ^{13}C NMR spectra for compound **7**

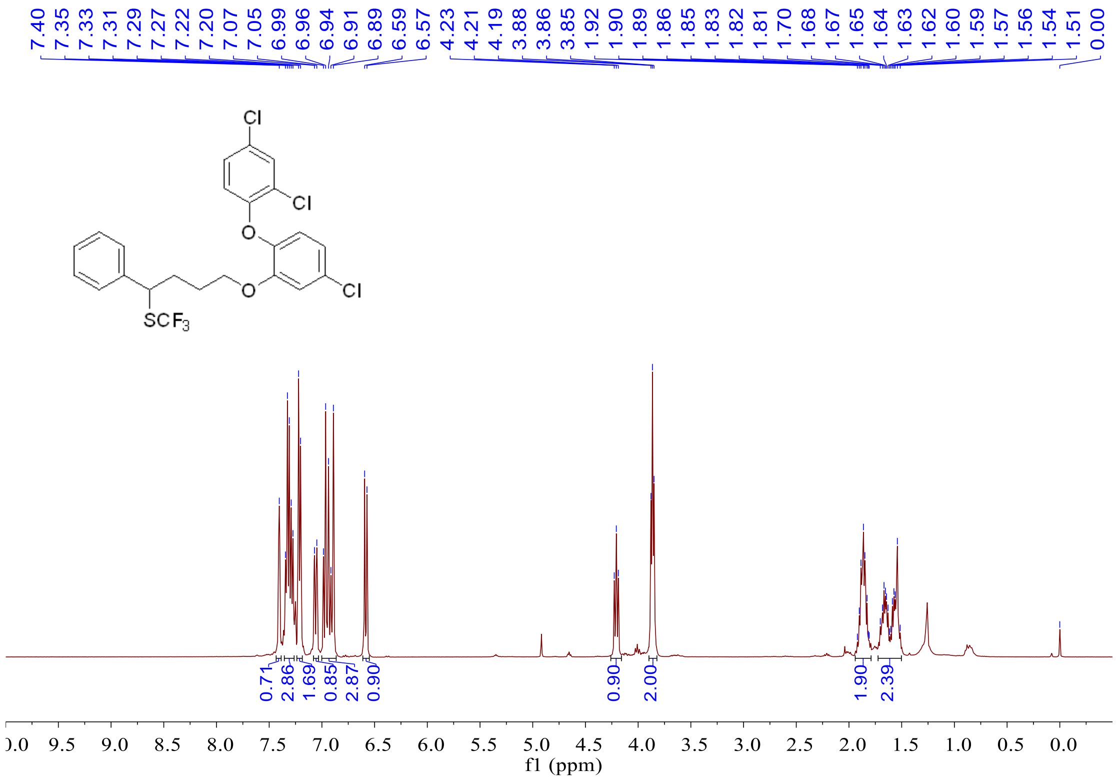

Supplementary Figure 141. ^{1}H NMR spectra for compound **8**

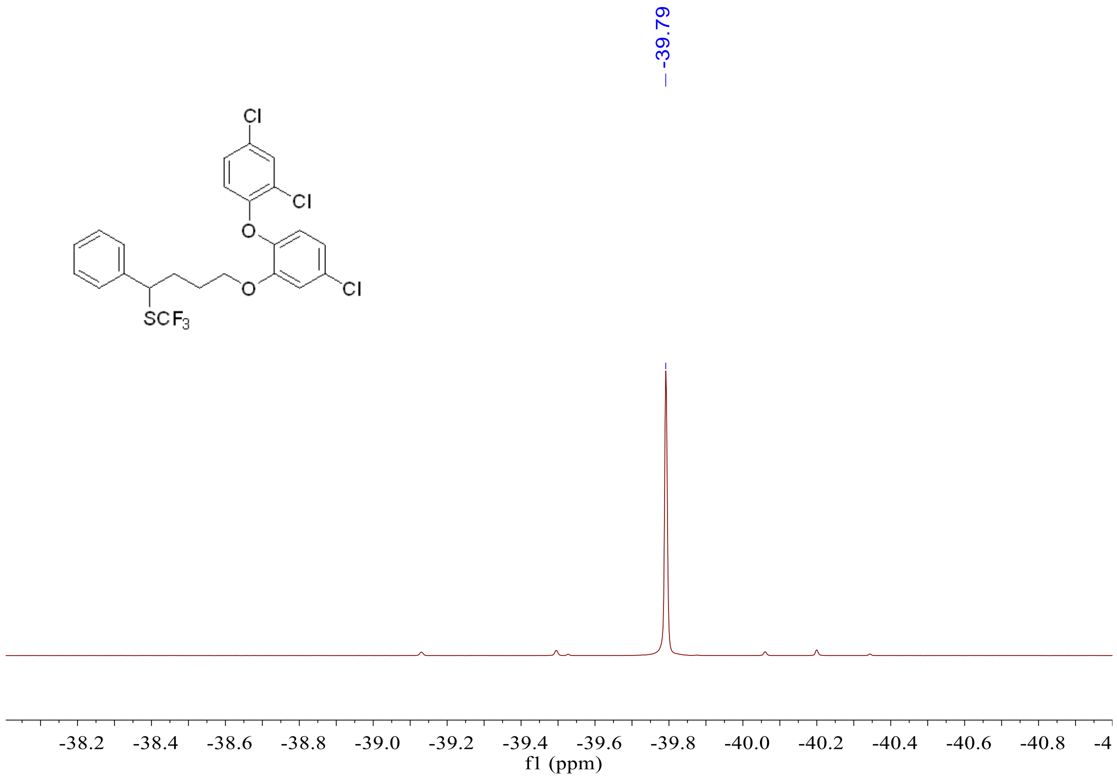

Supplementary Figure 142. ^{19}F NMR spectra for compound **8**

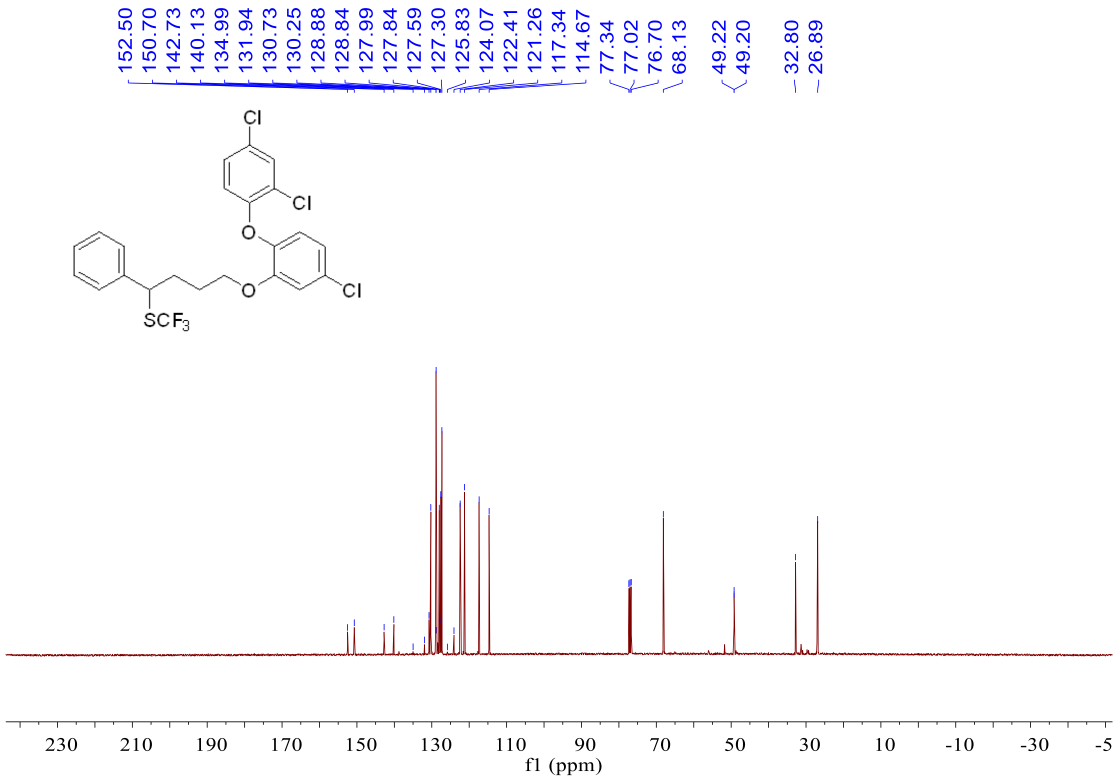

Supplementary Figure 143. ^{13}C NMR spectra for compound **8**

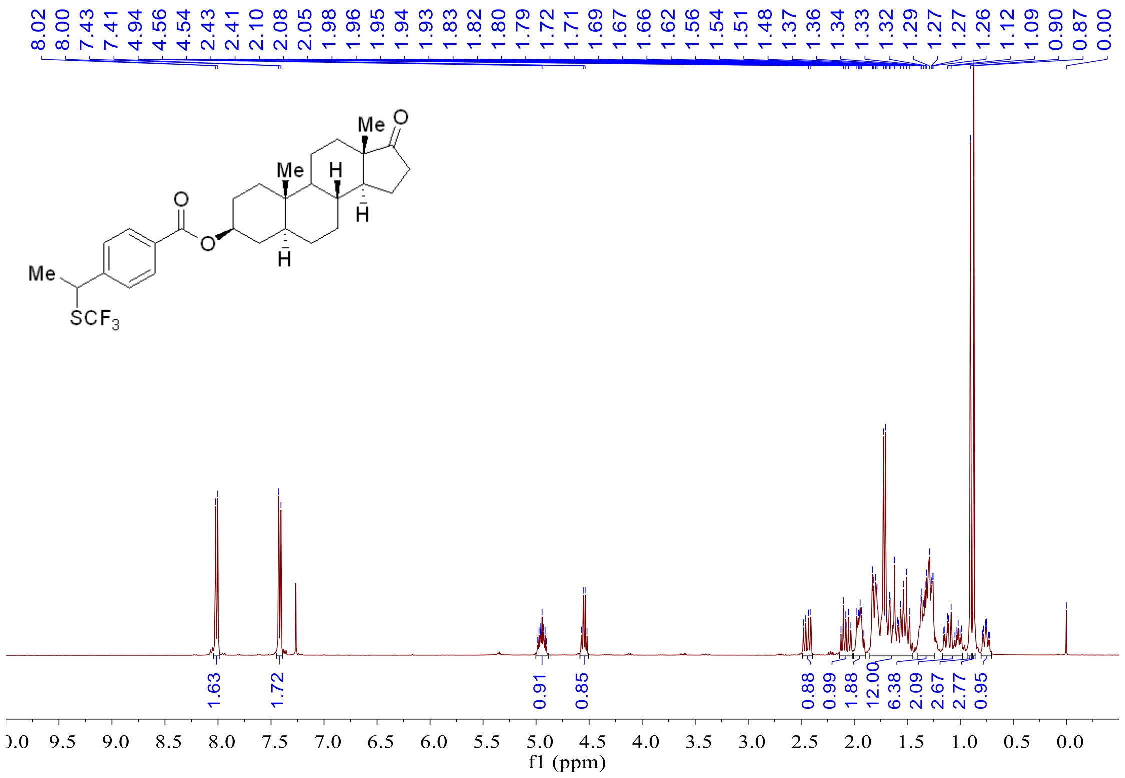

Supplementary Figure 144. ^{1}H NMR spectra for compound **9**

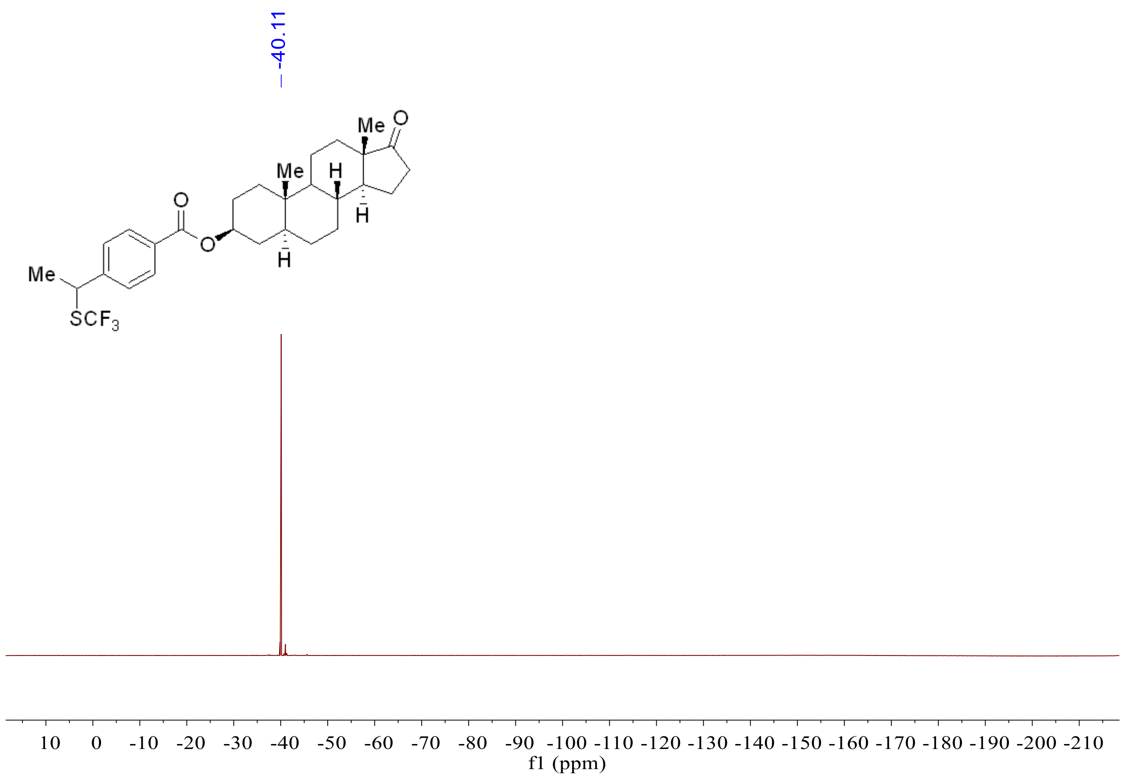

Supplementary Figure 145. ^{19}F NMR spectra for compound **9**

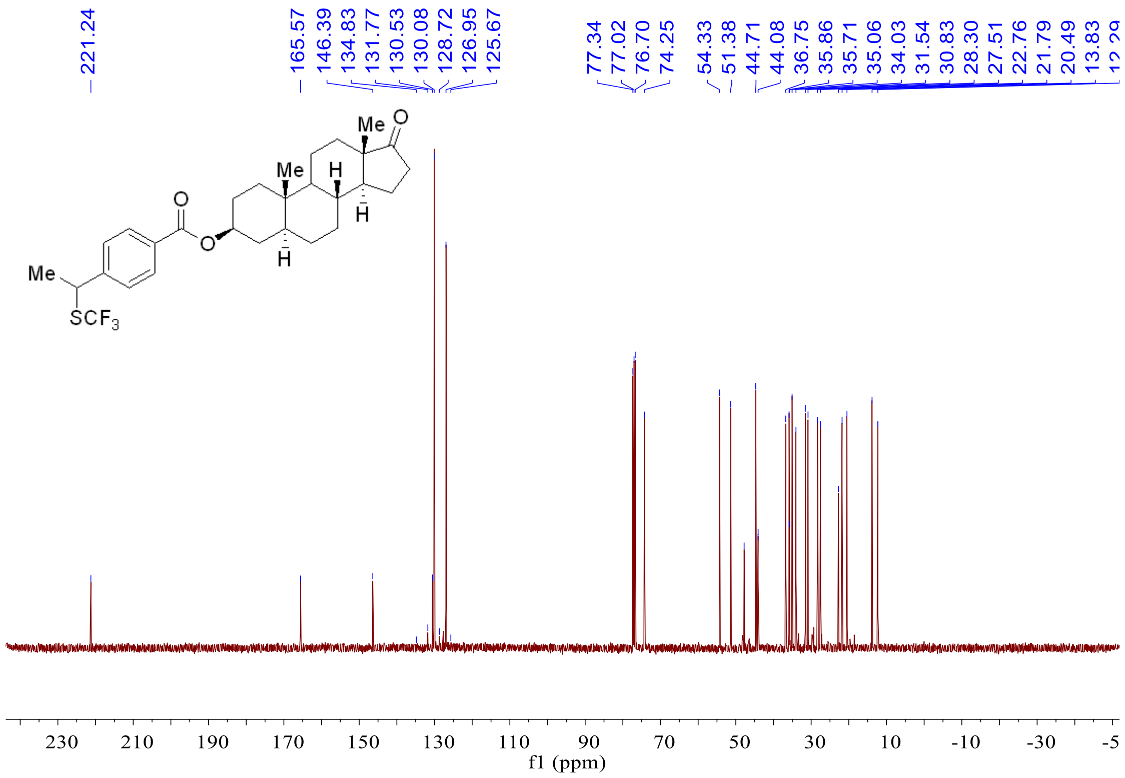

Supplementary Figure 146. ^{13}C NMR spectra for compound **9**

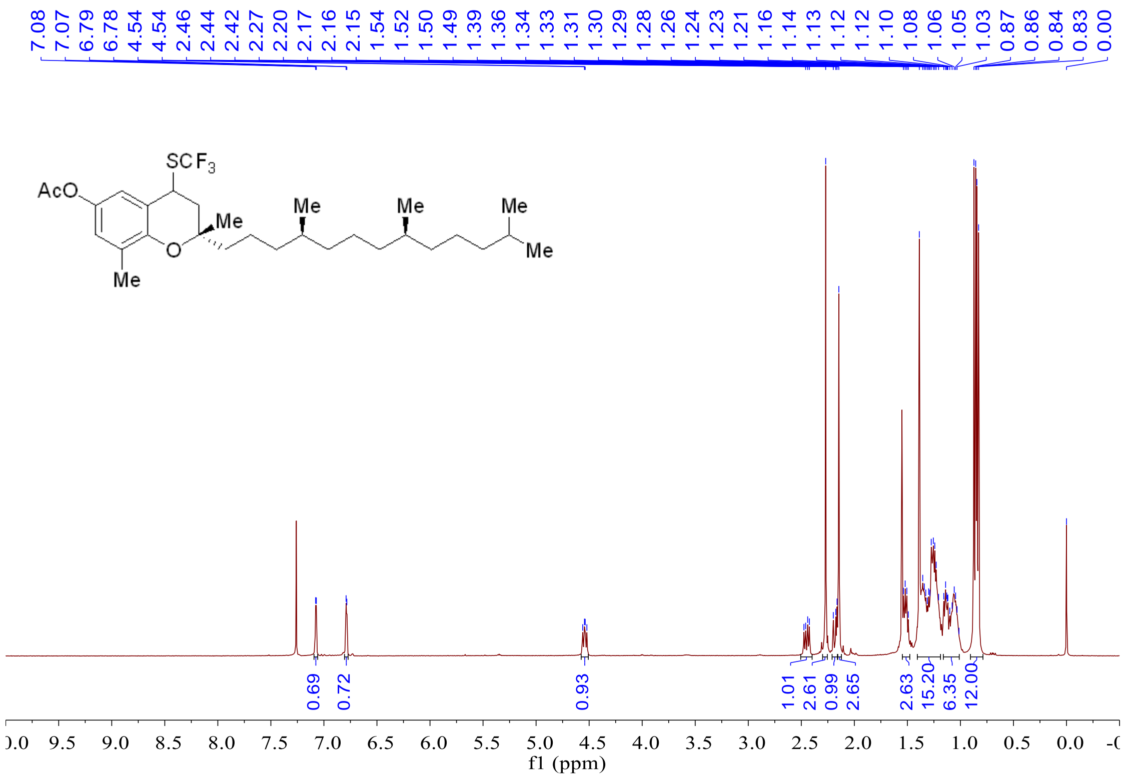

Supplementary Figure 147. ^{1}H NMR spectra for compound **10**

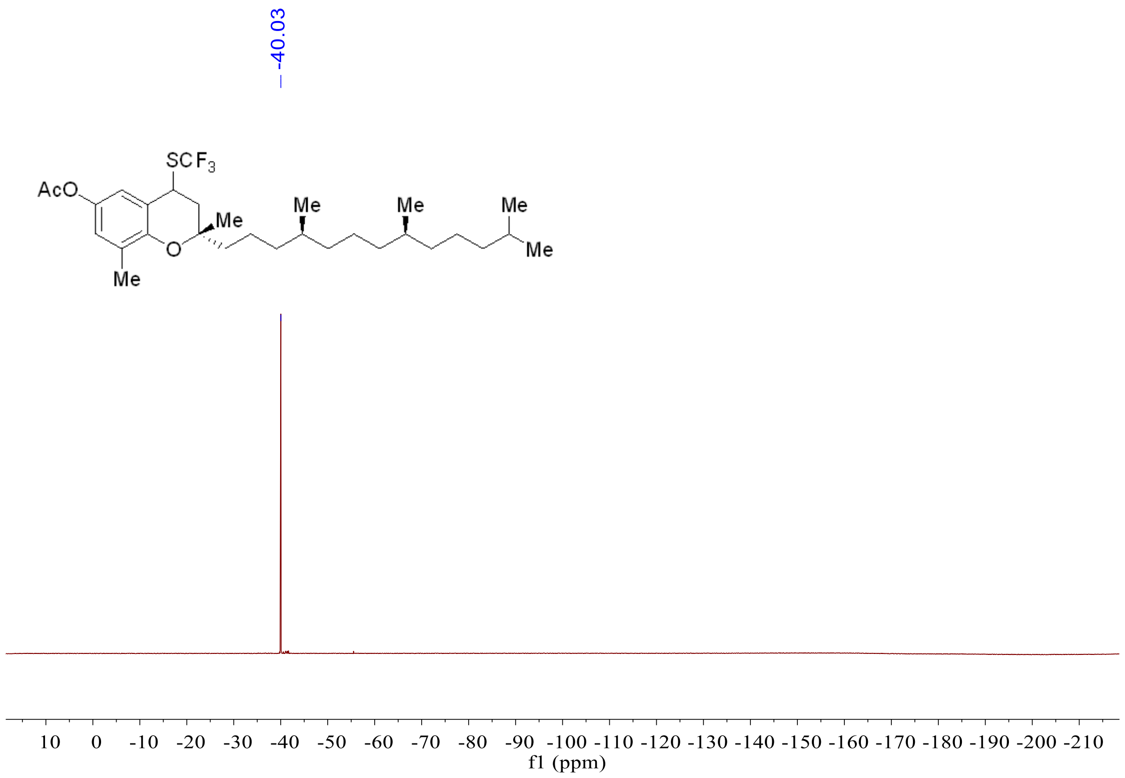

Supplementary Figure 148. ^{19}F NMR spectra for compound **10**

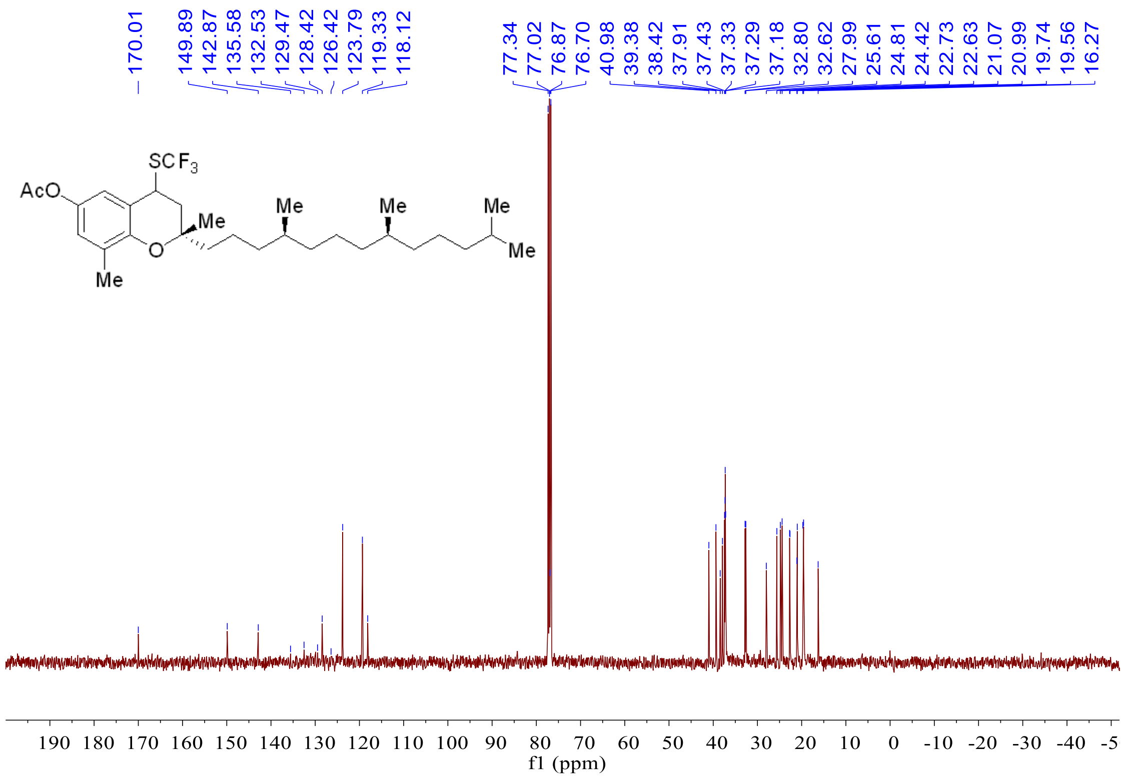

Supplementary Figure 149. ^{13}C NMR spectra for compound **10**

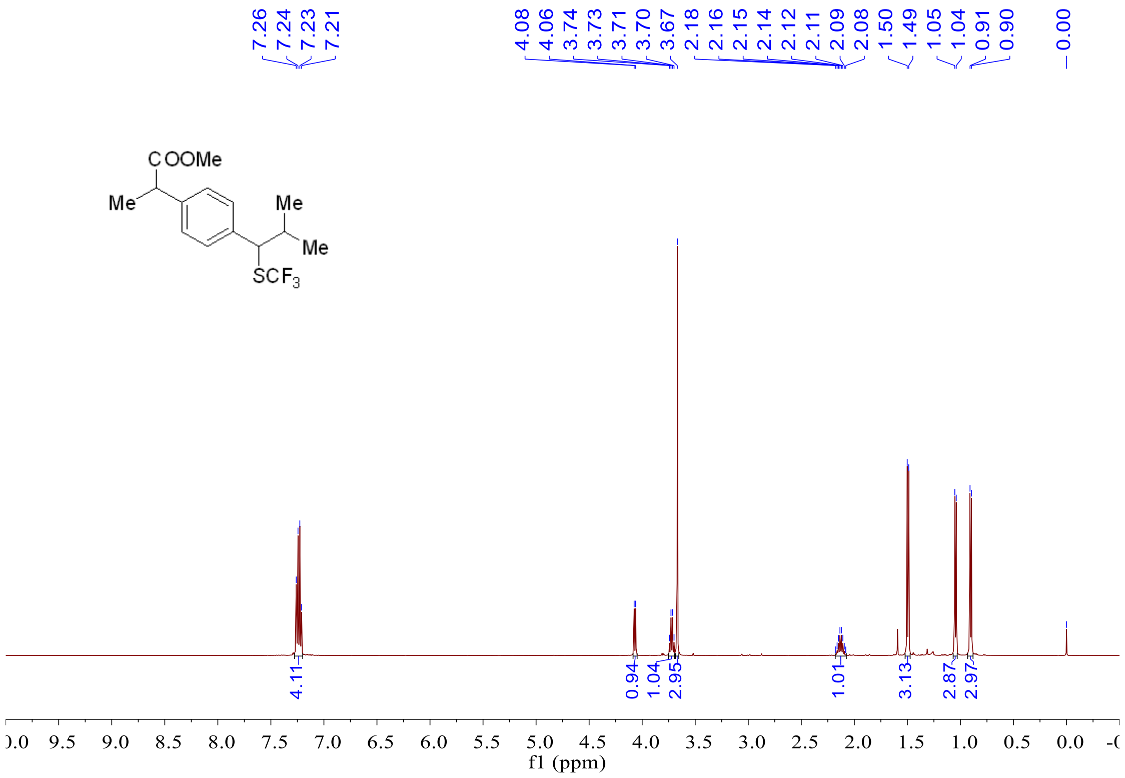

Supplementary Figure 150. ^{1}H NMR spectra for compound **11**

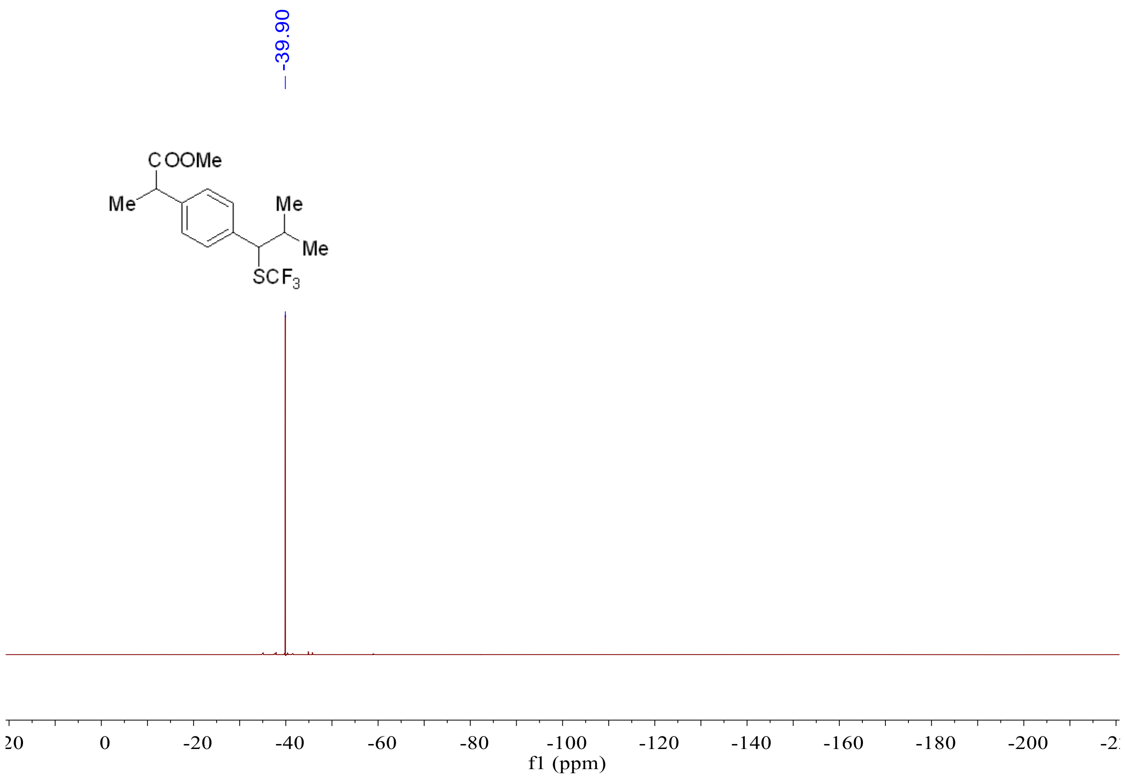

Supplementary Figure 151. ^{19}F NMR spectra for compound **11**

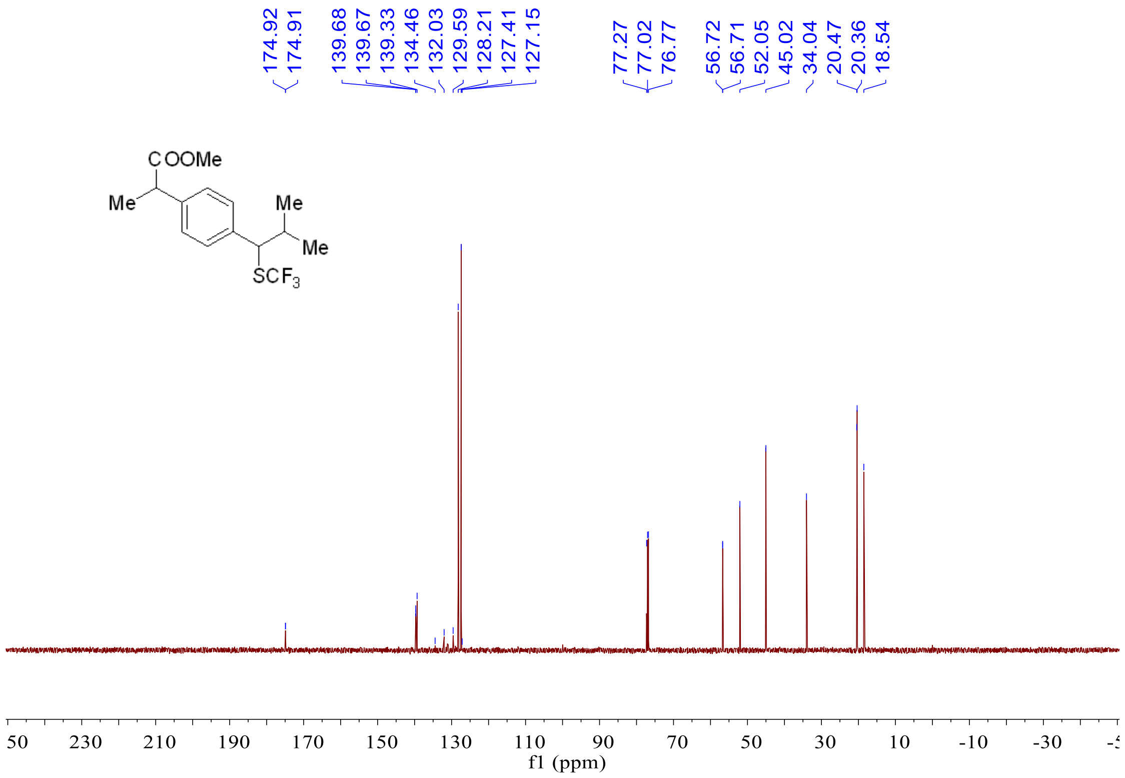

Supplementary Figure 152. ^{13}C NMR spectra for compound **11**

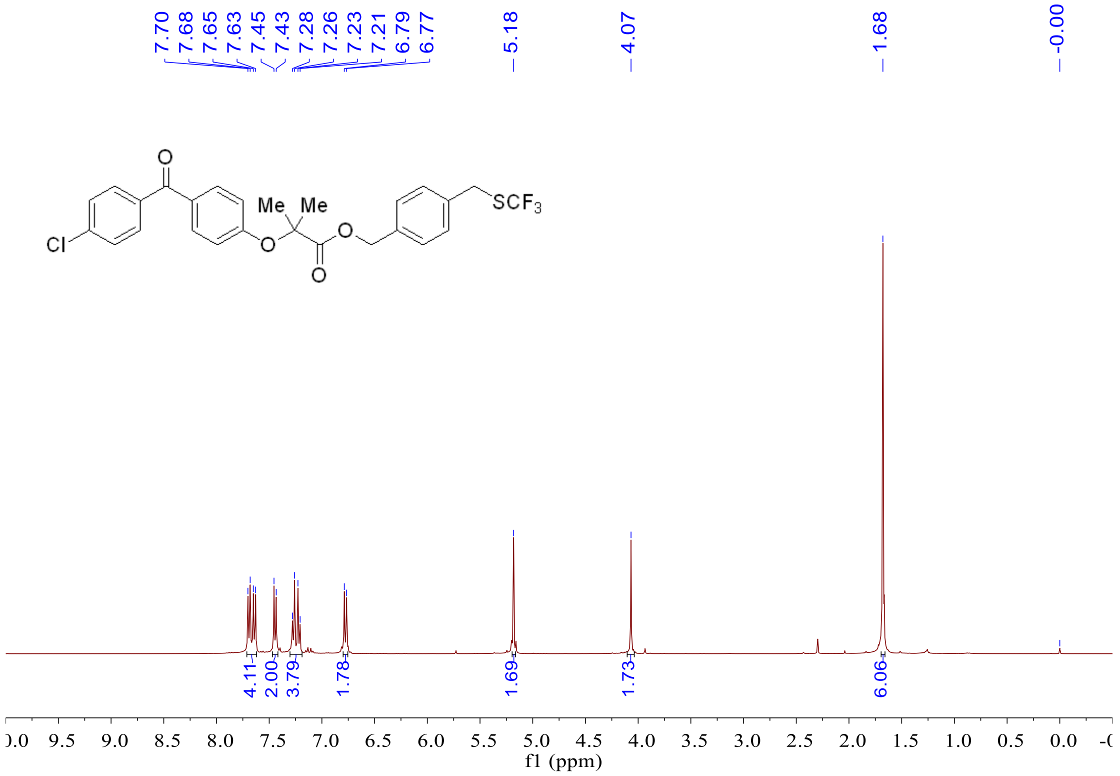

Supplementary Figure 153. ^{1}H NMR spectra for compound **12**

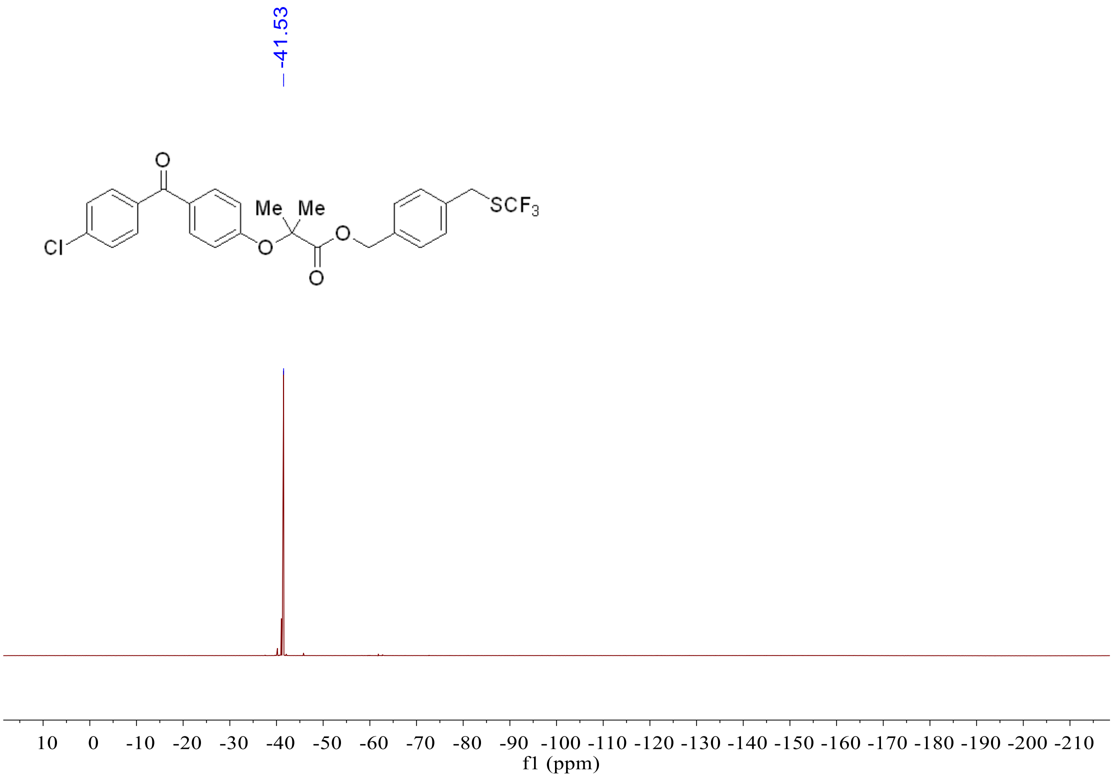

Supplementary Figure 154. ^{19}F NMR spectra for compound **12**

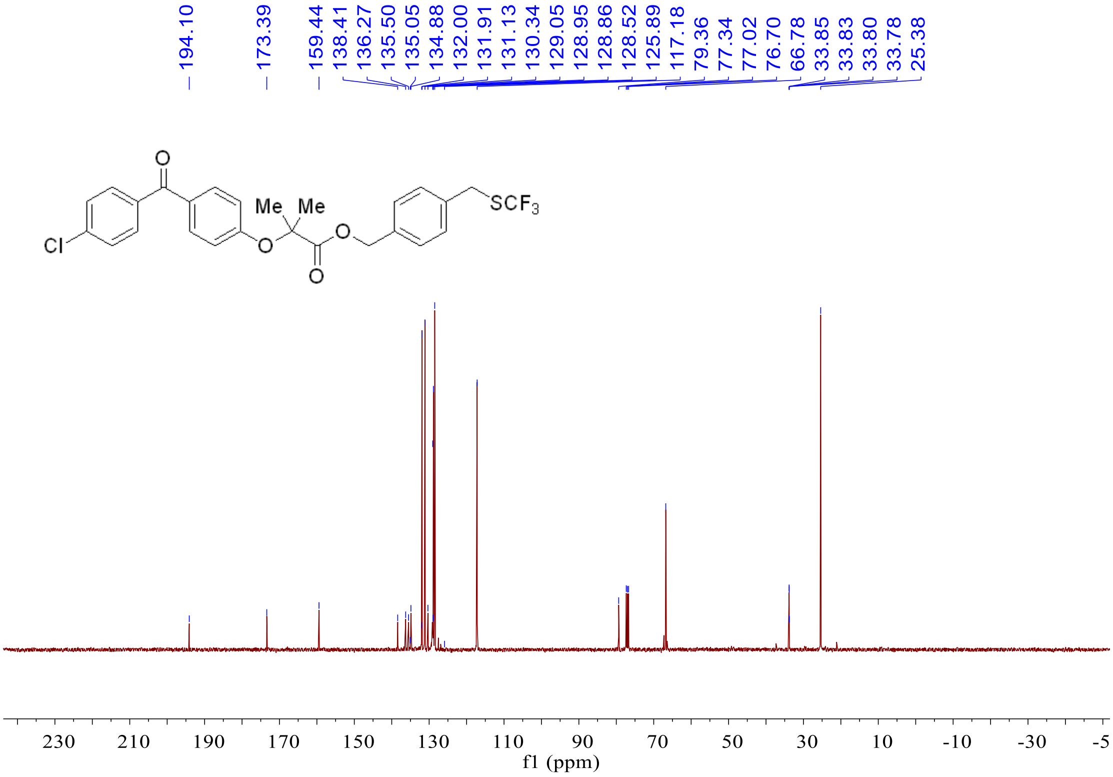

Supplementary Figure 155. ^{13}C NMR spectra for compound **12**

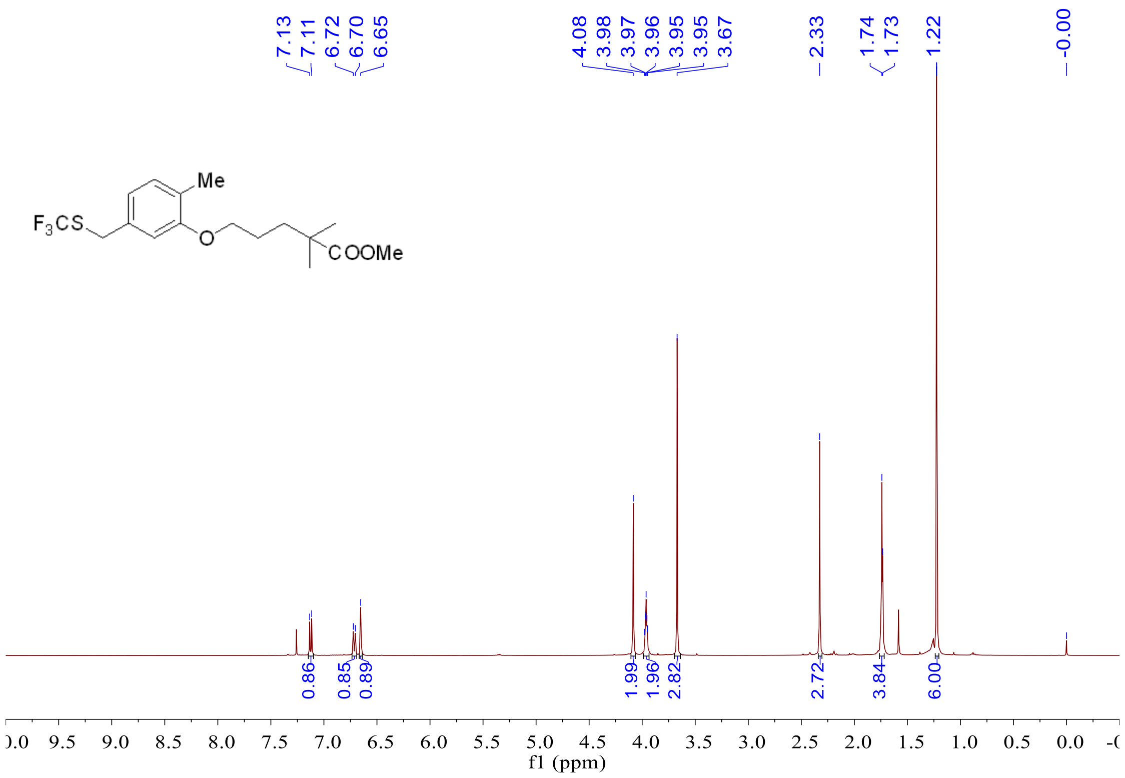

Supplementary Figure 156. ^{1}H NMR spectra for compound **13**

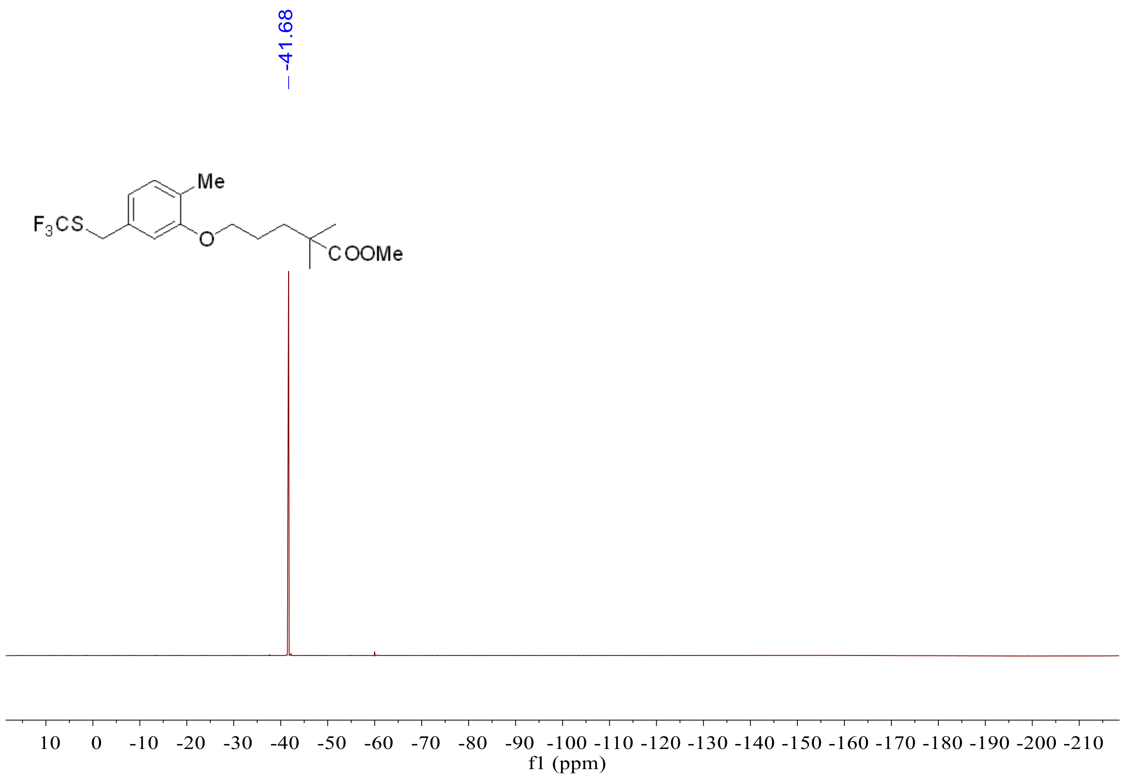

Supplementary Figure 157. ^{19}F NMR spectra for compound **13**

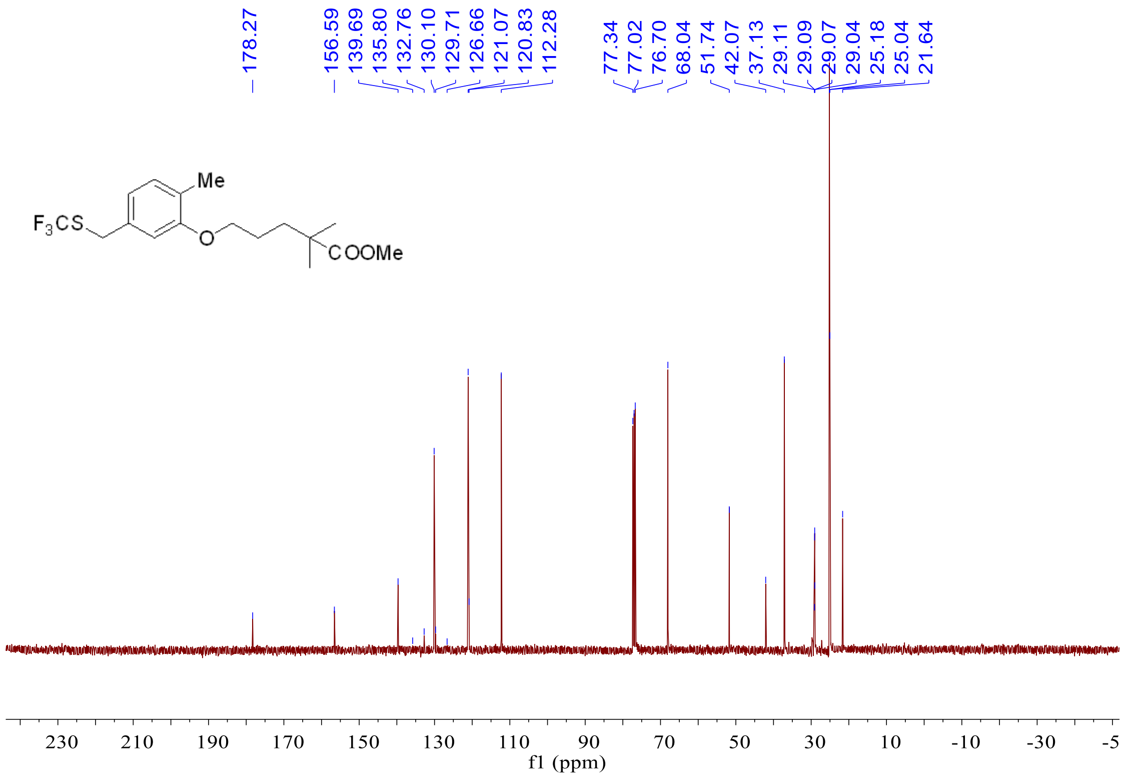

Supplementary Figure 158. ^{13}C NMR spectra for compound **13**

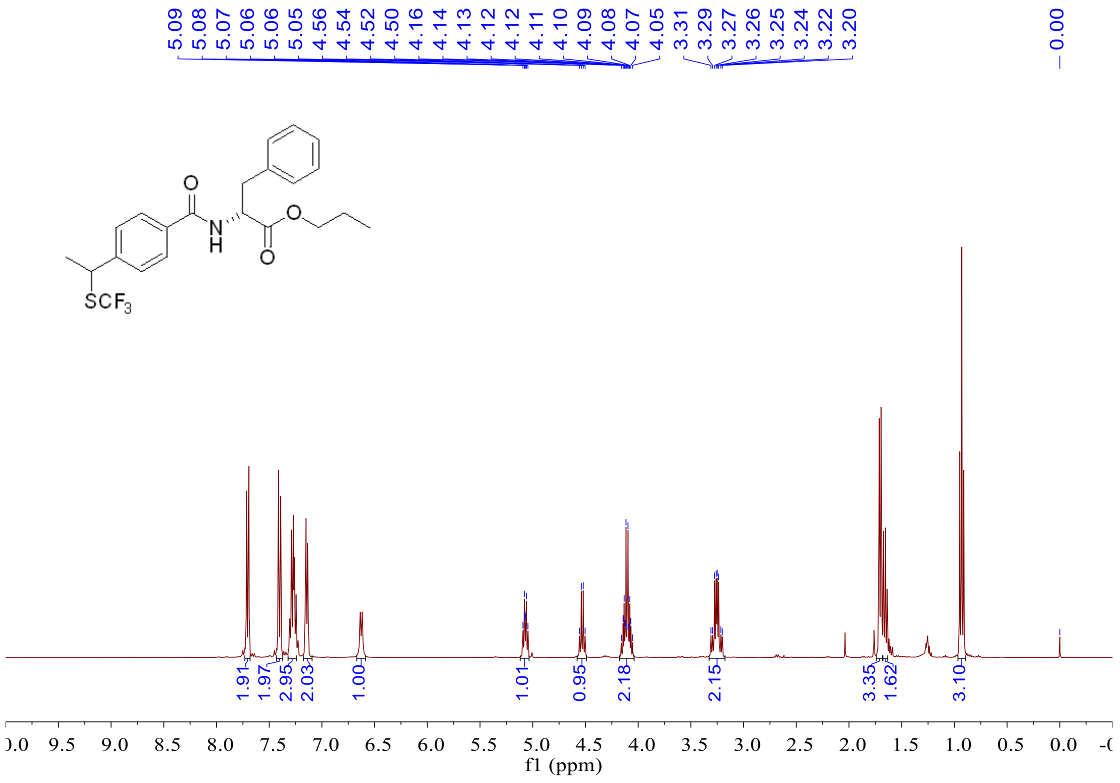

Supplementary Figure 159. ^{1}H NMR spectra for compound **14**

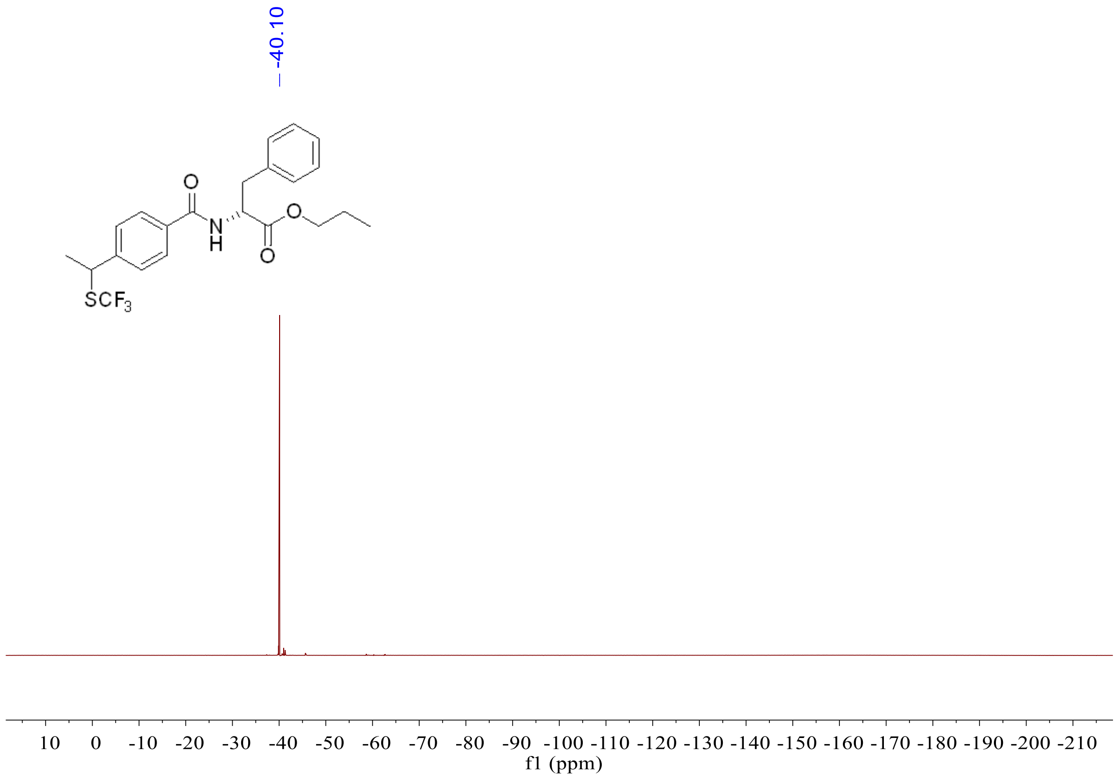

Supplementary Figure 160. ^{19}F NMR spectra for compound **14**

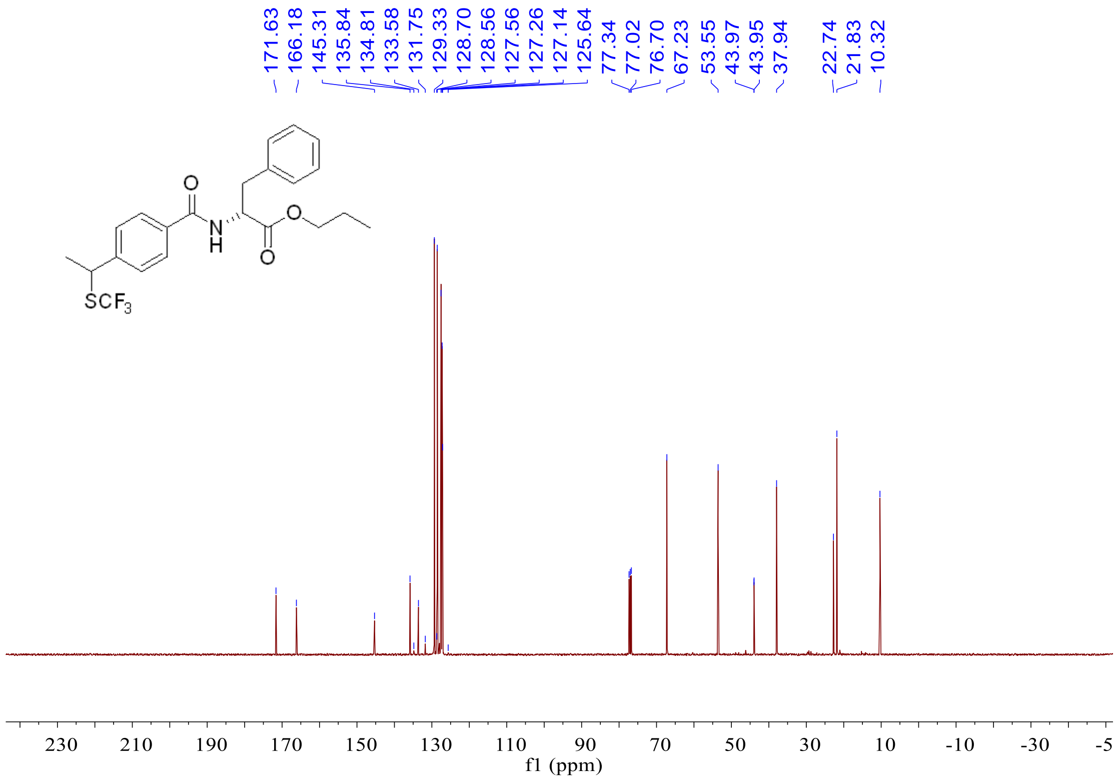

Supplementary Figure 161. ^{13}C NMR spectra for compound **14**

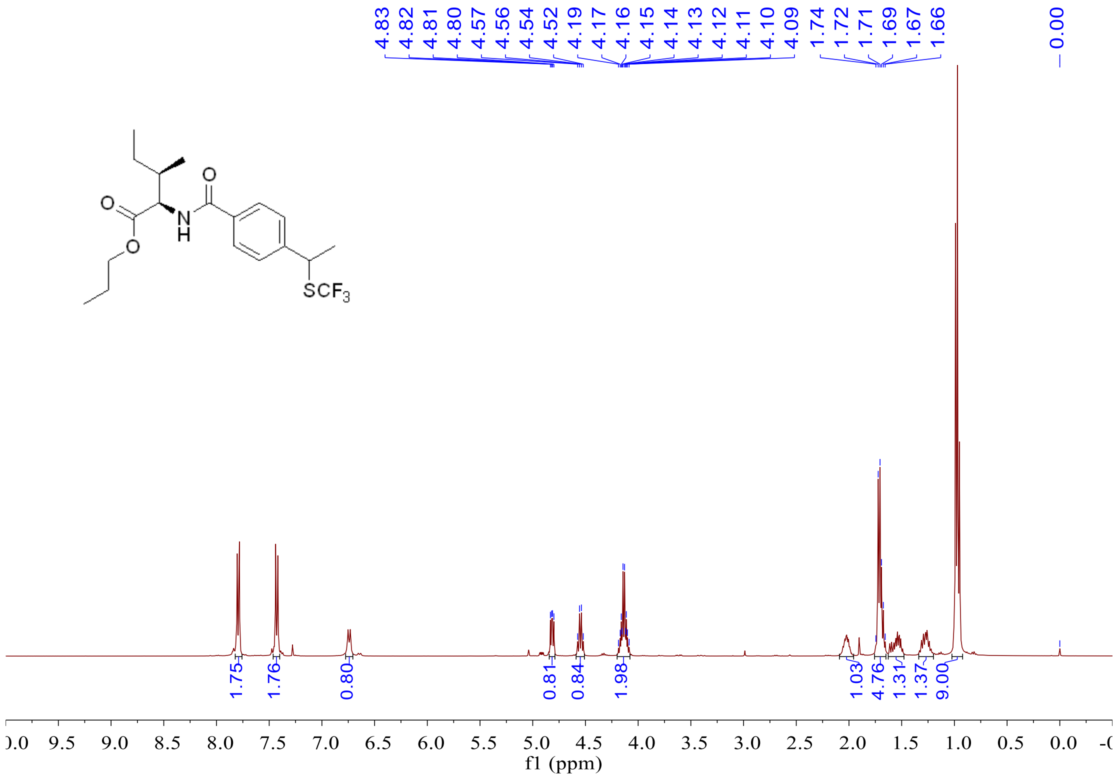

Supplementary Figure 162. ^{1}H NMR spectra for compound **15**

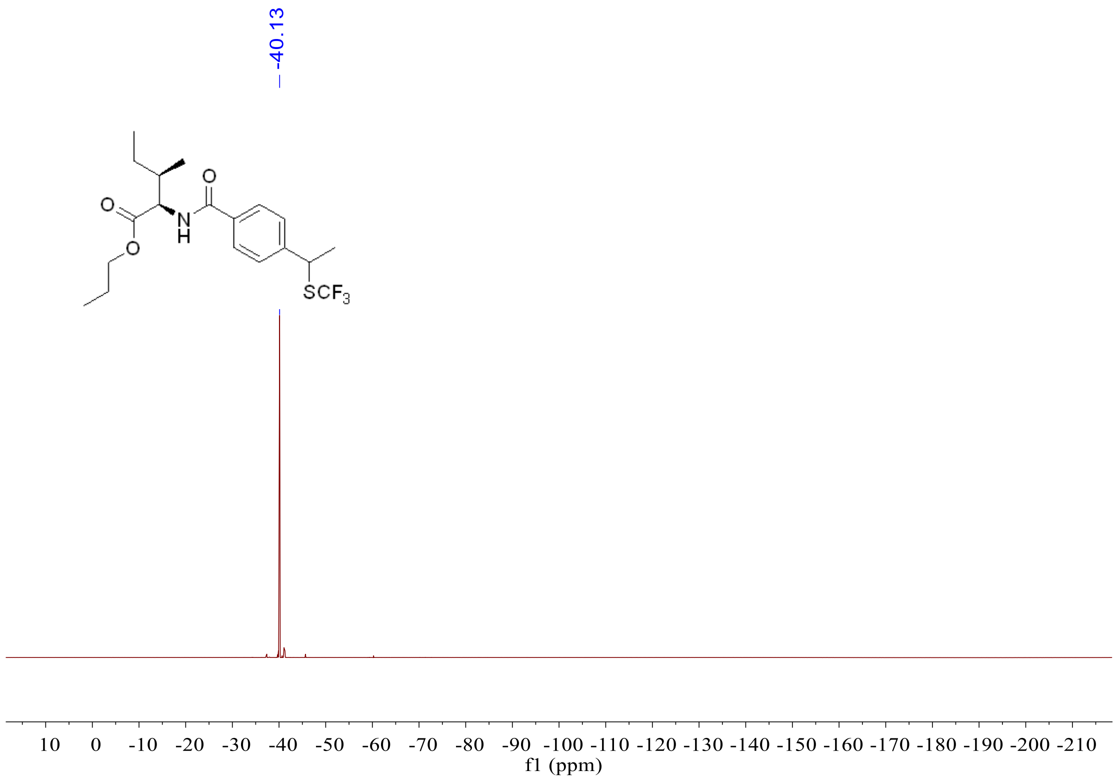

Supplementary Figure 163. ^{19}F NMR spectra for compound **15**

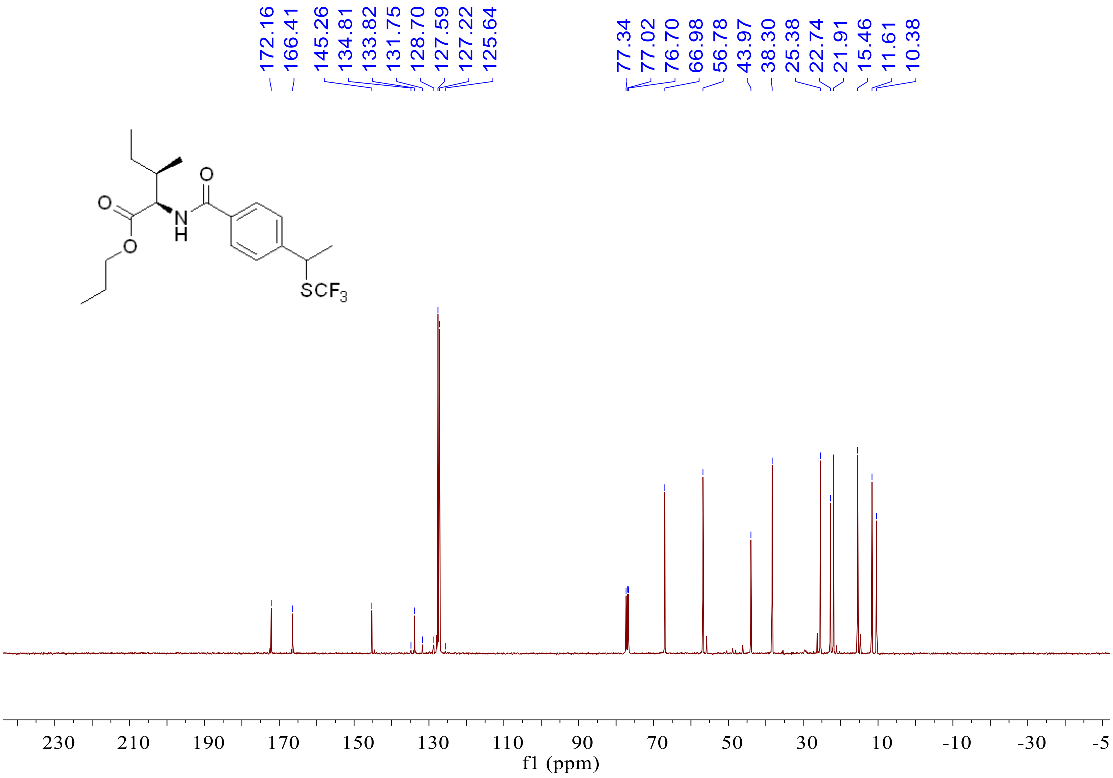

Supplementary Figure 164. ^{13}C NMR spectra for compound **15**

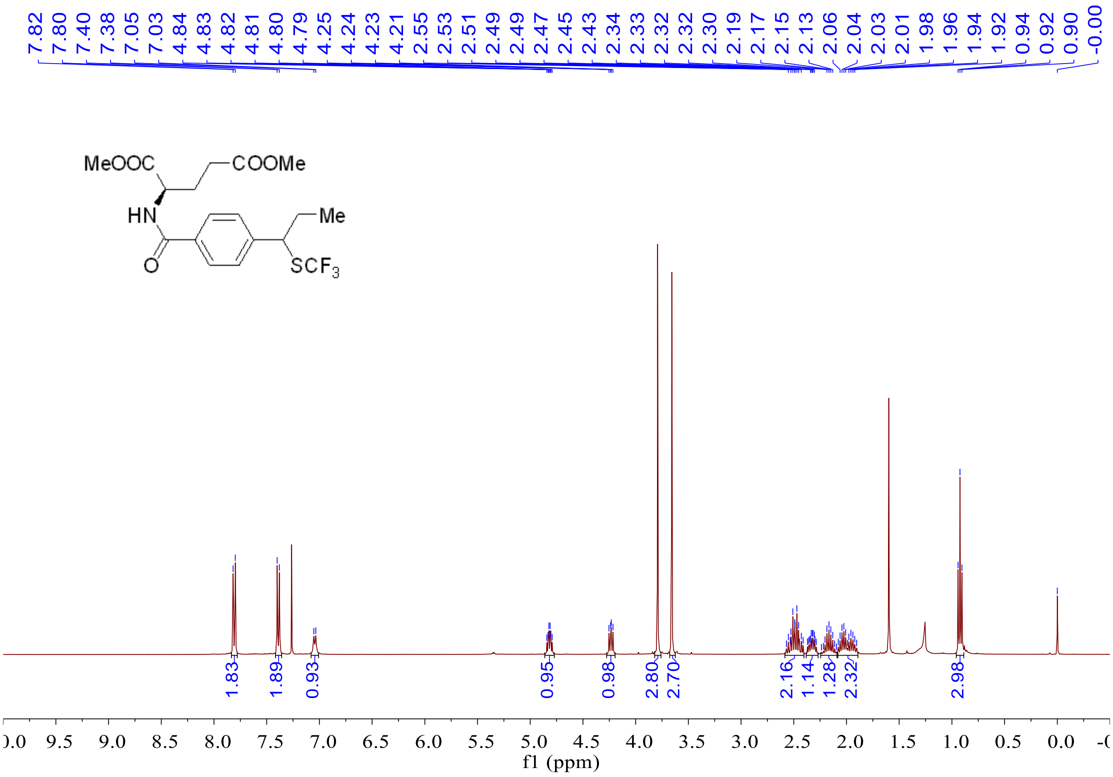

Supplementary Figure 165. ^{1}H NMR spectra for compound **16**

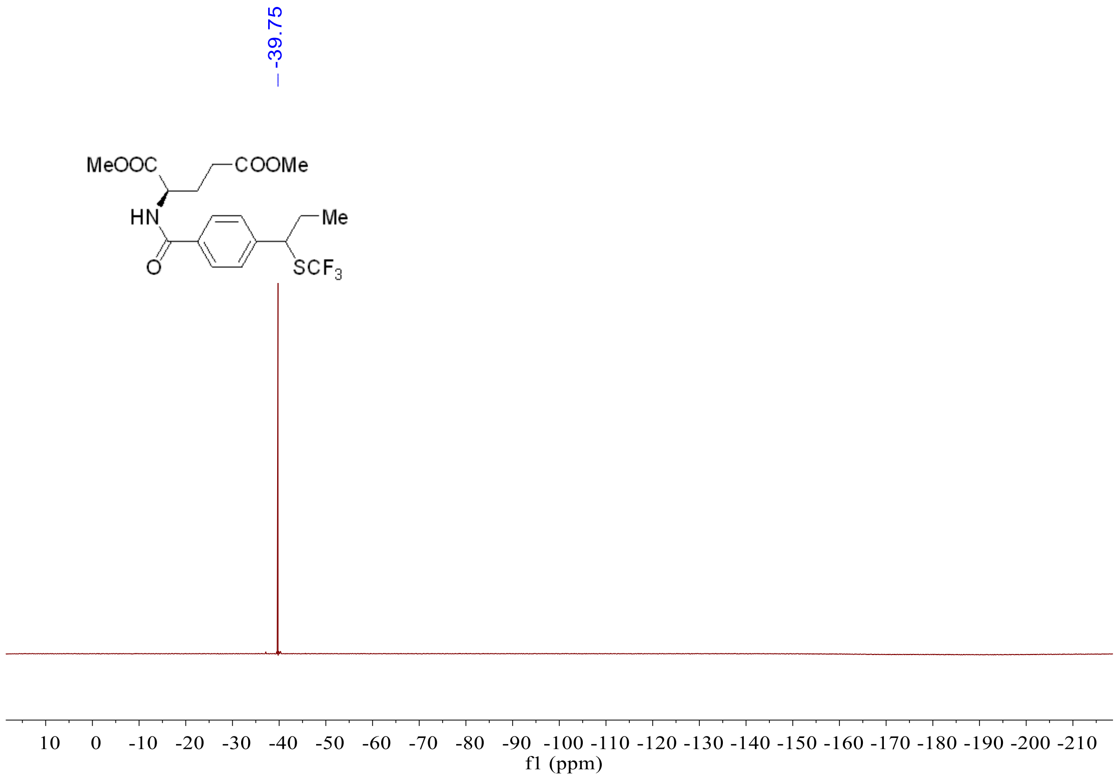

Supplementary Figure 166. ^{19}F NMR spectra for compound **16**

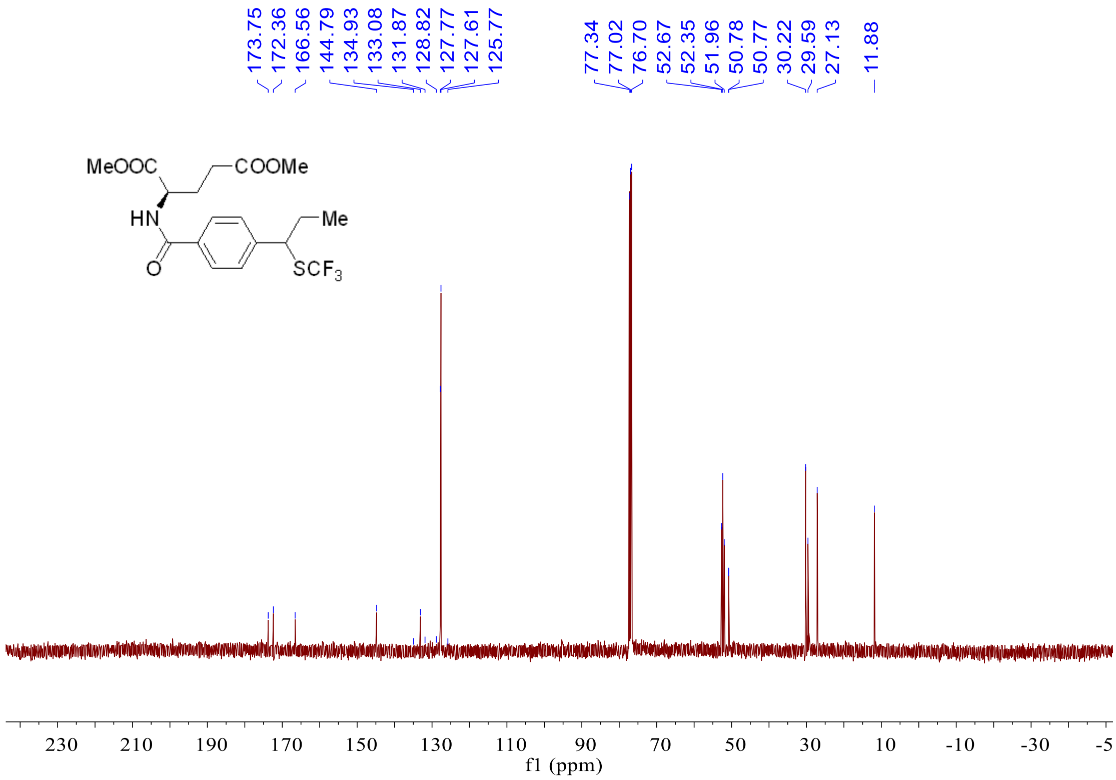

Supplementary Figure 167. ^{13}C NMR spectra for compound **16**

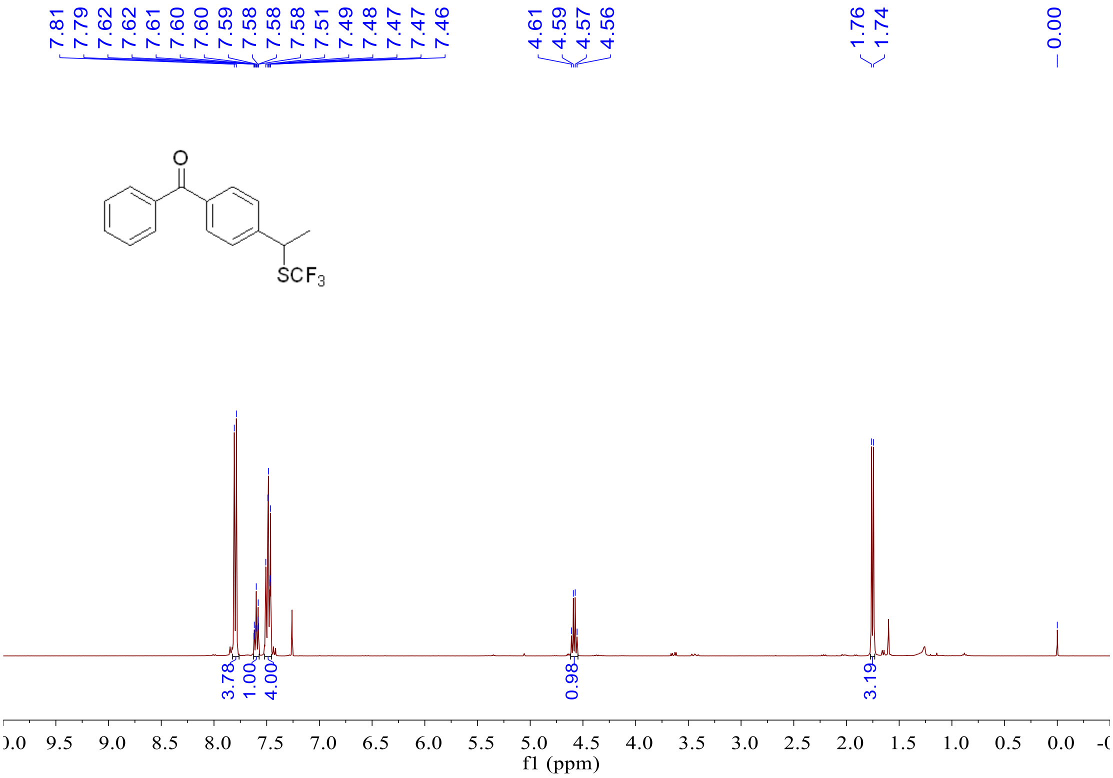

Supplementary Figure 168. ^{1}H NMR spectra for compound **17**

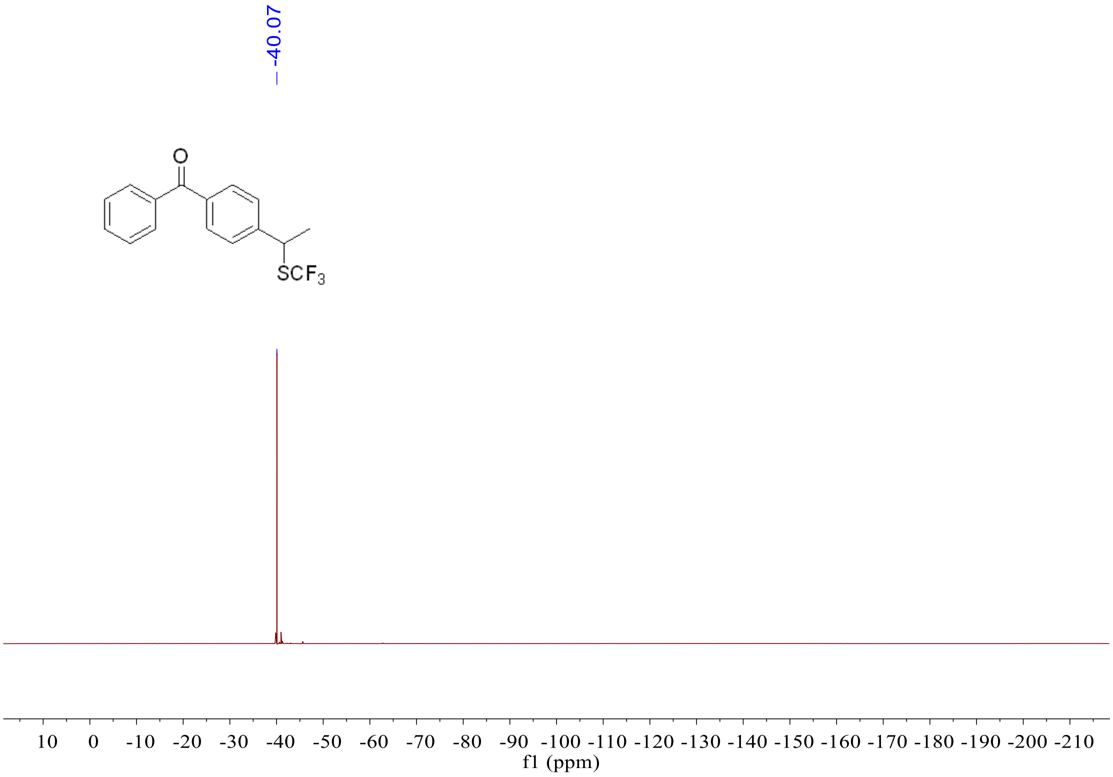

Supplementary Figure 169. ^{19}F NMR spectra for compound **17**

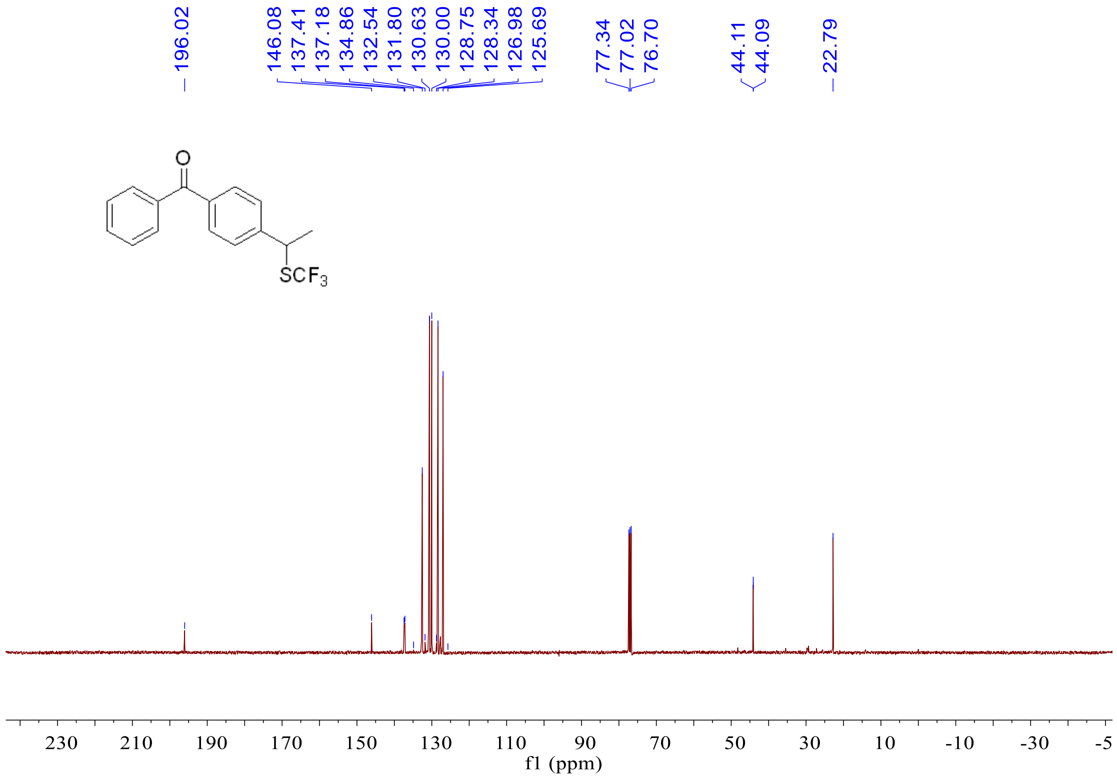

Supplementary Figure 170. ^{13}C NMR spectra for compound **17**

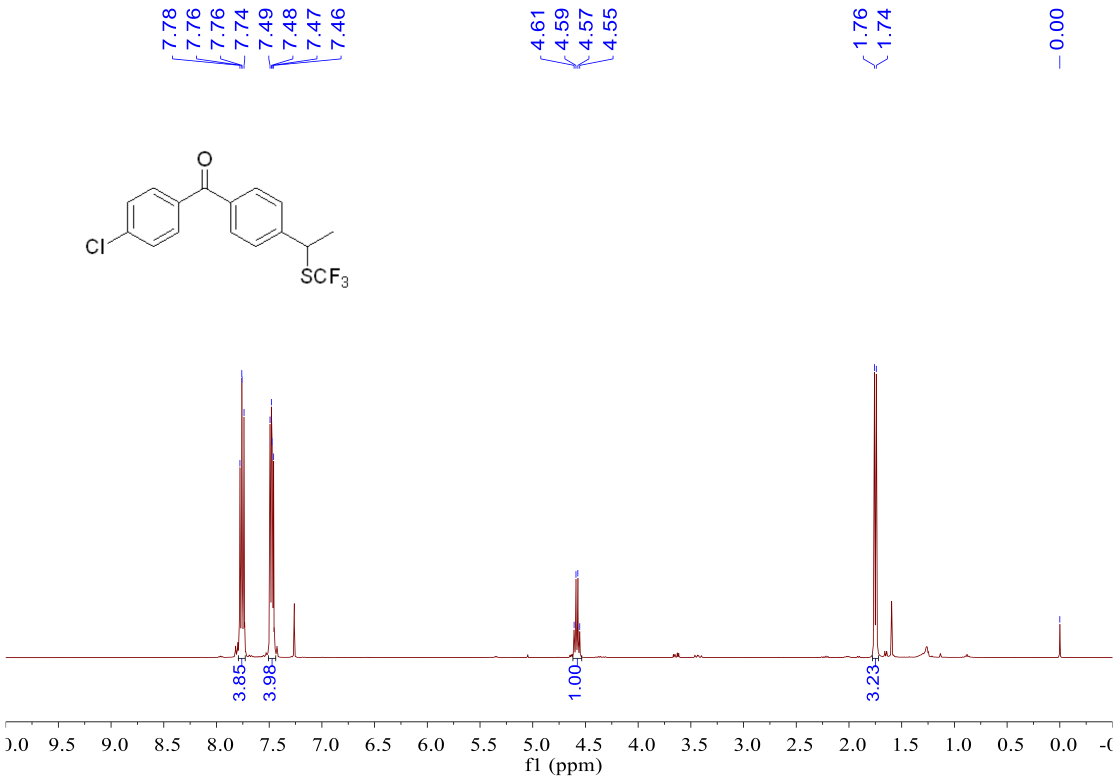

Supplementary Figure 171. ^{1}H NMR spectra for compound **18**

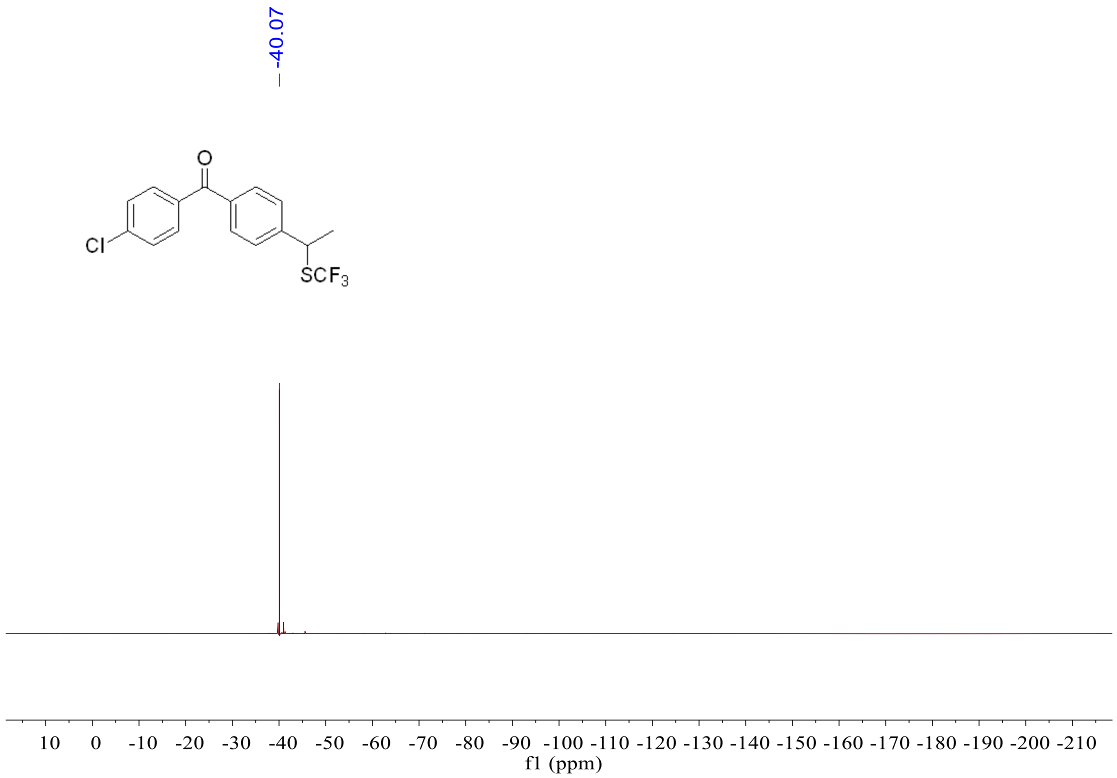

Supplementary Figure 172. ^{19}F NMR spectra for compound **18**

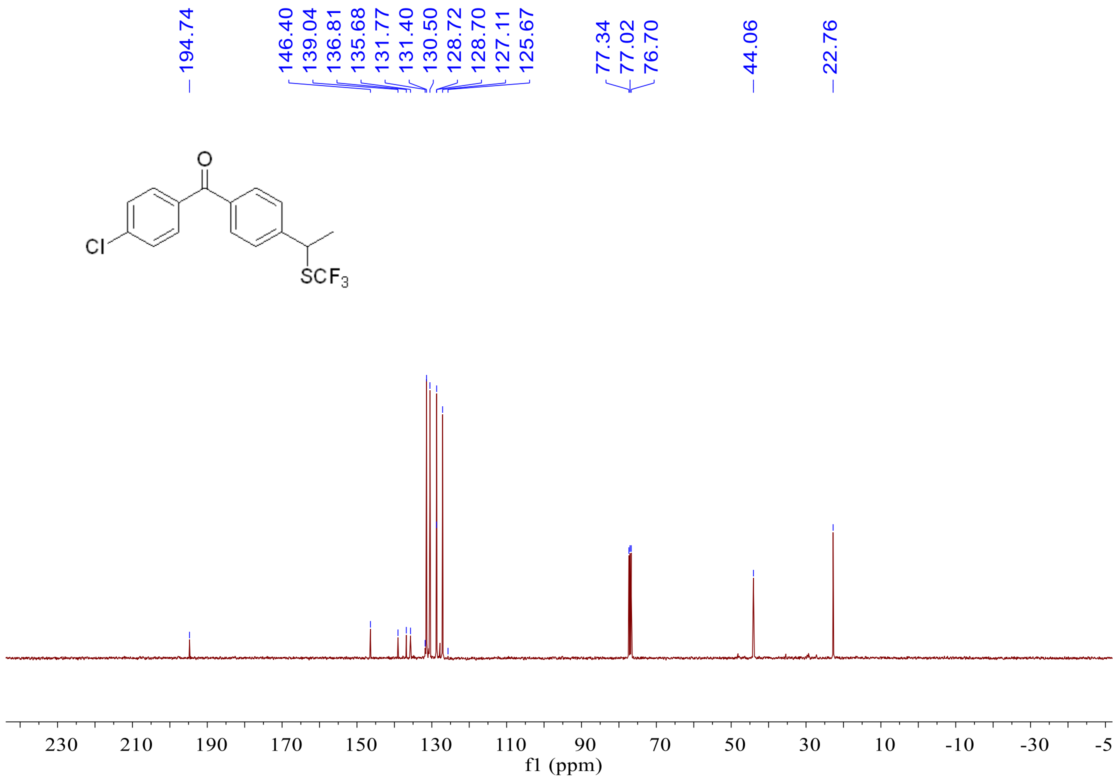

Supplementary Figure 173. ^{13}C NMR spectra for compound **18**

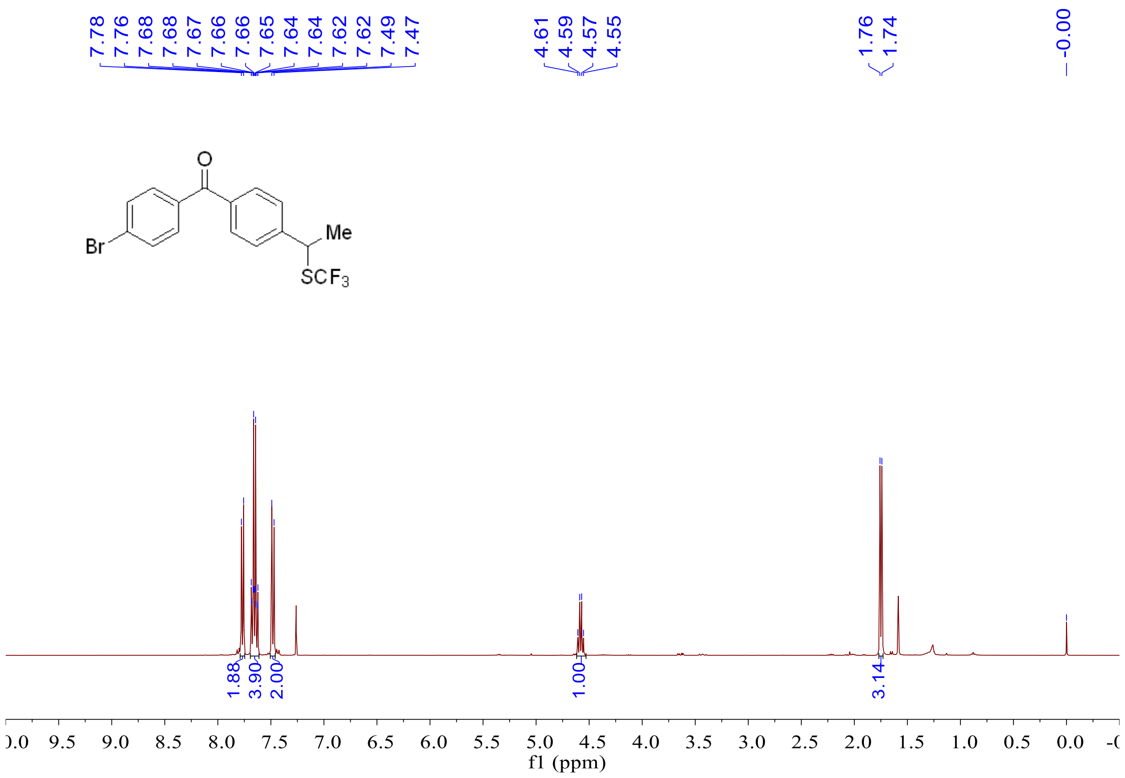

Supplementary Figure 174. ^{1}H NMR spectra for compound **19**

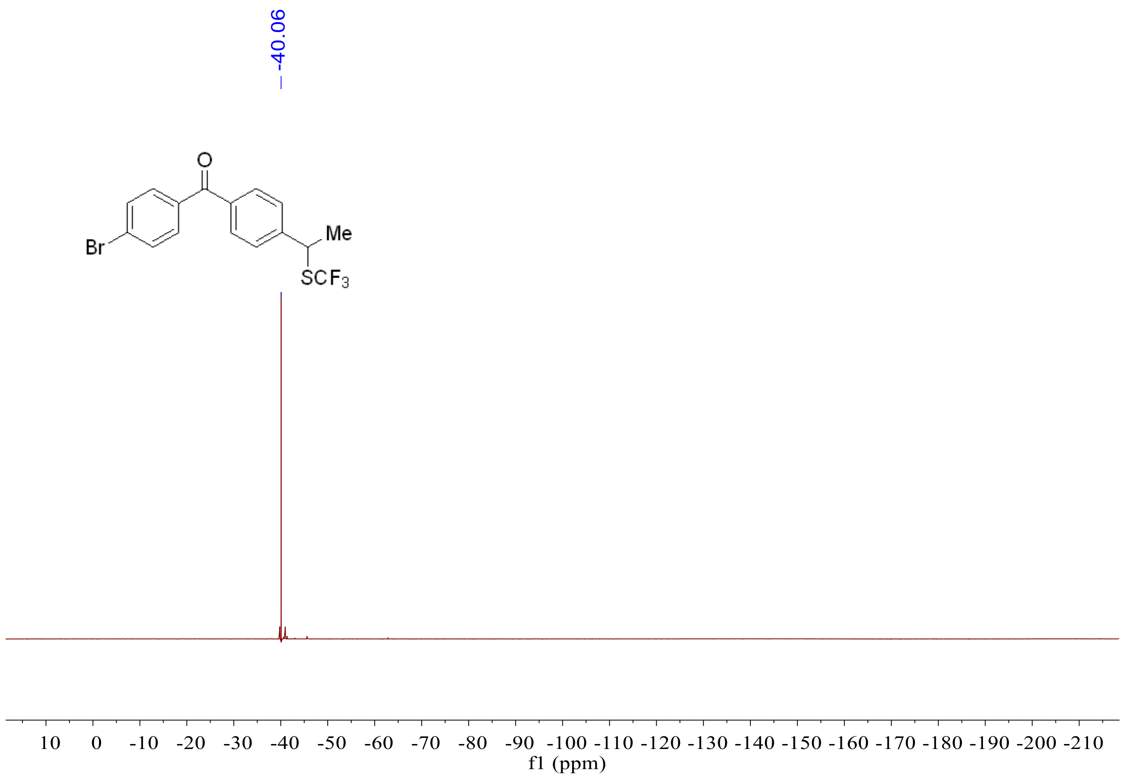

Supplementary Figure 175. ^{19}F NMR spectra for compound **19**

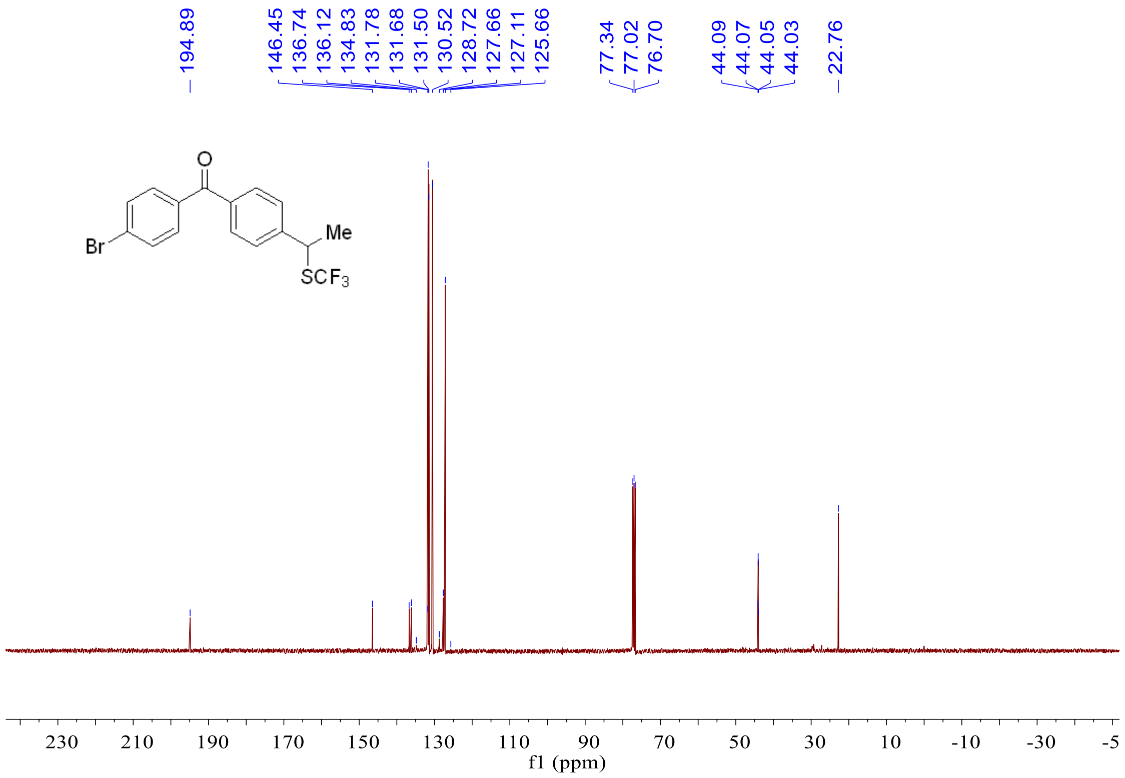

Supplementary Figure 176. ^{13}C NMR spectra for compound **19**

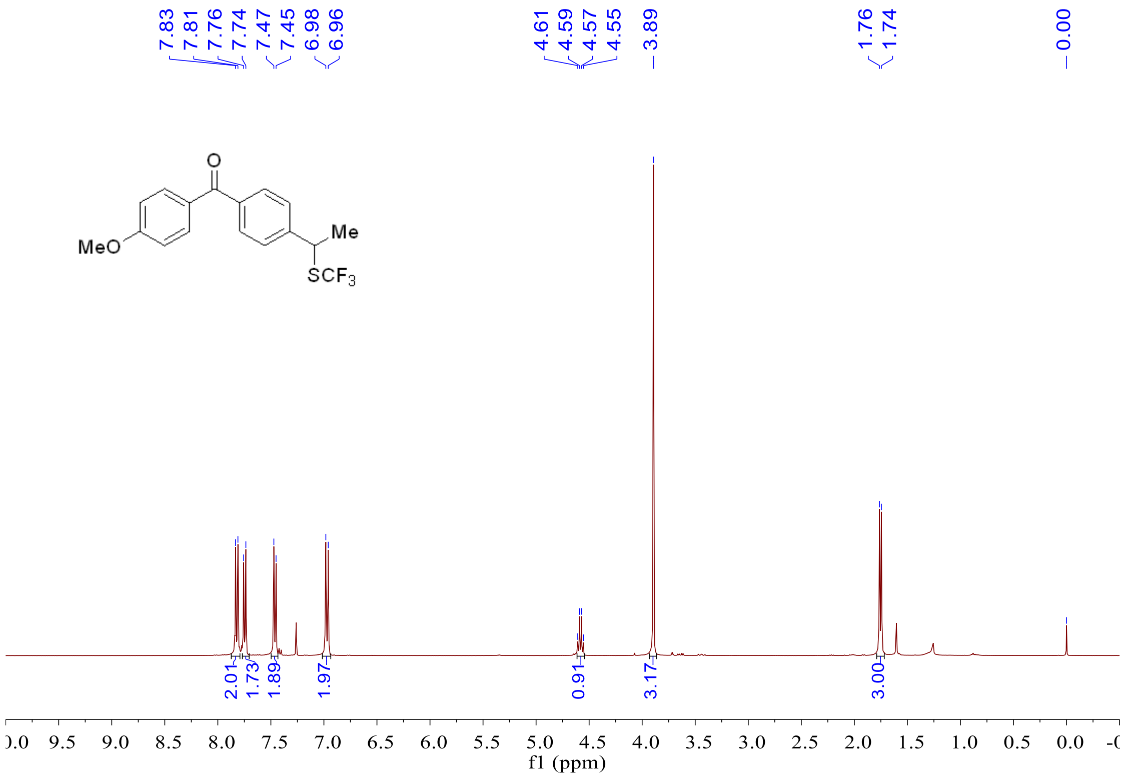

Supplementary Figure 177. ^{1}H NMR spectra for compound **20**

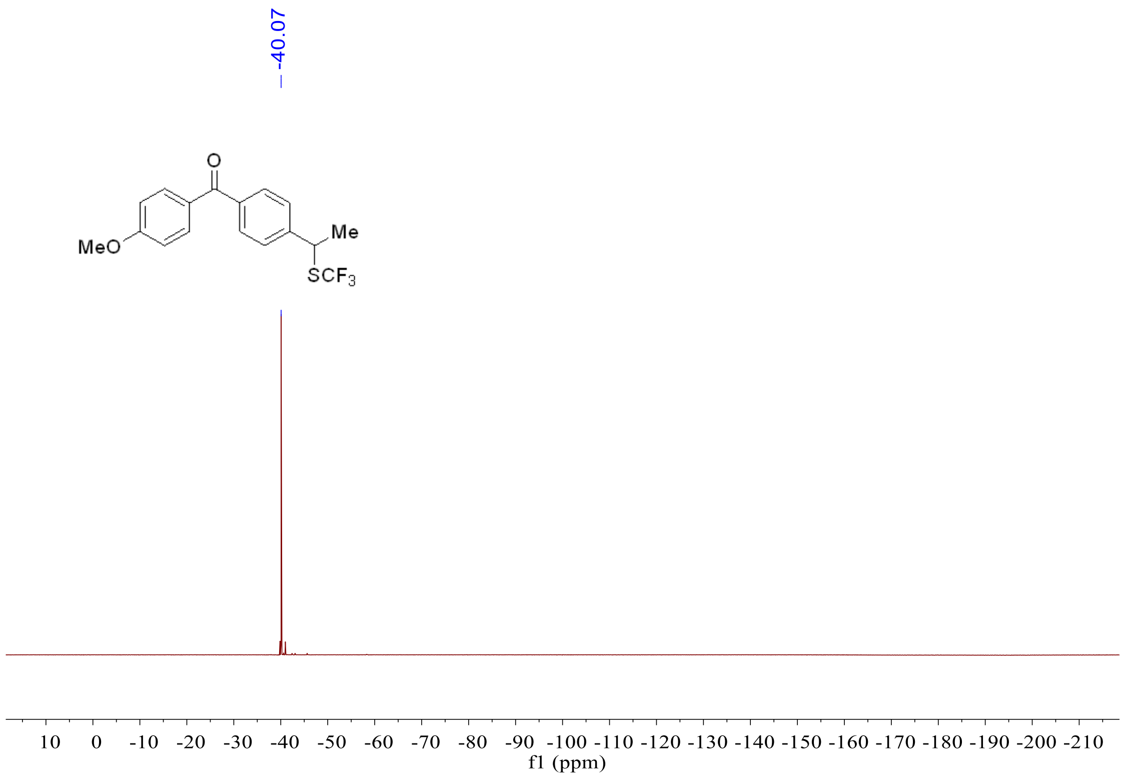

Supplementary Figure 178. ^{19}F NMR spectra for compound **20**

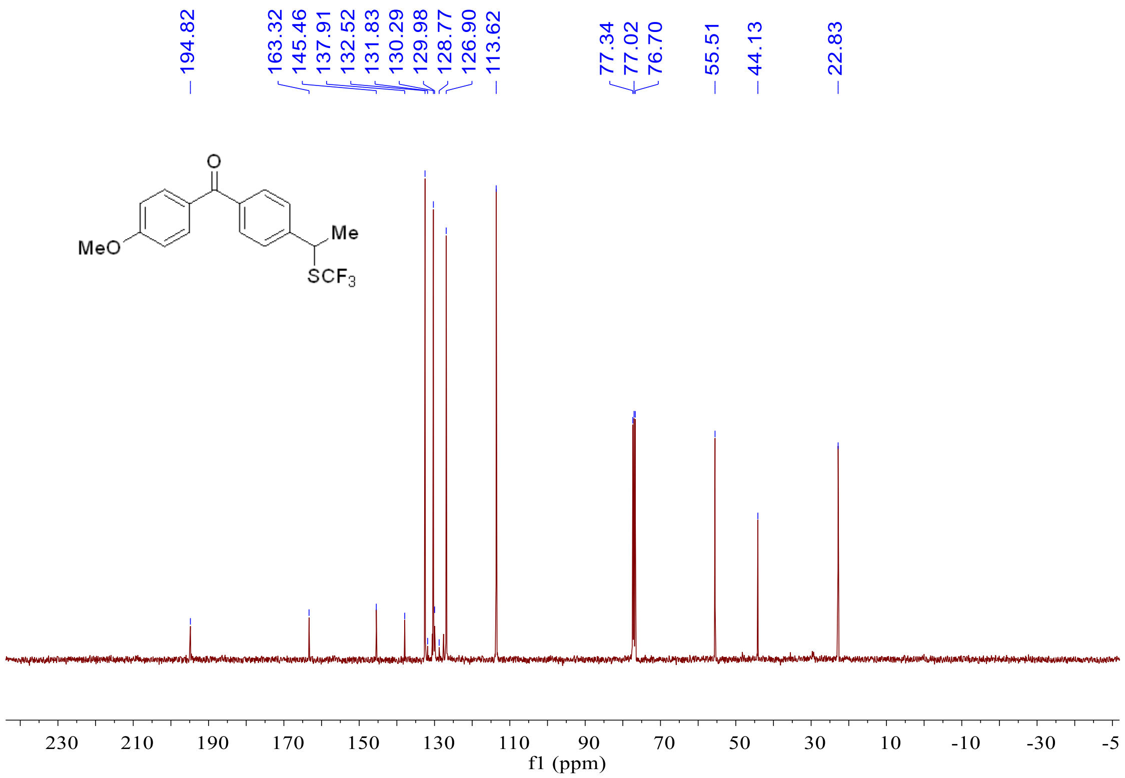

Supplementary Figure 179. ^{13}C NMR spectra for compound **20**

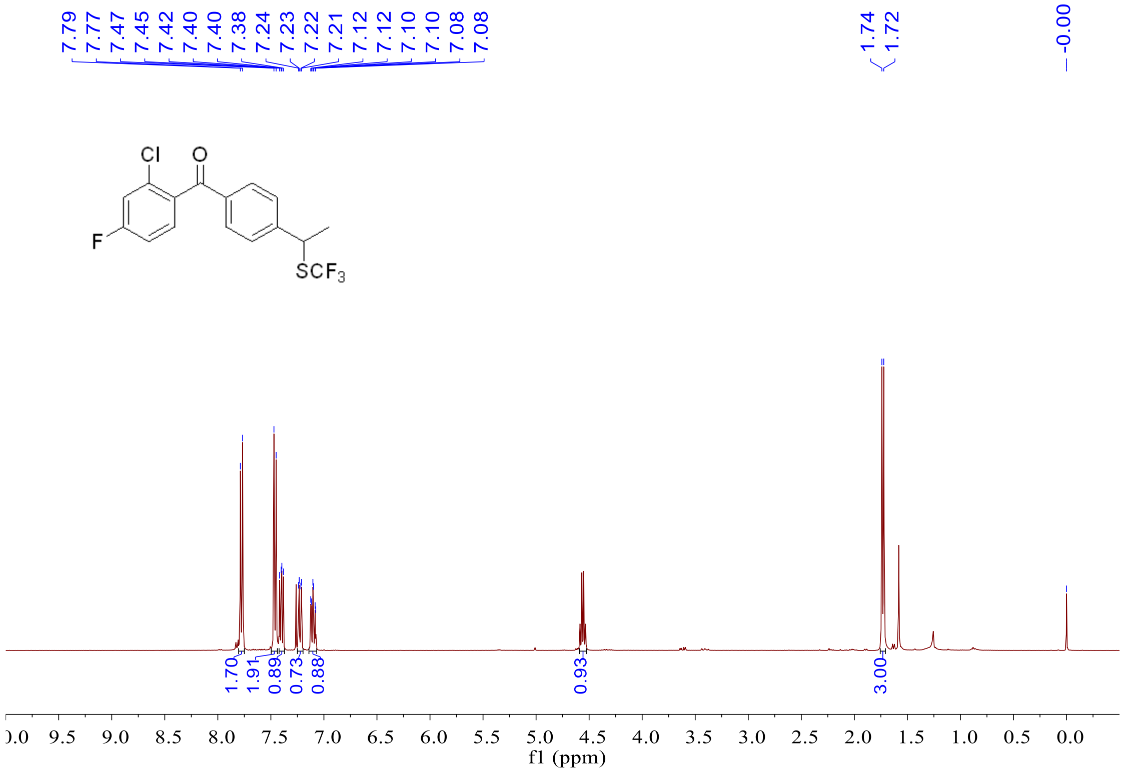

Supplementary Figure 180. ^{1}H NMR spectra for compound **21**

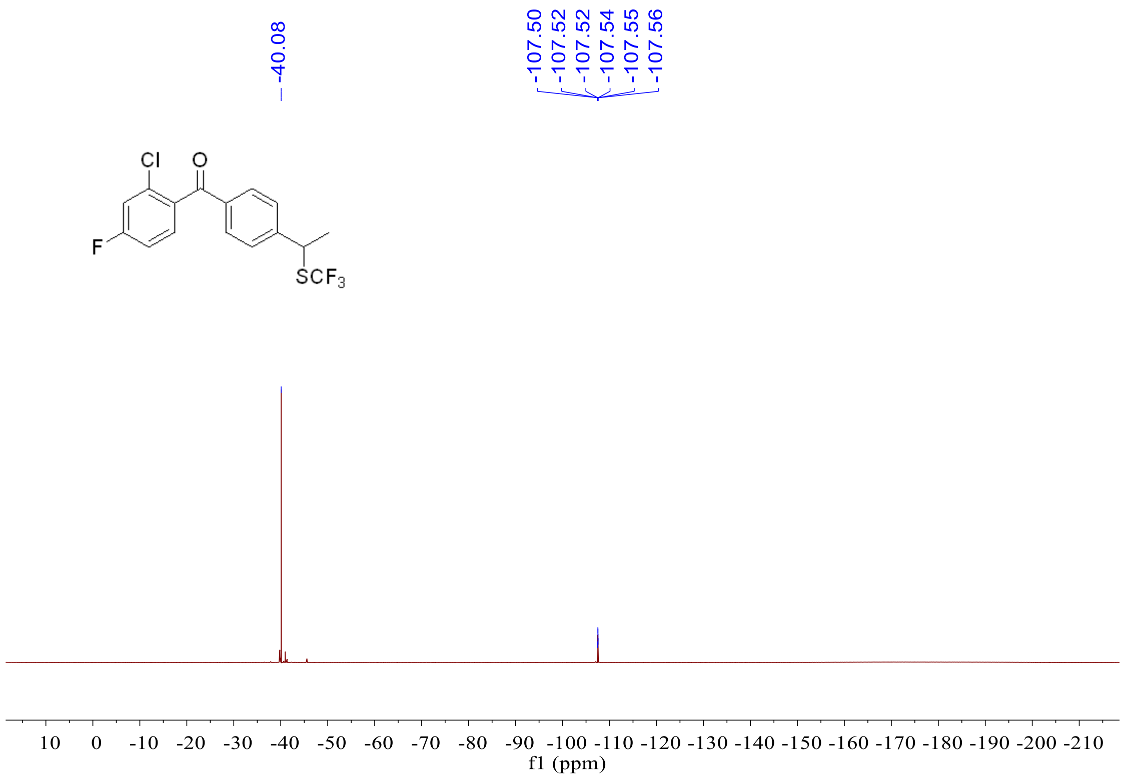

Supplementary Figure 181. ^{19}F NMR spectra for compound **21**

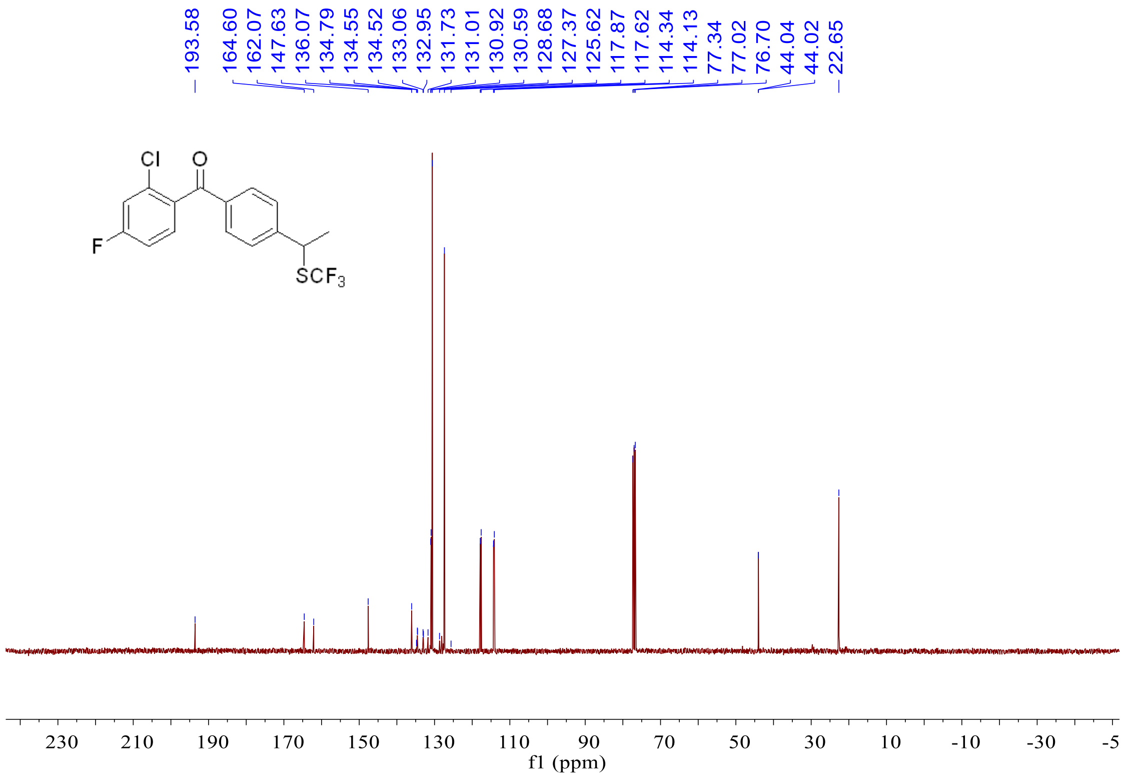

Supplementary Figure 182. ^{13}C NMR spectra for compound **21**

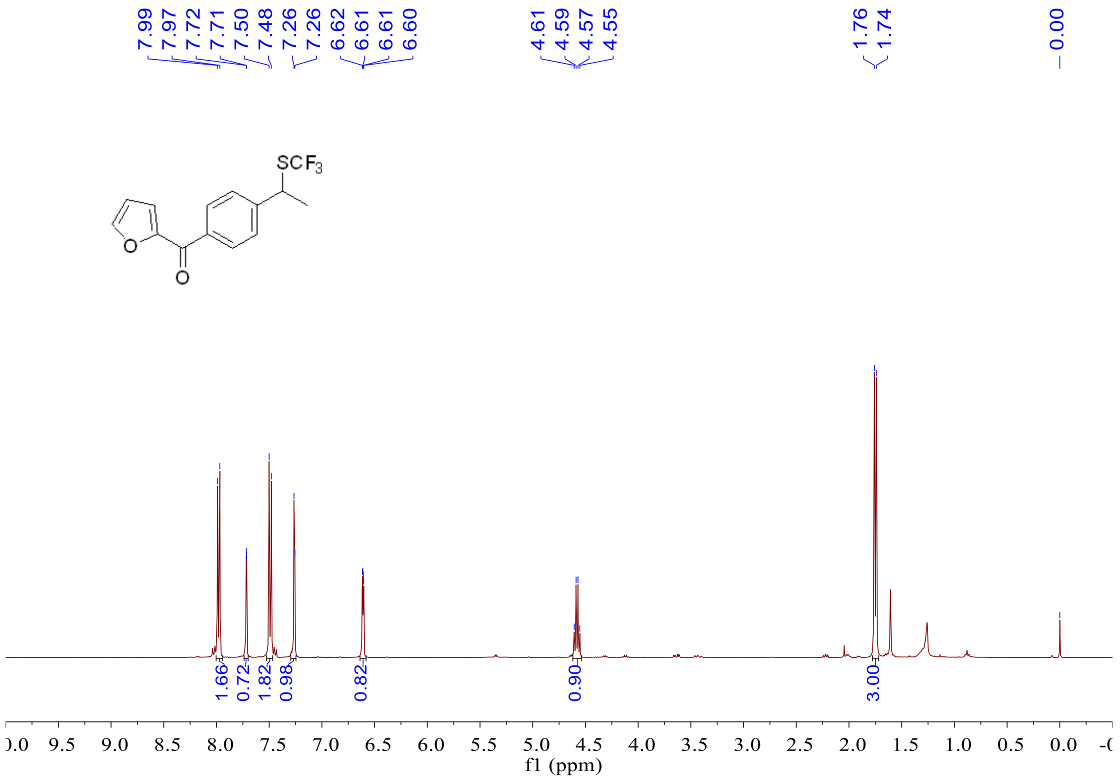

Supplementary Figure 183. ^{1}H NMR spectra for compound **22**

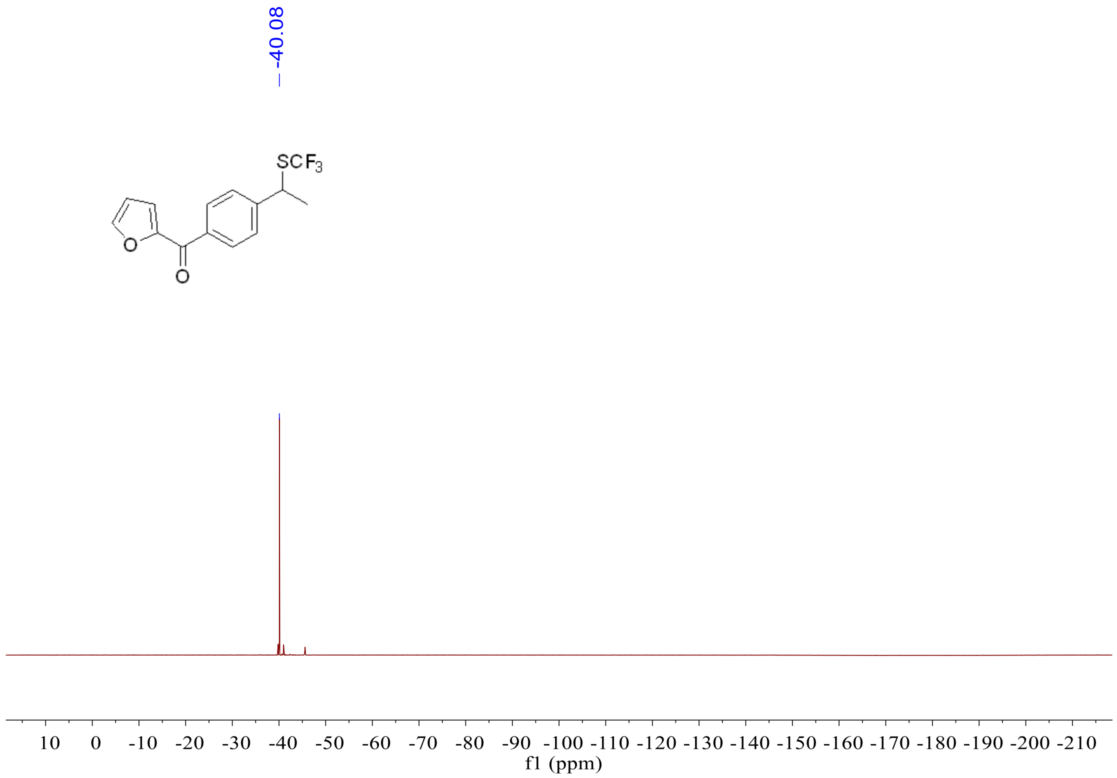

Supplementary Figure 184. ^{19}F NMR spectra for compound **22**

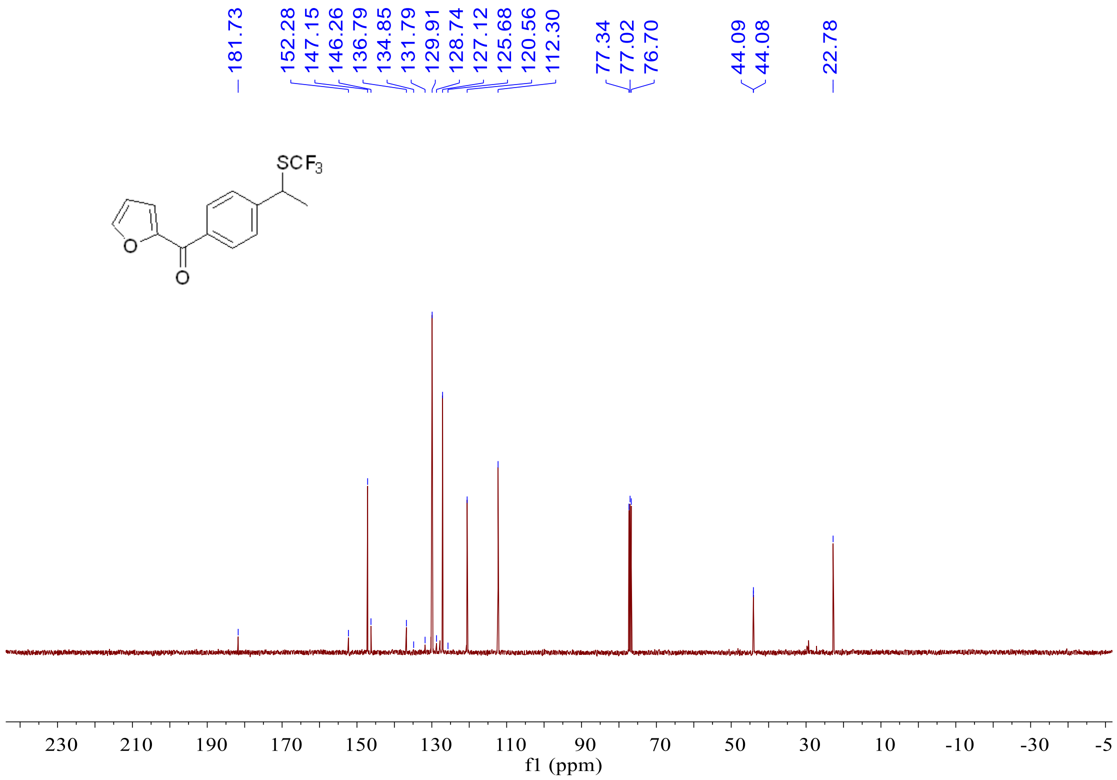

Supplementary Figure 185. ^{13}C NMR spectra for compound **22**

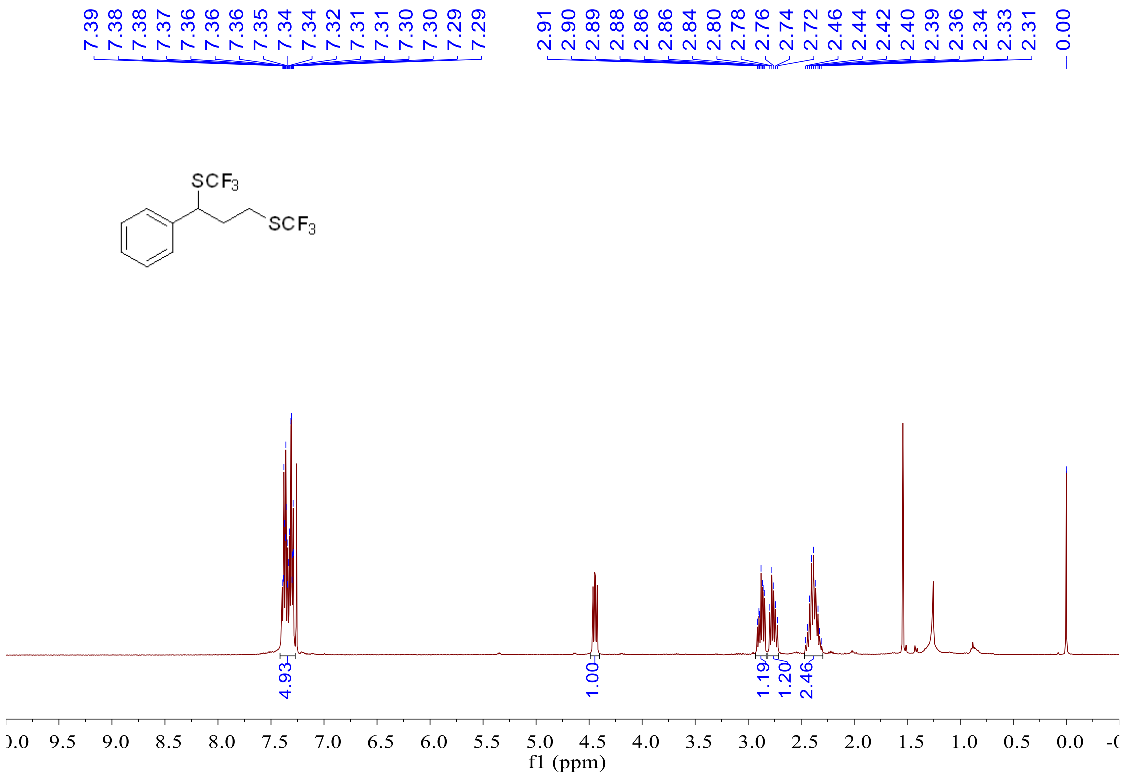

Supplementary Figure 186. ^{1}H NMR spectra for compound **23**

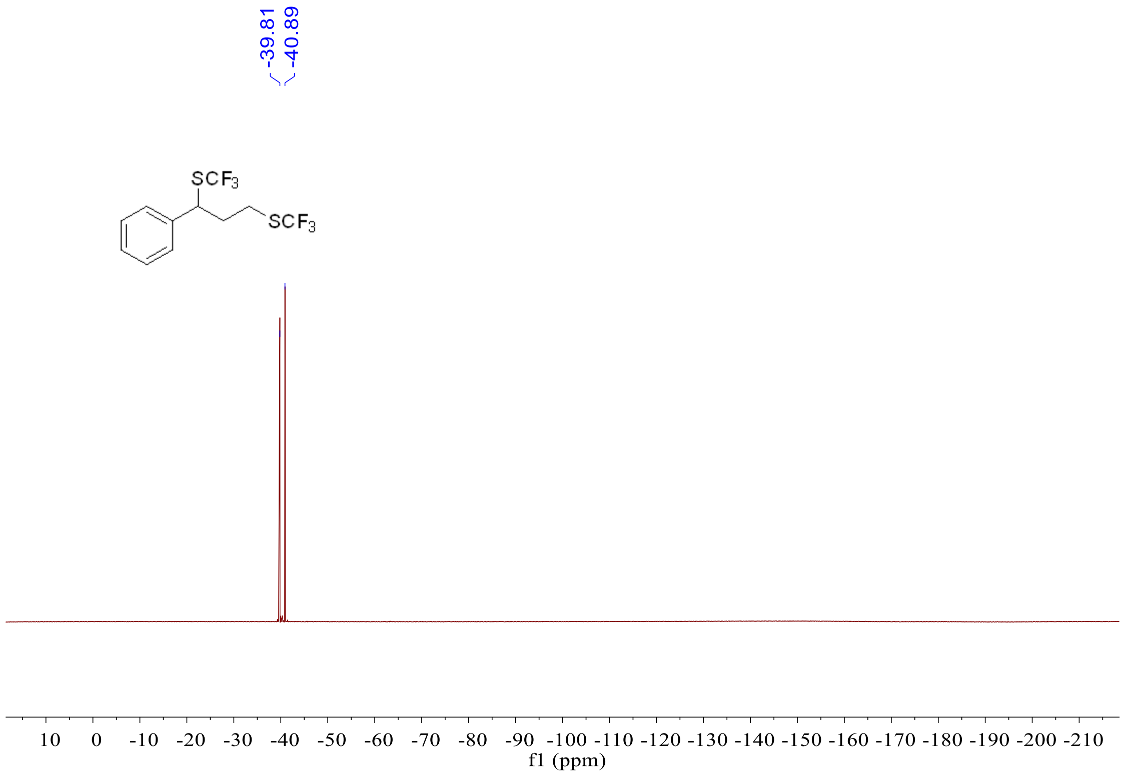

Supplementary Figure 187. ^{19}F NMR spectra for compound **23**

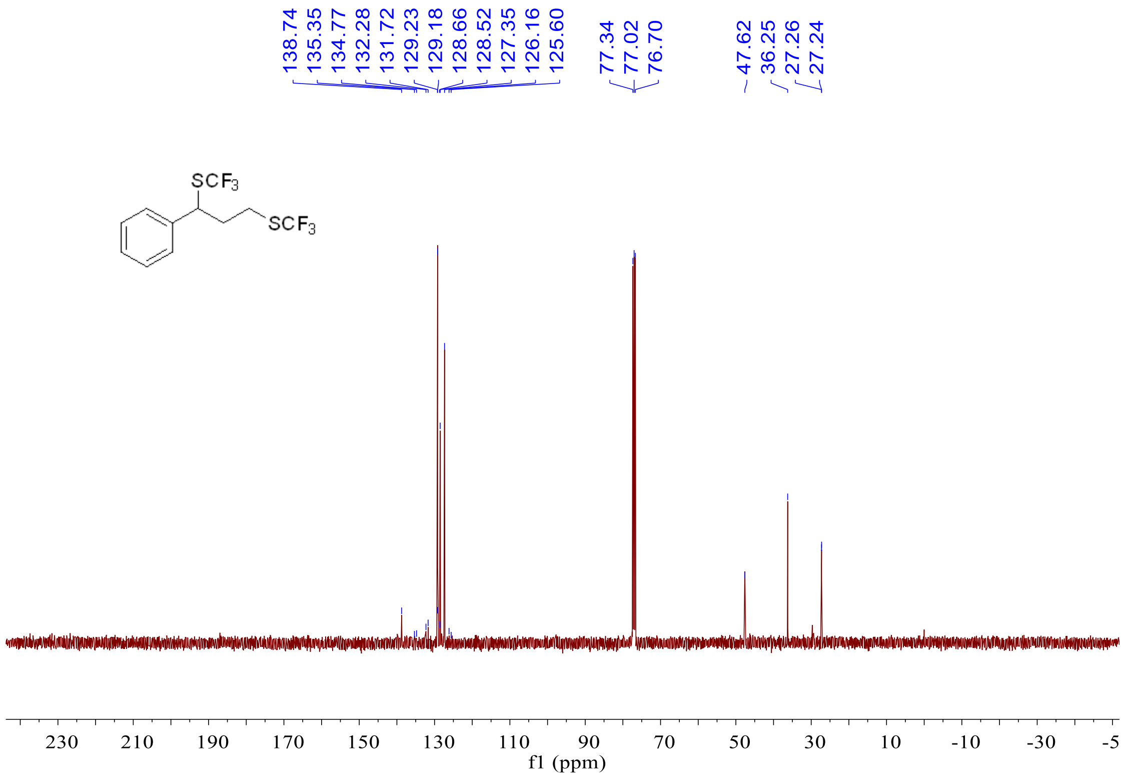

Supplementary Figure 188. ^{13}C NMR spectra for compound **23**

Supplementary Reference

1. Liu J. B., Xu X. H., Chen Z. H. & Qing F. L. Direct dehydroxytrifluoromethylthiolation of alcohols using silver(I) trifluoromethanethiolate and tetra-N-butylammonium iodide. *Angew. Chem. Int. Ed.* **54**, 897-900 (2015).
2. Garreau M., Le Vaillant F. & Waser J. C-terminal bioconjugation of peptides through photoredox catalyzed decarboxylative alkynylation. *Angew. Chem. Int. Ed.* **58**, 8182-8186 (2019).
3. Howell J. M., Feng K., Clark J. R., Trzepkowski L. J. & White M. C. Remote oxidation of aliphatic C-H bonds in nitrogen-containing molecules. *J. Am. Chem. Soc.* **137**, 14590-14593 (2015).
4. Phipps R. J., Grimster N. P. & Gaunt M. J. Cu(II)-catalyzed direct and site-selective arylation of indoles under mild conditions. *J. Am. Chem. Soc.* **130**, 8172-8174 (2008).
5. Everson D. A., Shrestha R. & Weix D. J. Nickel-catalyzed reductive cross-coupling of aryl halides with alkyl halides. *J. Am. Chem. Soc.* **132**, 920-921 (2010).
